# Tetrafluoropyridyl (TFP): a general phenol protecting group readily cleaved under mild conditions†
†Electronic supplementary information (ESI) available. CCDC 1856218–1856219. For ESI and crystallographic data in CIF or other electronic format see DOI: 10.1039/c8ob02899k


**DOI:** 10.1039/c8ob02899k

**Published:** 2019-01-09

**Authors:** William D. G. Brittain, Steven L. Cobb

**Affiliations:** a Department of Chemistry , Durham University , South Road , Durham , DH1 3LE , UK . Email: william.d.brittain@durham.ac.uk ; Email: s.l.cobb@durham.ac.uk

## Abstract

Phenols are extremely valuable building blocks in the areas of pharmaceuticals, natural products, materials and catalysts. In order to carry out modifications on phenols, the phenolic oxygen is routinely protected to prevent unwanted side reactions. Presently many of the protecting groups available can require harsh conditions, specialist equipment, expensive or air/moisture-sensitive reagents to install and remove. Here we introduce the use of the tetrafluoropyridyl (TFP) group as a general protecting group for phenols. TFP can be installed in one step with no sensitivity to water or air, and it is stable under a range of commonly employed reaction conditions including acid and base. The TFP protecting group is readily cleaved under mild conditions with quantitative conversion to the parent phenol, observed in many cases in less than 1 hour.

## Abstract 2

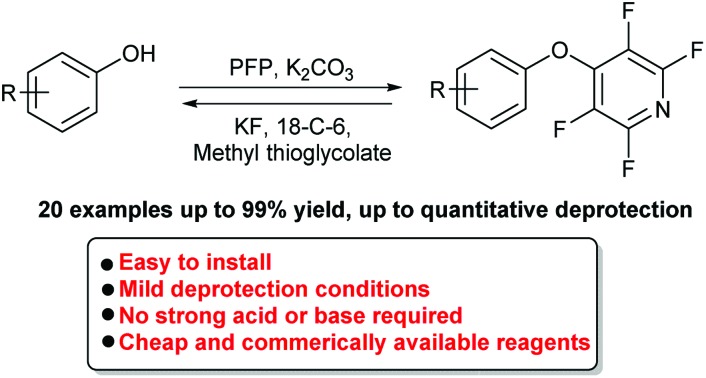
Herein we introduce tetrafluoropyridyl (TFP) as a new general protecting group for phenols. The TFP protecting group is readily cleaved under mild conditions.

## Introduction

**Scheme 1:**
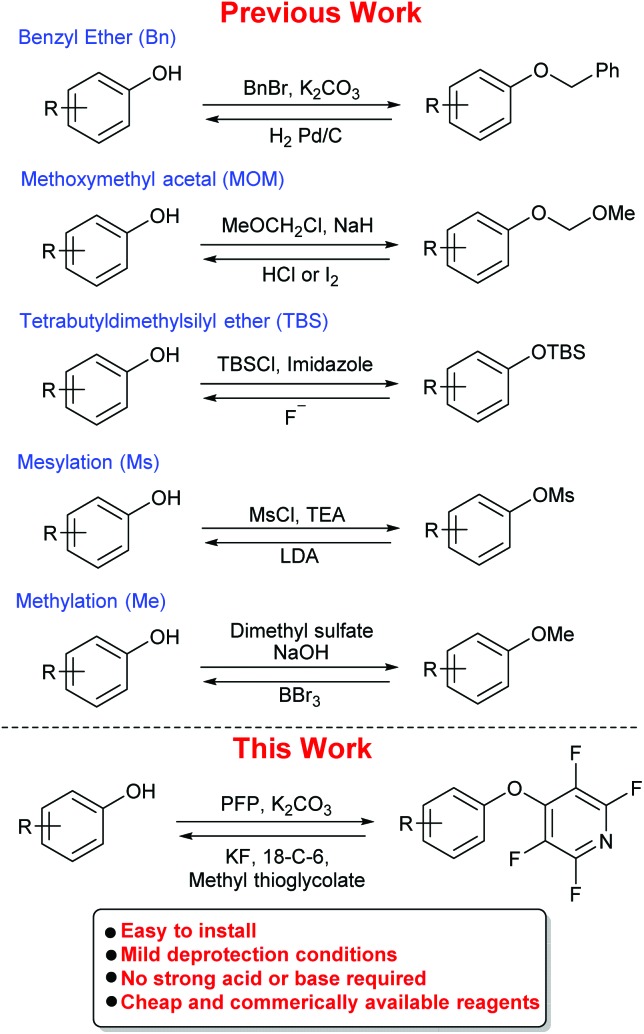
Previous work, currently available protecting groups for phenols including their installation and deprotection conditions. This work, the use of a tetrafluoropyridine moiety as an easily installable and removable phenol protecting group.

Phenols are a ubiquitous aromatic motif present in a wide range of chemical entities. Natural products (*e.g.*, flavonoids, cannabinoids and rotenoids),^1^ pharmaceuticals (*e.g.*, antiseptics and disinfectants),^2^ catalysts^3^ and materials^4^ all contain phenols in their structures. Therefore, the need for effective phenol protecting groups is of importance to synthetic chemists. Common phenol protecting group strategies employed in synthetic organic chemistry include the use of methyl (Me) or benzyl (Bn) ethers and methoxymethyl acetals (MOM), all of which can require harsh conditions to remove (Scheme 1).^5^ Other protecting groups, such as mesylates (Ms) and silyl groups (*e.g.*, TBS), can require low temperatures^6^ and phase-transfer catalysts^7^ to be removed and installation of a methyl ether is often achieved using toxic reagents such as dimethyl sulfate. In addition, many silyl ethers are often found to be unstable in the presence of acids.^8^ In many sub-classes of organic chemistry, phenol protection still presents significant problems. For example, in peptide synthesis the protection of tyrosine is important due to the potential for acylation or alkylation of the phenolic oxygen or the aromatic ring.^9^ Current protecting group strategies for tyrosine can require harsh deprotection conditions (*e.g.*, Bn/Z requires HF or hydrogenation, *t*Bu requires 50% TFA : DCM)^10^ and the limited number which can be removed under milder conditions are often sensitive to acid (*e.g.* Trt).^11^

As highlighted, current phenol protecting groups are often less than ideal in certain circumstances, and thus there is a need to develop new strategies to meet these challenges.

To address this need, we envisaged that one reaction class which could be exploited to develop a general phenol protecting group was S_N_Ar (nucleophilic aromatic substitution).^12^ In the literature, phenols have been shown to react in S_N_Ar reactions with a range of electron deficient aryl halides.^13^ Within these reported reactions, S_N_Ar processes between phenols and perfluoroaromatics/perfluoroheteroaromatics have been observed to be facile and rapid. For example, pentafluoropyridine (PFP) has been observed to undergo rapid S_N_Ar with phenolic compounds.^14^ In addition, our own group has reported the use of PFP as a tagging reagent in peptide chemistry. We observed that PFP could undergo S_N_Ar reactions with peptides containing nucleophilic side-chains, including tyrosine.^15^ We have also developed a range of unnatural amino acids using the same chemistry.^16^

The reaction between PFP and phenols is highly regioselective, with the 4-position of the pyridine ring being the most susceptible to substitution.^17^ Simply mixing a 1 : 1 ratio of phenol and PFP in the presence of base will give the tetrafluoropyridyl (TFP) bi-aryl ether at room temperature with no sensitivity to water or air.^18^

**Scheme 2:**
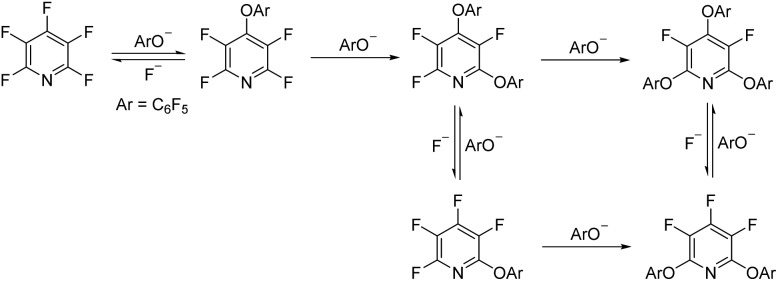
Previous study by Vlasov *et al.*; proposed mechanism for the reaction of pentafluoropyridine (PFP) with pentafluorophenol in the presence of potassium fluoride.

Previous studies by Vlasov *et al.*^19^ and Aksenov *et al.*^20^ have shown that the addition of potassium fluoride (KF) to a reaction between a phenol and PFP leads to a complex mixture of products. They rationalised this observation by proposing that the fluoride ion can cleave the pyridine ether bond, thus regenerating a perfluoroheteroaromatic species which can undergo further S_N_Ar reactions to give a complex mixture of products (Scheme 2).

This led us to postulate that if it were possible to intercept the regenerated PFP after exposure to KF, then the tetrafluoropyridyl (TFP) group could act as a readily-cleavable protecting group for phenols. Herein, we explore this idea in detail and demonstrate that TFP ethers can be installed across a wide range of phenolic compounds and cleaved under mild conditions utilising a combination of fluoride salts and methyl thioglycolate.

## Results and discussion

**Scheme 3:**
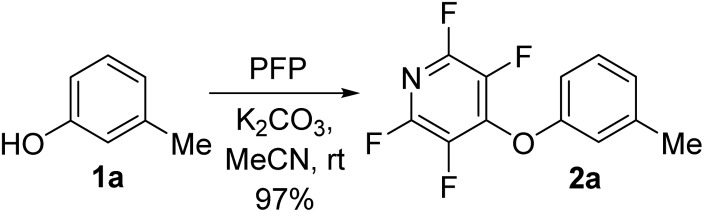
Installation of the tetrafluoropyridyl (TFP) moiety through an S_N_Ar reaction.

To begin our studies, we wished to develop conditions for cleavage of a TFP ether using the model compound **2a**. Exposing *m*-cresol to PFP in the presence of potassium carbonate in acetonitrile led to clean installation of the TFP ether and compound **2a** in 97% yield (Scheme 3). We utilised ^1^H NMR spectroscopy to develop cleavage conditions due to its ease of use and utility in high-throughput applications. Exposing the model compound **2a** to the potassium halides KI, KBr and KCl in a mixture of acetonitrile-*d*_3_ and D_2_O (D_2_O was required to solubilise the inorganic salts) led to no reaction (Table 1, entries 1–3). A trace amount of the desired phenol **1a** was observed with KF (Table 1, entry 4). Addition of a crown ether did not improve the conversion of the reaction (Table 1, entries 5 and 6). Switching to NaI also did not furnish the desired species (Table 1, entry 7). Finally, upon the addition of a thiol, clean and rapid deprotection was observed, and conversion to **1a** was observed to be 78% after 1 h at 50 °C (Table 1, entry 8). In the absence of the crown ether the reaction still proceeded, albeit with a decreased rate, with 61% conversion observed after 1 h (Table 1, entry 9). Decreasing the number of equivalents of reagents also led to clean deprotection but at a decreased rate (Table 1, entry 10). This result demonstrated that if rapid deprotection is not required, significantly fewer equivalents of reagents can be employed during deprotection.

**Table 1:** Deprotection condition screening

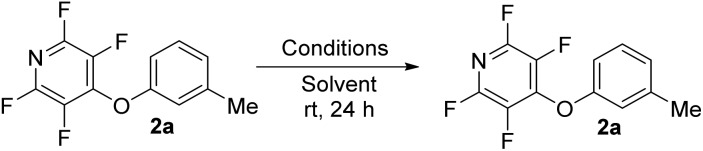			
Entry	Conditions	Time/h	Conversion^*a*^/%
1	KI (2 equiv.)	24	—
2	KBr (2 equiv.)	24	—
3	KCl (2 equiv.)	24	—
4	KF (2 equiv.)	24	<1
5	KI (2 equiv.), 18-C-6 (3 equiv.)	24	<1
6	KF (2 equiv.), 18-C-6 (3 equiv.)	24	1
7	NaI (2 equiv.), 18-C-6 (3 equiv.)	24	<1
8	KF (2 equiv.), 18-C-6 (3 equiv.), methyl thioglycolate (10 equiv.)	1	78
9	KF (2 equiv.), methyl thioglycolate (10 equiv.)	1	61
10	KF (1 equiv.), 18-C-6 (1 equiv.), methyl thioglycolate (2 equiv.)	1	57
11	Methyl thioglycolate (10 equiv.)	24	<1
12	Methyl thioglycolate (10 equiv.). K_2_CO_3_ (2 equiv.)	24	90

^*a*^Conversion was determined by ^1^H NMR analysis of the reaction mixture (see ESI). All reactions were carried out in a water bath set to 50 °C.

We also tested the thiol on its own, but only a trace conversion to the phenol **1a** was observed after 24 h (Table 1, entry 11). Finally, we decided to try adding base and thiol to the TFP ether; this also led to deprotection with the reaction reaching 90% conversion after 24 h (Table 1, entry 12). Due to the rapid nature of deprotection, we decided to continue the study using the conditions from Table 1, entry 8, but it should be noted that deprotection will occur (but at a slower rate) with fewer equivalents of KF, 18-C-6 and methyl thioglycolate or by the application of methyl thioglycolate in the presence of potassium carbonate.

With the selected deprotection conditions in hand, we then progressed to study the stability of the bi-aryl tetrafluoropyridine moiety. **2a** was dissolved in chloroform-*d* (non-polar), methanol-*d*_4_ (polar, protic) or acetonitrile-*d*_3_ (polar, aprotic) and exposed to a range of common synthetic reagents at room temperature for 24 h (see ESI† for details). Under all of the conditions tested, compound **2a** was seen to be stable in chloroform-*d* (Table 2, entries 1–11). Notably, both acid and base were tolerated (Table 2, entries 1 and 5). In methanol-*d*_4_ the TFP ether was seen to be unstable in the presence of base (Table 2, entries 3, 4 and 12). It has previously been reported that perfluoroaromatic ethers are unstable in the presence of methoxide and thus it was not surprising that the TFP-ether was also unstable under such conditions.^21^ In acetonitrile-*d*_3_ the TFP ether was unstable in the presence of strong base (Table 2, entries 4 and 5). Across all the other conditions tested, the TFP ether was observed to be stable.

**Table 2:** TFP stability screening

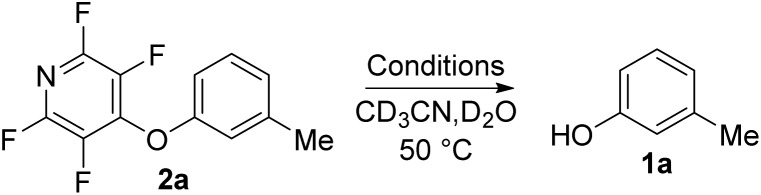				
Entry	Conditions^*a*^	Stability (CDCl_3_)	Stability (MeOD)	Stability (CD_3_CN)
1	0.1 mL TFA	Stable	Stable	Stable
2	0.1 mL 12 M HCl	Stable	Stable	—
3	20 mg K_2_CO_3_	Stable	Unstable	Stable
4	20 mg ^*t*^BuOK	Stable	Unstable	Unstable
5	20 mg NaH	Stable	—	Unstable
6	0.1 mL TEA^*b*^	Stable	Stable	Stable
7	0.1 mL DIPEA	Stable	Stable	Stable
8	0.1 mL SOCl_2_	Stable	Stable	Stable
9	20 mg NaBH_4_	Stable	Stable	Stable
10	20 mg I_2_	Stable	Stable	Stable
11	20 mg DCC	Stable	Stable	Stable
12	20 mg NaOH	—	Unstable	—

^*a*^All reactions were carried out in 0.7 mL of solvent with 10 mg of the tetrafluoropyridyl ether **2a**.

^*b*^ ^1^H NMR resonances were seen to shift in the presence of TEA. Upon concentration under reduced pressure and resuspension in chloroform-*d*, the resonances were observed to shift back to their original positions.

**Scheme 4:**
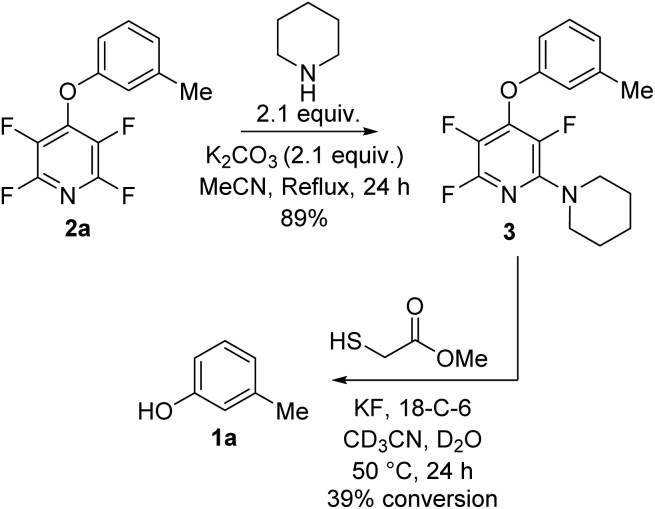
Exposure of TFP aryl ether **2a** to nucleophiles under forcing conditions followed by deprotection to the parent phenol.

Next we tested the TFP protecting group in the presence of other nucleophiles in a basic environment. It has been previously reported^17^ that PFP can react sequentially with multiple nucleophiles and we wished to confirm that the fluoropyridine group could still be cleaved even if additional fluorine substitutions did occur. Exposing **2a** to forcing reaction conditions of 2.1 equivalents of piperidine, and 2.1 equivalents of potassium carbonate in refluxing acetonitrile for 24 h resulted in recovery of compound **3** (Scheme 4).

Exposure of **3** to the developed deprotection conditions showed conversion to the free phenol, but the rate of conversion was slower than that for **2a** (Scheme 4). This reaction demonstrated that it was still possible to cleave the fluoropyridine group even if it has undergone unwanted S_N_Ar reactions.

**Fig. 1:**
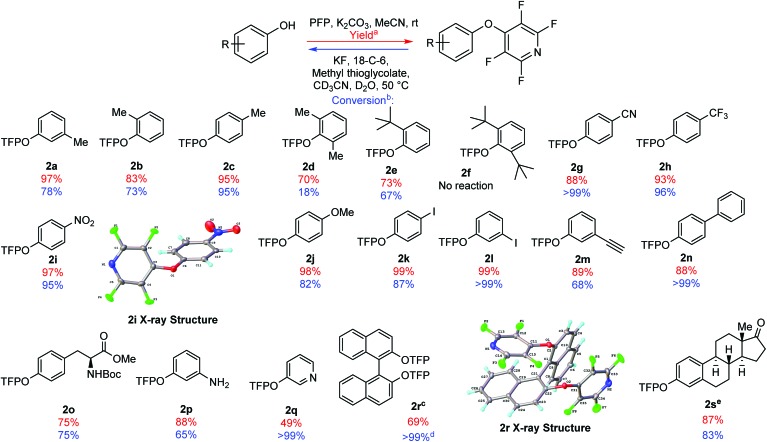
Scope of installation (yields shown in red) and subsequent cleavage of TFP aryl ethers (conversion to the phenol shown in blue). (a) Isolated yield following purification. (b) Conversion to the phenol following 1 h under deprotection conditions, measured by integration of ^1^H NMR spectra (see ESI†). (c) TFP ether formation carried out in DMF due to solubility. (d) Reaction time = 96 h. (e) TFP ether formation carried out in a mixture of MeCN and DMF.

In order to probe the utility of the protecting group, we synthesised a range of TFP bi-aryl ethers in good to excellent yields (70–99%) (Fig. 1, **2a–2n**). The only substrate tested which was not compatible with TFP-ether formation was di-*tert*-butyl **2f** presumably due to its steric bulk. All of the TFP bi-aryl ethers prepared were susceptible to the selected deprotection conditions with complete quantitative conversion to the phenol, observed in some cases in less than 1 h (**2g**, **2l**, **2n** see ESI, pages S-107/S-112/S-114†).

It should also be noted that di-*ortho*-substituted TFP bi-aryl ethers took longer to deprotect than mono substituted varients. For example, di-methyl **2d** had only reached 18% conversion to its parent phenol after 1 h. Presumably, this is due to the increased steric bulk surrounding the aryl-ether bond.

We were able to demonstrate the flexibility of the TFP group in protecting a range of phenols (Fig. 1). The TFP moiety was also installed and cleanly removed from hydroxypyridine, showing quantitative conversion after 1 h (Fig. 1, **2q**). Tyrosine was another phenolic species we wished to test, due to its utility in peptide chemistry, and as part of our ongoing research program into the synthesis of new amino acids.^22^ The TFP group was installed to give ether **2o** in 75% yield, and after 1 h under deprotection conditions the reaction had reached 75% conversion.

Modification of 1,1′-bi-2-naphthol (BINOL) through S_N_Ar with PFP has been previously disclosed by Koltunov *et al.*^23^ BINOL and substituted BINOLs have been utilised extensively in asymmetric catalysis.^24^ Exposing BINOL to 2 equivalents of PFP in the presence of K_2_CO_3_ gave the bis-TFP aryl ether **2r** in 69% yield. This product was susceptible to the cleavage conditions, but the rate of deprotection was observed to be slow, with 96 h required to reach quantitative conversion back to BINOL.

To demonstrate the TFP group's applicability to steroid chemistry, we synthesised the TFP protected estrone derivative **2s** in 87% yield. Exposure of the steroid derivative **2s** to the deprotection conditions cleanly removed the TFP ether, with 83% conversion to estrone observed after 1 h. It should be noted that other perfluoroaromatics have been previously employed as protecting groups in steroid synthesis, but these required the use of sodium methoxide in DMF to recover the parent steroids.^21^

To confirm that the phenol was being successfully liberaterated using our developed conditions NMR spiking experiments were undertaken on a selection of the synthesised substrates. In all cases, spiking the reaction sample with reference compounds clearly demonstrated that the phenols were being successfully generated (see ESI pages S-123 to S-129†).

We also probed the fate of the methyl thioglycolate using mass spectrometry. Analysing the deprotection reaction mixture of **2a** with LCMS, we observed a species with a mass equating to a pentafluoropyridine (PFP) with 2 positions substituted by methyl thioglycolate (see ESI†). This supports our hypothesis in terms of a mechanism for the deprotection in that after the fluoride regenerates the free PFP it is intercepted *in situ* by the thiol (*e.g.* 2× methyl thioglycolate) to form a species which is unable to undergo an S_N_Ar reaction with the released phenol.

To further validate the developed conditions we undertook the deprotection of **2i** using non-deuterated solvents on a larger scale. After 2 h, we were able to successfully isolate 4-nitrophenol from the reaction mixture in 98% isolated yield (see ESI page S-15†).

During the synthesis of the TFP aryl ethers (Fig. 1), we observed that may of the examples were highly stable crystalline solids. Therefore, we were able to obtain crystal structures for compounds **2r** and **2i** (Fig. 1) which confirmed the regiochemistry of the products.^25^

**Scheme 5:**
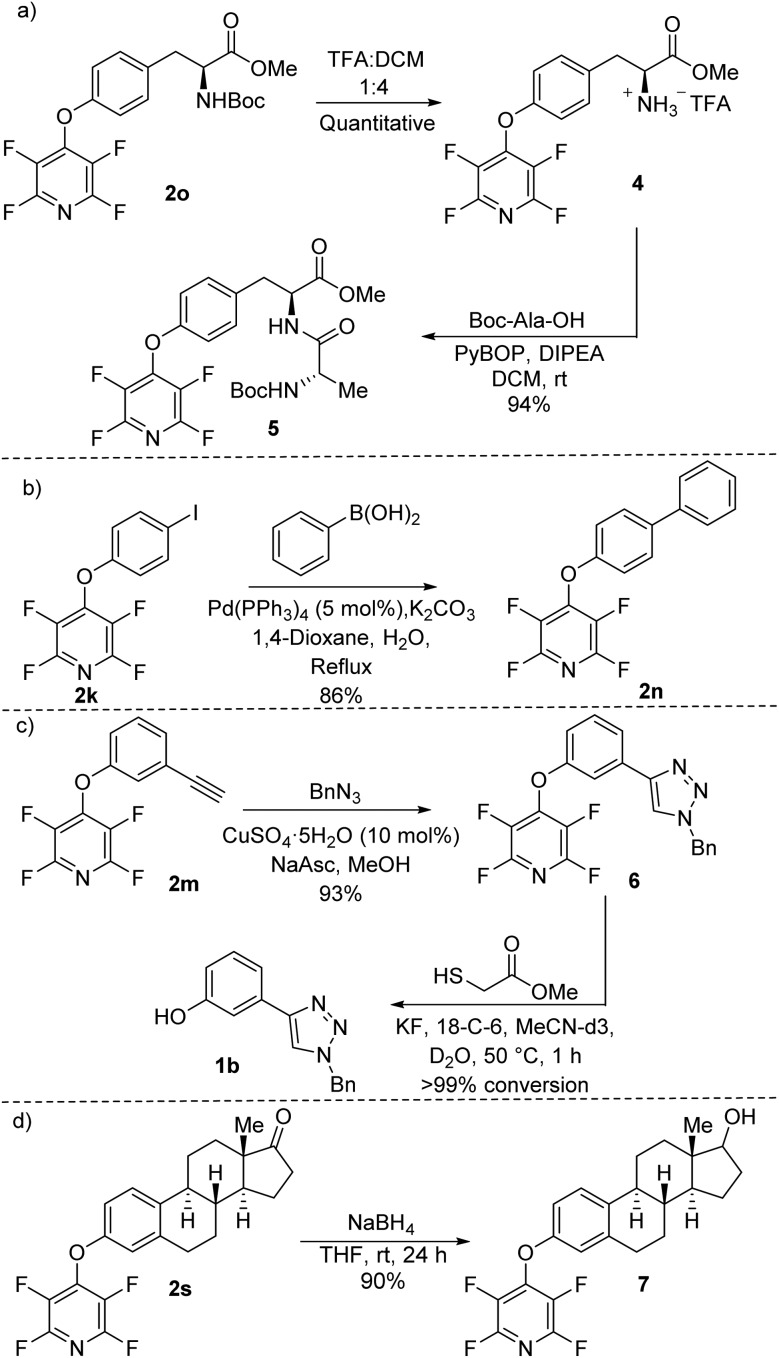
(a) Boc-deprotection of a TFP ether followed by amide formation to garner a dipeptide. (b) Suzuki–Miyaura cross-coupling of an iodo-TFP aryl ether. (c) CuAAC reaction of a TFP ether. (d) Reduction of estrone TFP ether **2s**.

Finally, to further demonstrate the synthetic scope of the methodology developed we carried out a range of chemical transformations on our synthesised TFP ethers (Scheme 5). The free amine **4** was prepared by exposing the tyrosine TFP ether **2o** to standard Boc-deprotection conditions. **4** was allowed to react with Boc-Ala-OH using common amide bond-forming reagents (*e.g.* PyBOP) to afford the dipeptide **5** in 94% yield (Scheme 5a). No cleavage of the TFP moiety was observed during the process.

Using Suzuki–Miyaura cross-coupling conditions, the iodo-TFP ether **2k** reacted effectively with phenylboronic acid to give compound **2n** in 86% yield (Scheme 5b). **2n** had previously been found to be readily susceptible to the developed deprotection conditions (Fig. 1). We found that the alkyne-appended TFP ether **2m** could undergo copper-catalysed azide–alkyne cycloaddition (CuAAC) (click chemistry) to give triazole **6** in excellent 93% yield (Scheme 5c).

Exposure of **6** to the developed deprotection conditions afforded the phenolic triazole **1b** with quantitative conversion observed by ^1^H NMR spectroscopy (see ESI page S-120†). We carried out a borohydride-mediated reduction of the TFP-estrone derivative **2s** (Scheme 5d). The reaction between **2s** and sodium borohydride proceeded smoothly to give the secondary alcohol **7** in 90% yield. We deliberately allowed the reaction to stir for an extended period of 24 h to make sure that the TFP group was stable; no cleavage of the ether was observed under the reaction conditions.

## Conclusions

In conclusion, we have demonstrated that TFP ethers can be utilised as general protecting groups for phenolic oxygens. The TFP ether moiety was installed in up to 99% yield across a diverse range of substrates. TFP ethers were shown to be stable to a range of synthetic reaction conditions, and there is no need to take additional precautions to exclude water or air during their synthesis or storage. In addition, in many cases the TFP ethers were highly crystalline solids, and this offers the opportunity to characterise intermediates in long synthetic sequences using X-ray crystallography. The TFP group also conveniently incorporates a ^19^F NMR handle, which allows for additional spectroscopic possibilities during structural analysis or reaction monitoring. A mixture of KF, 18-C-6 and methyl thioglycolate led to rapid and clean removal of the TFP group to liberate the parent phenols, and in some cases deprotection occurred with quantitative conversion in 1 h at 50 °C. TFP ethers were demonstrated to be compatible with the reaction conditions required to carry out amide bond formation, palladium cross-coupling, carbonyl reduction and click chemistry (CuAAC). The ease of installation and deprotection of the TFP group in combination with its chemical stability and physical properties make it an attractive option for phenol protection.

## Experimental

### General procedure for the synthesis of tetrafluoropyridyl ethers

To a stirred solution of phenol (1 equiv.) in acetonitrile (20 mL) was added pentafluoropyridine (1.05 equiv.) and potassium carbonate (1.05 equiv.). The reaction mixture was stirred at room temperature for 16 h. After this time the reaction mixture was concentrated under reduced pressure and the resulting residue was purified by flash column chromatography.

### General procedure for the deprotection of tetrafluoropyridyl ethers

To a stirred solution of TFP ether (1 equiv.) in acetonitrile (5 mL) and water (0.1 mL) was added potassium fluoride (2 equiv.), 18-crown-6 (3 equiv.) and methyl thioglycolate (10 equiv.). The reaction mixture was stirred for 2 h at 50 °C. After this time the reaction mixture was concentrated under reduced pressure and the resulting residue purified by flash column chromatography.

## Conflicts of interest

There are no conflicts to declare.

## Supplementary Material

Supplementary information

Crystal structure data
