# Supplementary material for: Tetrafluoropyridyl (TFP): a general phenol protecting group readily cleaved under mild conditions
Source: Org Biomol Chem. 2019 Jan 9;17(8):2110–5. doi: 10.1039/c8ob02899k (PMC6390695; doi:10.1039/c8ob02899k)
Supplement: Supplementary file 1 [file OB-017-C8OB02899K-s001.pdf]

Electronic Supplementary Material (ESI) for Organic & Biomolecular Chemistry.
This journal is © The Royal Society of Chemistry 2018

Supporting Information

Tetrafluoropyridyl (TFP): A General Phenol Protecting Group Readily Cleaved Under Mild Conditions

William D. G. Brittain*[a] and Steven L. Cobb*[a]

[a]*Department of Chemistry, Durham University, South Road, Durham DH1 3LE, United Kingdom*

Email: william.d.brittain@durham.ac.uk, s.l.cobb@durham.ac.uk

Contents

General 2
- General procedure for the synthesis of tetrafluoropyridyl ethers 3
- General procedure for the deprotection of tetrafluoropyridyl ethers 3
- Synthesis of 2,3,5,6-tetrafluoro-4-(m-tolyloxy)pyridine 2a 3
- Synthesis of 2,3,5,6-tetrafluoro-4-(o-tolyloxy)pyridine 2b 4
- Synthesis of 2,3,5,6-tetrafluoro-4-(p-tolyloxy)pyridine 2c 4
- Synthesis of 4-(2,6-dimethylphenoxy)-2,3,5,6-tetrafluoropyridine 2d 5
- Synthesis of 4-(2-(tert-butyl)phenoxy)-2,3,5,6-tetrafluoropyridine 2e 6
- Synthesis of 4-((perfluoropyridin-4-yl)oxy)benzonitrile 2g 6
- Synthesis of 2,3,5,6-tetrafluoro-4-(4-(trifluoromethyl)phenoxy)pyridine 2h 7
- Synthesis of 2,3,5,6-tetrafluoro-4-(4-nitrophenoxy)pyridine 2i 7
- Synthesis of 2,3,5,6-tetrafluoro-4-(4-methoxyphenoxy)pyridine 2j 8
- Synthesis of 2,3,5,6-tetrafluoro-4-(4-iodophenoxy)pyridine 2k 9
- Synthesis of 2,3,5,6-tetrafluoro-4-(3-iodophenoxy)pyridine 2l 9
- Synthesis of 4-(3-ethynylphenoxy)-2,3,5,6-tetrafluoropyridine 2m 10
- Synthesis of 4-([1,1'-biphenyl]-4-yloxy)-2,3,5,6-tetrafluoropyridine 2n 11
- Synthesis of methyl (*S*)-2-((tert-butoxycarbonyl)amino)-3-(4-((perfluoropyridin-4-yl)oxy)phenyl)propanoate 2o 12
- Synthesis of 3-[(2,3,5,6-tetrafluoropyridin-4-yl)oxy]aniline 2p 12
- Synthesis of 2,3,5,6-tetrafluoro-4-(pyridin-3-yloxy)pyridine 2q 13
- Synthesis of 2,2'-bis((perfluoropyridin-4-yl)oxy)-1,1'-binaphthalene 2r 14
- Synthesis of (1S,10R,11S,15S)-15-methyl-5-[(2,3,5,6-tetrafluoropyridin-4-yl)oxy]tetracyclo[8.7.0.$0^{2,7}$.$0^{11,15}$]heptadeca-2(7),3,5-trien-14-one 2s 15
- Deprotection of 2i to 4-nitrophenol 16

Synthesis of 2,3,5-trifluoro-4-(3-methylphenoxy)-6-(piperidin-1-yl)pyridine 3 16
Synthesis of methyl (2*S*)-2-amino-3-{4-[(2,3,5,6-tetrafluoropyridin-4-yl)oxy]phenyl}propanoate 4 17
Synthesis of methyl (S)-2-((S)-2-((tert-butoxycarbonyl)amino)propanamido)-3-(4-((perfluoropyridin-4-yl)oxy)phenyl)propanoate 5 18
Suzuki-Miyaura Cross Coupling to form 4-([1,1'-biphenyl]-4-yloxy)-2,3,5,6-tetrafluoropyridine 2n 19
Synthesis of 4-[3-(1-benzyl-1*H*-1,2,3-triazol-4-yl)phenoxy]-2,3,5,6-tetrafluoropyridine 6 19
Synthesis of 3-(1-benzyl-1*H*-1,2,3-triazol-4-yl)phenol 1b 20
Synthesis of (1S,10R,11S,15S)-15-methyl-5-[(2,3,5,6-tetrafluoropyridin-4-yl)oxy]tetracyclo[8.7.0.0^{2,7}.0^{11,15}]heptadeca-2(7),3,5-trien-14-ol 7 21
NMR Data for Synthesised Compounds 23
Deprotection Reactions Monitored by NMR 98
General Procedure for TFP Ether Cleavage monitored by ^1H NMR 98
General Procedure for stability testing using ^1H NMR 101
NMR Spiking Experiments 125
Mass Spectrometry 132
X-ray Crystallography 132
References 134

General

All starting materials and reagents were bought from commercial sources and used as received. All reference phenols were purchased from Sigma Aldrich and their NMR spectra recorded as received. All reactions apart from where noted were carried out in air using non-dried solvents or reagents. All flash column chromatography was carried out using silica purchased from Sigma Aldrich using the solvent system noted. ^1H NMR spectra were recorded at 400 and 700 MHz using Bruker Avance III and Varian VNMRS-700 spectrometers. ^{13}C NMR spectra were recorded at 101, 151 and 176 MHz using Bruker Avance III, Varian VNMRS-600 and Varian VNMRS-700 spectrometers. ^{19}F NMR spectra were recorded at 376 MHz using a Bruker Avance III spectrometer. All coupling constants are reported in Hertz (Hz). In cases where it was required 2D NMR techniques were used to confirm compound identity. Chemical shifts are reported in ppm and are referenced to residual solvent peaks; $CHCl_3$ (^1H 7.26 ppm, ^{13}C 77.0 ppm) and CH_3CN (^1H 1.96 ppm, ^{13}C 118.3). Mass spectra were

collected on a Waters TQD mass spectrometer and accurate mass spectra were collected on a Waters LCT Premier XE mass spectrometer. Optical rotations were measured with a Jasco P-1020 polarimeter. Melting points were carried out in triplicate and an average of the values taken and reported as a range using a Stuart SMP10 melting point apparatus. Melting points were carried out directly on material purified by flash column chromatography

General procedure for the synthesis of tetrafluoropyridyl ethers

To a stirred solution of phenol (1 equiv.) in acetonitrile (20 mL) was added pentafluoropyridine (1.05 equiv.) and potassium carbonate (1.05 equiv.). The reaction mixture was stirred at room temperature for 16 h. After this time the reaction mixture was concentrated under reduced pressure and the resulting residue was purified directly by flash column chromatography.

General procedure for the deprotection of tetrafluoropyridyl ethers

To a stirred solution of TFP ether (1 equiv.) in acetonitrile (5 mL) and water (0.1 mL) was added potassium fluoride (2 equiv.), 18-Crown-6 (3 equiv.) and methyl thioglycolate (10 equiv.). The reaction mixture was stirred for 2 h at 50 °C. After this time the reaction mixture was concentrated under reduced pressure and the resulting residue purified directly by flash column chromatography.

Synthesis of 2,3,5,6-tetrafluoro-4-(m-tolyloxy)pyridine 2a

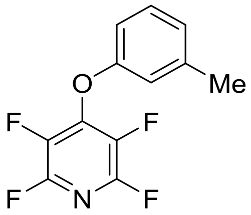

The title compound was synthesised according to the general procedure for the synthesis of tetrafluoropyridyl ethers from 4.63 mmol of the corresponding phenol as a white solid (1.16 g) in 97% yield.

^{1}H NMR (400 MHz, Chloroform-*d*) δ 7.32 – 7.25 (m, 1H, Ar*H*), 7.05 (dt, *J* = 7.6, 0.8, 1H, Ar*H*), 6.91 – 6.85 (m, 2H, Ar*H*), 2.39 (s, 3H, CH_3).

^{13}C NMR (176 MHz, Chloroform-*d*) δ 155.80, 145.86 – 144.10 (m), 144.26 – 142.26 (m), 140.46, 138.27 – 136.67 (m), 136.27 – 133.55 (m), 129.66, 125.89, 117.20, 113.53, 21.33.

^{19}F NMR (376 MHz, Chloroform-*d*) δ -88.63 – -89.17 (m), -152.14 – -156.08 (m).

HRMS ESI$^-$ Calculated for [M-H]$^-$ $C_{12}H_6NOF_4^-$ = 256.0386 Found = 256.0383

MP 39-40 °C

Synthesis of 2,3,5,6-tetrafluoro-4-(o-tolyloxy)pyridine 2b

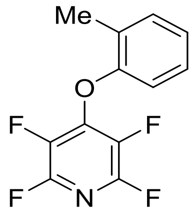

The title compound was synthesised according to the general procedure for the synthesis of tetrafluoropyridyl ethers from 4.63 mmol of the corresponding phenol as a clear oil (0.980 g) in 83% yield.

^1H NMR (400 MHz, Chloroform-*d*) δ 7.35 – 7.28 (m, 1H, Ar*H*), 7.25 – 7.11 (m, 2H, Ar*H*), 6.84 (d, *J* = 7.8 Hz, 1H, Ar*H*), 2.40 (s, 3H, C*H*$_3$).

^{13}C NMR (176 MHz, Chloroform-*d*) δ 154.29, 145.15 – 144.98 (m), 144.96 – 144.71 (m), 143.69 – 143.17 (m), 136.73 – 136.39 (m), 135.30 – 134.94 (m), 131.73, 127.99, 127.19, 125.28, 115.75, 15.82.

^{19}F NMR (376 MHz, Chloroform-*d*) δ -88.13 – -90.17 (m), -154.52 – -156.92 (m).

HRMS ESI$^-$ Calculated for [M-H]$^-$ $C_{12}H_6NOF_4^-$ = 256.0386 Found = 256.0389

Synthesis of 2,3,5,6-tetrafluoro-4-(p-tolyloxy)pyridine 2c

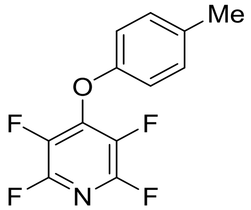

The title compound was synthesised according to the general procedure for the synthesis of tetrafluoropyridyl ethers from 4.63 mmol of the corresponding phenol as a clear oil (1.13 g) in 95% yield.

^{1}H NMR (400 MHz, Chloroform-*d*) δ 7.25 – 7.11 (m, 2H, Ar*H*), 6.98 (app d, *J* = 8.6, 2H, Ar*H*), 2.38 (s, 3H, C*H*$_3$).

^{13}C NMR (176 MHz, Chloroform-*d*) δ 153.78, 145.04 – 144.57 (m), 143.86 – 143.04 (m), 137.16 – 136.45 (m), 135.55 – 135.17 (m), 134.90, 130.38, 116.56, 20.66.

^{19}F NMR (376 MHz, Chloroform-*d*) δ -88.46 – -89.71 (m), -154.17 – -155.60 (m).

HRMS ESI$^-$ Calculated for [M-H]$^-$ $C_{12}H_6NOF_4^-$ = 256.0386 Found = 256.0376

Synthesis of 4-(2,6-dimethylphenoxy)-2,3,5,6-tetrafluoropyridine 2d

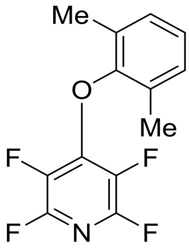

The title compound was synthesised according to the general procedure for the synthesis of tetrafluoropyridyl ethers from 4.09 mmol of the corresponding phenol as a clear oil (0.722 g) in

70% yield.

^{1}H NMR (400 MHz, Chloroform-*d*) δ 7.15 – 7.07 (m, 3H, Ar*H*), 2.25 (s, 6H, C*H*$_3$).

^{13}C NMR (176 MHz, Chloroform-*d*) δ 152.29, 145.88 – 145.48 (m), 145.06 – 144.60 (m), 143.82 – 143.31 (m), 135.25 – 134.65 (m), 133.68 – 133.17 (m), 129.19, 129.08, 126.26, 16.03.

^{19}F NMR (376 MHz, Chloroform-*d*) δ -89.33 – -90.11 (m), -159.23 – -160.18 (m).

HRMS ESI$^-$ Calculated for [M-H]$^-$ $C_{13}H_8NOF_4^-$ = 270.0542 Found = 270.0539

Synthesis of 4-(2-(tert-butyl)phenoxy)-2,3,5,6-tetrafluoropyridine 2e

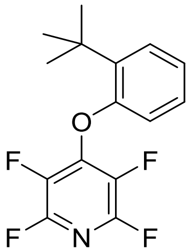

The title compound was synthesised according to the general procedure for the synthesis of tetrafluoropyridyl ethers from 3.33 mmol of the corresponding phenol as a clear oil (0.726 g) in 73% yield.

^{1}H NMR (400 MHz, Chloroform-*d*) δ 7.49 – 7.44 (m, 1H, Ar*H*), 7.23 – 7.16 (m, 2H, Ar*H*), 6.79 – 6.75 (m, 1H, Ar*H*), 1.48 (s, 9H, C*H*$_3$).

^{13}C NMR (176 MHz, Chloroform-*d*) δ 154.80, 145.11 – 144.85 (m), 144.49 – 144.26 (m), 143.75 – 143.43 (m), 139.09, 136.79 – 136.30 (m), 135.52 – 134.92 (m), 127.81, 127.37, 125.07, 116.02, 34.88, 29.91.

^{19}F NMR (376 MHz, Chloroform-*d*) δ -88.20 – -89.85 (m), -154.13 – -155.13 (m).

HRMS ESI$^-$ Calculated for [M-H]$^-$ $C_{15}H_{12}NOF_4^-$ = 298.0855 Found = 298.0857

Synthesis of 4-((perfluoropyridin-4-yl)oxy)benzonitrile 2g

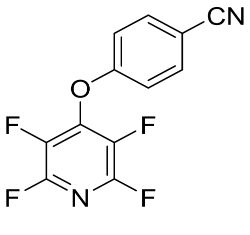

The title compound was synthesised according to the general procedure for the synthesis of tetrafluoropyridyl ethers from 4.20 mmol of the corresponding phenol as a clear oil (0.991 g) in 88% yield.

^{1}H NMR (400 MHz, Chloroform-*d*) δ 7.77 – 7.71 (m, 2H, Ar*H*), 7.23 – 7.14 (m, 2H, Ar*H*).

^{13}C NMR (176 MHz, Chloroform-*d*) δ 158.30, 145.14 – 144.52 (m), 143.69 – 143.25 (m), 142.84 – 142.53 (m), 137.15 – 136.55 (m), 135.53 – 135.18 (m), 134.49, 117.76, 117.14, 109.07.

^{19}F NMR (376 MHz, Chloroform-*d*) δ -86.57 – -87.49 (m), -152.55 – -154.31 (m).

HRMS ESI$^-$ Calculated for $[M\text{-}H]^-$ $C_{12}H_3N_2OF_4^-$ = 267.0182 Found = 267.0179

Synthesis of 2,3,5,6-tetrafluoro-4-(4-(trifluoromethyl)phenoxy)pyridine 2h

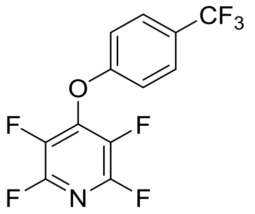

The title compound was synthesised according to the general procedure for the synthesis of tetrafluoropyridyl ethers from 3.08 mmol of the corresponding phenol as a clear oil (0.890 g) in 93% yield.

^{1}H NMR (400 MHz, Chloroform-*d*) δ 7.70 (d, *J* = 8.4, 2H, Ar*H*), 7.18 (d, *J* = 8.4, 2H, Ar*H*).

^{13}C NMR (176 MHz, Chloroform-*d*) δ 157.77, 145.07 – 144.67 (m), 143.66 – 143.11 (m), 137.17 – 136.54 (m), 135.73 – 135.15 (m), 128.28 – 127.09 (m), 123.61 (q, *J* = 271.9), 116.64.

^{19}F NMR (376 MHz, Chloroform-*d*) δ -62.21 (s), -87.19 – -88.83 (m), -153.38 – -154.82 (m).

HRMS ESI$^-$ Calculated for $[M\text{-}H]^-$ $C_{12}H_3NOF_7^-$ = 310.0103 Found = 310.0115

Synthesis of 2,3,5,6-tetrafluoro-4-(4-nitrophenoxy)pyridine 2i

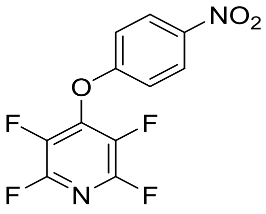

The title compound was synthesised according to the general procedure for the synthesis of tetrafluoropyridyl ethers from 3.60 mmol of the corresponding phenol as a white solid (1.01 g) in 97% yield.

^{1}H NMR (400 MHz, Chloroform-*d*) δ 8.33 (d, *J* = 9.2, 2H, Ar*H*), 7.20 (d, *J* = 9.2, 2H, Ar*H*).

^{13}C NMR (176 MHz, Chloroform-*d*) δ 159.59, 145.01 – 144.75 (m), 144.66, 143.63 – 143.35 (m), 142.76 – 142.51 (m), 137.08 – 136.64 (m), 135.54 – 135.19 (m), 126.19, 116.69.

^{19}F NMR (376 MHz, Chloroform-*d*) δ -85.75 – -87.34 (m), -152.80 – -154.91 (m).

HRMS ESI$^-$ Calculated for $[M-H]^-$ $C_{11}H_4N_2O_3F_4^-$ = 287.0080 Found = 287.0091

MP 84-85 °C

Synthesis of 2,3,5,6-tetrafluoro-4-(4-methoxyphenoxy)pyridine 2j

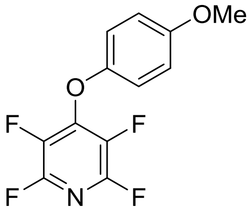

The title compound was synthesised according to the general procedure for the synthesis of tetrafluoropyridyl ethers from 4.03 mmol of the corresponding phenol as a white solid (1.08 g) in 98% yield.

^{1}H NMR (400 MHz, Chloroform-*d*) δ 7.11 – 6.98 (m, 2H, Ar*H*), 6.95 – 6.83 (m, 2H, Ar*H*), 3.83 (s, 3H, CH_3).

^{13}C NMR (176 MHz, Chloroform-*d*) δ 156.91, 149.64, 145.29 – 145.11 (m), 144.99 – 144.78 (m), 143.70 – 143.34 (m), 136.78 – 136.43 (m), 135.40 – 134.98 (m), 118.18, 114.85, 55.66.

^{19}F NMR (376 MHz, Chloroform-*d*) δ -88.92 – -89.19 (m), -154.98 – -155.19 (m).

HRMS ESI$^-$ Calculated for [M-H]$^-$ $C_{12}H_6NO_2F_4^-$ = 272.0335 Found = 272.0326

MP 78-79 °C

Synthesis of 2,3,5,6-tetrafluoro-4-(4-iodophenoxy)pyridine 2k

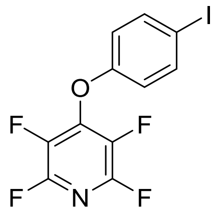

The title compound was synthesised according to the general procedure for the synthesis of tetrafluoropyridyl ethers from 2.27 mmol of the corresponding phenol as a white solid (0.835 g) in 99% yield.

^{1}H NMR (400 MHz, Chloroform-*d*) δ 7.71 (d, *J* = 9.0, 2H, Ar*H*), 6.86 (d, *J* = 9.0, 2H, Ar*H*).

^{13}C NMR (176 MHz, Chloroform-*d*) δ 155.62, 144.98 – 144.70 (m), 143.95 – 143.66 (m), 143.57 – 143.31 (m), 138.99, 136.99 – 136.64 (m), 135.46 – 135.15 (m), 118.74, 88.39.

^{19}F NMR (376 MHz, Chloroform-*d*) δ -87.54 – -88.84 (m), -153.20 – -154.50 (m).

HRMS ESI$^-$ Calculated for [M-H]$^-$ $C_{11}H_3NOF_4I^-$ = 367.9196 Found = 367.9203

MP 86-87 °C

Synthesis of 2,3,5,6-tetrafluoro-4-(3-iodophenoxy)pyridine 2l

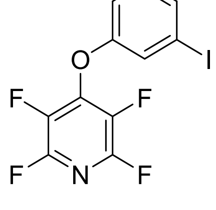

The title compound was synthesised according to the general procedure for the synthesis of tetrafluoropyridyl ethers from 2.27 mmol of the corresponding phenol as a clear oil (0.828 g) in 99% yield.

^{1}H NMR (400 MHz, Chloroform-*d*) δ 7.59 (d, *J* = 7.8, 1H, Ar*H*), 7.44 (t, *J* = 2.1, 1H, Ar*H*), 7.14 (t, *J* = 8.1, 1H, Ar*H*), 7.05 (dd, *J* = 8.1, 2.1, 1H, Ar*H*).

^{13}C NMR (176 MHz, Chloroform-*d*) δ 155.78, 144.96 – 144.69 (m), 143.79 – 143.58 (m), 143.54 – 143.32 (m), 136.96 – 136.70 (m), 135.47 – 135.19 (m), 134.36, 131.27, 125.82, 115.97, 94.14.

^{19}F NMR (376 MHz, Chloroform-*d*) δ -87.45 – -88.23 (m), -153.23 – -154.45 (m).

HRMS ESI^{-} Calculated for [M-H]$^{-}$ $C_{11}H_3NOF_4I^{-}$ = 367.9196 Found = 367.9201

Synthesis of 4-(3-ethynylphenoxy)-2,3,5,6-tetrafluoropyridine 2m

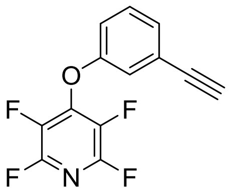

The title compound was synthesised according to the general procedure for the synthesis of tetrafluoropyridyl ethers from 2.11 mmol of the corresponding phenol as a clear oil (0.500 g) in

89% yield.

^{1}H NMR (400 MHz, Chloroform-*d*) δ 7.39 – 7.35 (m, 2H, Ar*H*), 7.20 – 7.16 (m, 1H, Ar*H*), 7.14 – 7.06 (m, 1H, Ar*H*), 3.15 (s, 1H, CC*H*).

^{13}C NMR (151 MHz, Chloroform-*d*) δ 155.40, 145.09 – 144.80 (m), 144.03 – 143.77 (m), 143.47 – 143.19 (m), 137.17 – 136.81 (m), 135.46 – 135.10 (m), 130.02, 128.94, 124.12, 119.95, 117.34, 82.06, 78.68.

^{19}F NMR (376 MHz, Chloroform-*d*) δ -87.94 – -88.26 (m), -153.86 – -154.10 (m).

HRMS ESI$^-$ Calculated for $[M-H]^-$ $C_{13}H_4NOF_4^-$ = 266.0229 Found = 266.0225

Synthesis of 4-([1,1'-biphenyl]-4-yloxy)-2,3,5,6-tetrafluoropyridine 2n

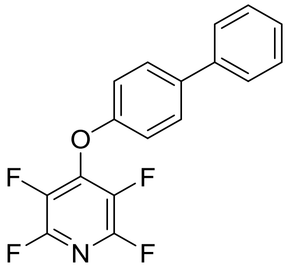

The title compound was synthesised according to the general procedure for the synthesis of tetrafluoropyridyl ethers from 2.94 mmol of the corresponding phenol as a white solid (0.827 g) in 88% yield.

^{1}H NMR (400 MHz, Chloroform-*d*) δ 7.66 – 7.55 (m, 4H, Ar*H*), 7.51 – 7.45 (m, 2H, Ar*H*), 7.42 – 7.36 (m, 1H, Ar*H*), 7.15 (d, *J* = 8.9, 2H, Ar*H*).

^{13}C NMR (176 MHz, Chloroform-*d*) δ 155.22, 145.05 – 144.70 (m), 144.52 – 144.18 (m), 143.62 – 143.31 (m), 139.80, 138.40, 137.14 – 136.80 (m), 135.62 – 135.24 (m), 128.85, 128.65, 127.50, 126.99, 116.92.

^{19}F NMR (376 MHz, Chloroform-*d*) δ -88.36 – -88.61 (m), -154.03 – -154.24 (m).

HRMS ESI$^-$ Calculated for $[M-H]^-$ $C_{17}H_8NOF_4^-$ = 318.0544 Found = 318.0542

MP 130-131 °C

Synthesis of methyl (*S*)-2-((tert-butoxycarbonyl)amino)-3-(4-((perfluoropyridin-4-yl)oxy)phenyl)propanoate 2o

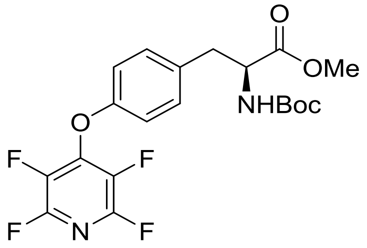

The title compound was synthesised according to the general procedure for the synthesis of tetrafluoropyridyl ethers from 0.68 mmol of the corresponding phenol as a white solid (0.226 g) in 75% yield.

^{1}H NMR (400 MHz, Chloroform-*d*) δ 7.17 (d, *J* = 8.7, 2H, Ar*H*), 7.01 (d, *J* = 8.6, 2H, Ar*H*), 5.03 (br d, *J* = 8.3, 1H, N*H*), 4.64 – 4.57 (m, 1H, C*H*), 3.73 (s, 3H, C*H$_3$*), 3.15 (ABX, *J* = 13.9, 5.8, 1H, C*H$_2$*), 3.05 (ABX, *J* = 13.9, 6.4, 1H, C*H$_2$*), 1.44 (s, 9H, BocC*H$_3$*).

^{13}C NMR (176 MHz, Chloroform-*d*) δ 172.06, 154.93, 154.82, 145.01 – 144.66 (m), 144.50 – 144.27 (m), 143.82 – 143.09 (m), 137.32 – 136.59 (m), 135.68 – 135.13 (m), 133.20, 130.85, 116.72, 80.06, 54.34, 52.26, 37.79, 28.23.

^{19}F NMR (376 MHz, Chloroform-*d*) δ -88.04 – -88.96 (m), -153.31 – -155.42 (m).

HRMS ESI$^-$ Calculated for [M-H]$^-$ $C_{20}H_{19}N_2O_5F_4^-$ = 443.1230 Found = 443.1248

MP 105-106 °C

$[\alpha]_D^{27}$ = + 18.14 (c = 1.0, CH_2Cl_2)

Synthesis of 3-[(2,3,5,6-tetrafluoropyridin-4-yl)oxy]aniline 2p

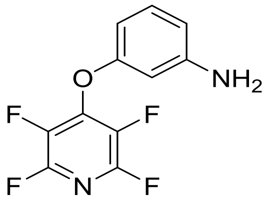

The title compound was synthesised according to the general procedure for the synthesis of tetrafluoropyridyl ethers from 4.59 mmol of the corresponding phenol as a white crystalline solid (1.05 g) in 88% yield.

^{1}H NMR (700 MHz, Chloroform-*d*) δ 7.13 – 7.08 (m, 1H), 6.52 – 6.47 (m, 1H), 6.39 – 6.35 (m, 1H), 3.79 (br s, 2H).

^{13}C NMR (176 MHz, Chloroform-*d*) δ 156.94, 148.21, 144.91 – 144.67 (m), 144.60 – 144.38 (m), 143.54 – 143.26 (m), 137.18 – 136.86 (m), 135.66 – 135.36 (m), 130.56, 111.71, 106.02, 103.22.

^{19}F NMR (376 MHz, Chloroform-*d*) δ -88.74 – -88.99 (m), -154.25 – -154.46 (m).

HRMS ESI$^+$ Calculated for $[M+H]^+$ $C_{11}H_7N_2OF_4^+$ = 259.0495 Found = 259.0496

MP 97-98 °C

Synthesis of 2,3,5,6-tetrafluoro-4-(pyridin-3-yloxy)pyridine 2q

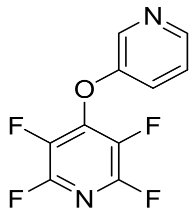

The title compound was synthesised according to the general procedure for the synthesis of tetrafluoropyridyl ethers from 5.26 mmol of the corresponding phenol as a brown oil (0.624 g) in 49% yield.

^{1}H NMR (400 MHz, Chloroform-*d*) δ 8.55 – 8.48 (m, 2H, Ar*H*), 7.44 – 7.34 (m, 2H, Ar*H*).

^{13}C NMR (176 MHz, Chloroform-*d*) δ 152.31, 146.52, 145.00 – 144.68 (m), 143.76 – 143.30 (m), 139.32, 136.90 – 136.50 (m), 135.36 – 134.98 (m), 124.29, 123.90.

^{19}F NMR (376 MHz, Chloroform-*d*) δ -87.05 – -88.31 (m), -153.62 – -154.19 (m).

HRMS ESI$^-$ Calculated for $[M-H]^-$ $C_{10}H_3N_2OF_4^-$ = 243.0182 Found = 243.0183

Synthesis of 2,2'-bis((perfluoropyridin-4-yl)oxy)-1,1'-binaphthalene 2r

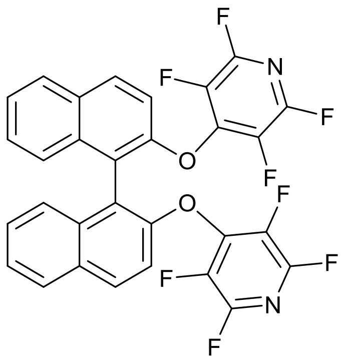

To a solution of 1,1′-Bi-2-naphthol (0.500 g, 1.75 mmol) in DMF (5 mL) was added pentafluoropyridine (0.592 g, 3.50 mmol) and K_2CO_3 (0.506 g, 3.67 mmol). The reaction mixture was stirred at room temperature for 1 h. After this time the reaction mixture was directly poured into water (100 mL). The resulting precipitant was filtered off and dried under vacuum to give the title compound as a white solid (0.702 g) in 69% yield.

^{1}H NMR (400 MHz, Chloroform-*d*) δ 8.02 (d, *J* = 8.9, 2H, Ar*H*), 7.93 (d, *J* = 8.2, 2H, Ar*H*), 7.54 – 7.44 (m, 4H, Ar*H*), 7.41 – 7.34 (m, 2H, Ar*H*), 7.23 (d, *J* = 8.5, 2H, Ar*H*).

^{13}C NMR (176 MHz, Chloroform-*d*) δ 152.19, 144.66 – 144.40 (m), 144.26 – 143.98 (m), 142.89 – 142.49 (m), 136.07 – 135.57 (m), 134.62 – 133.93 (m), 132.86, 131.22, 130.82, 128.25, 127.74, 126.21, 124.95, 119.33, 117.87.

^{19}F NMR (376 MHz, Chloroform-*d*) δ -89.31 – -90.70 (m), -153.43 – -154.82 (m).

HRMS ESI$^-$ Calculated for $[M-H]^-$ $C_{30}H_{11}N_2O2F_8^-$ = 583.0693 Found = 583.0679

MP 210-212 °C

Synthesis of (1S,10R,11S,15S)-15-methyl-5-[(2,3,5,6-tetrafluoropyridin-4-yl)oxy]tetracyclo[8.7.0.0^{2,7}.0^{11,15}]heptadeca-2(7),3,5-trien-14-one 2s

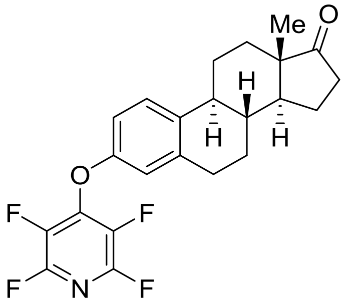

The title compound was synthesised according to the general procedure for the synthesis of tetrafluoropyridyl ethers from 1.85 mmol of estrone with the following modification. Due to the solubility of estrone in MeCN, 1 mL of DMF was added to the reaction mixture and the reaction mixture heated at 70 °C for 2 h. The reaction mixture was concentrated under reduced pressure and the residue purified by flash column chromatography (100% hexane to 90% hexane 10% EtOAc). This gave the title compound as a white crystalline solid (0.676 g) in 87% yield.

^{1}H NMR (400 MHz, Chloroform-*d*) δ 7.30 (d, *J* = 8.6, 1H), 6.86 (dd, *J* = 8.6, 2.7, 1H), 6.80 (d, *J* = 2.7, 1H), 3.06 – 2.86 (m, 2H), 2.61 – 2.49 (m, 1H), 2.46 – 2.39 (m, 1H), 2.35 – 2.26 (m, 1H), 2.24 – 1.97 (m, 4H), 1.73 – 1.42 (m, 7H), 0.95 (s, 3H).

^{13}C NMR (176 MHz, Chloroform-*d*) δ 220.51, 153.85, 144.95 – 144.70 (m), 144.68 – 144.48 (m), 143.56 – 143.33 (m), 138.76, 137.08 – 136.83 (m), 136.73, 135.59 – 135.33 (m), 126.87, 116.52, 113.85, 50.38, 47.89, 44.00, 37.98, 35.80, 31.50, 29.45, 26.24, 25.77, 21.55, 13.81.

^{19}F NMR (376 MHz, Chloroform-*d*) δ -88.67 – -88.96 (m), -154.29 – -154.48 (m).

HRMS ESI$^-$ Calculated for [M-H]$^-$ $C_{23}H_{20}NO_2F_4^-$ = 418.1430 Found = 418.1432

MP 153-154 °C

Deprotection of 2i to 4-nitrophenol

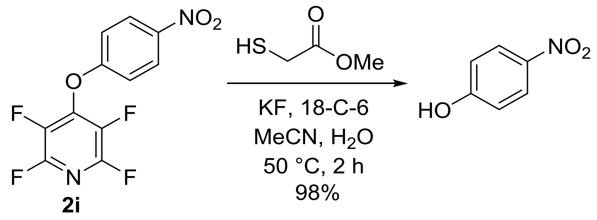

The title compound was synthesised according to the general procedure for the deprotection of tetrafluoropyridyl ethers from 0.22 mmol of compound **1b** as a cream solid (0.030 g) in 98% yield.

Characterisation data were in agreement with reported literature values.[1]

^{1}H NMR (400 MHz, Chloroform-*d*) δ 8.25 – 8.16 (m, 2H, Ar*H*), 6.99 – 6.92 (m, 2H, Ar*H*).

^{13}C NMR (101 MHz, $CDCl_3$) δ 161.28, 141.68, 126.30, 115.71.

MS ESI$^-$ m/z = 138.1 $[M-H]^-$

Synthesis of 2,3,5-trifluoro-4-(3-methylphenoxy)-6-(piperidin-1-yl)pyridine 3

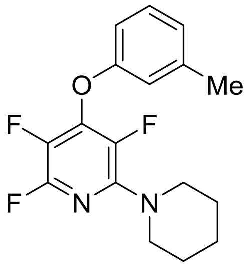

To a solution of 2,3,5,6-tetrafluoro-4-(m-tolyloxy)pyridine (0.100 g, 0.39 mmol) in acetonitrile (10 mL) was added K_2CO_3 (0.113 g, 0.82 mmol) and piperidine (0.070 g, 0.82 mmol). The reaction mixture was heated at reflux for 24 h. After this time the reaction mixture was filtered and concentrated under reduced pressure. The recovered residue was purified by flash column chromatography (100% hexane to 80% hexane 20% EtOAc). This gave the title compound as a clear oil (0.112 g) in 89% yield.

^{1}H NMR (400 MHz, Chloroform-*d*) δ 7.24 (t, *J* = 7.9, 1H, Ar*H*), 6.99 – 6.81 (m, 3H, Ar*H*), 3.47 – 3.36 (m, 4H, C*H*$_2$), 2.37 (s, 3H, C*H*$_3$), 1.67 (s, 6H, C*H*$_2$).

^{19}F NMR (376 MHz, Chloroform-*d*) δ -89.50 (dd, *J* = 27.8, 25.1), -146.38 (dd, *J* = 27.8, 3.1), -165.88 (dd, *J* = 25.1, 3.1).

^{13}C NMR (176 MHz, Chloroform-*d*) δ 156.61, 145.73 (dd, *J* = 12.2, 2.5), 144.42 (dd, *J* = 12.2, 2.5), 143.38 – 143.16 (m), 142.04 – 141.74 (m), 140.07, 139.97 (dd, *J* = 6.8, 2.0), 138.52 (dd, *J* = 6.8, 2.0), 131.93 (d, *J* = 32.7), 130.48 (d, *J* = 32.7), 129.41, 124.65, 116.58, 112.81, 48.56 (d, *J* = 5.8), 25.67, 24.49, 21.35.

HRMS ESI$^+$ Calculated for $[M+H]^+$ $C_{17}H_{18}N_2OF_3^+$ = 323.1371 Found = 323.1385

Synthesis of methyl (2*S*)-2-amino-3-{4-[(2,3,5,6-tetrafluoropyridin-4-yl)oxy]phenyl}propanoate 4

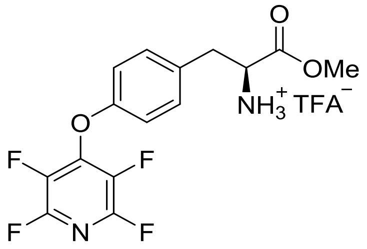

To a solution of methyl (*S*)-2-((tert-butoxycarbonyl)amino)-3-(4-((perfluoropyridin-4-yl)oxy)phenyl)propanoate (0.100 g, 0.26 mmol) in DCM (2 mL) was added TFA (0.5 mL). The reaction mixture was stirred at rt for 2 h. After this time the reaction mixture was concentrated under reduced pressure. This gave the title compound as a colourless crystalline solid (0.112 g) in quantitative yield.

^{1}H NMR (400 MHz, Chloroform-*d*) δ 8.45 (br s, 3H,N*H*), 7.24 (d, *J* = 8.5, 2H, Ar*H*), 7.02 (d, *J* = 8.5 Hz, 2H, Ar*H*), 4.26 (t, *J* = 6.6, 1H, C*H*), 3.71 (s, 3H, CH_3), 3.27 (d, *J* = 6.6, 2H, CH_2).

^{13}C NMR (176 MHz, Chloroform-*d*) δ 169.21, 155.46, 144.95 – 144.66 (m), 144.07 – 143.90 (m), 143.58 – 143.30 (m), 136.99 – 136.63 (m), 135.47 – 135.17 (m), 130.94, 130.16, 117.24, 54.11, 53.19, 35.41.

^{19}F NMR (376 MHz, Chloroform-*d*) δ -76.08, -88.42 – -88.64 (m), -154.37 – -154.56 (m).

HRMS ESI^{+} Calculated for $[M+H]^{+}$ $C_{15}H_{13}N_2O_3F_4^{+}$ = 345.0862 Found = 345.0878

MP 115-116 °C

Synthesis of methyl (S)-2-((S)-2-((tert-butoxycarbonyl)amino)propanamido)-3-(4-((perfluoropyridin-4-yl)oxy)phenyl)propanoate 5

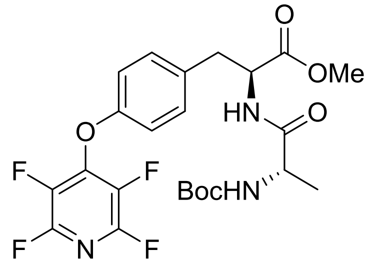

To a solution of **3** (0.025 g, 0.068 mmol) in DCM (10 mL) was added PyBOP (0.038 g, 0.074 mmol), DIPEA (0.010 g, 0.074 mmol) and Boc-Ala-OH (0.011 g, 0.068 mmol) and the reaction mixture stirred for 16 h at rt. After this time the reaction mixture was concentrated under reduced pressure and the residue purified by flash column chromatography (100% hexane to 50% hexane 50% EtOAc). This gave the title compound (0.033 g) as a cream solid in 94% yield.

^{1}H NMR (400 MHz, Chloroform-*d*) δ 7.16 (d, *J* = 8.6, 2H, Ar*H*), 7.00 (d, *J* = 8.6, 2H, Ar*H*), 6.60 (br d, *J* = 7.7, 1H, N*H*), 4.96 – 4.81 (m, 2H, C*H*/N*H*), 4.23 – 4.06 (m, 1H, C*H*), 3.74 (s, 3H, C*H$_3$*), 3.20 (ABX, *J* = 14.0, 5.9, 1H, C*H$_2$*), 3.10 (ABX, *J* = 14.0, 5.9, 1H, C*H$_2$*), 1.46 (s, 9H, C*H$_3$*), 1.34 (d, *J* = 7.1, 3H, C*H$_3$*).

^{13}C NMR (176 MHz, Chloroform-*d*) δ 172.20, 171.43, 154.84, 132.91, 130.86, 116.74, 53.08, 52.39, 50.18, 37.21, 28.23, 18.00. Note: at the concentration of the sample which the spectra was recorded the resonances corresponding to the carbons on the fluoropyridine ring were observed but only weakly therefore these have not been reported.

^{19}F NMR (376 MHz, Chloroform-*d*) δ -88.10 – -88.92 (m), -153.96 – -154.60 (m).

HRMS ESI^{+} Calculated for $[M+H]^{+}$ $C_{23}H_{26}N_3O_6F_4^{+}$ = 516.1752 Found = 517.1758

Suzuki-Miyaura Cross Coupling to form 4-([1,1'-biphenyl]-4-yloxy)-2,3,5,6-tetrafluoropyridine 2n

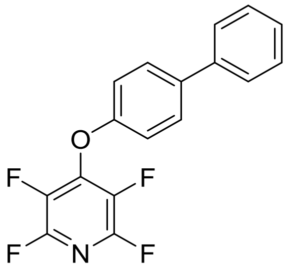

Under an inert atmosphere, a solution of 2,3,5,6-tetrafluoro-4-(4-iodophenoxy)pyridine (0.100 g, 0.27 mmol) and phenyl boronic acid (0.040 g, 0.33 mmol) in 1,4-dioxane (6 mL) was degassed with bubbling nitrogen for 1 h. At the same time a solution of K_2CO_3 (0.112 g, 0.81 mmol) in H_2O (3 mL) was also degassed with bubbling nitrogen for 1 h. After this time $Pd(PPh_3)_4$ (0.016 g, 5 mol%) was added to the dioxane solution and this was further degassed for 20 min. To the dioxane solution was transferred the K_2CO_3 water solution and the resulting mixture heated at reflux for 5 h. After this time the reaction mixture was concentrated under reduced pressure and the resulting residue purified by flash column chromatography (100% hexane to 90% hexane 10% EtOAc). This gave the title compound as a white solid (0.076 g) in 86% yield.

Characterisation data was in agreement with the values reported above for 4-([1,1'-biphenyl]-4-yloxy)-2,3,5,6-tetrafluoropyridine

Synthesis of 4-[3-(1-benzyl-1*H*-1,2,3-triazol-4-yl)phenoxy]-2,3,5,6-tetrafluoropyridine 6

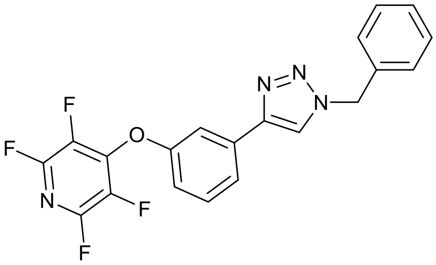

To a solution of **2m** (0.100 g, 0.37 mmol) and benzyl azide (0.049 g, 0.37 mmol) in methanol (10 mL) was added sodium-L-ascorbate (0.015 g, 0.07 mmol) and copper(II) sulfate pentahydrate (0.009 g, 10 mol%).The reaction mixture was heated at 50 °C for 16 hours after which point the reaction mixture

was concentrated under reduced pressure. The residue was purified by flash column chromatography (100% hexane to 100% EtOAc) to give the title compound as a white solid (0.139 g) in 93% yield.

^{1}H NMR (400 MHz, Acetonitrile-d_3) δ 8.16 (s, 1H, triazole C*H*), 7.75 (ddd, *J* = 7.8, 1.5, 0.9, 1H, Ar*H*), 7.62 (dd, *J* = 2.6, 1.5, 1H, Ar*H*), 7.51 (app t, *J* = 8.0, 1H, Ar*H*), 7.46 – 7.35 (m, 5H, Ar*H*), 7.17 (dd, *J* = 8.3, 2.7, 1H, Ar*H*), 5.61 (s, 2H, CH_2).

^{13}C NMR (151 MHz, Acetonitrile-d_3) δ 156.39, 146.12, 135.68, 133.26, 130.73, 128.93, 128.42, 128.02, 122.07, 121.54, 115.99, 113.30, 53.66. Note: due to the solubility of the compound the resonances corresponding to the carbons on the fluoropyridine ring were observed but only weakly therefore these have not been reported.

^{19}F NMR (376 MHz, Acetonitrile-d_3) δ -91.32 – -92.21 (m), -156.12 – -156.62 (m).

HRMS ESI$^+$ Calculated for $[M+H]^+$ $C_{20}H_{13}N_4OF_4^+$ = 401.1025 Found = 401.1046

MP 166-167 °C

Synthesis of 3-(1-benzyl-1*H*-1,2,3-triazol-4-yl)phenol 1b

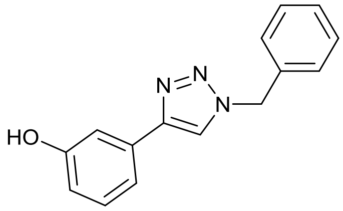

To a solution of 3-hydroxyphenylacetylene (0.100 g, 0.85 mmol) and benzyl azide (0.112 g, 0.85 mmol) in methanol (10 mL) was added sodium-L-ascorbate (0.034 g, 0.17 mmol) and copper(II) sulfate pentahydrate (0.021 g, 10 mol%).The reaction mixture was heated at 50 °C for 3 hours after which point the reaction mixture was concentrated under reduced pressure. The residue was purified by flash column chromatography (100% hexane to 100% EtOAc) to give the title compound as a cream solid (0.199 g) in 93% yield.

^{1}H NMR (400 MHz, Acetonitrile-d_3) δ 8.10 (s, 1H, triazole C*H*), 7.45 – 7.35 (m, 5H, Ar*H*), 7.35 – 7.25 (m, 3H, Ar*H*), 6.84 – 6.78 (m, 1H, Ar*H*), 5.60 (s, 2H, CH_2).

^{13}C NMR (151 MHz, Acetonitrile-d_3) δ 157.31, 147.22, 135.84, 132.30, 130.08, 128.91, 128.35, 127.96, 120.93, 120.92, 114.90, 112.06, 53.59.

HRMS ESI$^+$ Calculated for $[M+H]^+$ $C_{15}H_{14}N_3O^+$ = 252.1137 Found = 252.1152

MP 133-135 °C

Synthesis of (1S,10R,11S,15S)-15-methyl-5-[(2,3,5,6-tetrafluoropyridin-4-yl)oxy]tetracyclo[8.7.0.0^{2,7}.0^{11,15}]heptadeca-2(7),3,5-trien-14-ol 7

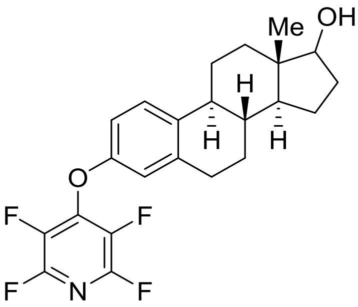

To a solution of **2s** (0.10 g, 0.24 mmol) in THF (10 mL) was added sodium borohydride (0.023 g, 0.60 mmol) and the reaction mixture stirred at rt for 24 h. The reaction mixture was quenched by the addition of water (5 mL) and then concentrated under reduced pressure. The recovered residue was taken up in EtOAc (30 mL) and washed with water (30 mL) and then brine (30 mL). The organic fraction was dried over $MgSO_4$, filtered and concentrated under reduced pressure. This gave the title compound as a white crystalline solid (0.091 g) in 90% yield.

^{1}H NMR (600 MHz, Chloroform-d) δ 7.26 (d, J = 8.6, 1H, ArH), 6.80 (dd, J = 8.6, 2.8, 1H, ArH), 6.74 (d, J = 2.8, 1H, ArH), 3.73 (t, J = 8.5 Hz, 1H, CH), 2.89 – 2.79 (m, 2H, CH_2), 2.34 – 2.27 (m, 1H, CH), 2.25 – 2.17 (m, 1H, CH), 2.16 – 2.08 (m, 1H, CH), 1.96 (dt, J = 12.6, 3.4, 2H, CH_2), 1.92 – 1.86 (m, 1H, CH), 1.75 – 1.65 (m, 1H, CH), 1.55 – 1.16 (m, 8H), 0.78 (s, 3H, CH_3).

^{13}C NMR (151 MHz, Chloroform-d) δ 153.72, 145.09 – 144.80 (m), 144.79 – 144.51 (m), 143.50 – 143.16 (m), 138.98, 137.35, 137.26 – 136.94 (m), 135.52 – 135.17 (m), 126.84, 116.47, 113.68, 81.78, 50.02, 43.98, 43.18, 38.45, 36.61, 30.55, 29.58, 26.93, 26.17, 23.09, 11.02.

^{19}F NMR (376 MHz, Chloroform-d) δ -88.82 – -89.09 (m), -154.29 – -154.55 (m).

HRMS ESI$^-$ Calculated for [M-H]$^-$ $C_{23}H_{22}NO_2F_4^-$ = 420.1587 Found = 420.1585

MP 99-101 °C

NMR Data for Synthesised Compounds

04131119.10.fid
SLC:WDGB:WB3-62 F5-8

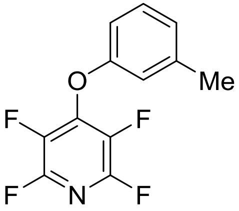

2a

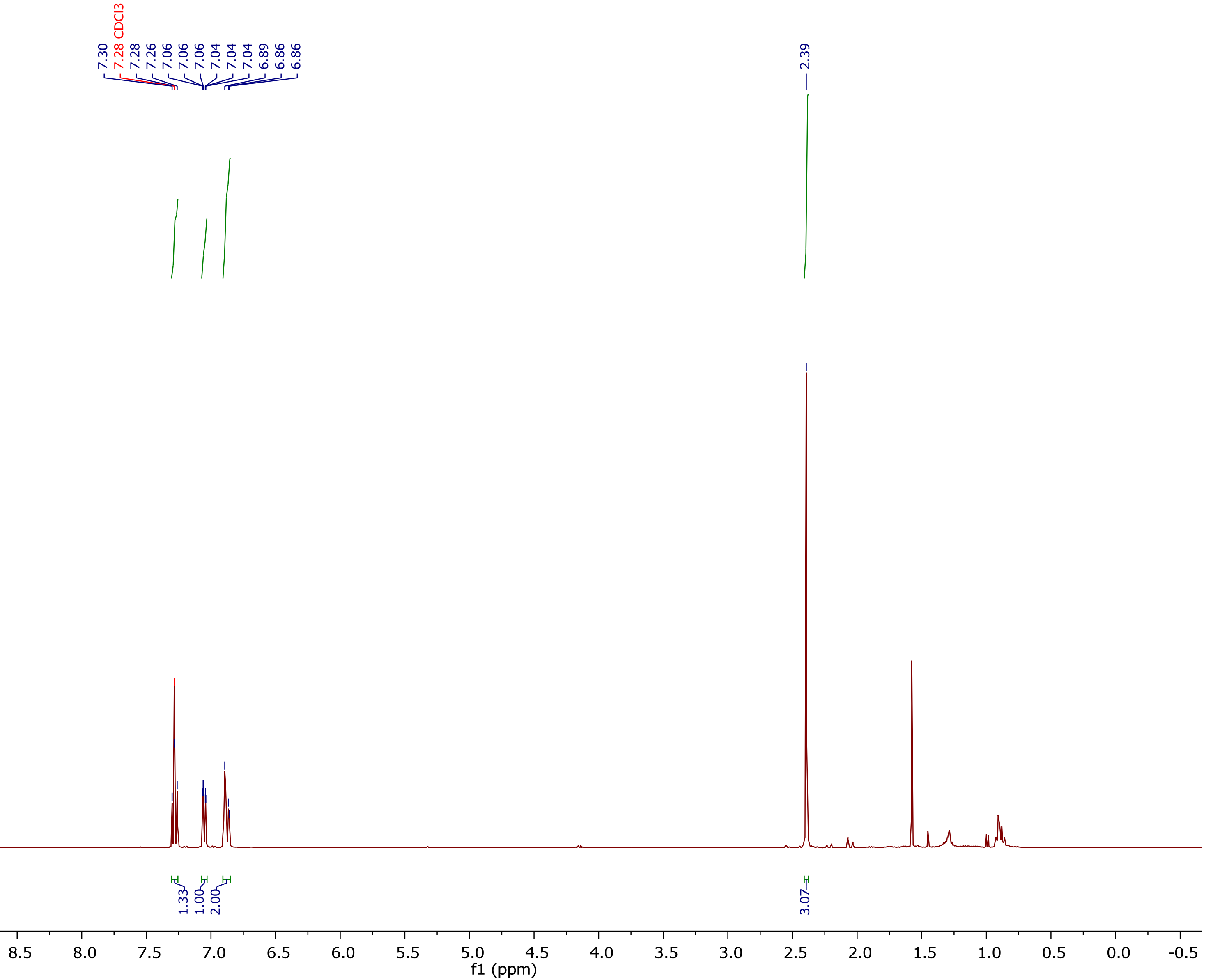

04131119.13.fid
SLC:WDGB:WB3-62:F5-8

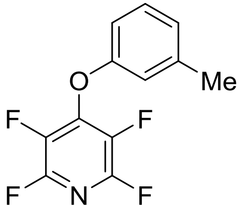

2a

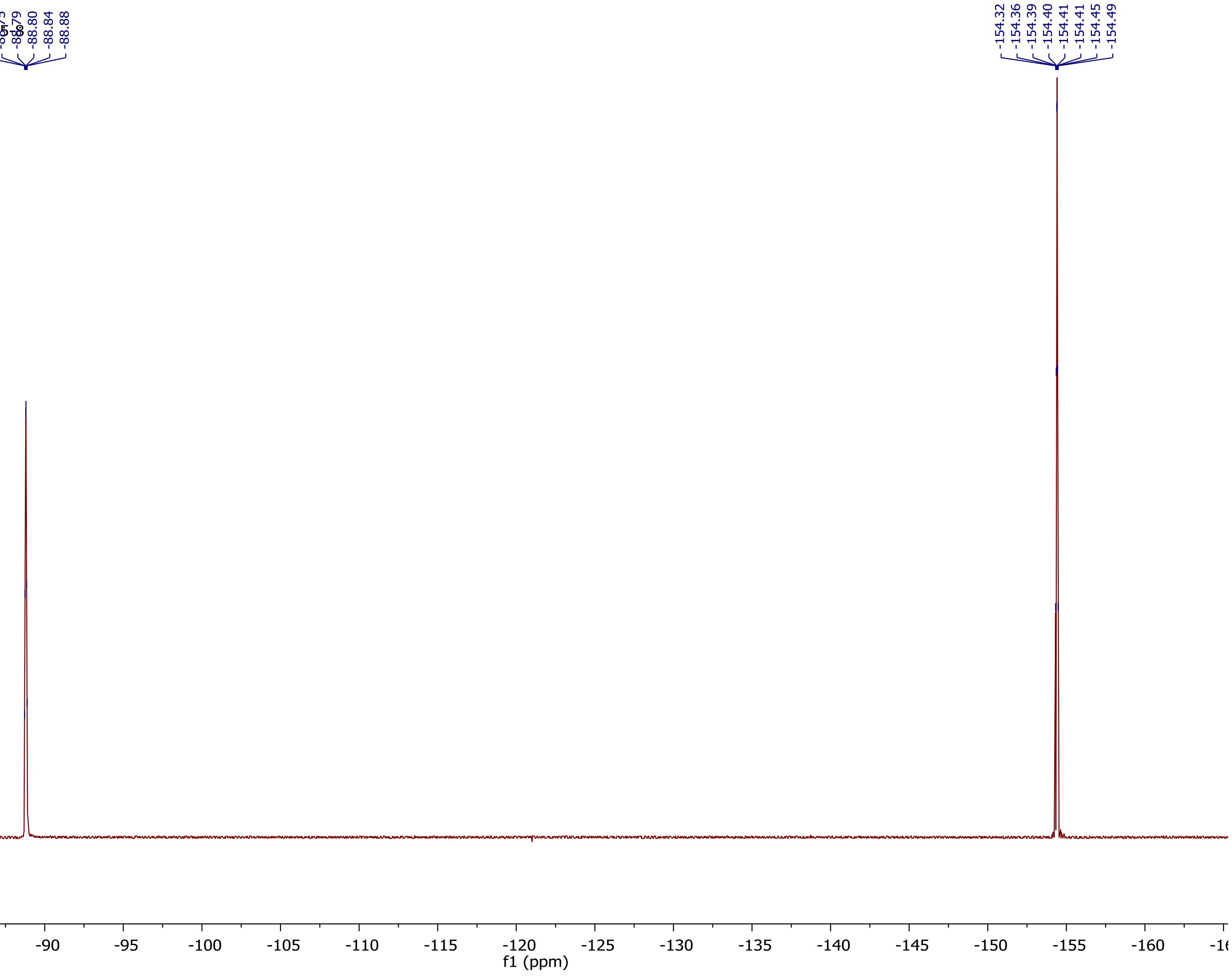

CARBON_01
SLC:WB:3-62

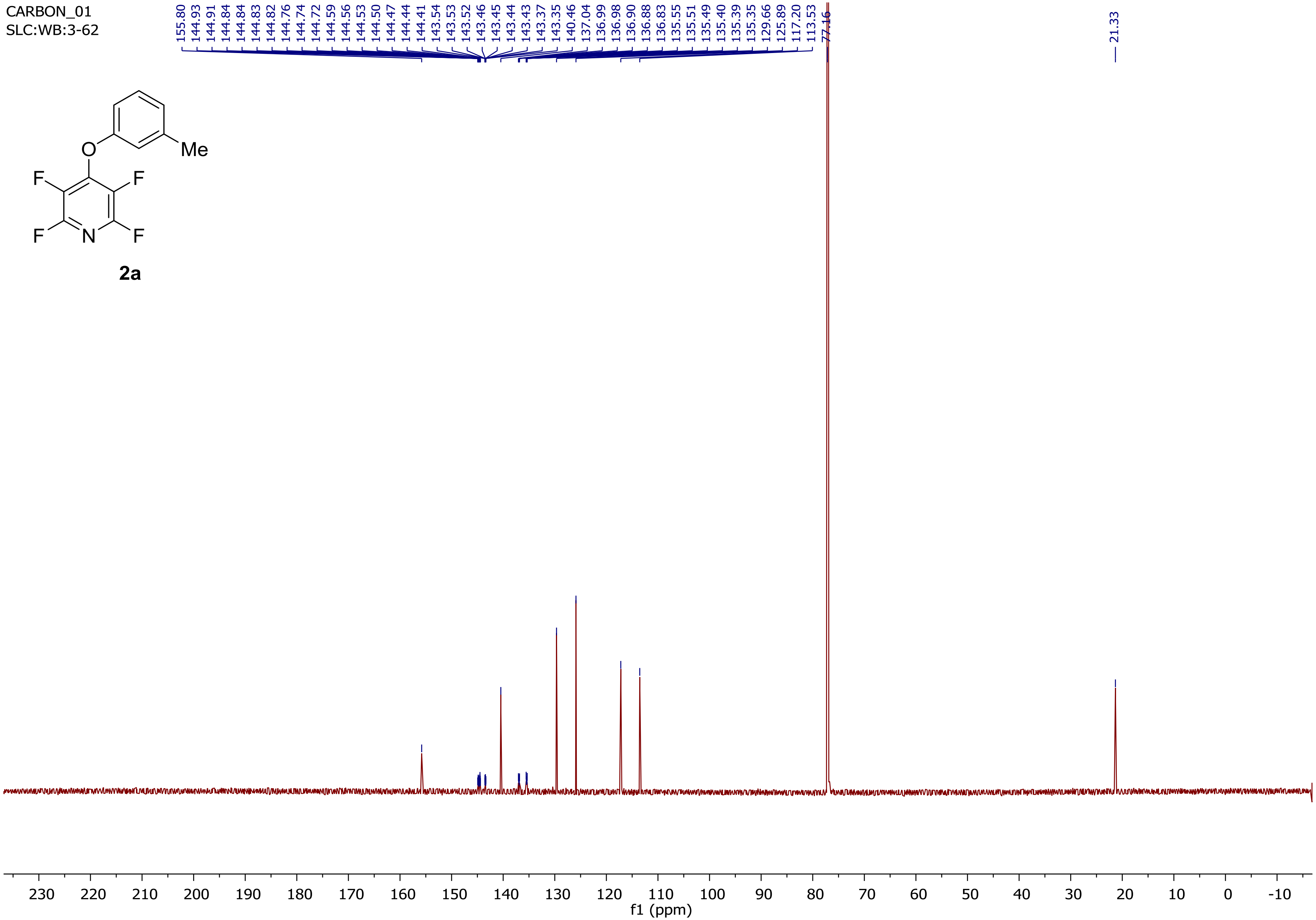

2a

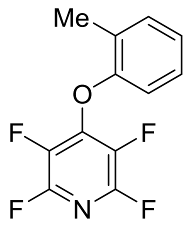

2b

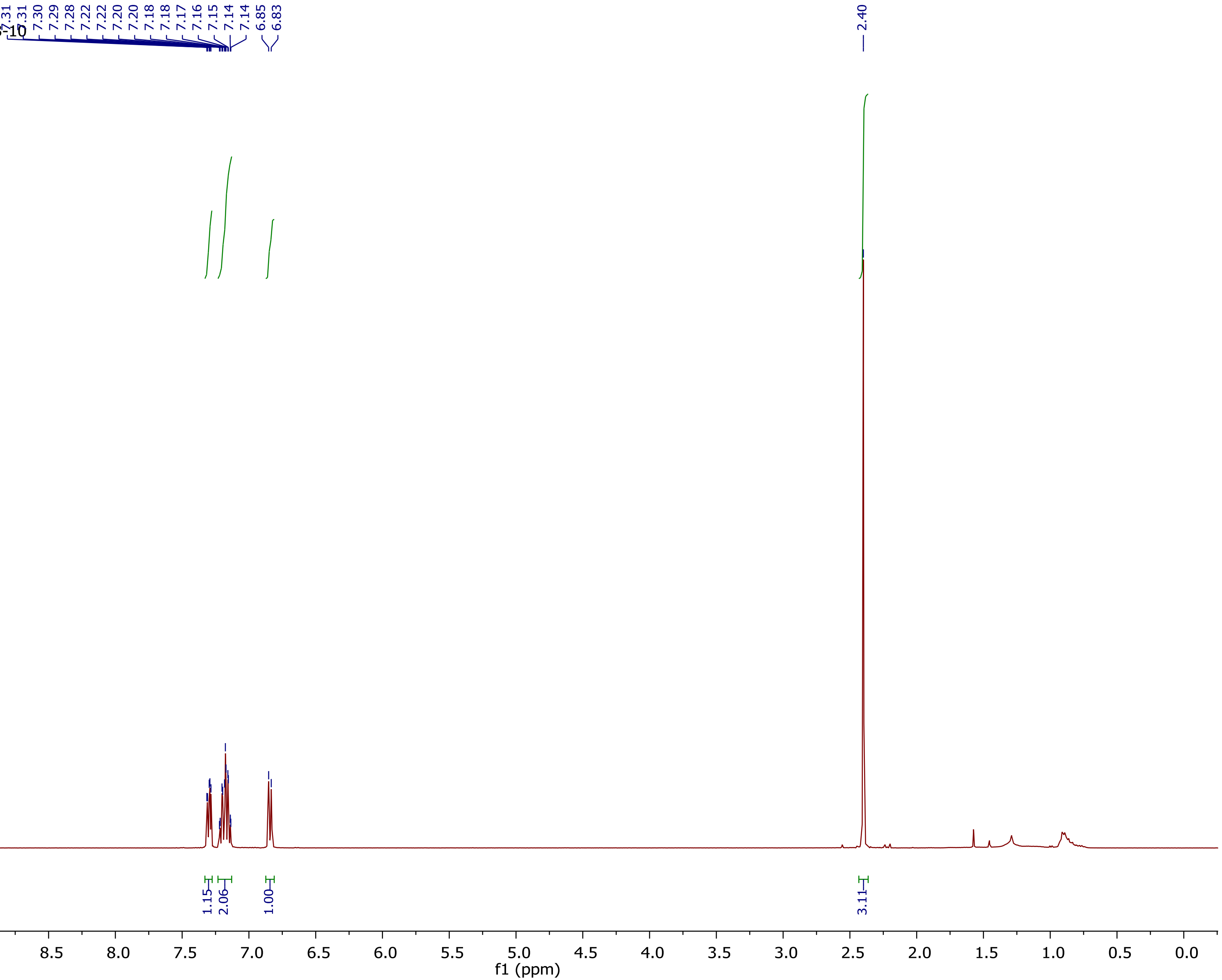
15171453.10.fid
SLC:WDGB:WB3-82 F3-10
7.31 7.31 7.30 7.29 7.28 7.22 7.22 7.20 7.20 7.18 7.18 7.17 7.16 7.15 7.14 7.14 6.85 6.83
2.40
1.15
2.06
1.00
3.11
10.0 9.5 9.0 8.5 8.0 7.5 7.0 6.5 6.0 5.5 5.0 4.5 4.0 3.5 3.0 2.5 2.0 1.5 1.0 0.5 0.0
f1 (ppm)

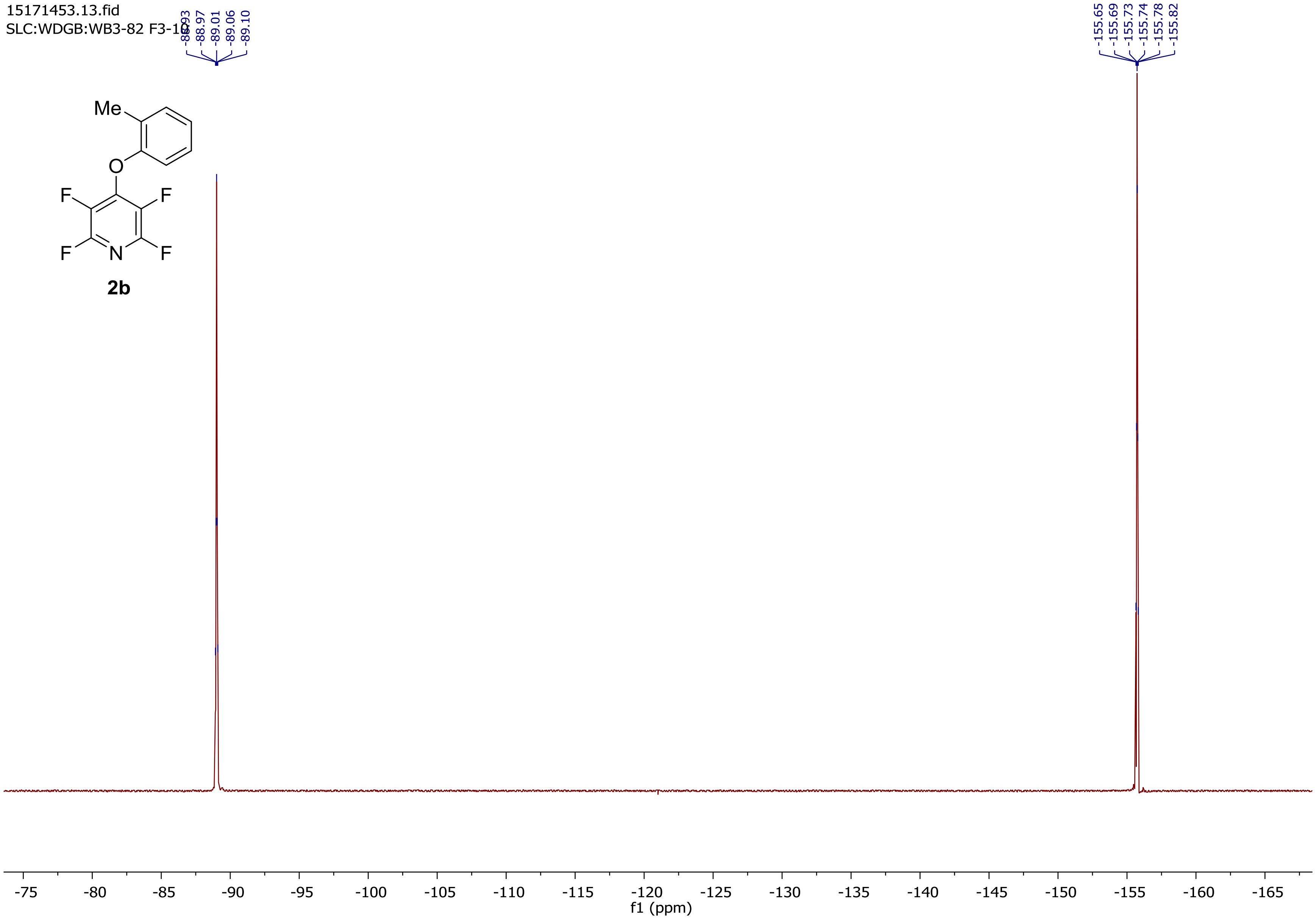

15171453.13.fid
SLC:WDGB:WB3-82 F3-10
-88.93
-88.97
-89.01
-89.06
-89.10
-155.65
-155.69
-155.73
-155.74
-155.78
-155.82
Me
O
F
F
F
N
F
2b
-75
-80
-85
-90
-95
-100
-105
-110
-115
-120
-125
-130
-135
-140
-145
-150
-155
-160
-165
f1 (ppm)

CARBON_01
SLC:WB:3-82

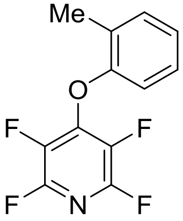

2b

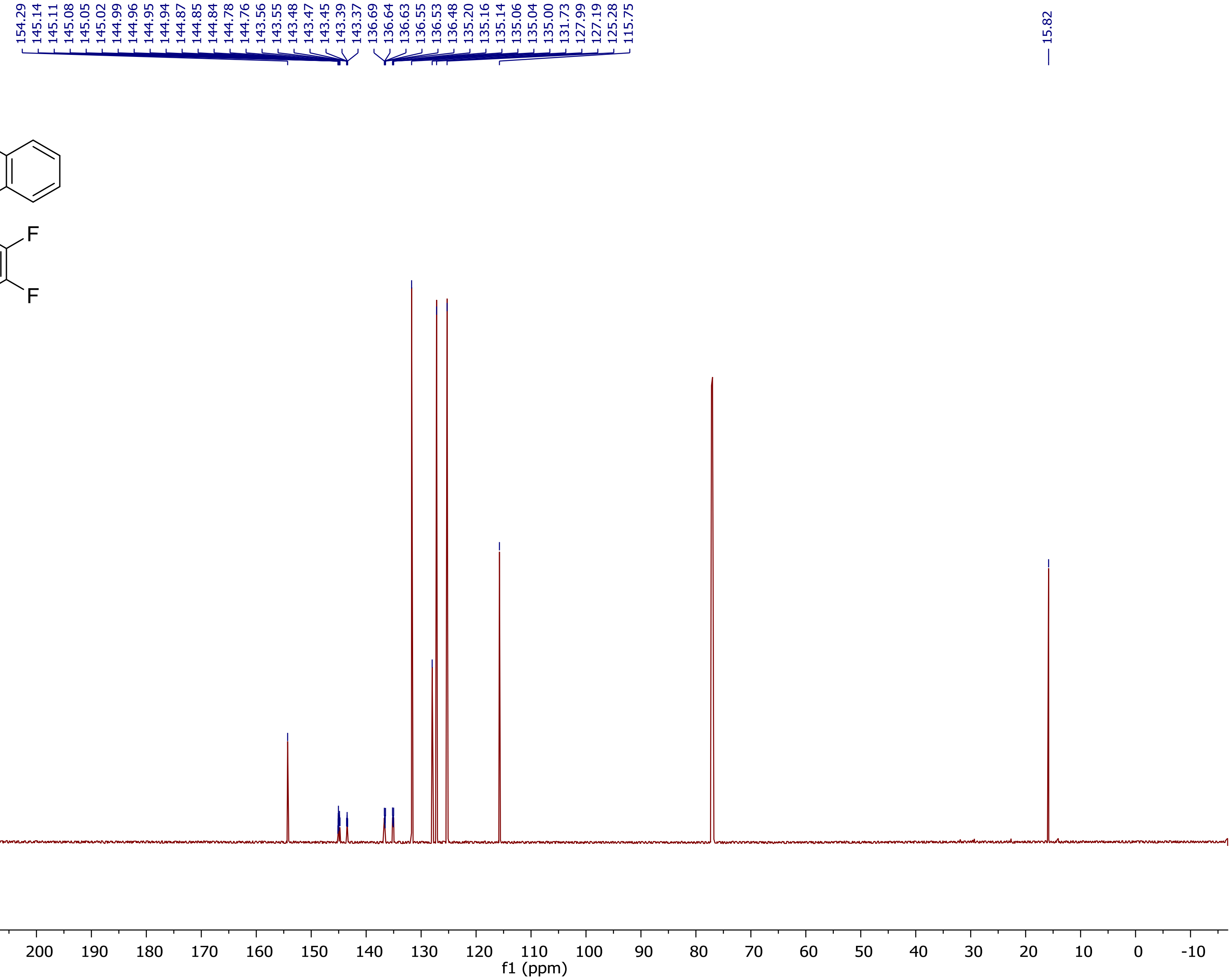

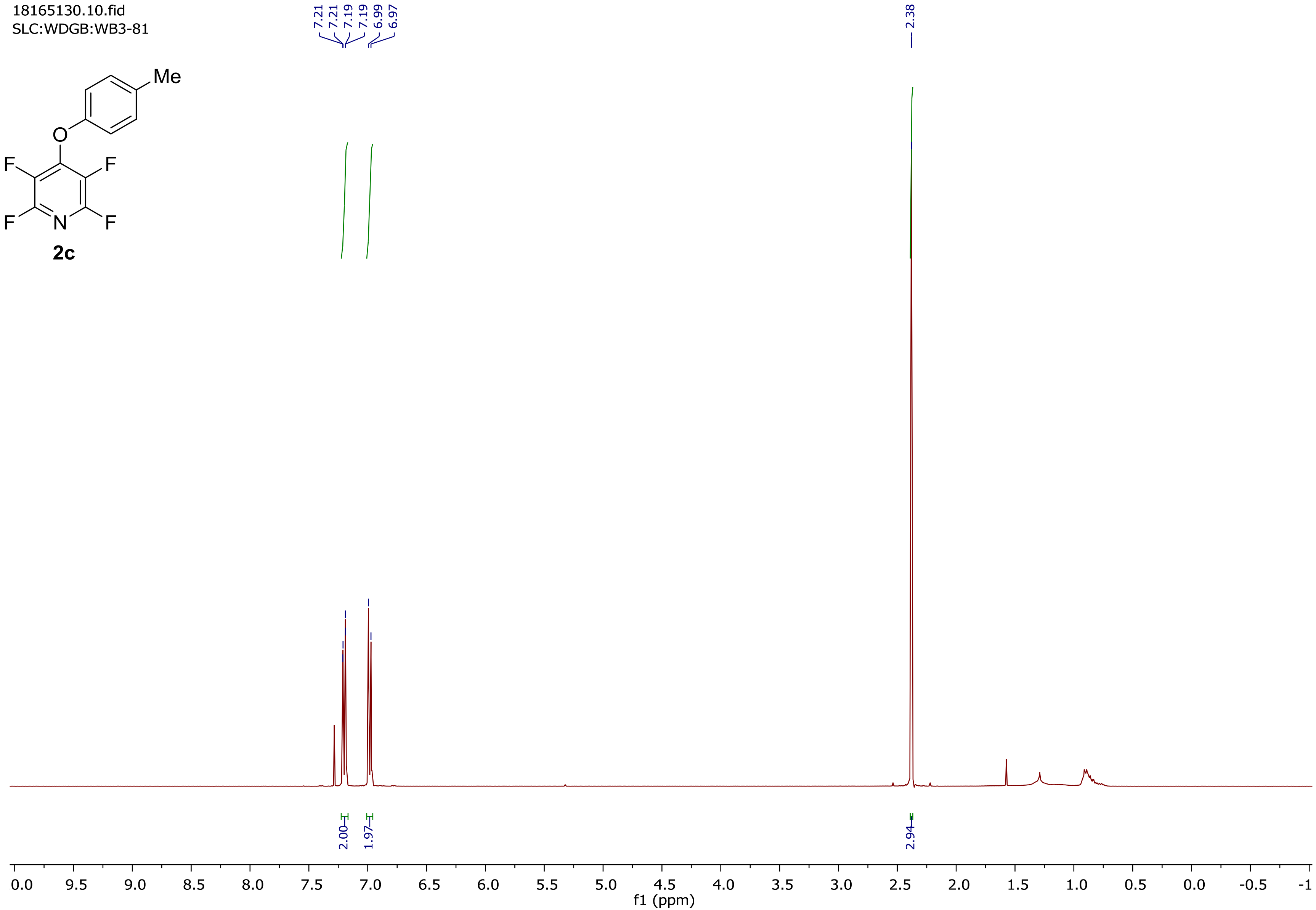
18165130.10.fid
SLC:WDGB:WB3-81
Me
O
F
F
F
N
F
2c
7.21
7.21
7.19
7.19
6.99
6.97
2.38
2.00
1.97
2.94
f1 (ppm)

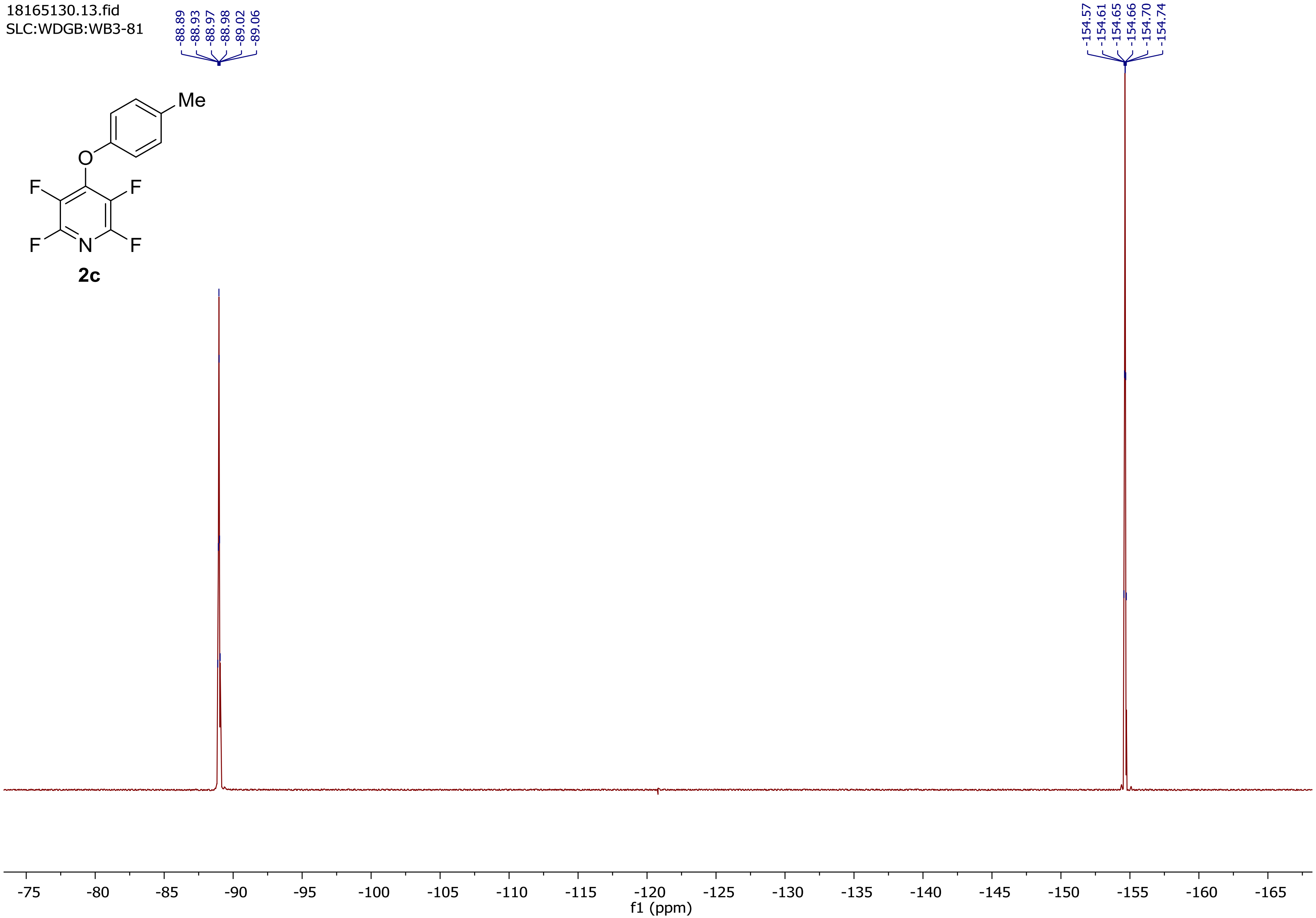
18165130.13.fid
SLC:WDGB:WB3-81
-88.89
-88.93
-88.97
-88.98
-89.02
-89.06
-154.57
-154.61
-154.65
-154.66
-154.70
-154.74
Me
O
F
F
F
F
N
2c
-75
-80
-85
-90
-95
-100
-105
-110
-115
-120
-125
-130
-135
-140
-145
-150
-155
-160
-165
f1 (ppm)

CARBON_01
SLC:WB:3-81

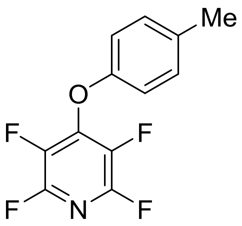

2c

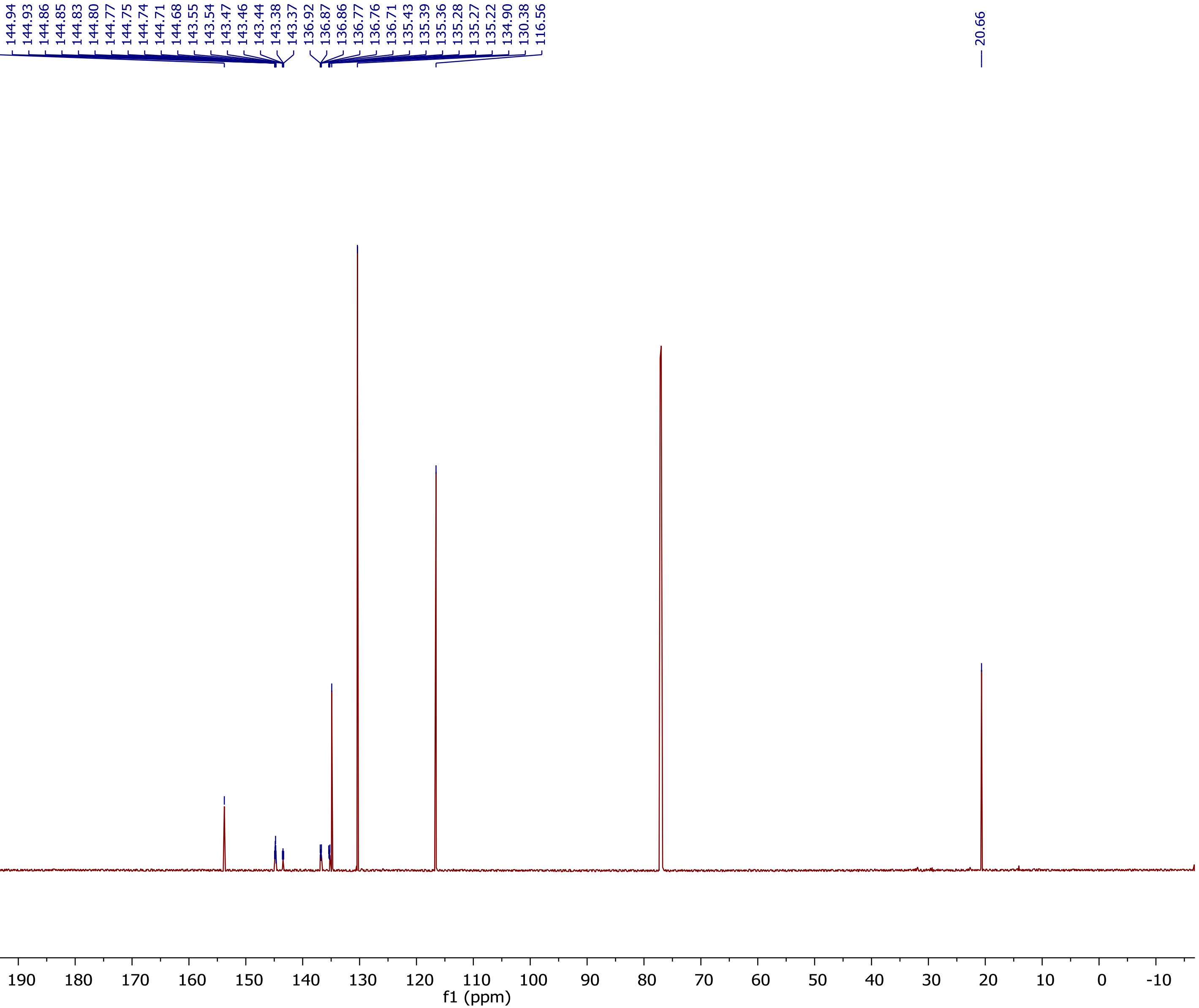

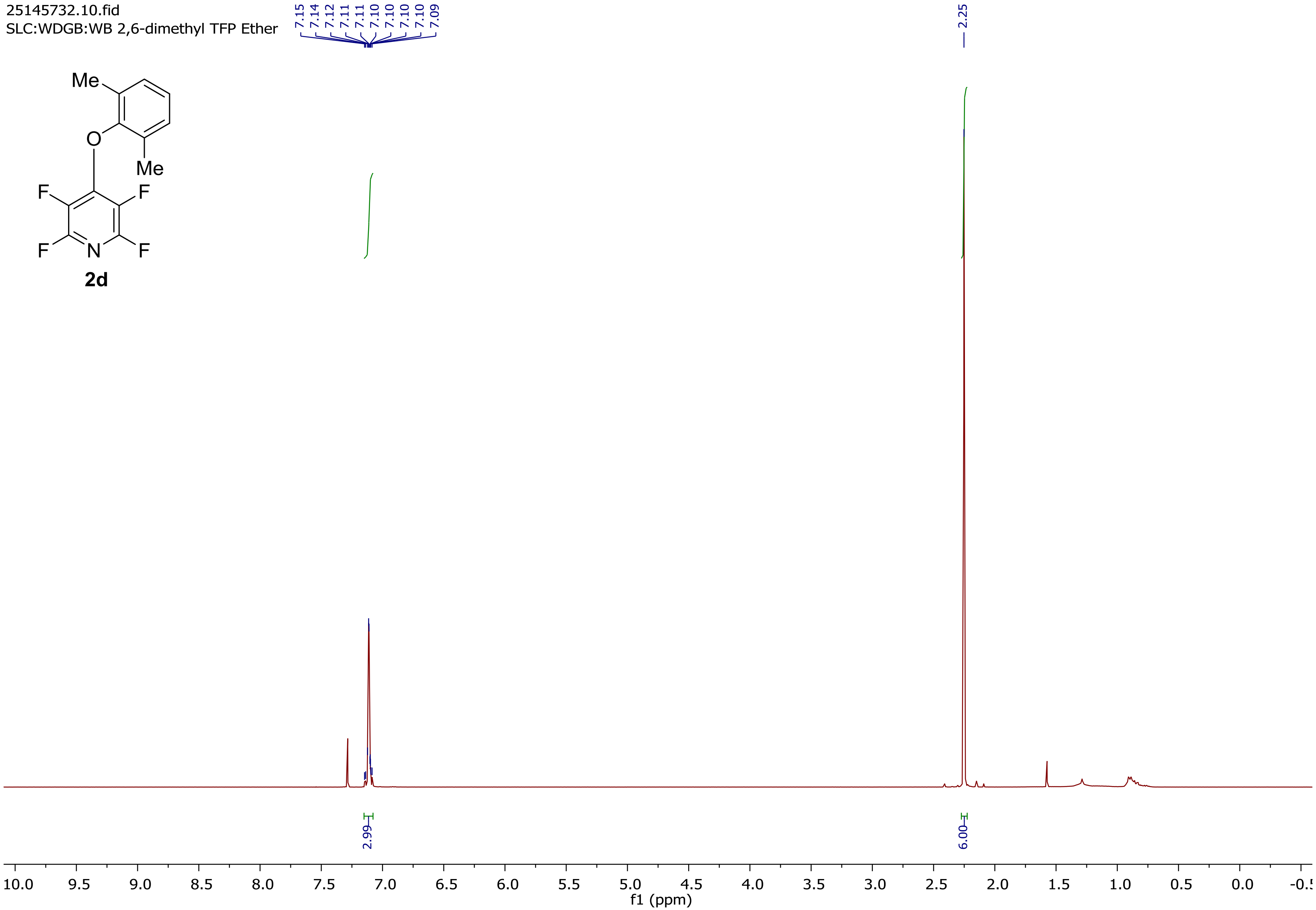
25145732.10.fid
SLC:WDGB:WB 2,6-dimethyl TFP Ether
7.15
7.14
7.12
7.11
7.11
7.10
7.10
7.10
7.10
7.09
2.25
Me
O
Me
F
F
F
N
F
2d
2.99
6.00
10.0
9.5
9.0
8.5
8.0
7.5
7.0
6.5
6.0
5.5
5.0
4.5
4.0
3.5
3.0
2.5
2.0
1.5
1.0
0.5
0.0
f1 (ppm)

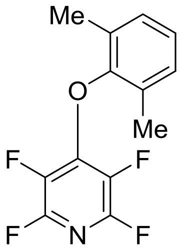
Me
O
Me
F
F
F
N
F

2d

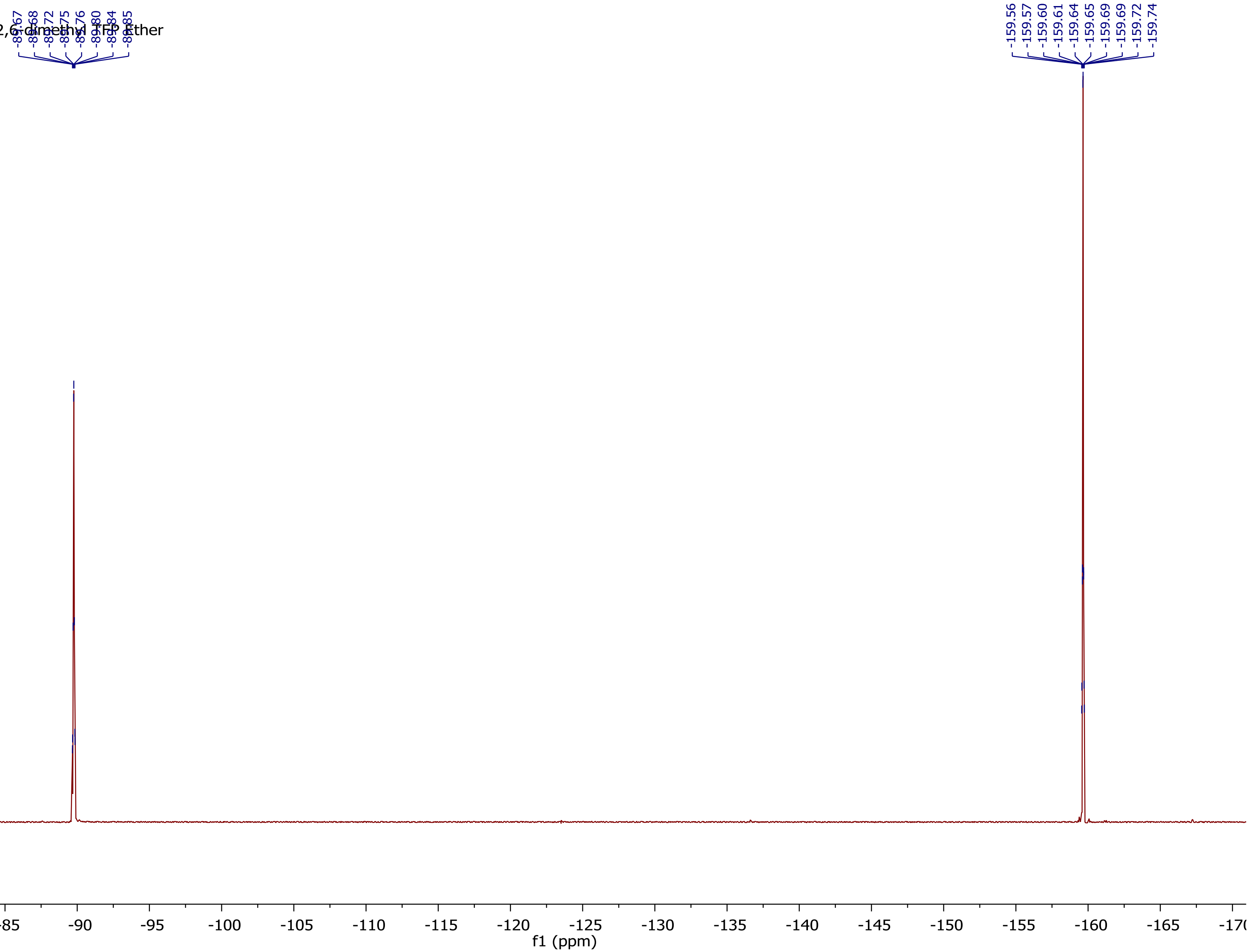
25145732.13.fid
SLC:WDGB:WB 2,6-dimethyl TFP Ether
-89.67
-89.68
-89.72
-89.75
-89.76
-89.80
-89.84
-89.85
-159.56
-159.57
-159.60
-159.61
-159.64
-159.65
-159.69
-159.69
-159.72
-159.74
-80
-85
-90
-95
-100
-105
-110
-115
-120
-125
-130
-135
-140
-145
-150
-155
-160
-165
f1 (ppm)

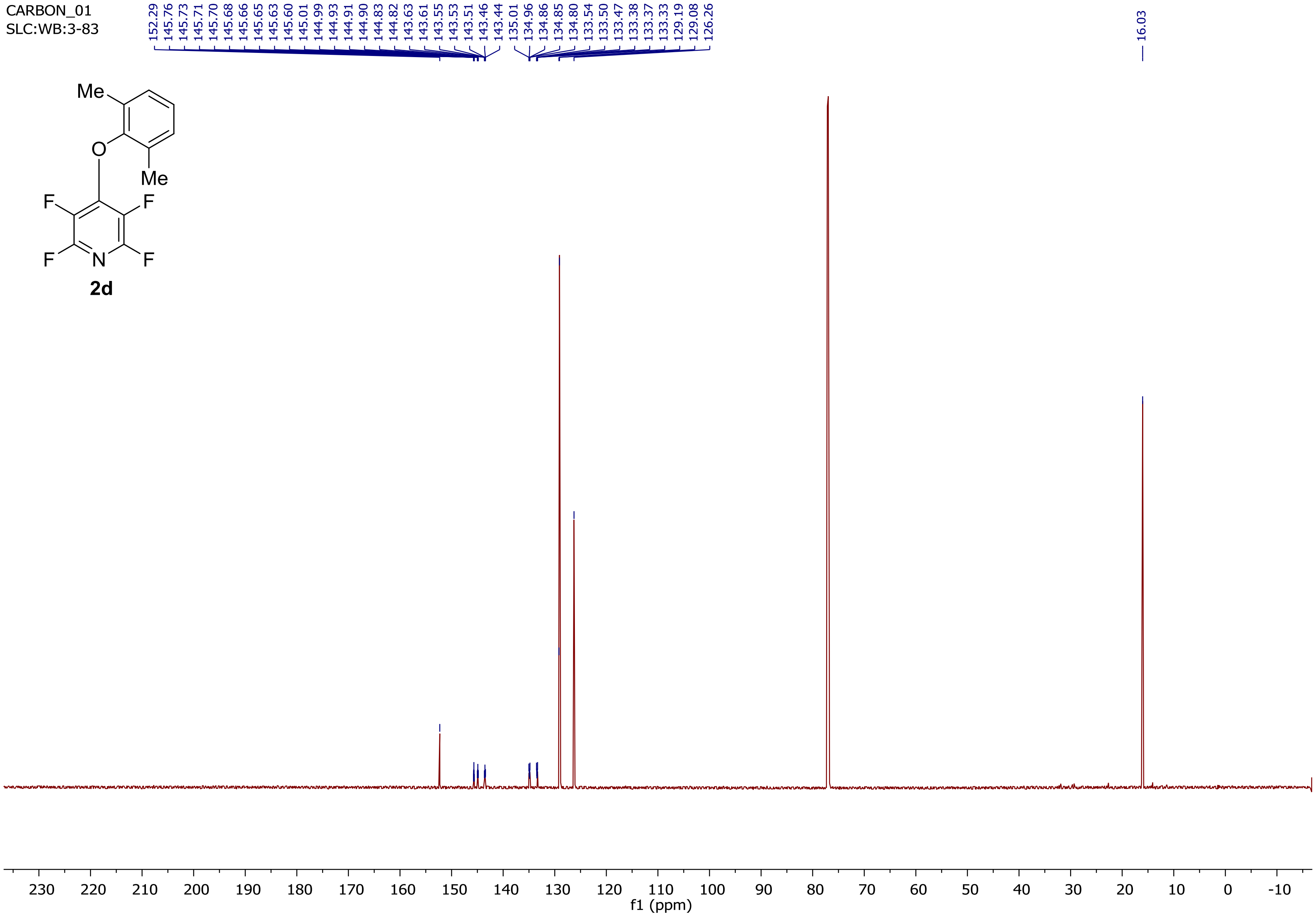
CARBON_01
SLC:WB:3-83
152.29
145.76
145.73
145.71
145.70
145.68
145.66
145.65
145.63
145.60
145.01
144.99
144.93
144.91
144.90
144.83
144.82
143.63
143.61
143.55
143.53
143.51
143.46
143.44
135.01
134.96
134.86
134.85
134.80
133.54
133.50
133.47
133.38
133.37
133.33
129.19
129.08
126.26
16.03
Me
O
Me
F
F
F
N
F
2d
230 220 210 200 190 180 170 160 150 140 130 120 110 100 90 80 70 60 50 40 30 20 10 0 -10
f1 (ppm)

18174556.10.fid
SLC:WDGB:WB3-77

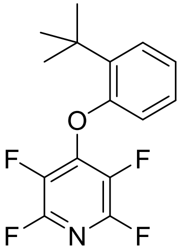

2e

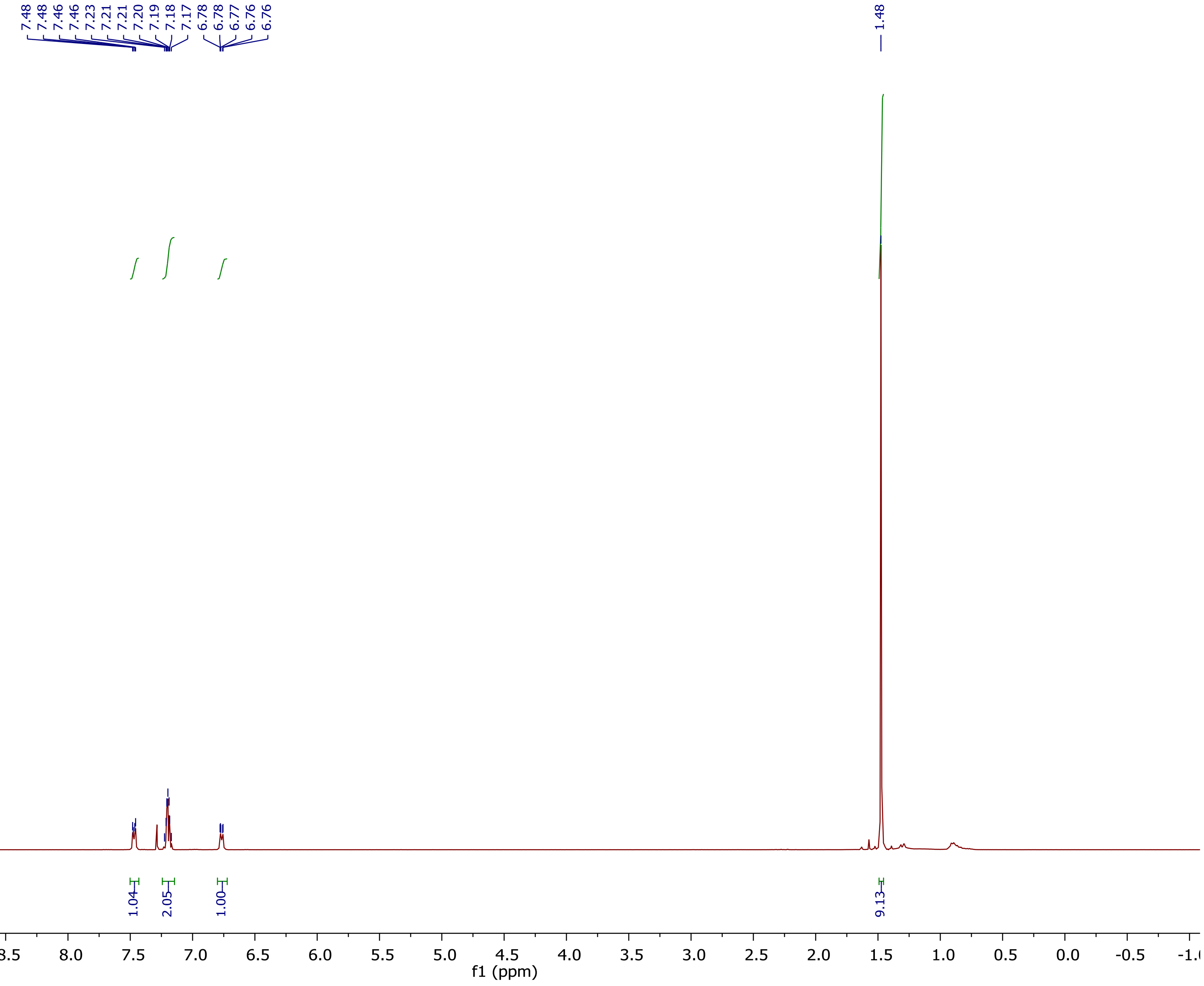

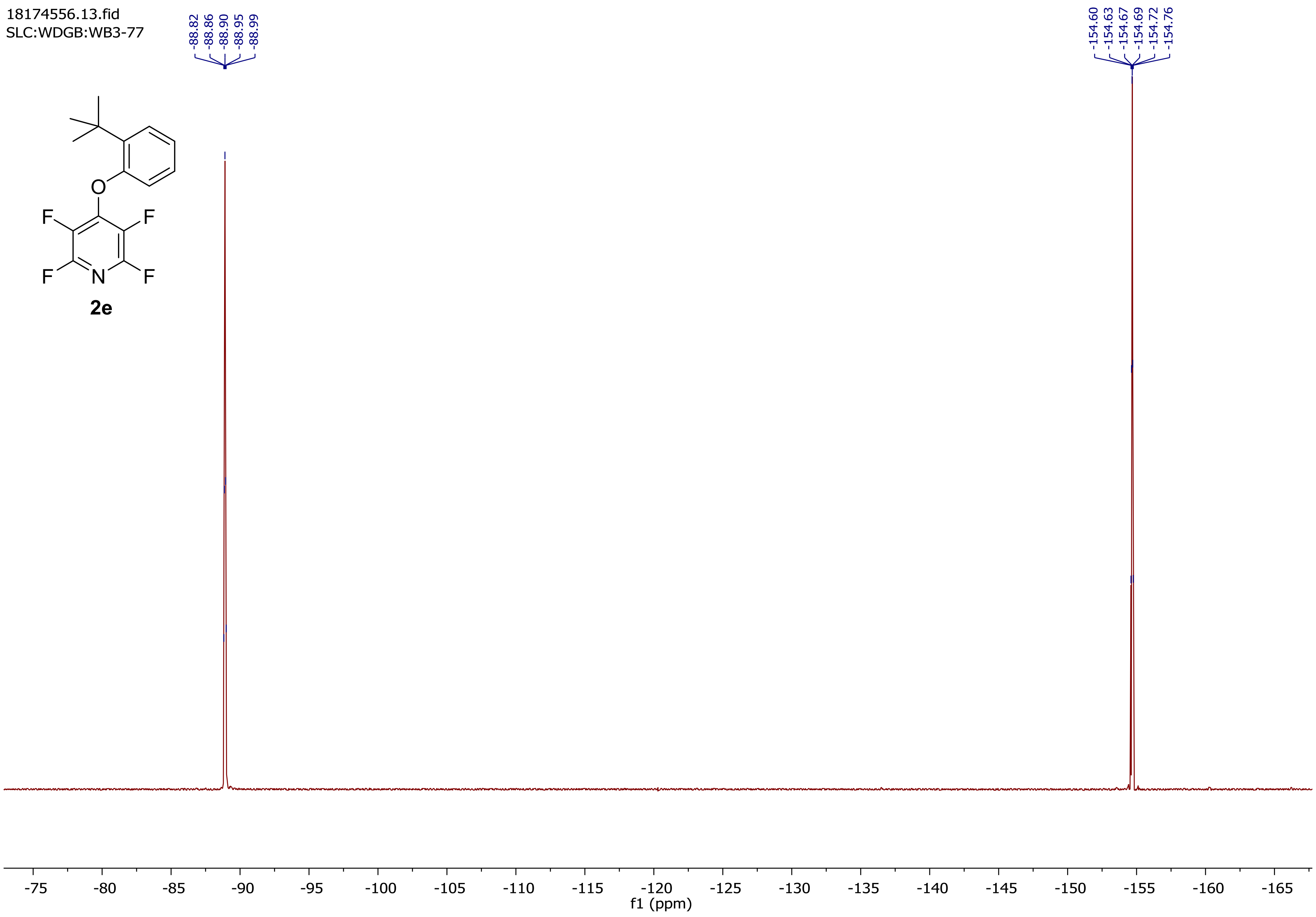
18174556.13.fid
SLC:WDGB:WB3-77
-88.82
-88.86
-88.90
-88.95
-88.99
-154.60
-154.63
-154.67
-154.69
-154.72
-154.76
O
F
F
F
N
F
2e
-75
-80
-85
-90
-95
-100
-105
-110
-115
-120
-125
-130
-135
-140
-145
-150
-155
-160
-165
f1 (ppm)

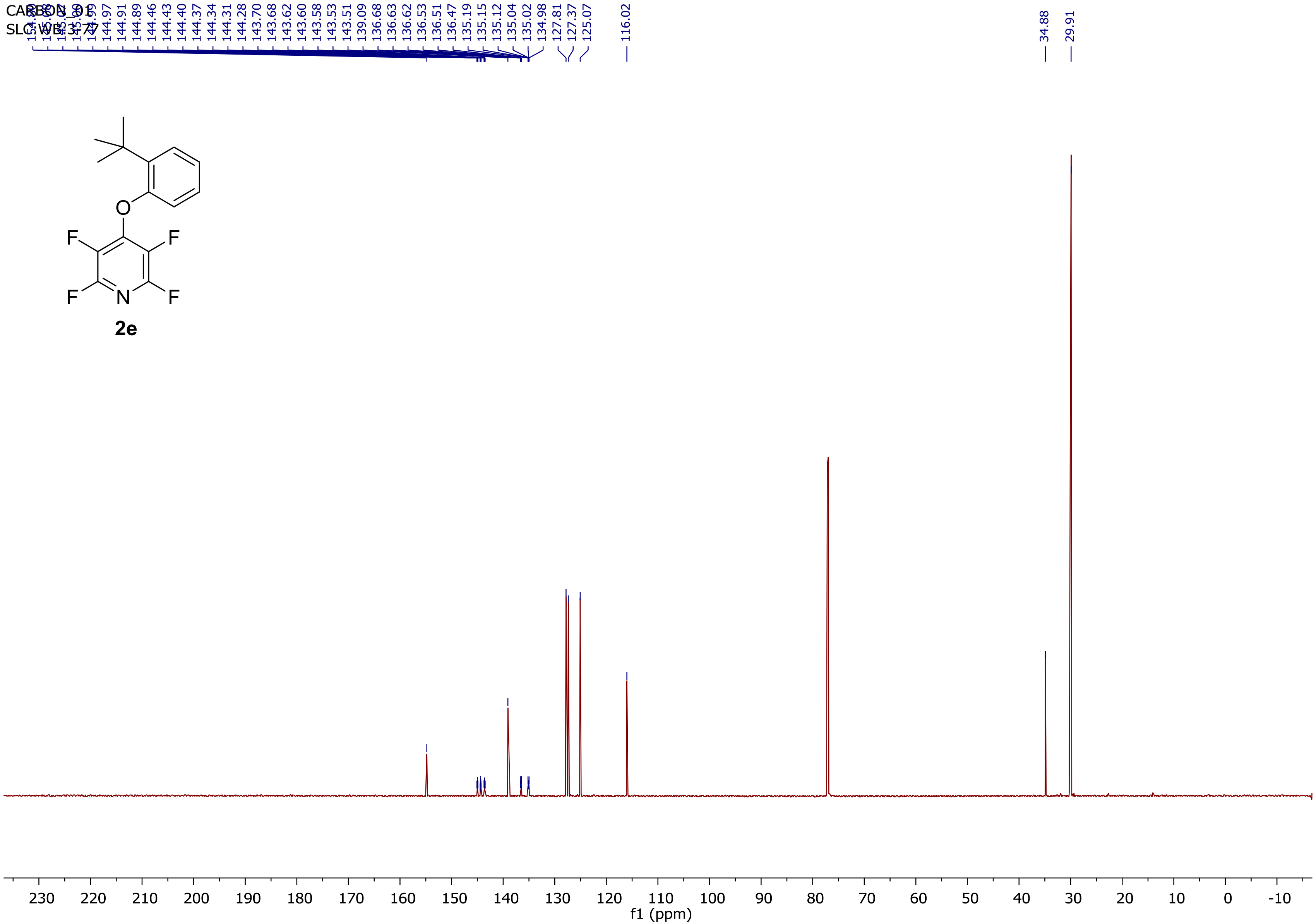

CARBON_01
SLC-WB3-77
144.97
144.91
144.89
144.46
144.43
144.40
144.37
144.34
144.31
144.28
143.70
143.68
143.62
143.60
143.58
143.53
143.51
139.09
136.68
136.63
136.62
136.53
136.51
136.47
135.19
135.15
135.12
135.04
135.02
134.98
127.81
127.37
125.07
116.02
34.88
29.91
O
F
N
2e
230 220 210 200 190 180 170 160 150 140 130 120 110 100 90 80 70 60 50 40 30 20 10 0 -10
f1 (ppm)

18165110.10.fid
SLC:WDGB:WB3-79

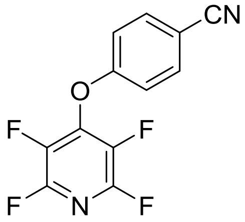

2g

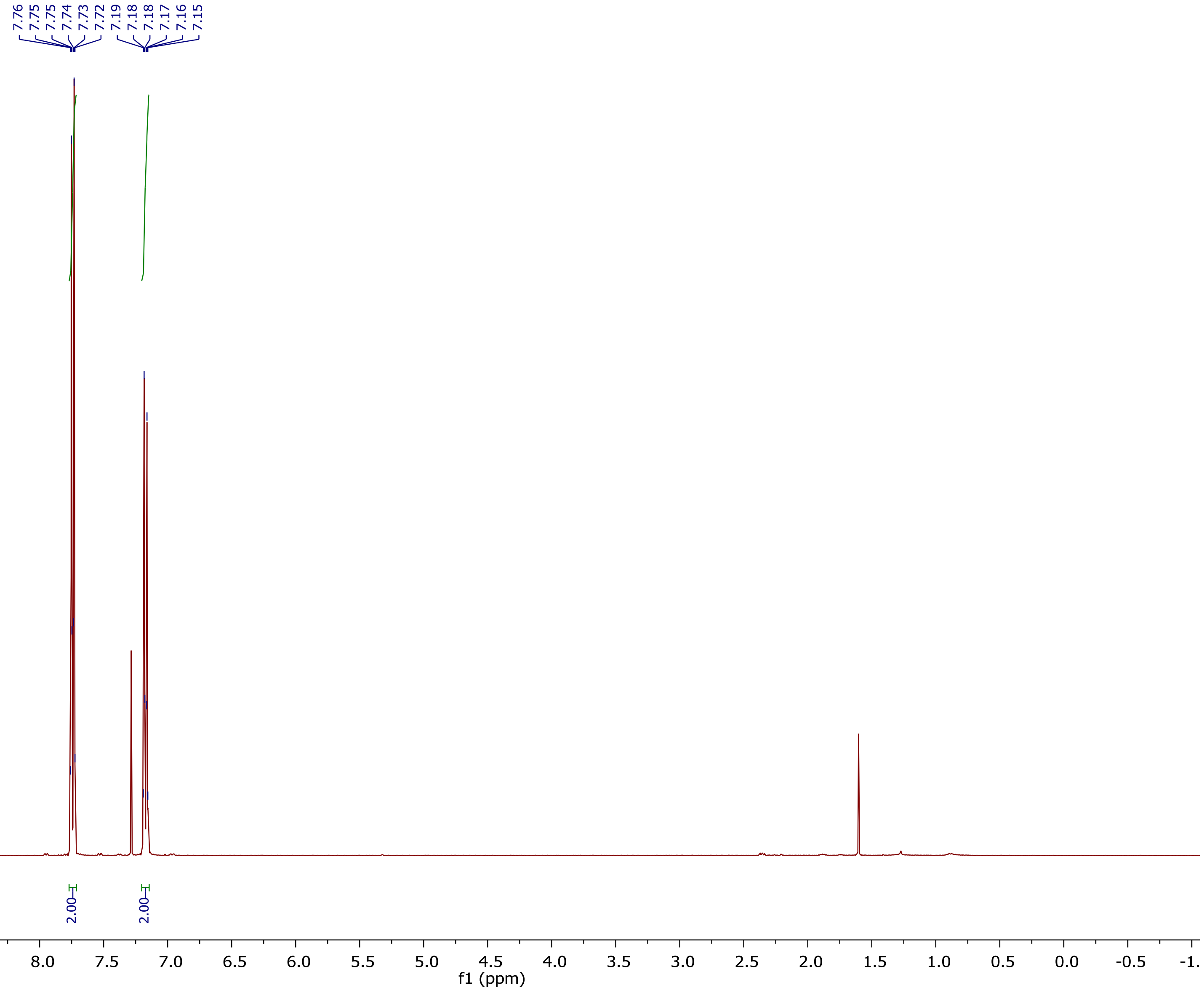

18165110.13.fid
SLC:WDGB:WB3-79

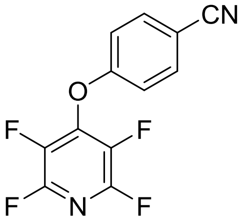

2g

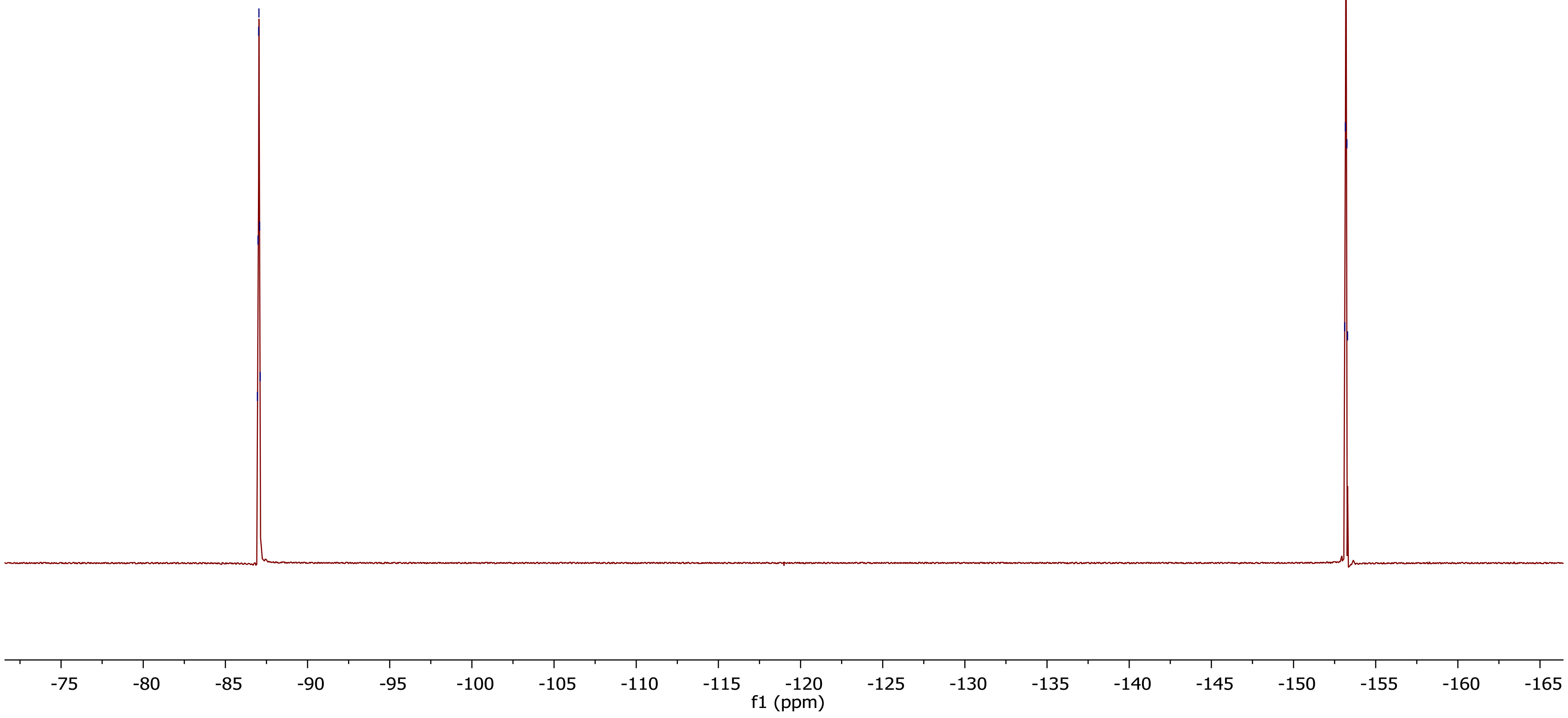

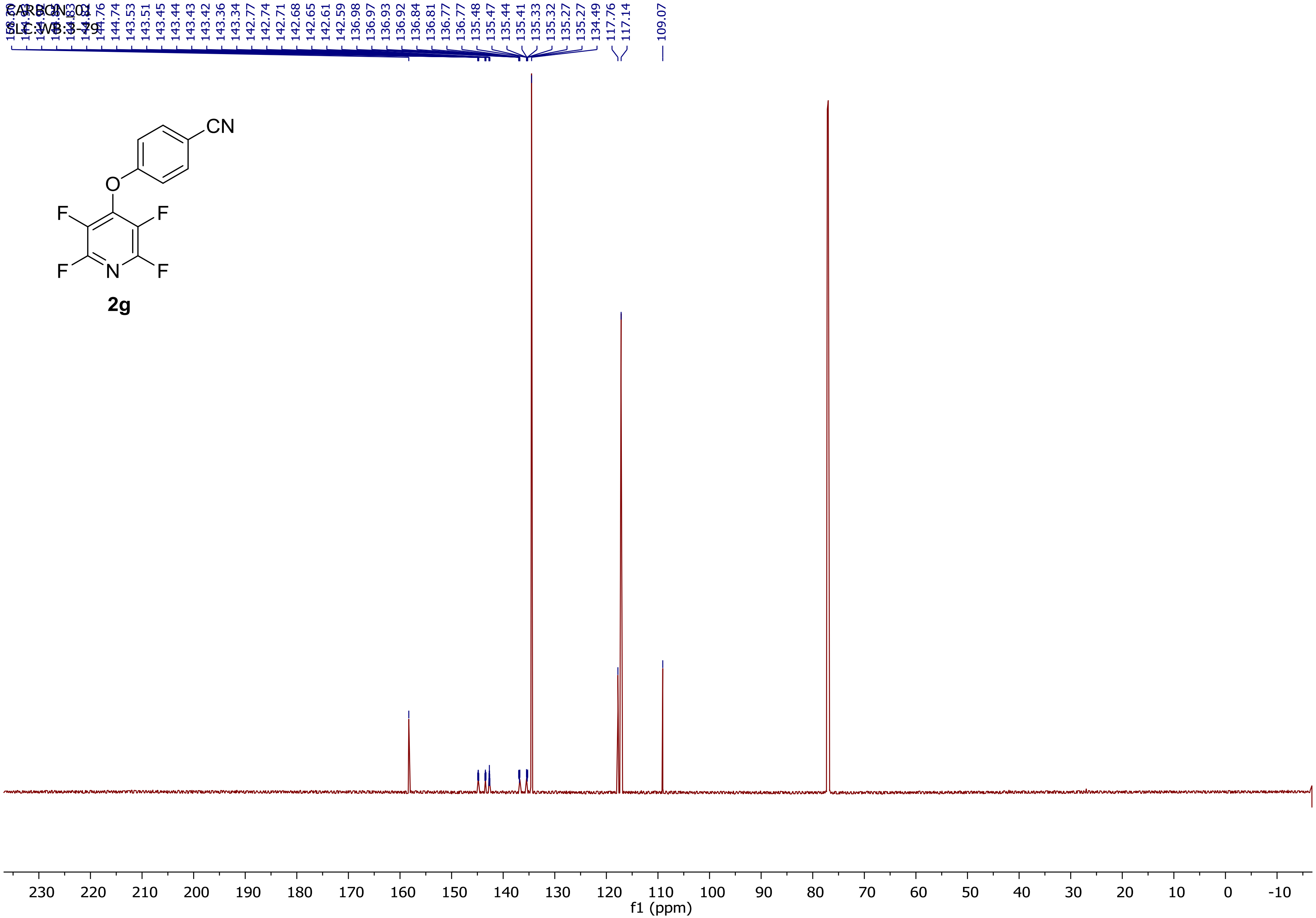

CARBON_01
144.76
144.74
143.53
143.51
143.45
143.44
143.43
143.42
143.36
143.34
142.77
142.74
142.71
142.68
142.65
142.61
142.59
136.98
136.97
136.93
136.92
136.84
136.81
136.77
136.77
135.48
135.47
135.44
135.41
135.33
135.32
135.27
135.27
134.49
117.76
117.14
109.07
CN
O
F
F
F
N
F
2g
230 220 210 200 190 180 170 160 150 140 130 120 110 100 90 80 70 60 50 40 30 20 10 0 -10
f1 (ppm)

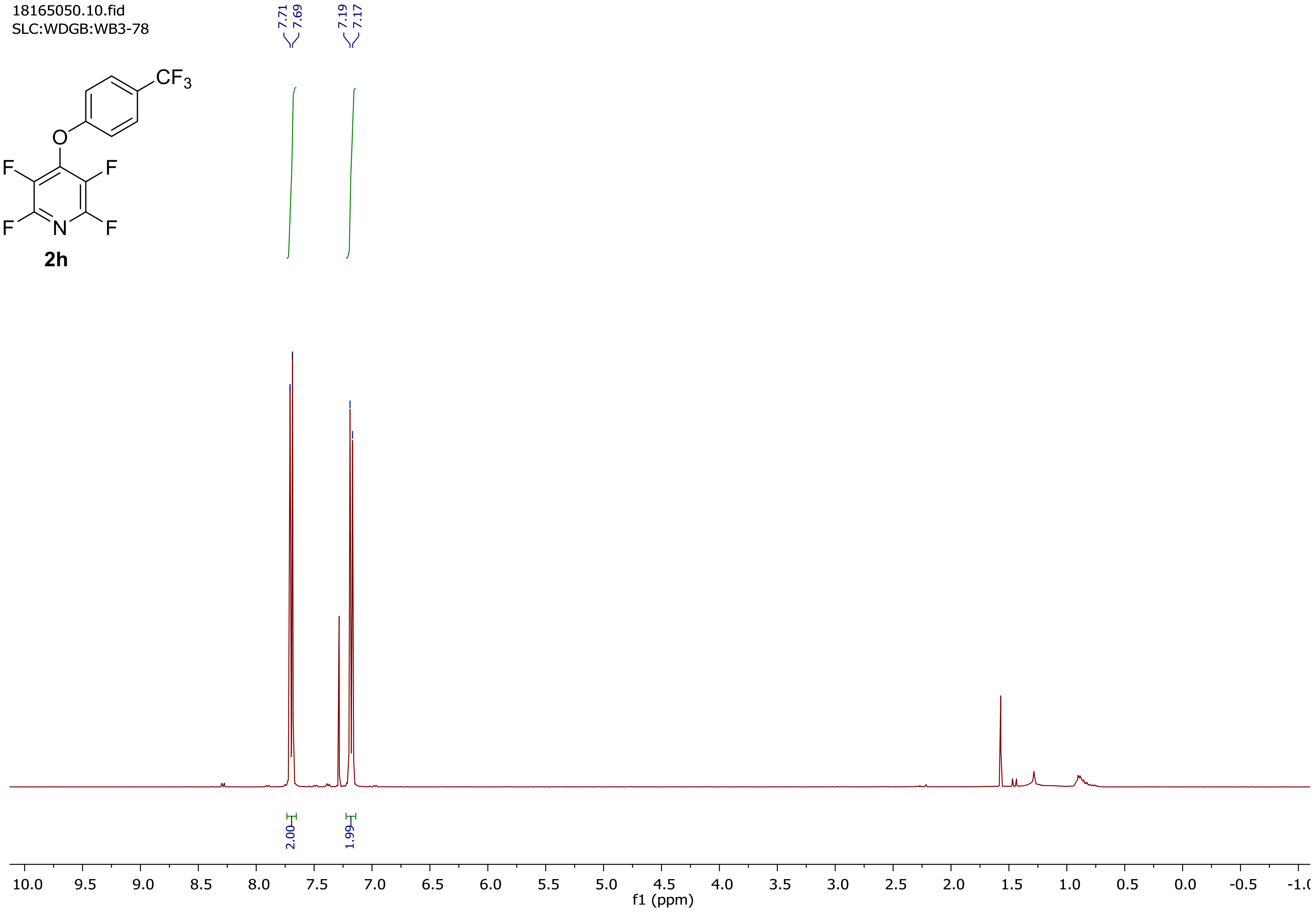
18165050.10.fid
SLC:WDGB:WB3-78
7.71
7.69
7.19
7.17
CF3
O
F
F
F
N
F
2h
2.00
1.99
10.0 9.5 9.0 8.5 8.0 7.5 7.0 6.5 6.0 5.5 5.0 4.5 4.0 3.5 3.0 2.5 2.0 1.5 1.0 0.5 0.0 -0.5 -1.0
f1 (ppm)

18165050.13.fid
SLC:WDGB:WB3-78

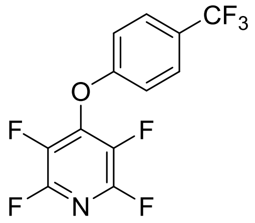

2h

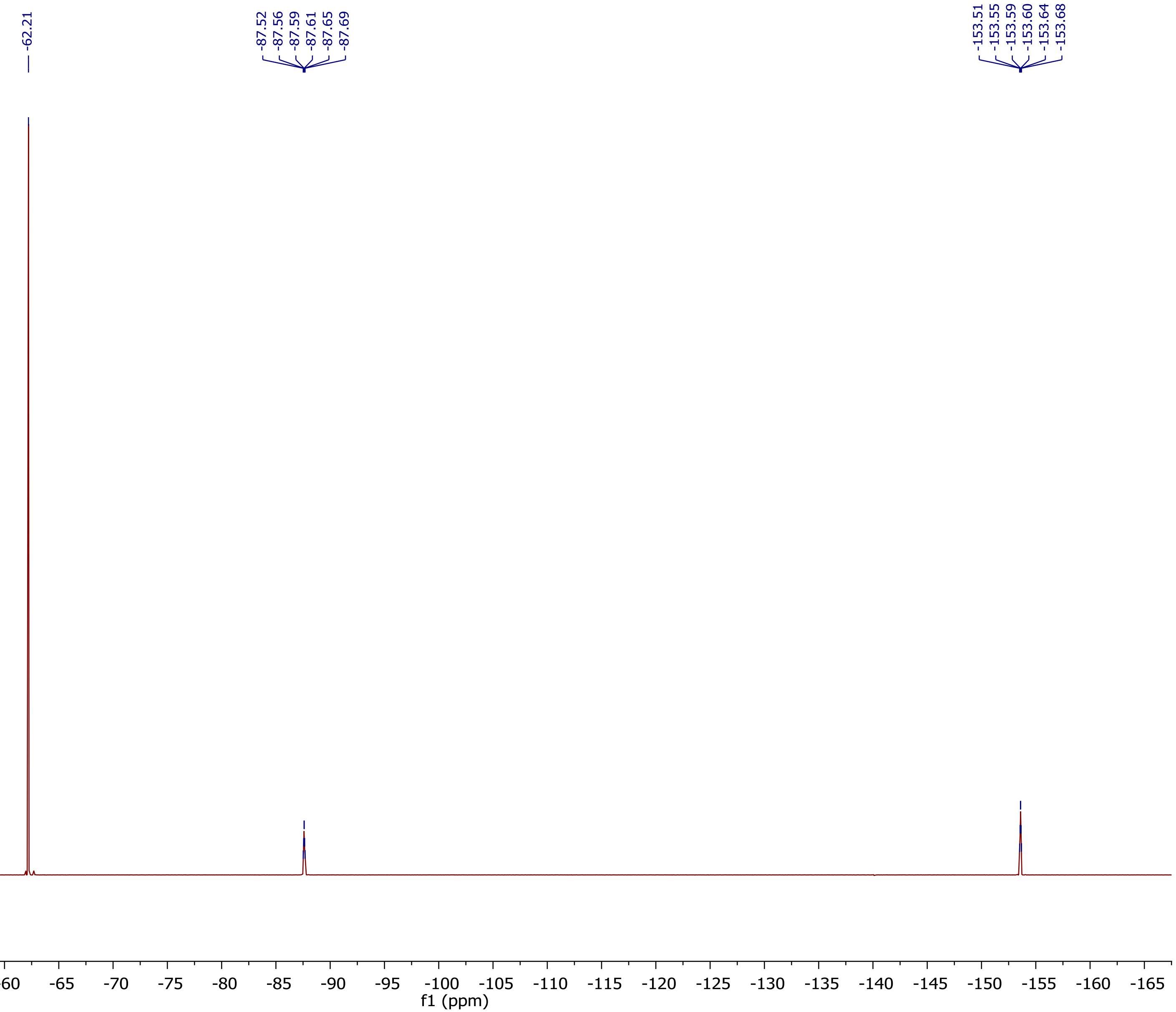

CARBON_01
SLC:WB:3-78

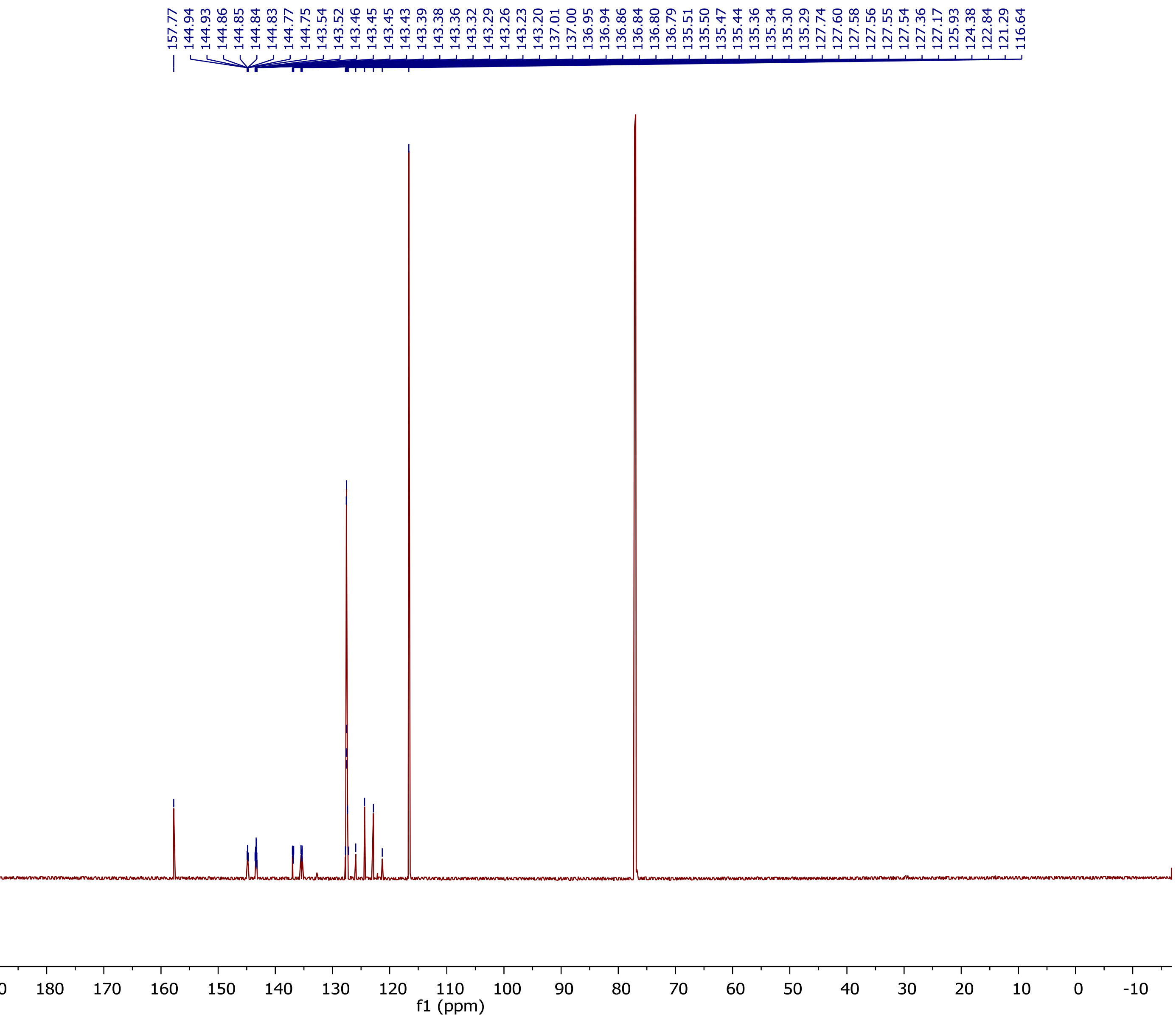

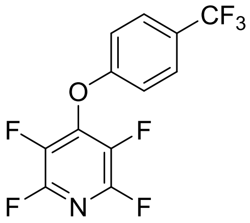

2h

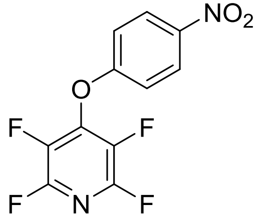

2i

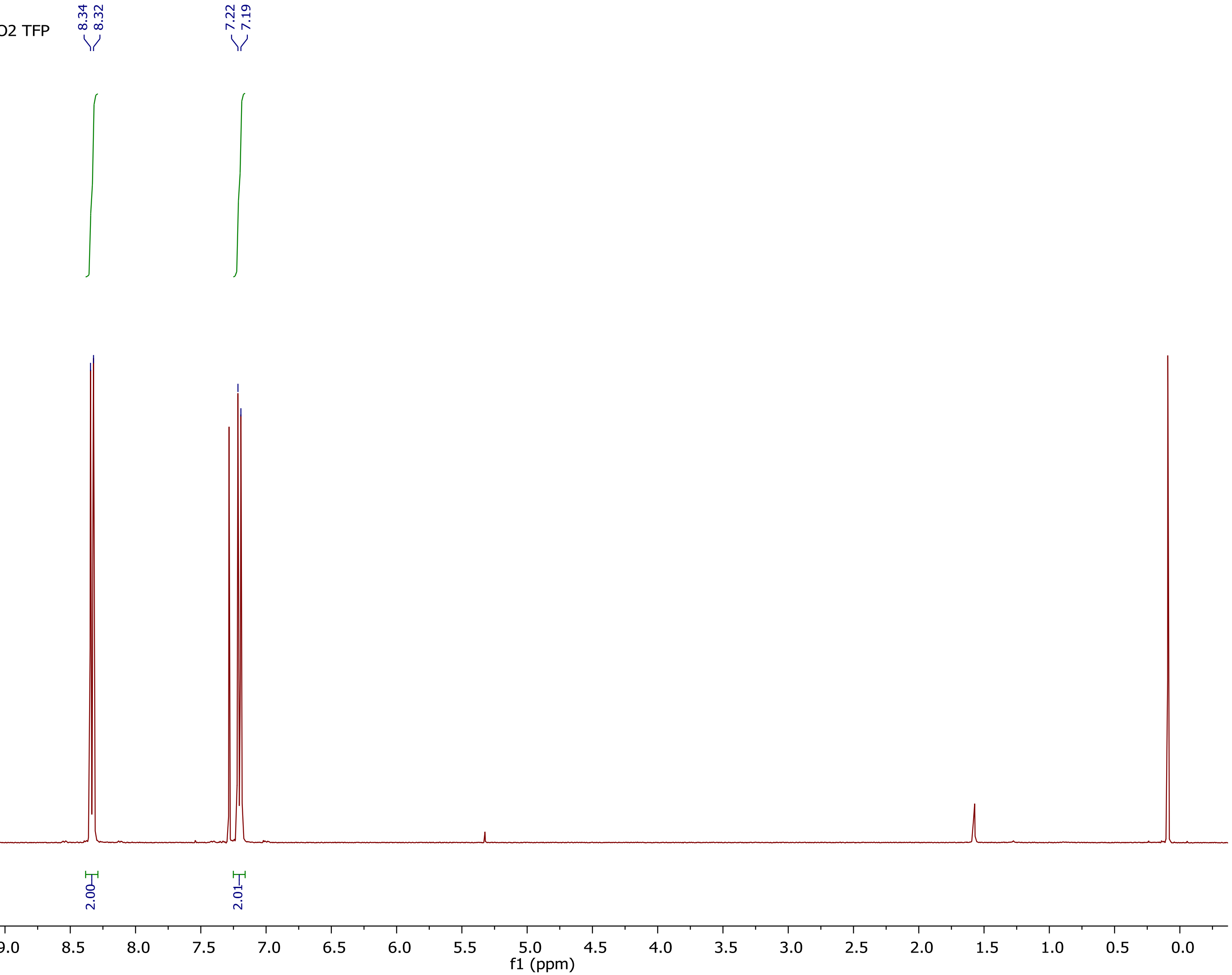

12170052.10.fid
SLC:WDGB:WB 4-NO2 TFP
8.34
8.32
7.22
7.19
2.00
2.01
10.0
9.5
9.0
8.5
8.0
7.5
7.0
6.5
6.0
5.5
5.0
4.5
4.0
3.5
3.0
2.5
2.0
1.5
1.0
0.5
0.0
f1 (ppm)

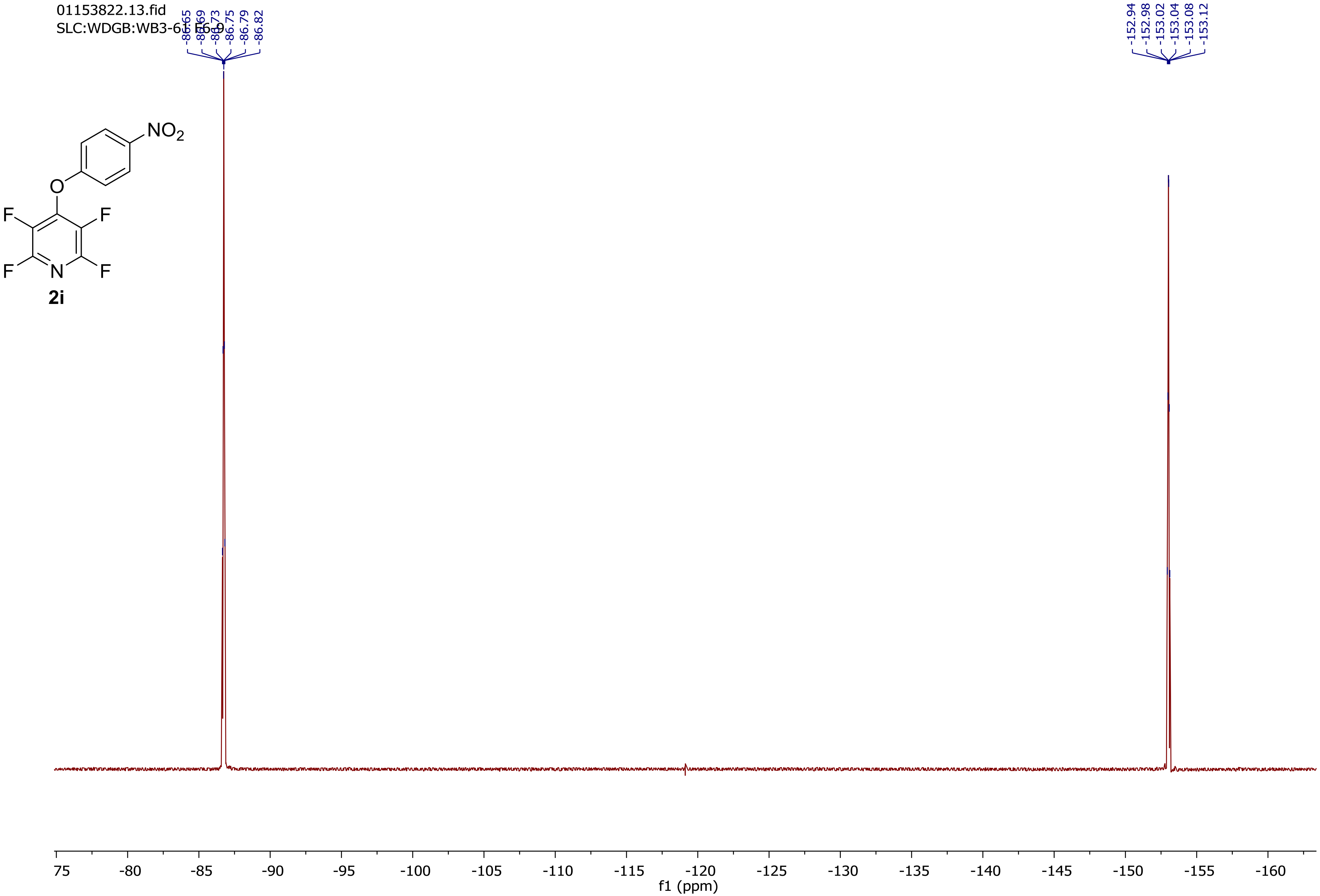

01153822.13.fid
SLC:WDGB:WB3-61 F6-9
-86.65
-86.69
-86.73
-86.75
-86.79
-86.82
-152.94
-152.98
-153.02
-153.04
-153.08
-153.12
NO2
O
F
F
F
N
F
2i
75 -80 -85 -90 -95 -100 -105 -110 -115 -120 -125 -130 -135 -140 -145 -150 -155 -160
f1 (ppm)

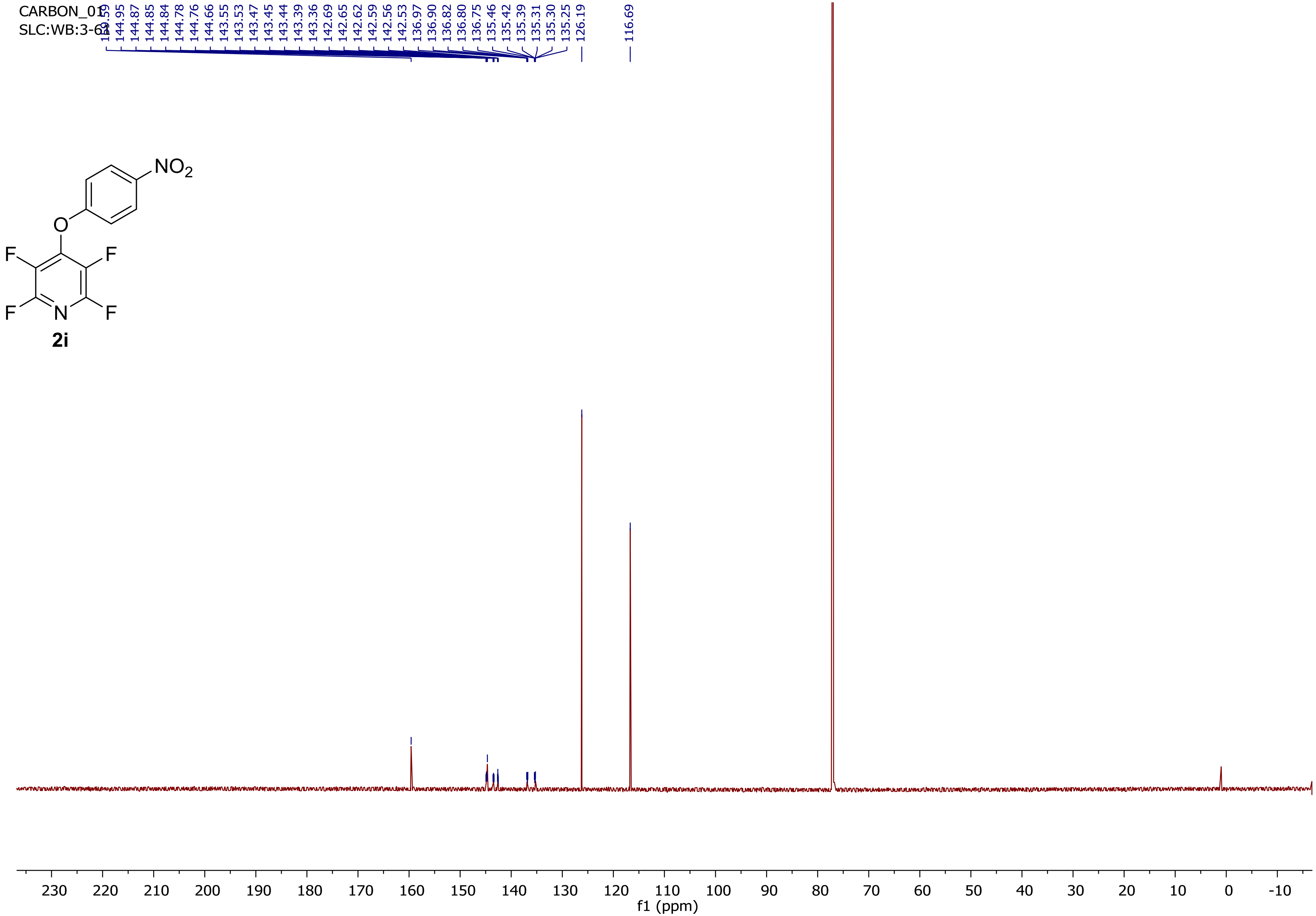
CARBON_01
SLC:WB:3-61
159.59
144.95
144.87
144.85
144.84
144.78
144.76
144.66
143.55
143.53
143.47
143.45
143.44
143.39
143.36
142.69
142.65
142.62
142.59
142.56
142.53
136.97
136.90
136.82
136.80
136.75
135.46
135.42
135.39
135.31
135.30
135.25
126.19
116.69
230 220 210 200 190 180 170 160 150 140 130 120 110 100 90 80 70 60 50 40 30 20 10 0 -10
f1 (ppm)
NO2
O
F
F
F
F
N

2i

12171734.10.fid
SLC:WDGB:WB 4OMe TFP

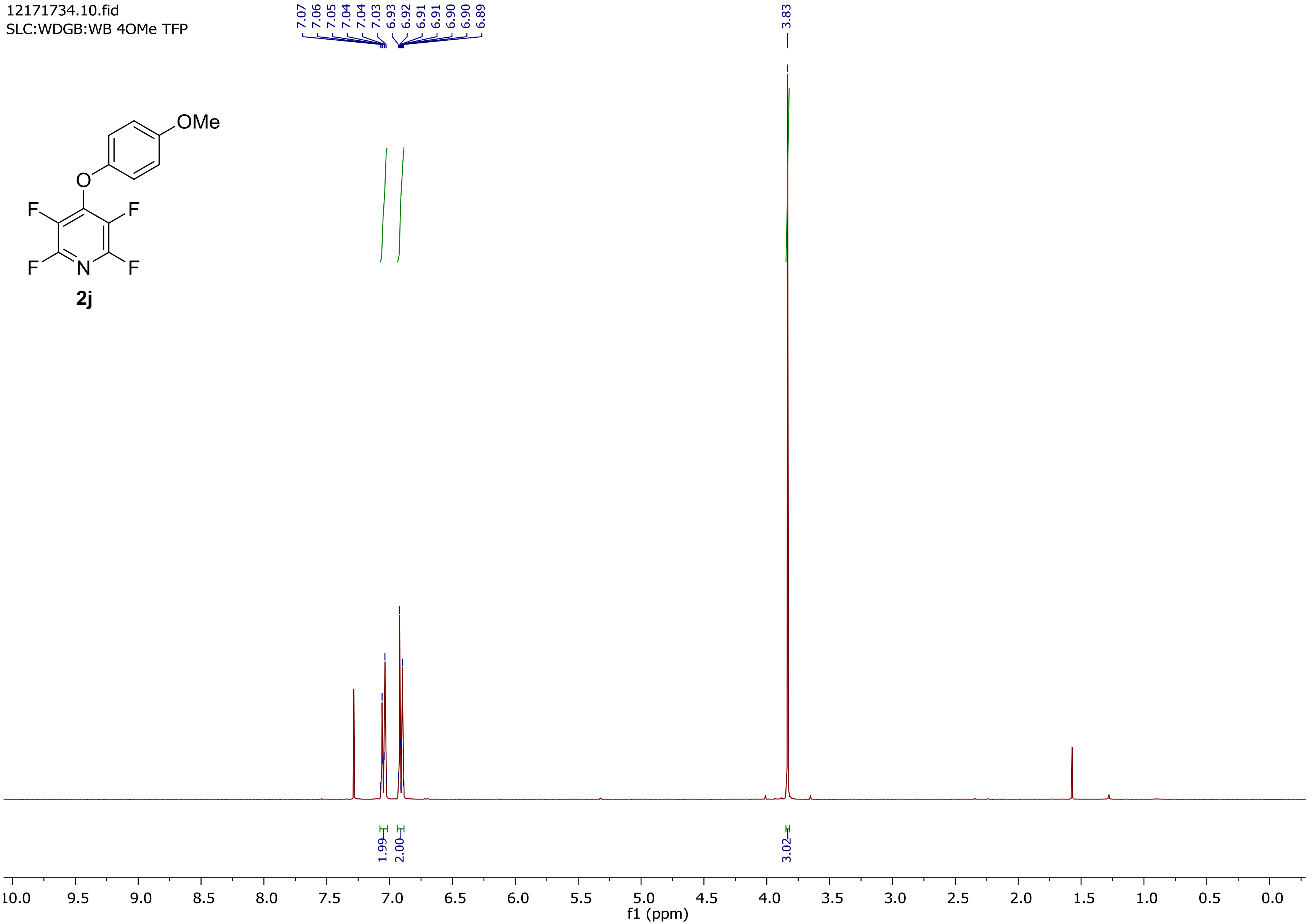

2j

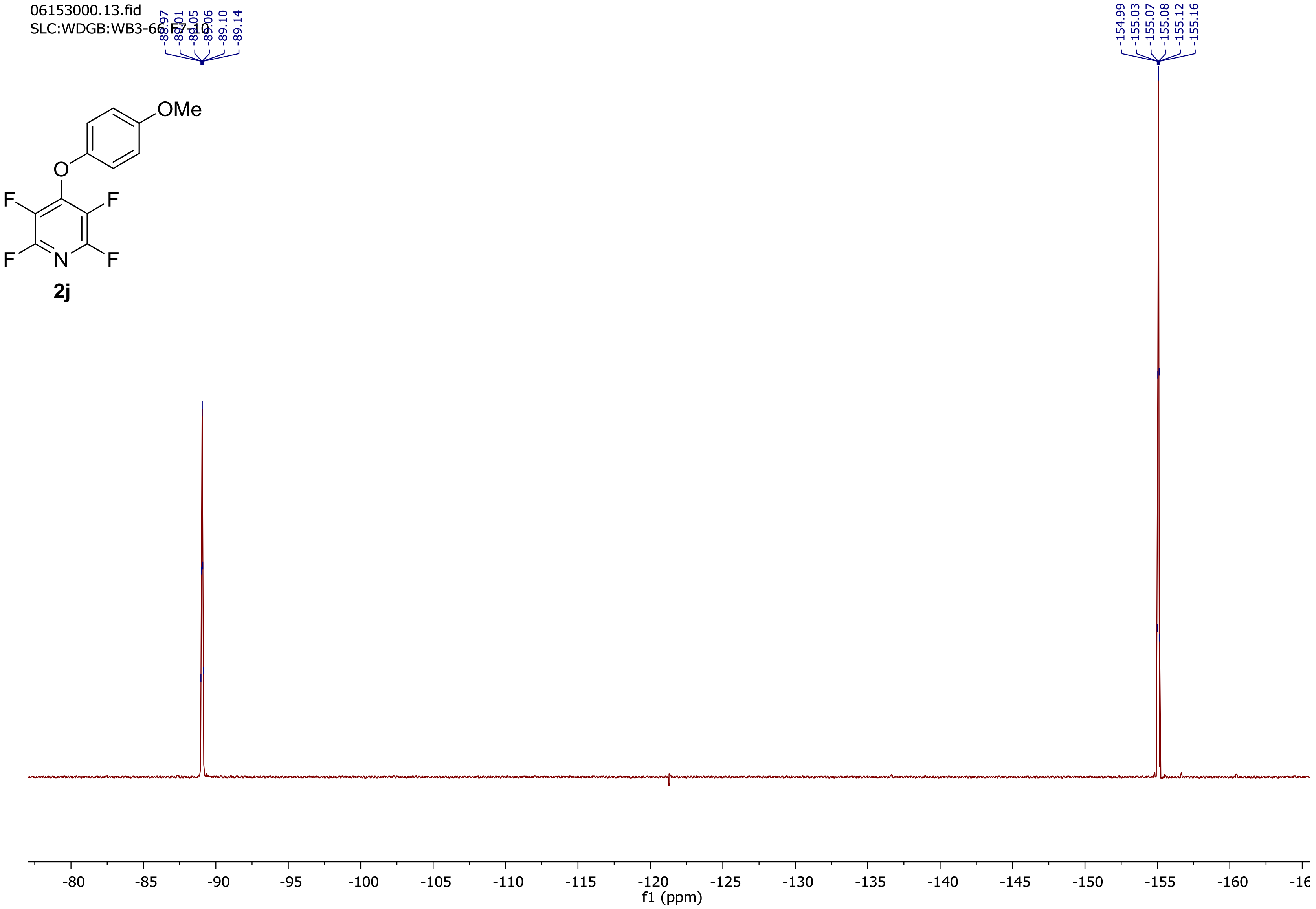
06153000.13.fid
SLC:WDGB:WB3-66
-88.97
-89.01
-89.05
-89.06
-89.10
-89.14
-154.99
-155.03
-155.07
-155.08
-155.12
-155.16
OMe
O
F
F
F
N
F
2j
-80
-85
-90
-95
-100
-105
-110
-115
-120
-125
-130
-135
-140
-145
-150
-155
-160
f1 (ppm)

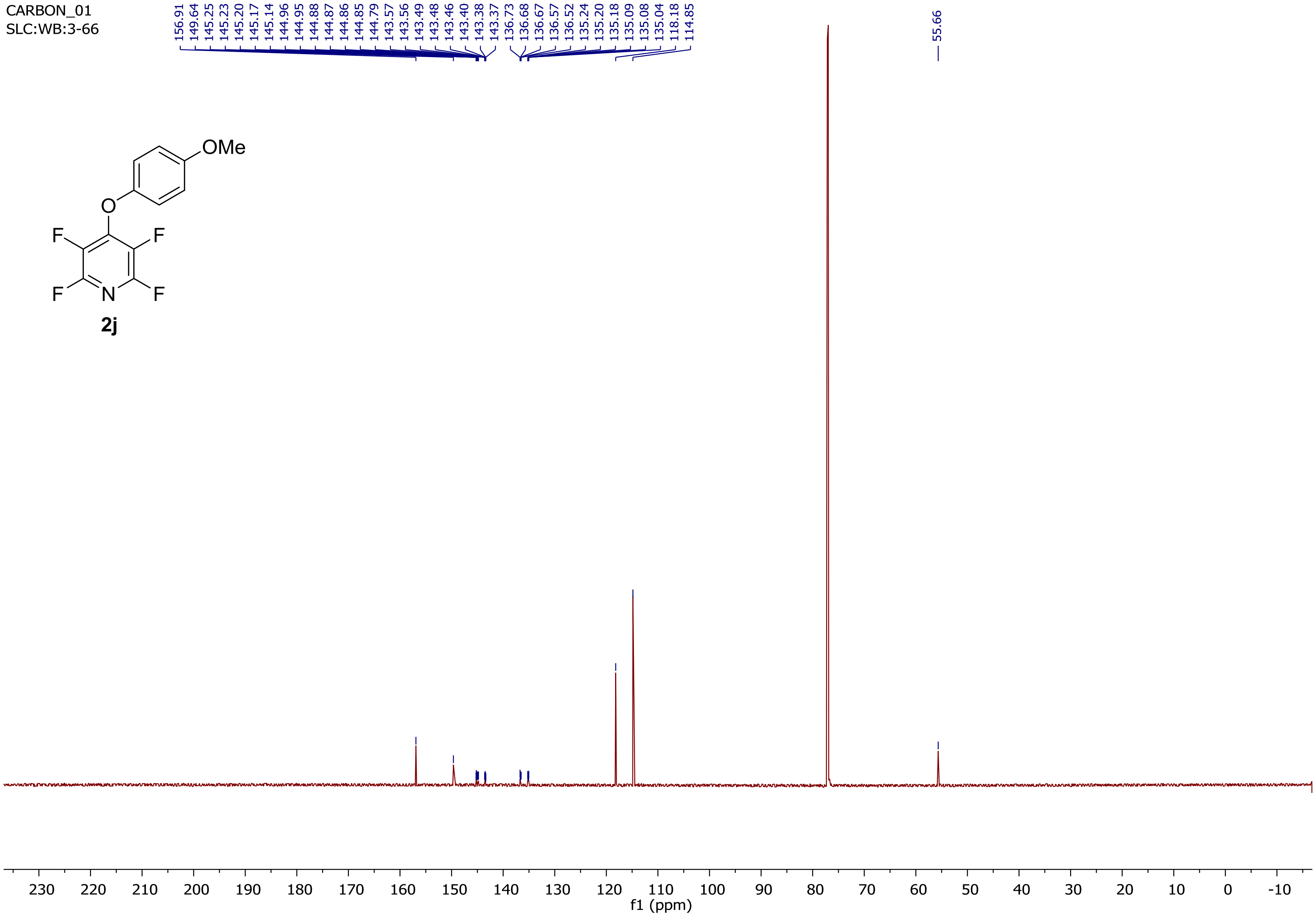
CARBON_01
SLC:WB:3-66
156.91
149.64
145.25
145.23
145.20
145.17
145.14
144.96
144.95
144.88
144.87
144.86
144.85
144.79
143.57
143.56
143.49
143.48
143.46
143.40
143.38
143.37
136.73
136.68
136.67
136.57
136.52
135.24
135.20
135.18
135.09
135.08
135.04
118.18
114.85
55.66
OMe
O
F
F
F
N
F
2j
230
220
210
200
190
180
170
160
150
140
130
120
110
100
90
80
70
60
50
40
30
20
10
0
-10
f1 (ppm)

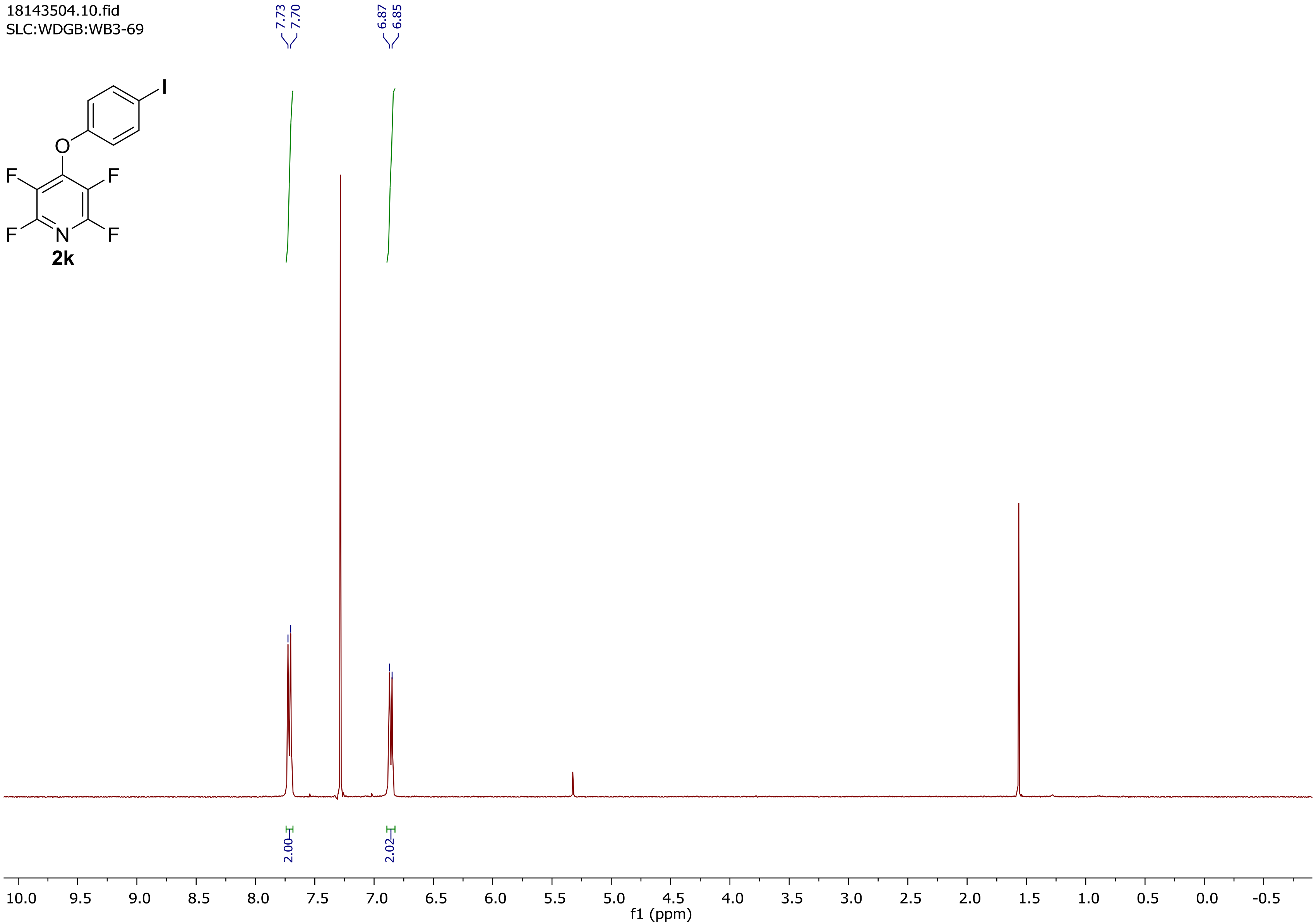
18143504.10.fid
SLC:WDGB:WB3-69
7.73
7.70
6.87
6.85
2k
2.00
2.02
10.0 9.5 9.0 8.5 8.0 7.5 7.0 6.5 6.0 5.5 5.0 4.5 4.0 3.5 3.0 2.5 2.0 1.5 1.0 0.5 0.0 -0.5
f1 (ppm)

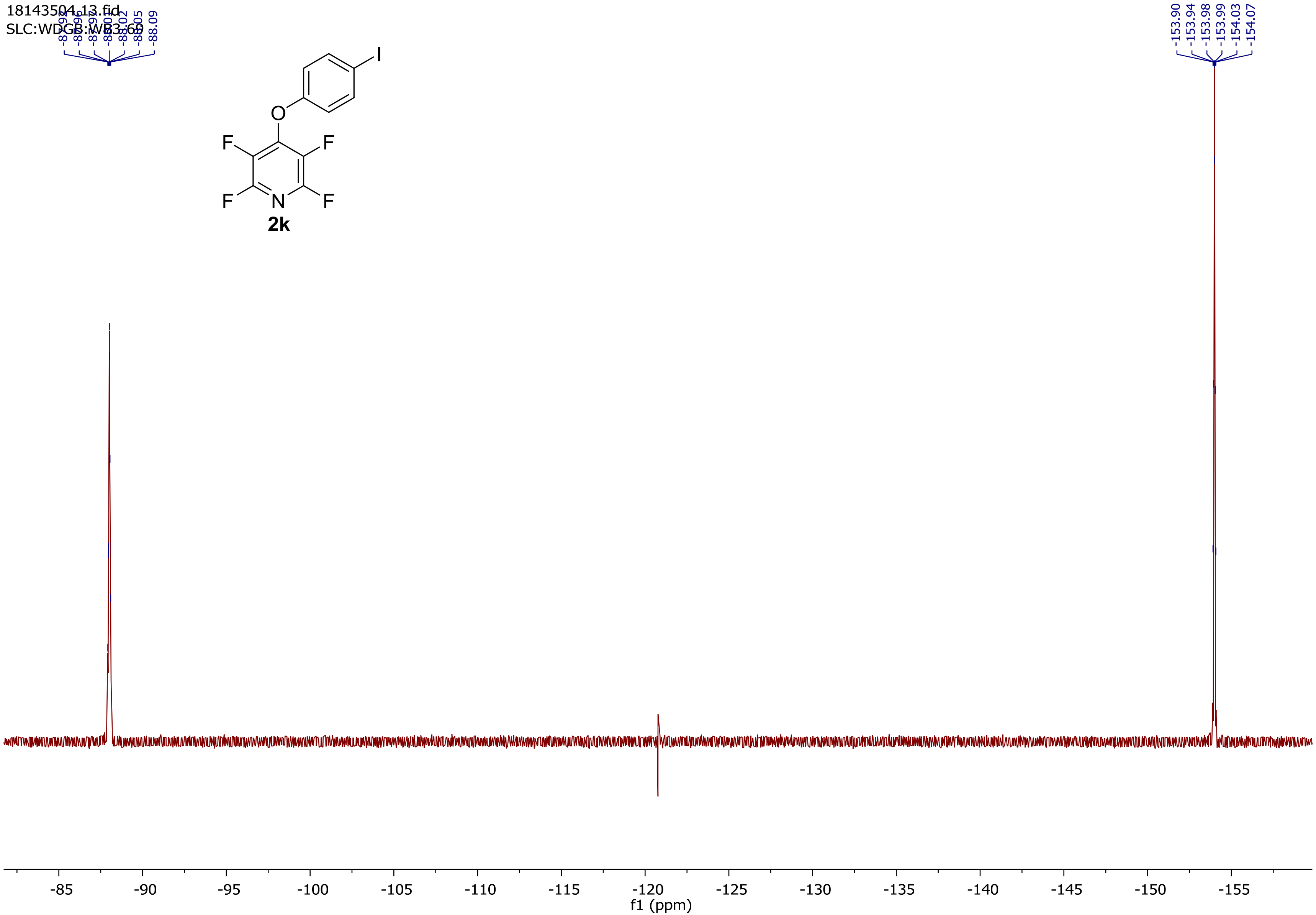
18143504.13.fid
SLC:WDGB:WB3.69
-87.92
-87.96
-87.97
-88.01
-88.02
-88.05
-88.09
-153.90
-153.94
-153.98
-153.99
-154.03
-154.07
F
F
F
F
O
I
N
2k
-85
-90
-95
-100
-105
-110
-115
-120
-125
-130
-135
-140
-145
-150
-155
f1 (ppm)

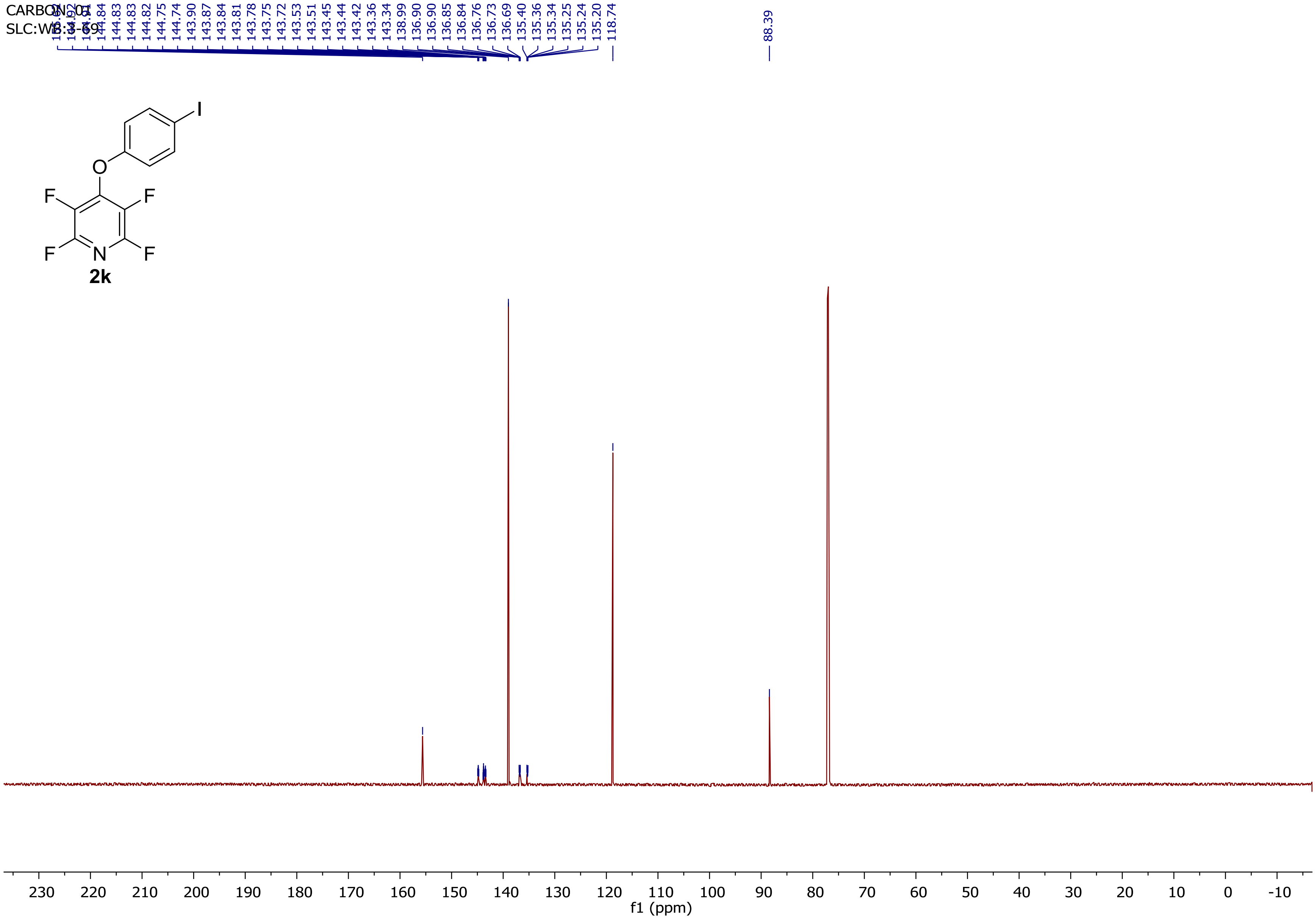
CARBON_01
SLC:WB-3-69
155.62
144.92
144.91
144.84
144.83
144.83
144.82
144.75
144.74
143.90
143.87
143.84
143.81
143.78
143.75
143.72
143.53
143.51
143.45
143.44
143.42
143.36
143.34
138.99
136.90
136.90
136.85
136.84
136.76
136.73
136.69
135.40
135.36
135.34
135.25
135.24
135.20
118.74
88.39
I
O
F
F
F
N
F
2k
230
220
210
200
190
180
170
160
150
140
130
120
110
100
90
80
70
60
50
40
30
20
10
0
-10
f1 (ppm)

18143523.10.fid
SLC:WDGB:WB3-71

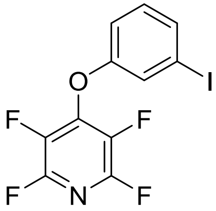

2l

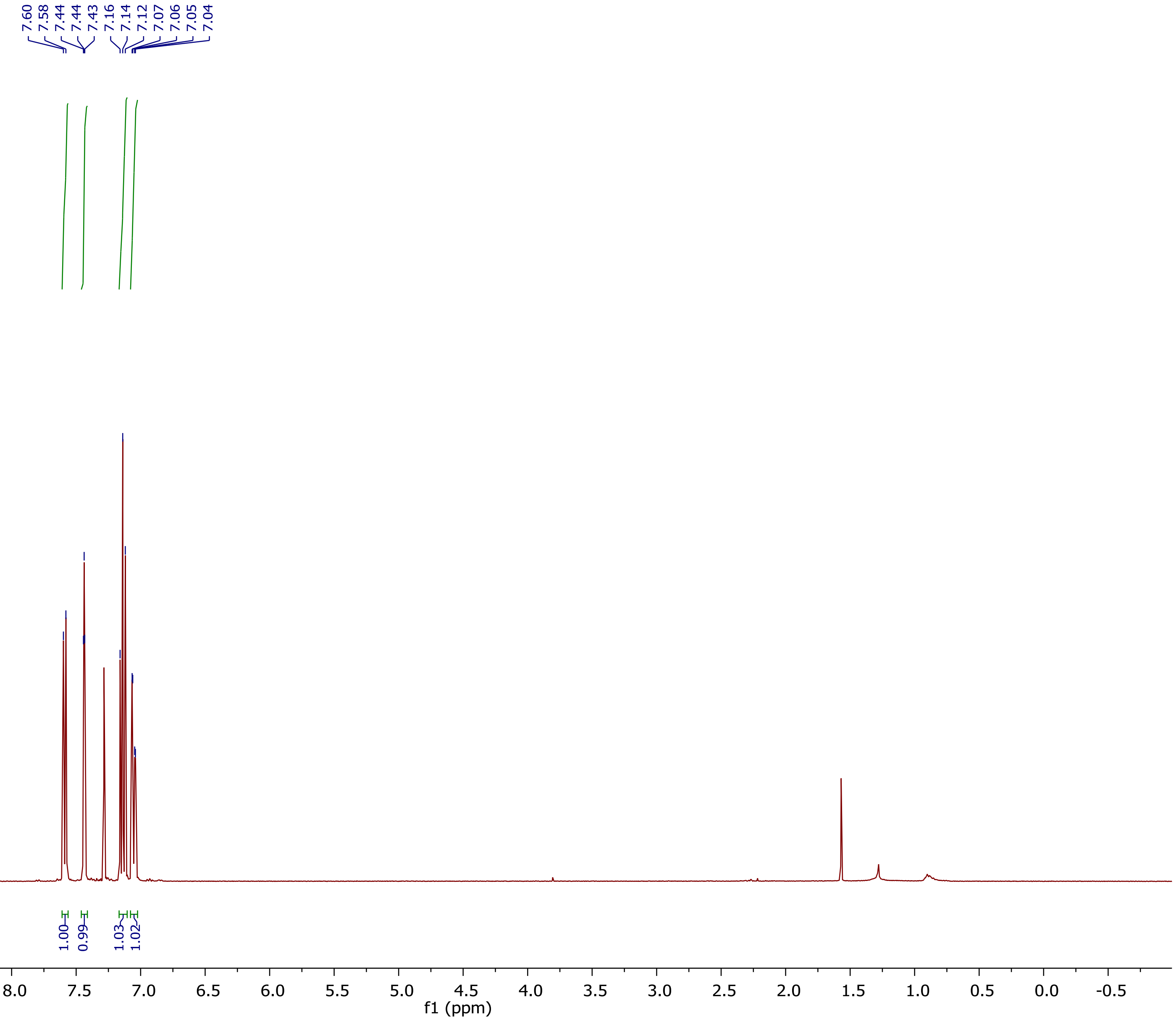

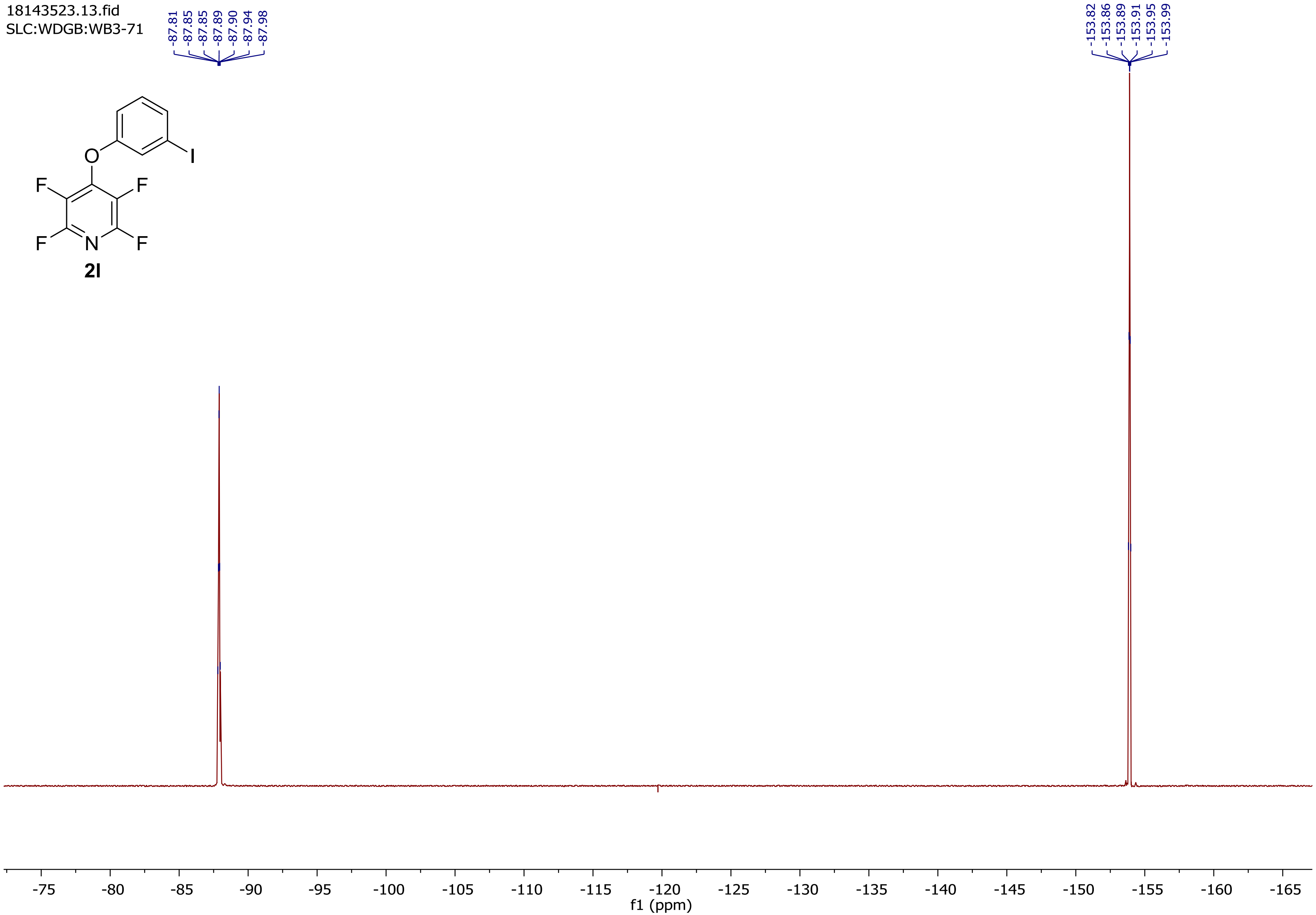
18143523.13.fid
SLC:WDGB:WB3-71
-87.81
-87.85
-87.85
-87.89
-87.90
-87.94
-87.98
-153.82
-153.86
-153.89
-153.91
-153.95
-153.99
O
I
F
F
F
N
F
2l
-75 -80 -85 -90 -95 -100 -105 -110 -115 -120 -125 -130 -135 -140 -145 -150 -155 -160 -165
f1 (ppm)

CARBON_01
SLC:WB:3-71

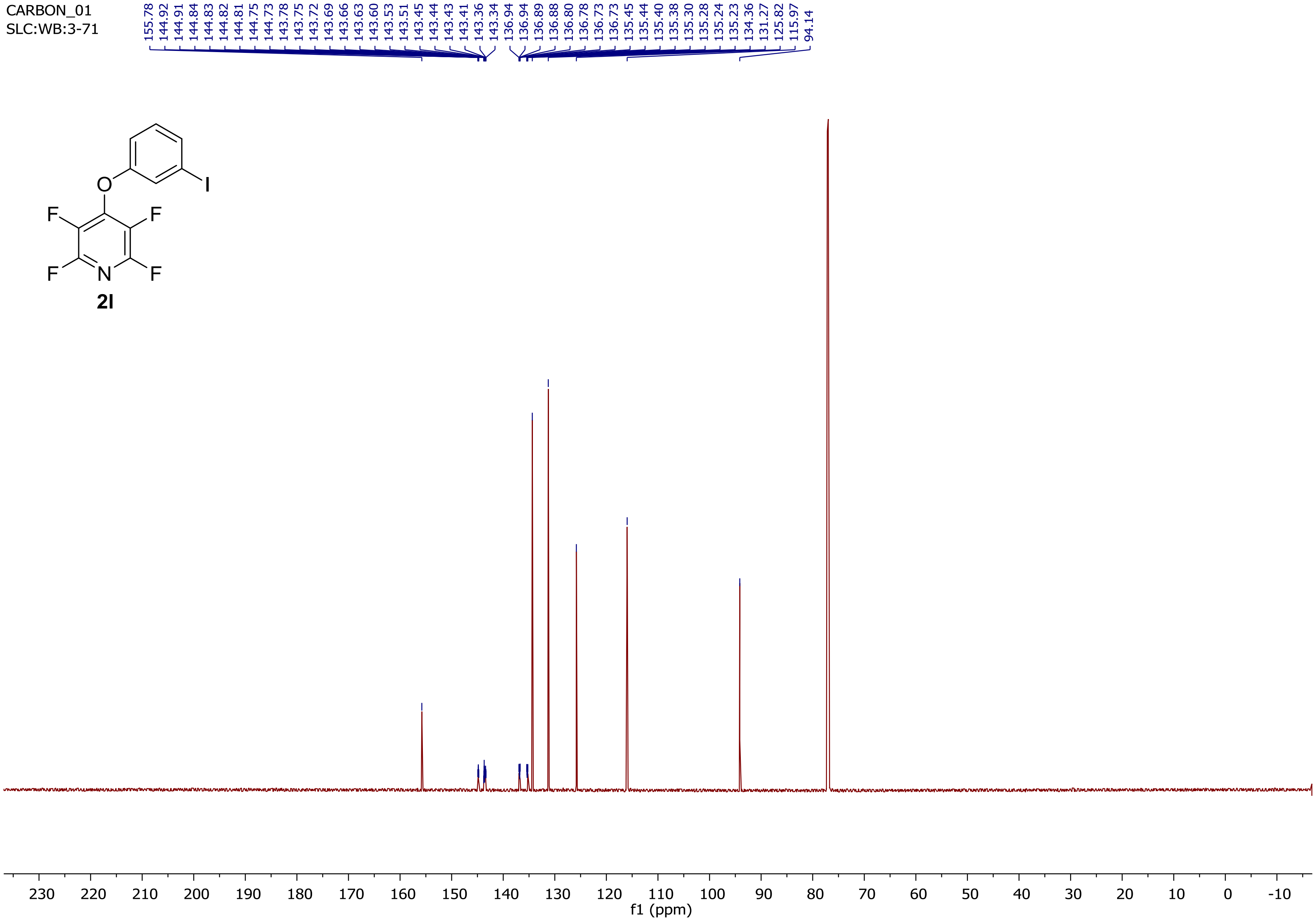

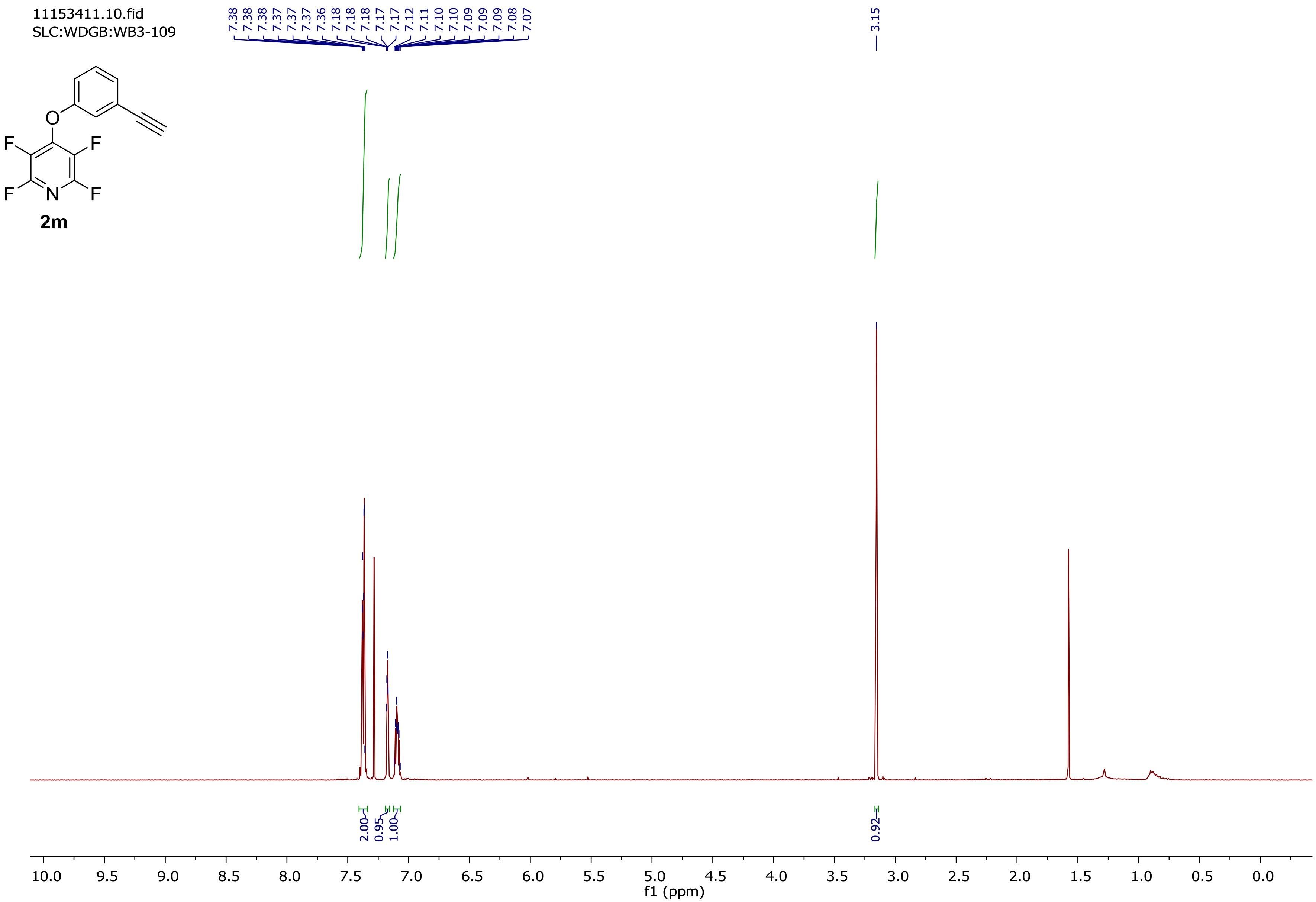
11153411.10.fid
SLC:WDGB:WB3-109
F
F
F
F
O
N
2m
7.38
7.38
7.38
7.37
7.37
7.37
7.36
7.18
7.18
7.17
7.17
7.12
7.11
7.10
7.10
7.09
7.09
7.09
7.08
7.07
3.15
2.00
0.95
1.00
0.92
10.0 9.5 9.0 8.5 8.0 7.5 7.0 6.5 6.0 5.5 5.0 4.5 4.0 3.5 3.0 2.5 2.0 1.5 1.0 0.5 0.0
f1 (ppm)

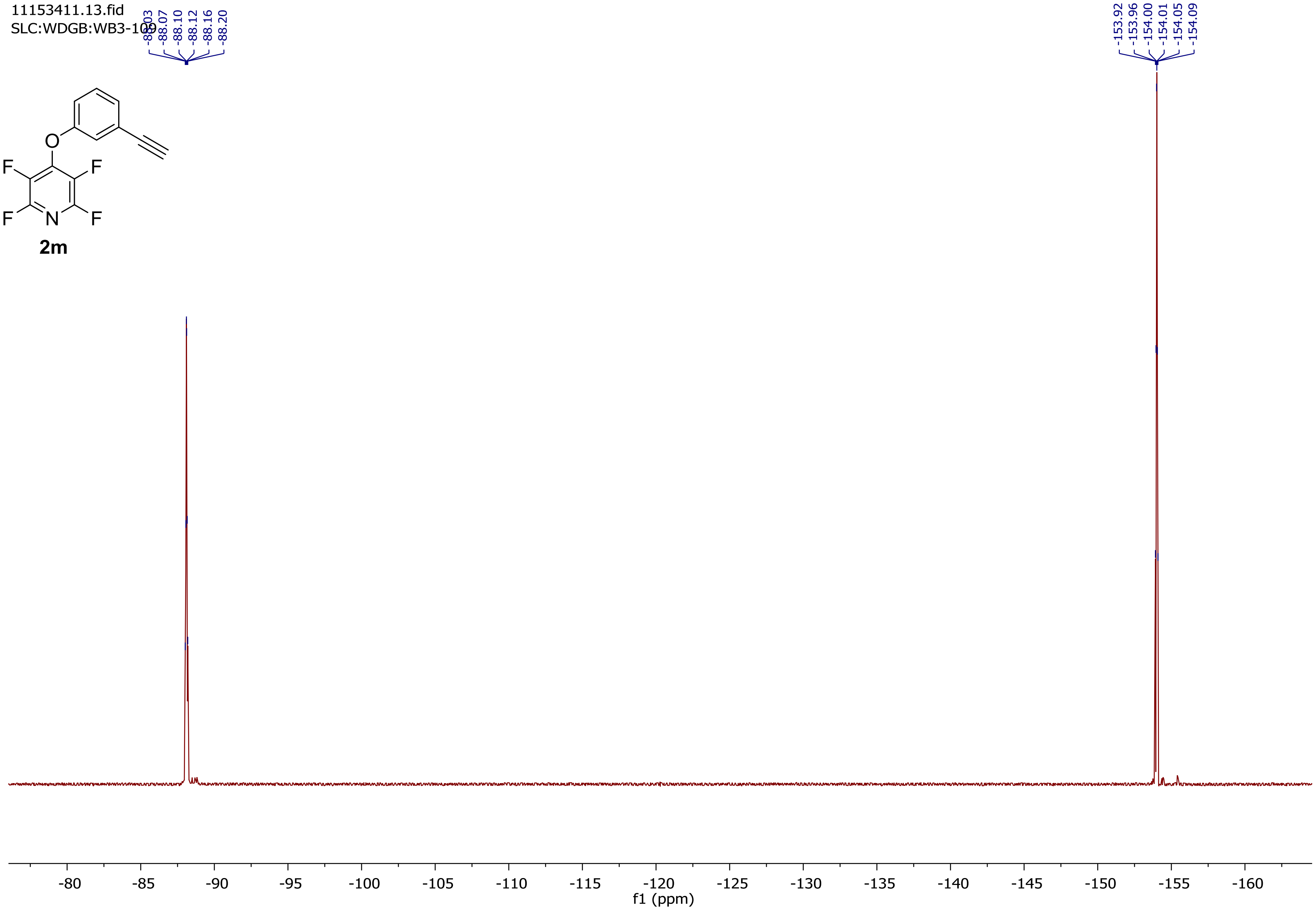
11153411.13.fid
SLC:WDGB:WB3-109
-88.03
-88.07
-88.10
-88.12
-88.16
-88.20
-153.92
-153.96
-154.00
-154.01
-154.05
-154.09
F
F
F
F
N
O
2m
-80
-85
-90
-95
-100
-105
-110
-115
-120
-125
-130
-135
-140
-145
-150
-155
-160
f1 (ppm)

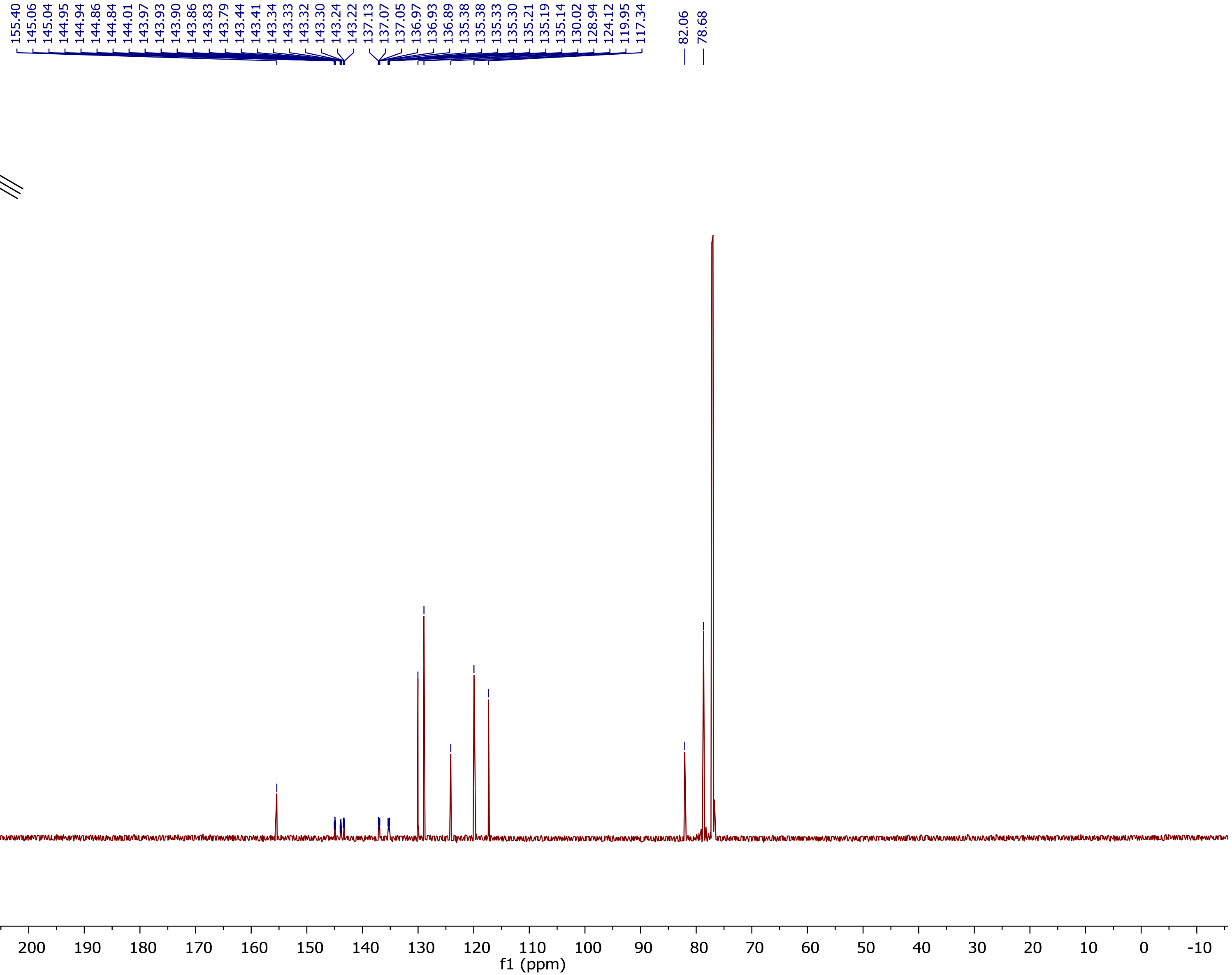
CARBON_01
SLC:WB3-109
155.40
145.06
145.04
144.95
144.94
144.86
144.84
144.01
143.97
143.93
143.90
143.86
143.83
143.79
143.44
143.41
143.34
143.33
143.32
143.30
143.24
143.22
137.13
137.07
137.05
136.97
136.93
136.89
135.38
135.38
135.33
135.30
135.21
135.19
135.14
130.02
128.94
124.12
119.95
117.34
82.06
78.68
230 220 210 200 190 180 170 160 150 140 130 120 110 100 90 80 70 60 50 40 30 20 10 0 -10
f1 (ppm)

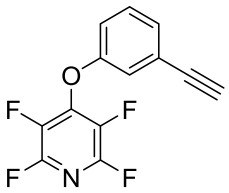
O
F
F
F
F
N

2m

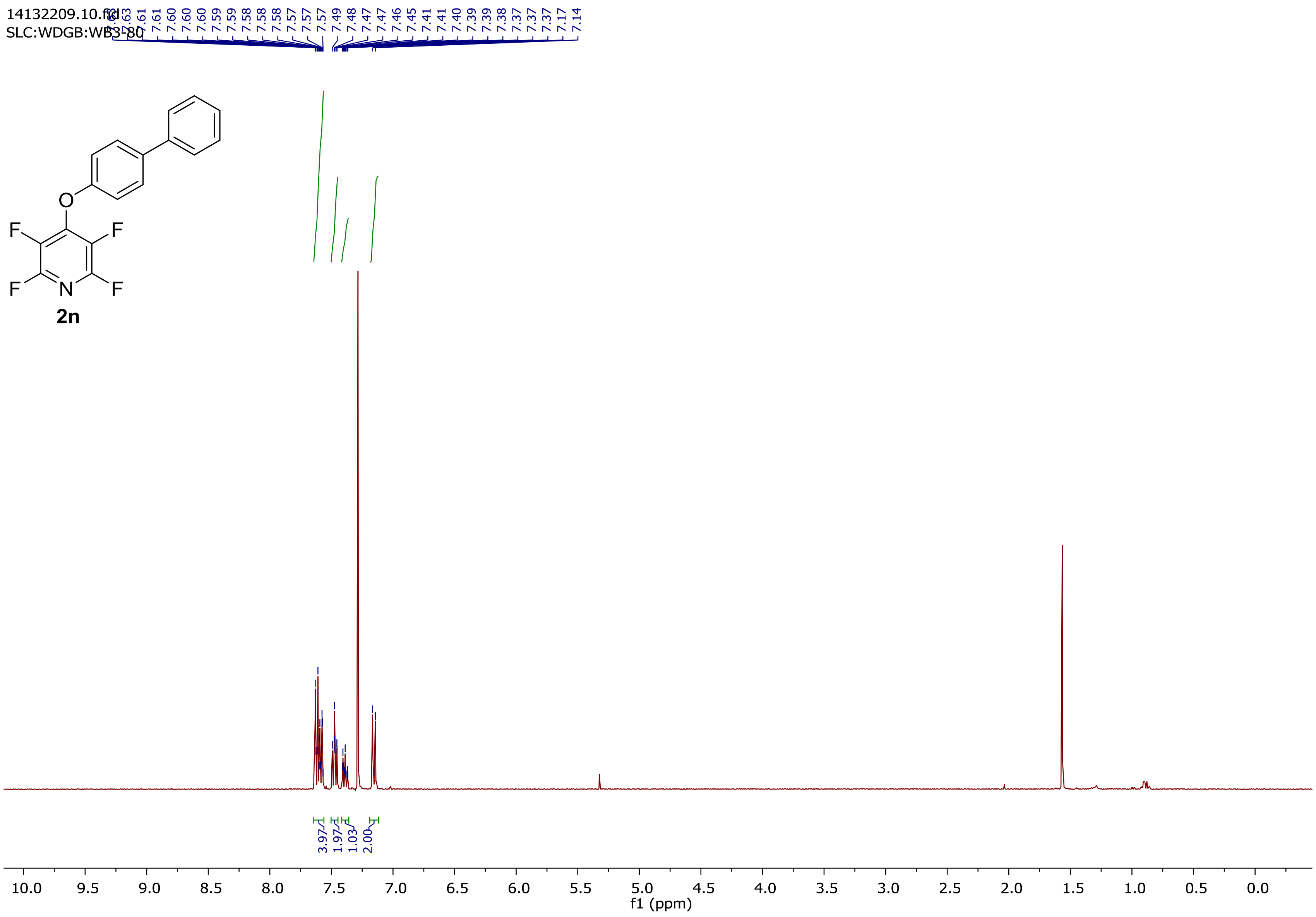
14132209.10.fid
SLC:WDGB:WB3-80
7.63 7.63 7.61 7.61 7.60 7.60 7.60 7.59 7.59 7.58 7.58 7.58 7.57 7.57 7.57 7.49 7.48 7.47 7.47 7.46 7.45 7.41 7.41 7.40 7.39 7.39 7.38 7.37 7.37 7.37 7.17 7.14
O
F
F
F
N
F
2n
3.97
1.97
1.03
2.00
10.0 9.5 9.0 8.5 8.0 7.5 7.0 6.5 6.0 5.5 5.0 4.5 4.0 3.5 3.0 2.5 2.0 1.5 1.0 0.5 0.0
f1 (ppm)

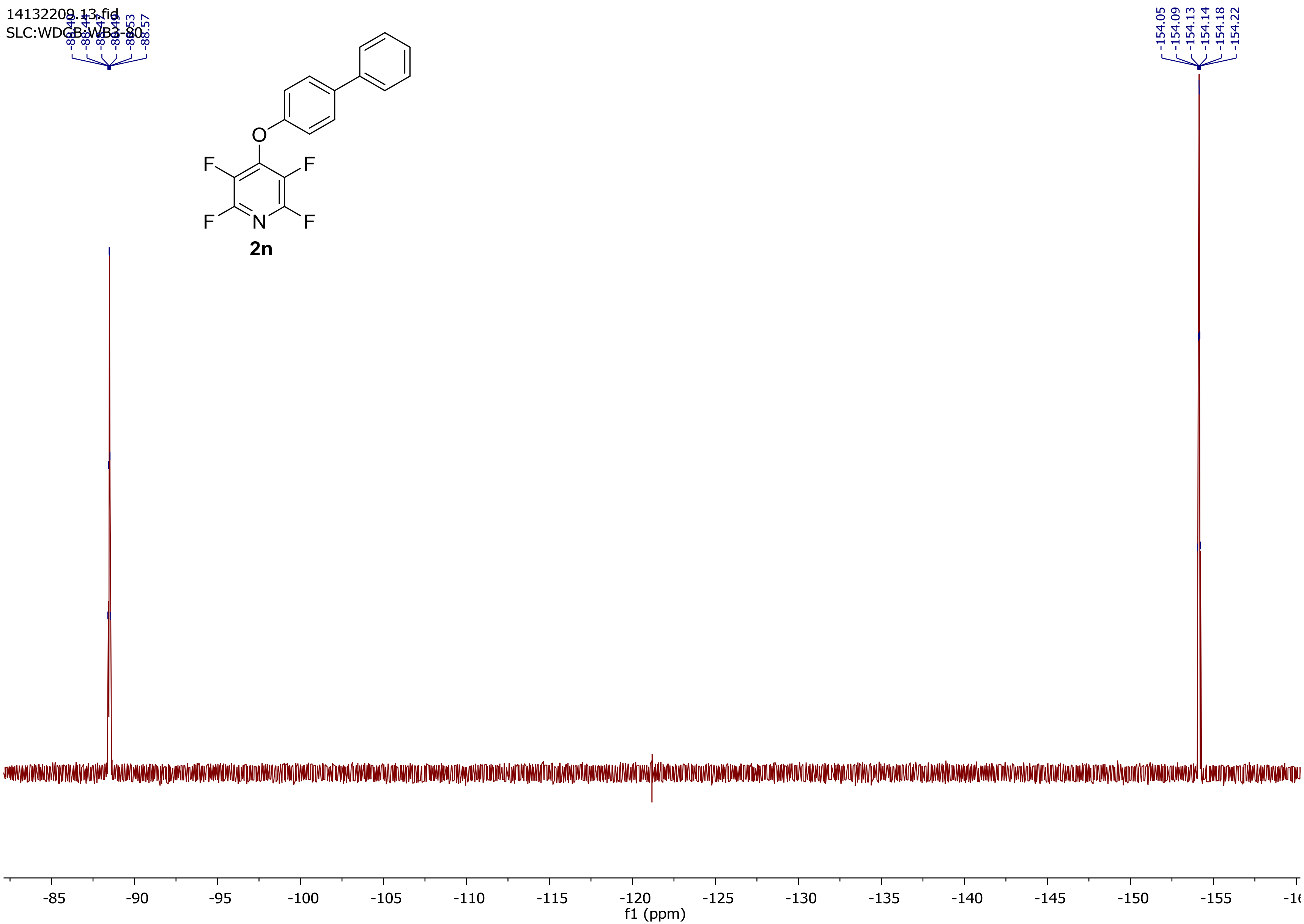

14132209.13.fid
SLC:WDCB:WB3-80.
-88.40
-88.44
-88.47
-88.49
-88.53
-88.57
-154.05
-154.09
-154.13
-154.14
-154.18
-154.22
O
F
F
F
N
F
2n
-85
-90
-95
-100
-105
-110
-115
-120
-125
-130
-135
-140
-145
-150
-155
f1 (ppm)

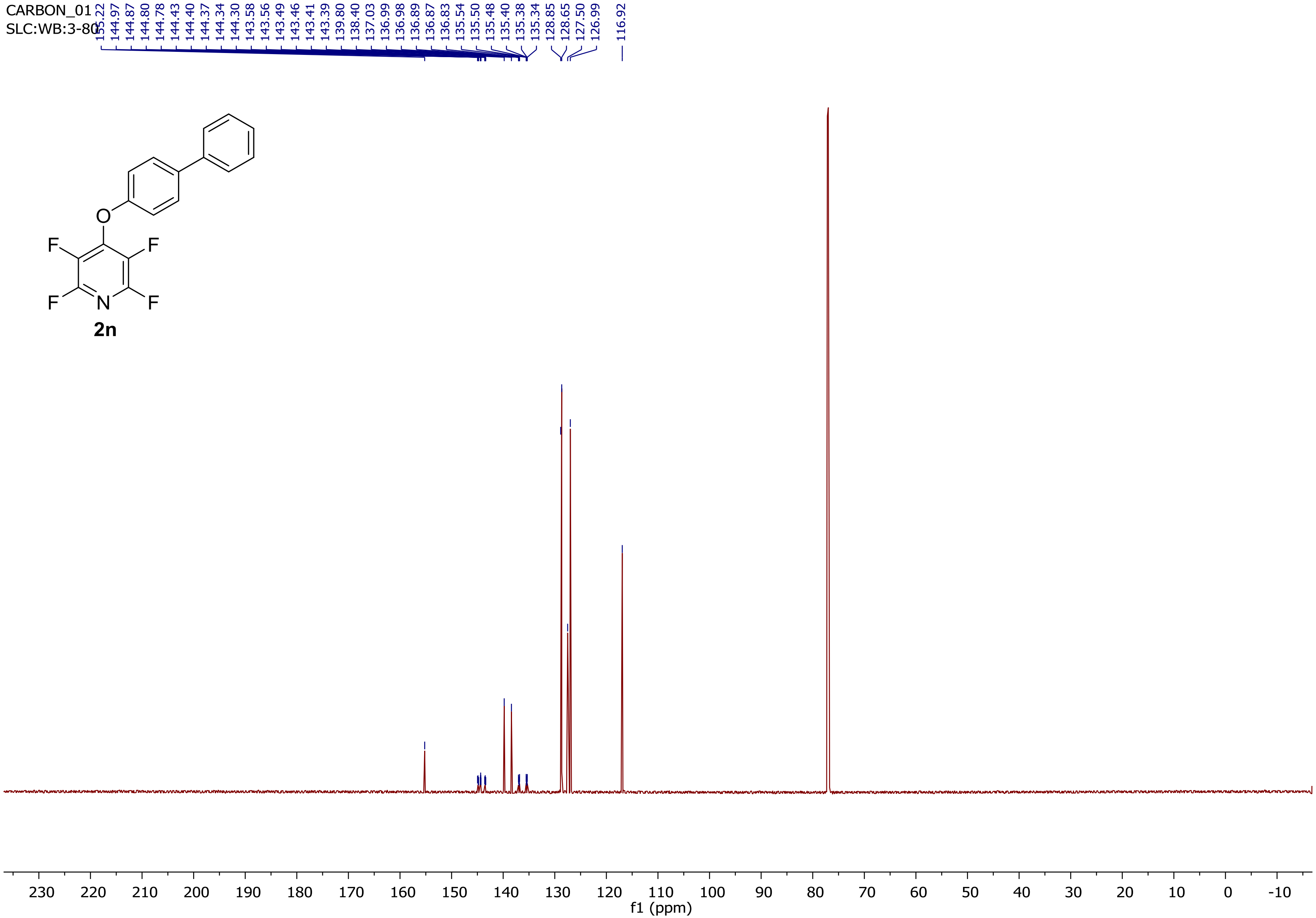
CARBON_01
SLC:WB:3-80
155.22
144.97
144.87
144.80
144.78
144.43
144.40
144.37
144.34
144.30
143.58
143.56
143.49
143.46
143.41
143.39
139.80
138.40
137.03
136.99
136.98
136.89
136.87
136.83
135.54
135.50
135.48
135.40
135.38
135.34
128.85
128.65
127.50
126.99
116.92
O
F
F
F
N
F
2n
230
220
210
200
190
180
170
160
150
140
130
120
110
100
90
80
70
60
50
40
30
20
10
0
-10
f1 (ppm)

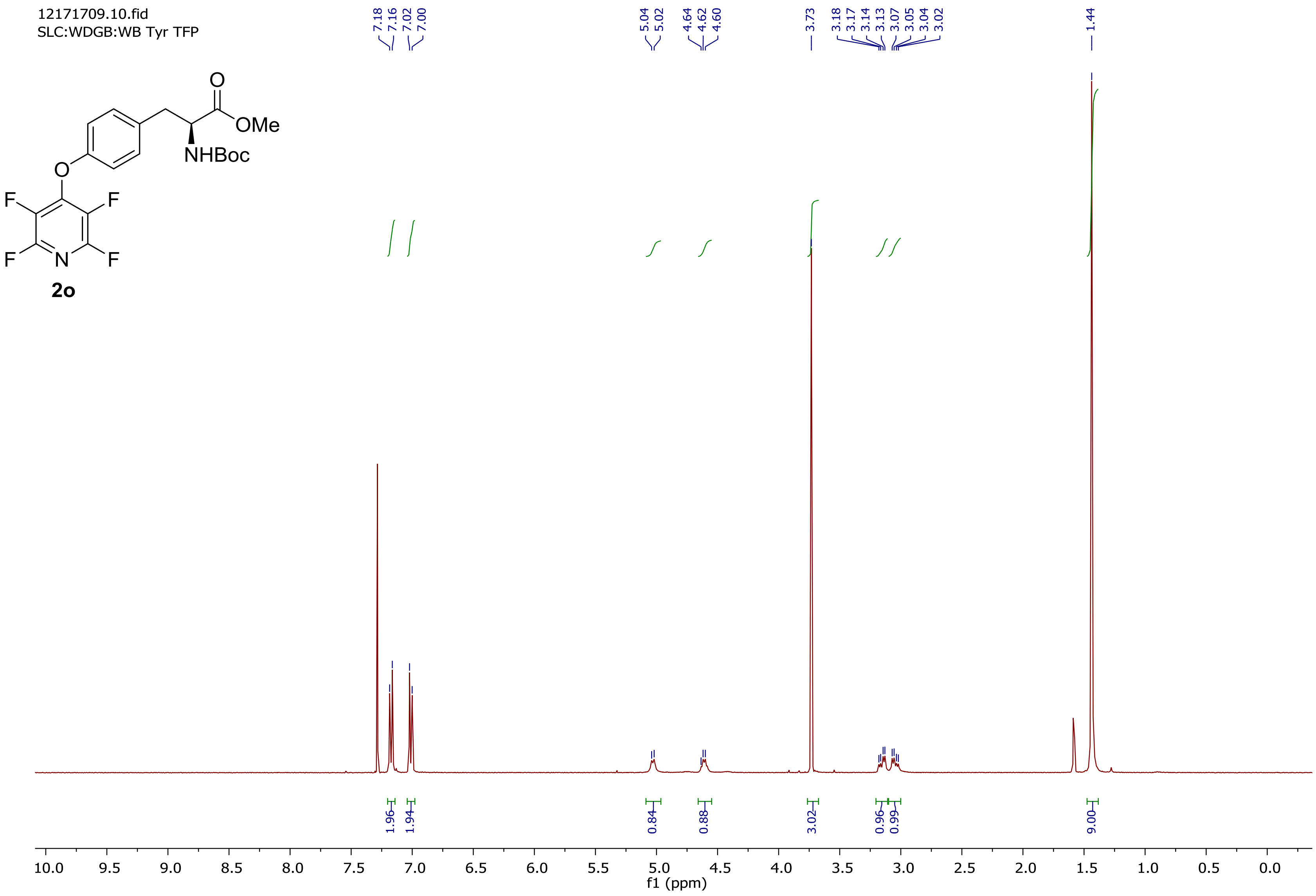
12171709.10.fid
SLC:WDGB:WB Tyr TFP
7.18
7.16
7.02
7.00
5.04
5.02
4.64
4.62
4.60
3.73
3.18
3.17
3.14
3.13
3.07
3.05
3.04
3.02
1.44
O
OMe
NHBoc
O
F
F
F
N
F
2o
1.96
1.94
0.84
0.88
3.02
0.96
0.99
9.00
10.0 9.5 9.0 8.5 8.0 7.5 7.0 6.5 6.0 5.5 5.0 4.5 4.0 3.5 3.0 2.5 2.0 1.5 1.0 0.5 0.0
f1 (ppm)

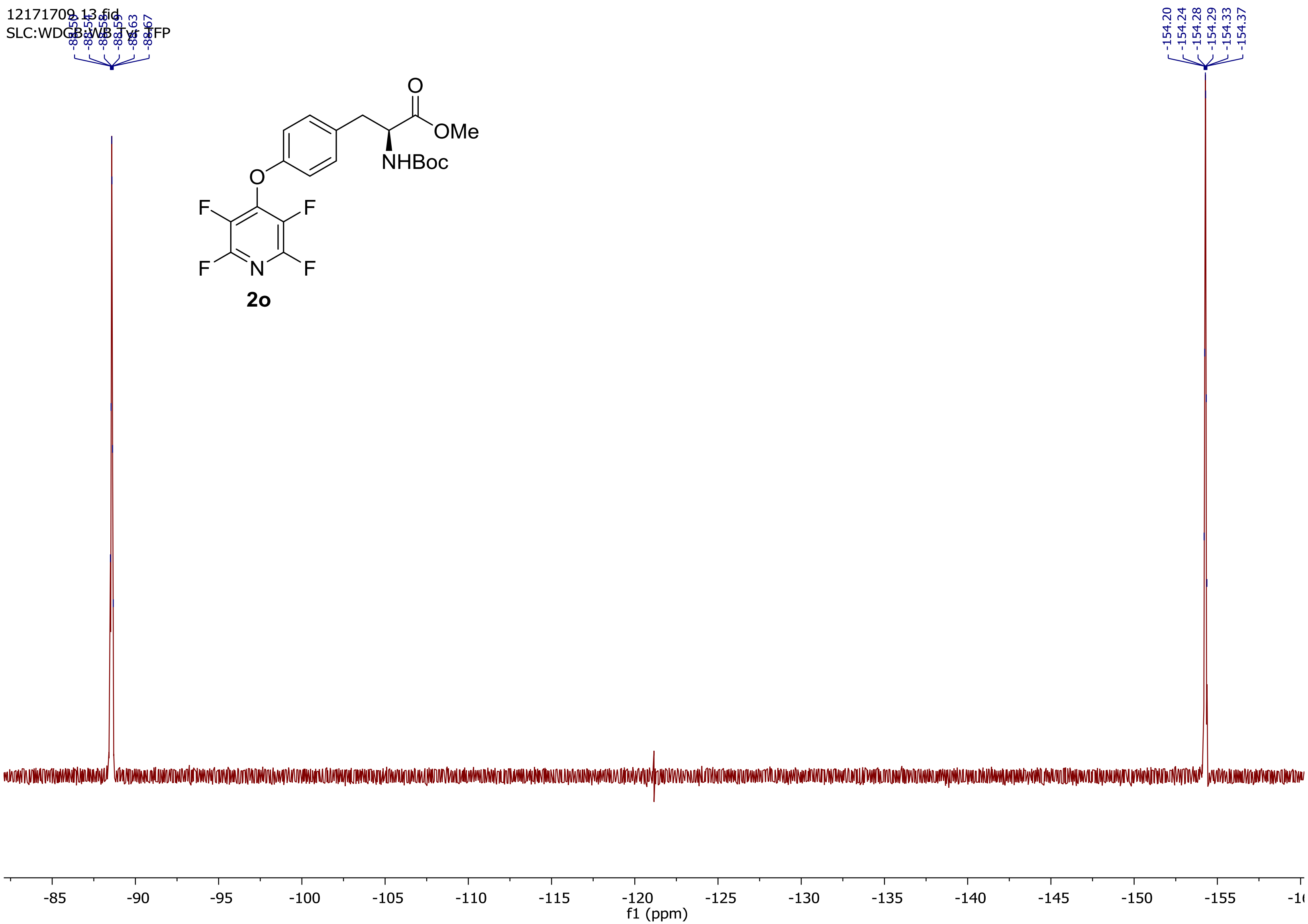
12171709.13.fid
SLC:WDGB:WB Tyr TFP
-88.50
-88.54
-88.58
-88.59
-88.63
-88.67
-154.20
-154.24
-154.28
-154.29
-154.33
-154.37
O
OMe
NHBoc
O
F
F
F
N
F
2o
-85
-90
-95
-100
-105
-110
-115
-120
-125
-130
-135
-140
-145
-150
-155
f1 (ppm)

CARBON_01
SLC:WB:3-41

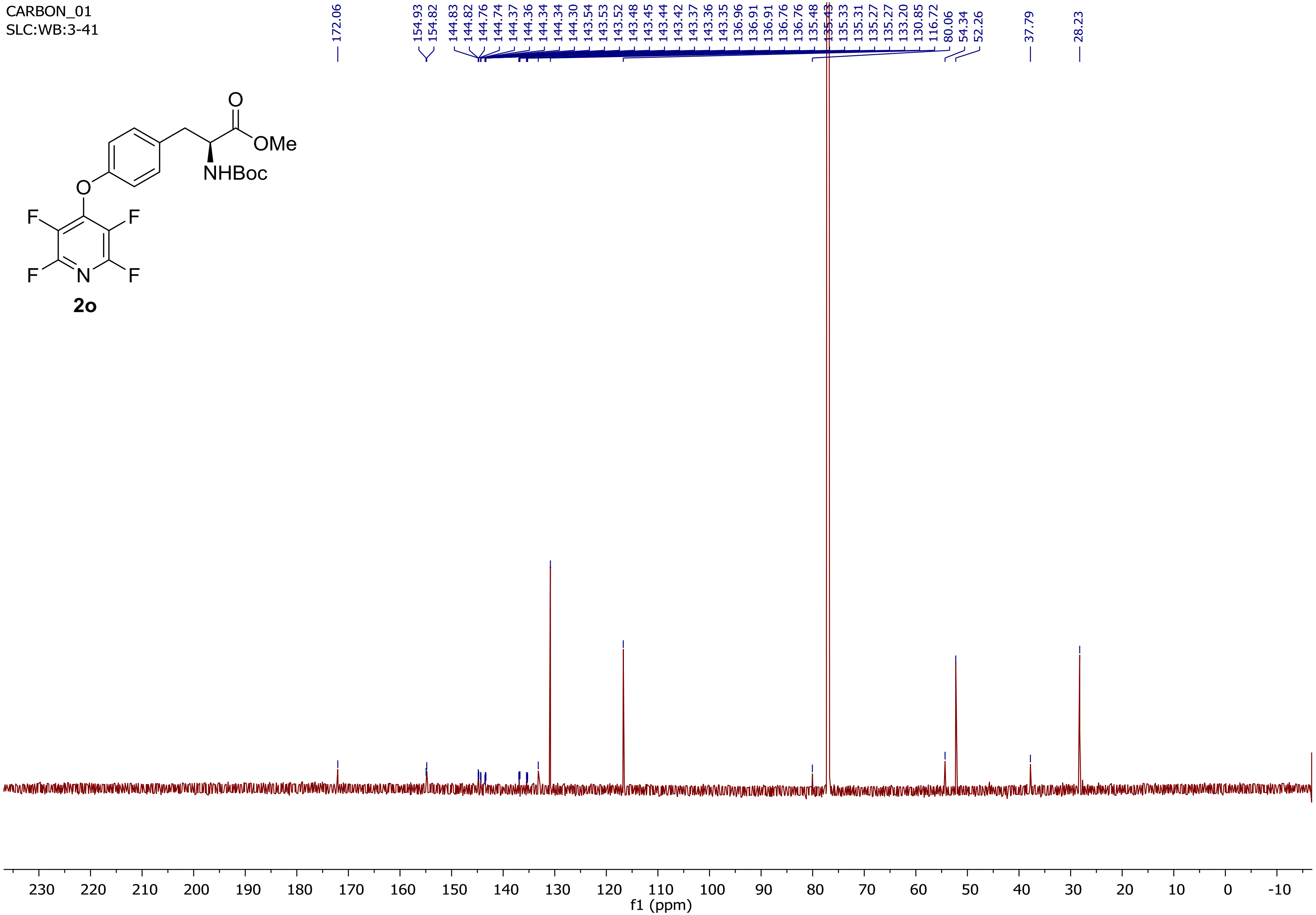

2o

PROTON_01
SLC:WB3-122

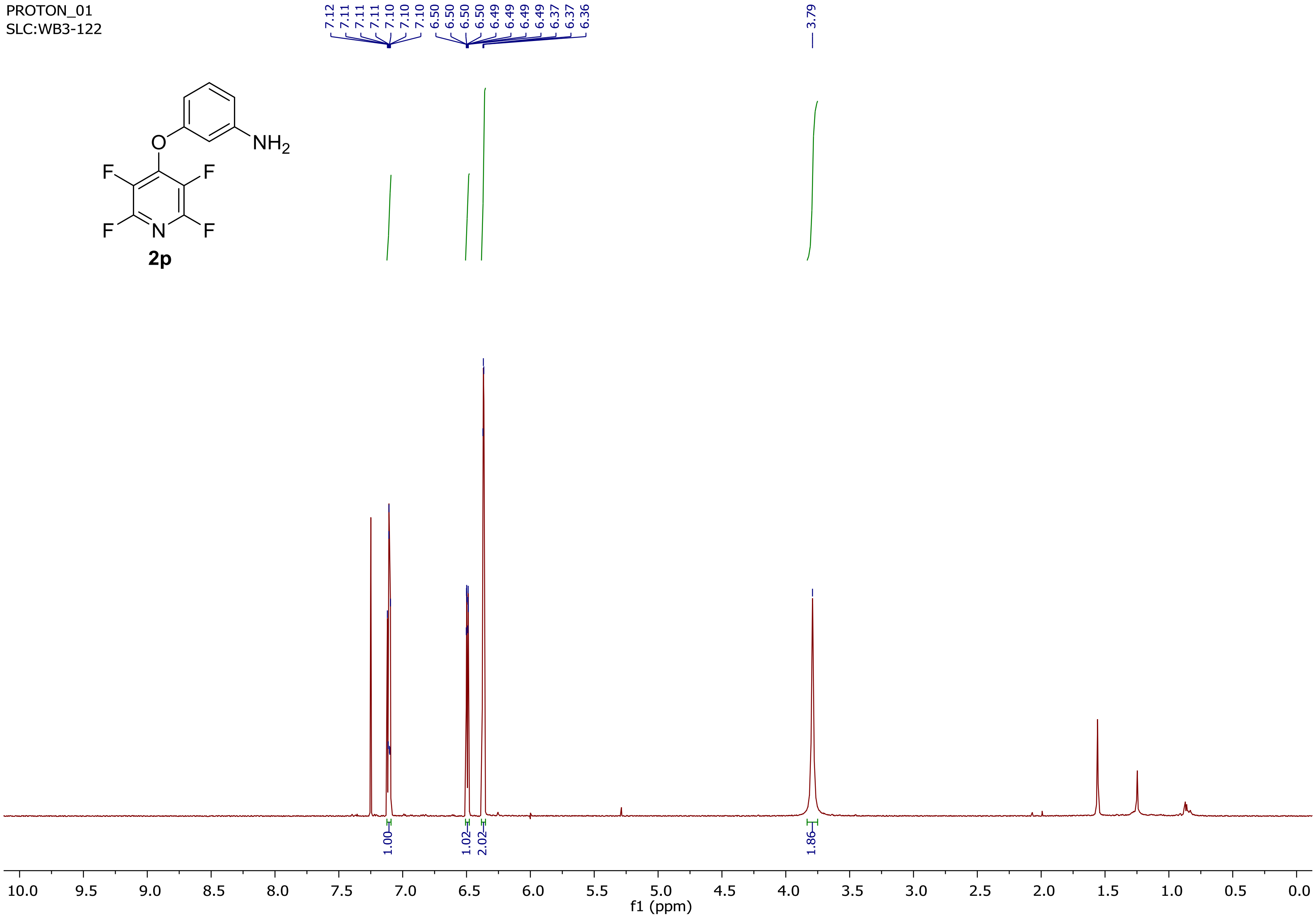

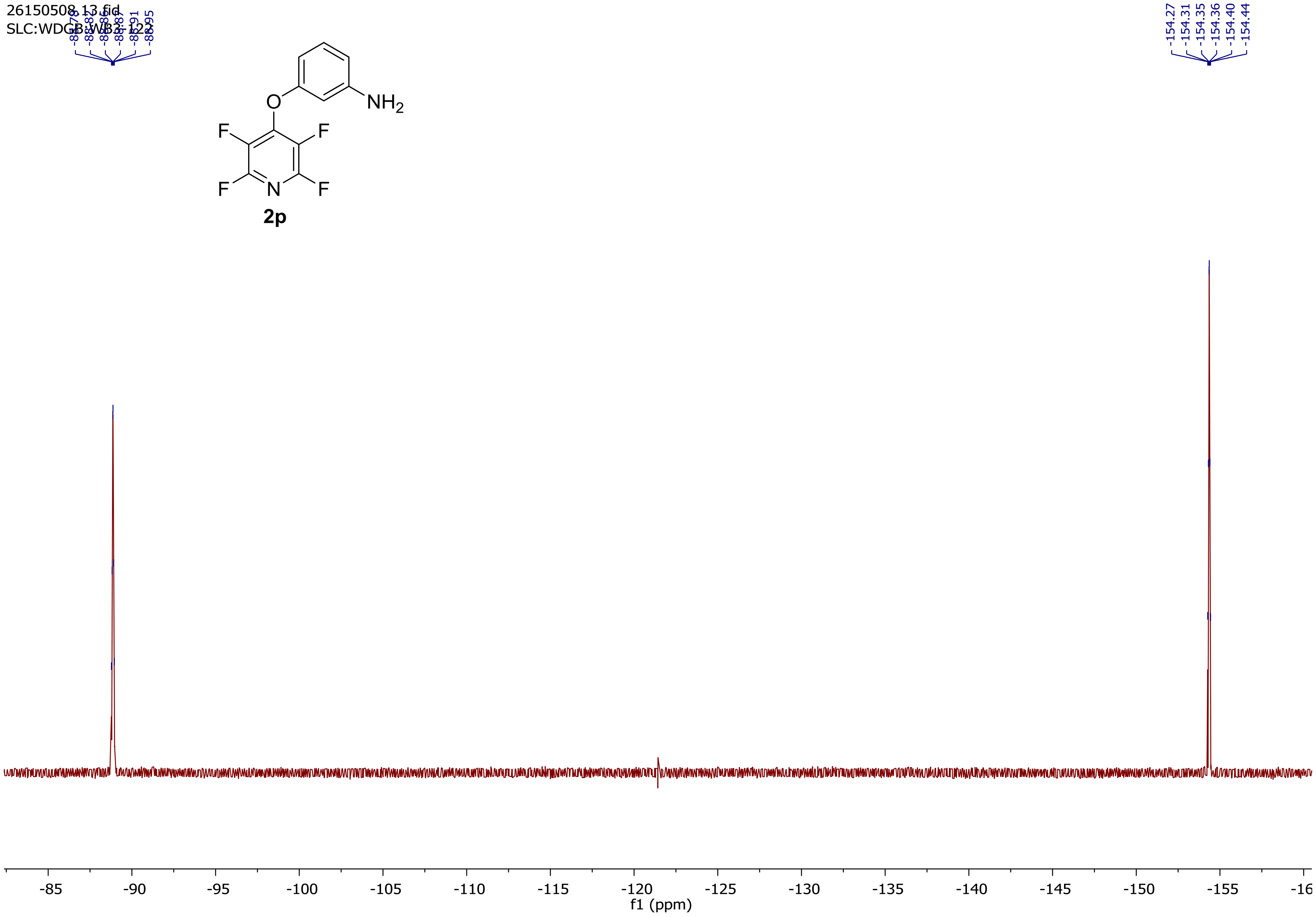
26150508.13.fid
SLC:WDGB-WB3-122
-88.78
-88.82
-88.86
-88.87
-88.91
-88.95
-154.27
-154.31
-154.35
-154.36
-154.40
-154.44
O
NH2
F
F
F
N
F
2p
-85
-90
-95
-100
-105
-110
-115
-120
-125
-130
-135
-140
-145
-150
-155
-16
f1 (ppm)

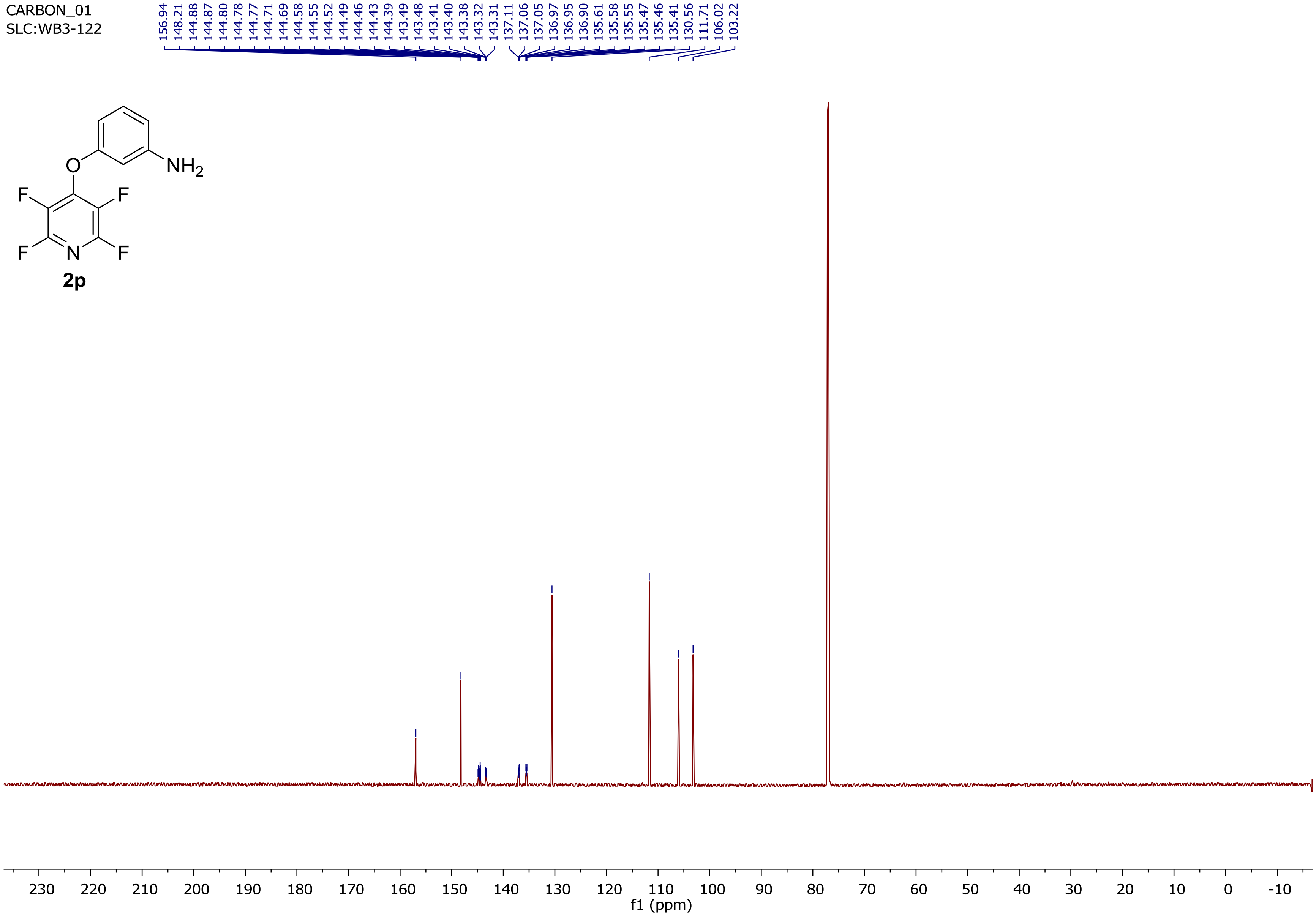
CARBON_01
SLC:WB3-122
156.94
148.21
144.88
144.87
144.80
144.78
144.77
144.71
144.69
144.58
144.55
144.52
144.49
144.46
144.43
144.39
143.49
143.48
143.41
143.40
143.38
143.32
143.31
137.11
137.06
137.05
136.97
136.95
136.90
135.61
135.58
135.55
135.47
135.46
135.41
130.56
111.71
106.02
103.22
O
NH2
F
F
F
N
F
2p
230 220 210 200 190 180 170 160 150 140 130 120 110 100 90 80 70 60 50 40 30 20 10 0 -10
f1 (ppm)

18143541.10.fid
SLC:WDGB:WB3-75

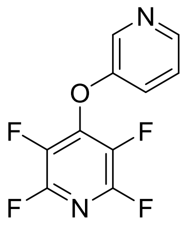

2q

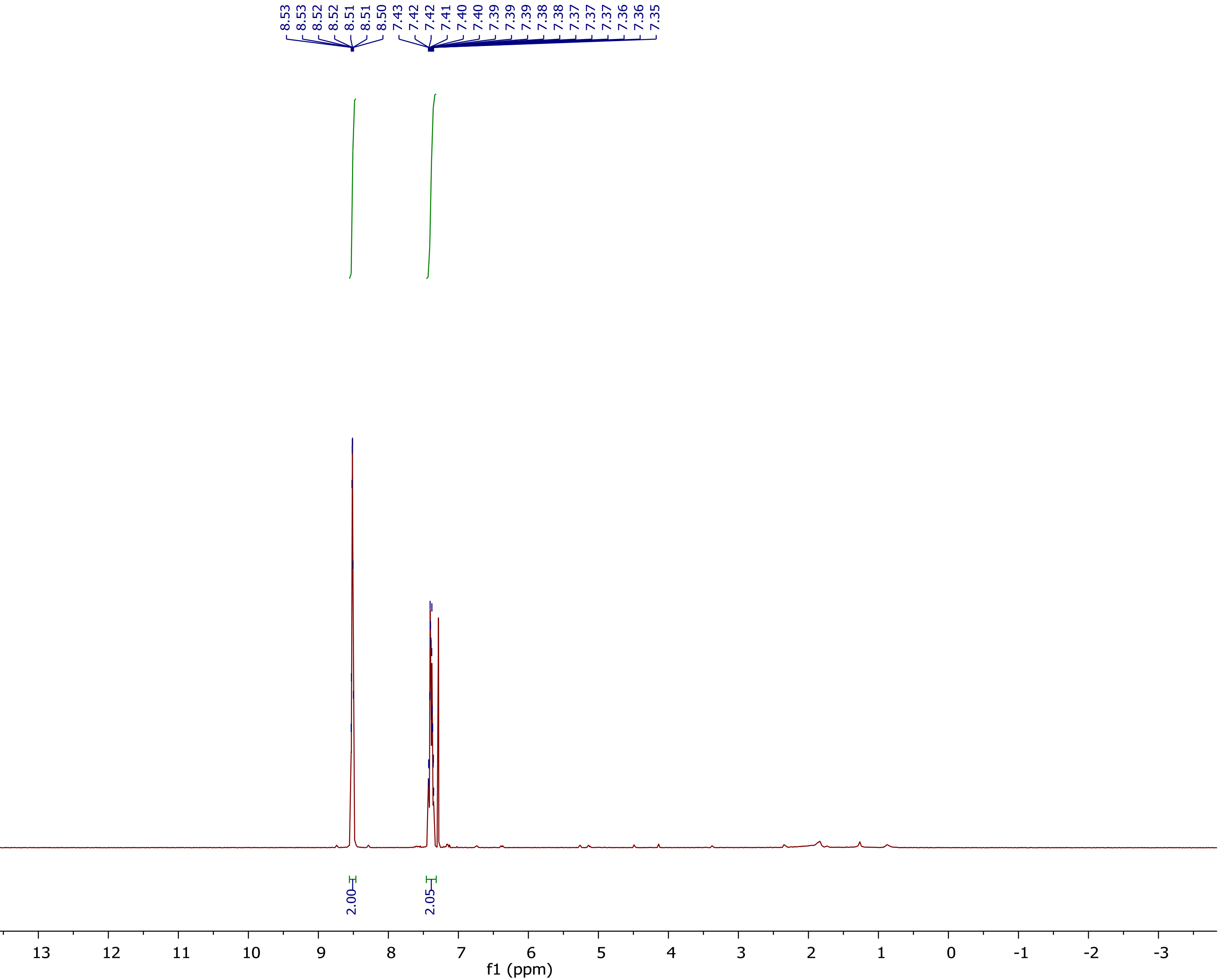

18143541.13.fid
SLC:WDGB:WB3-75

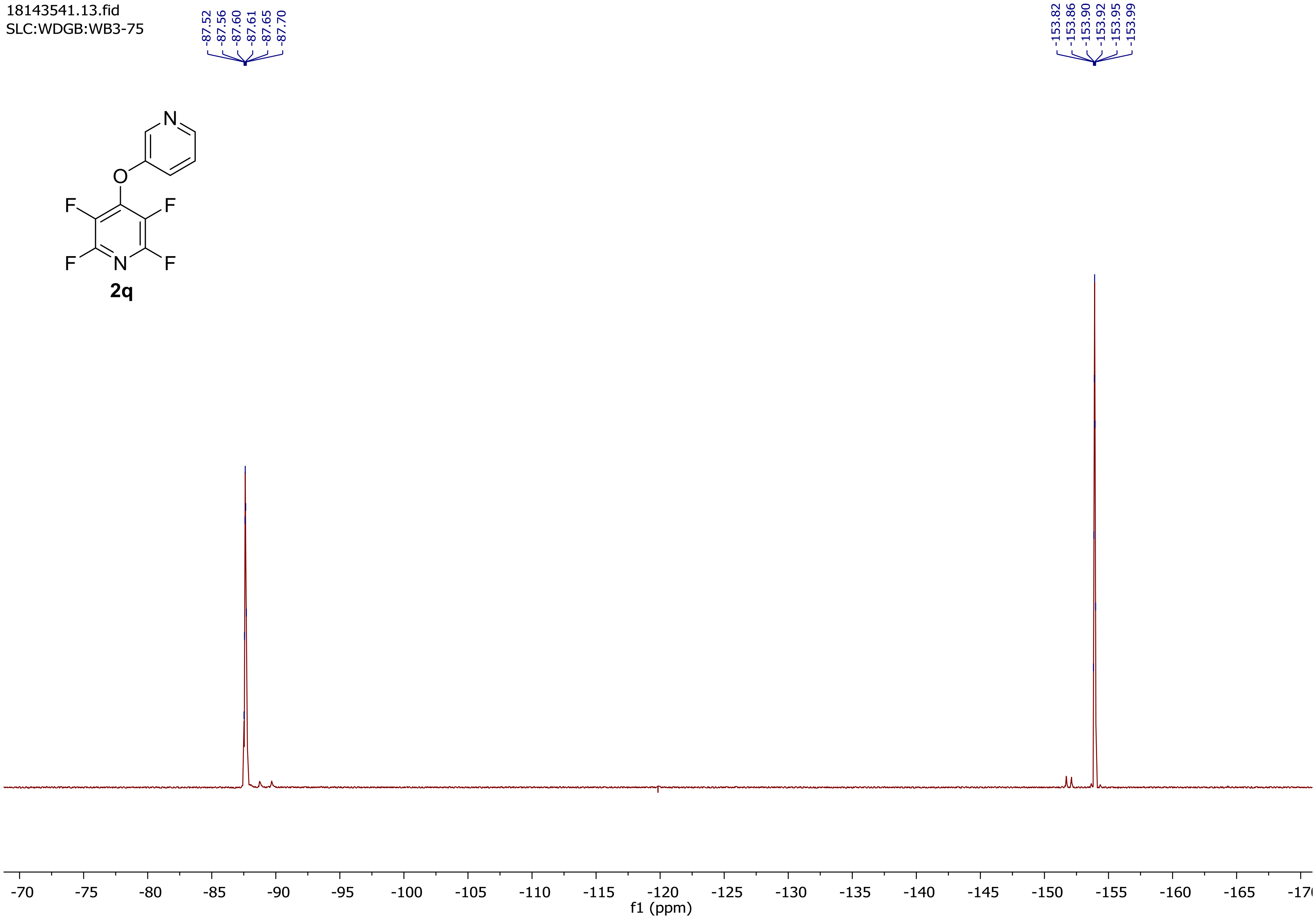

2q

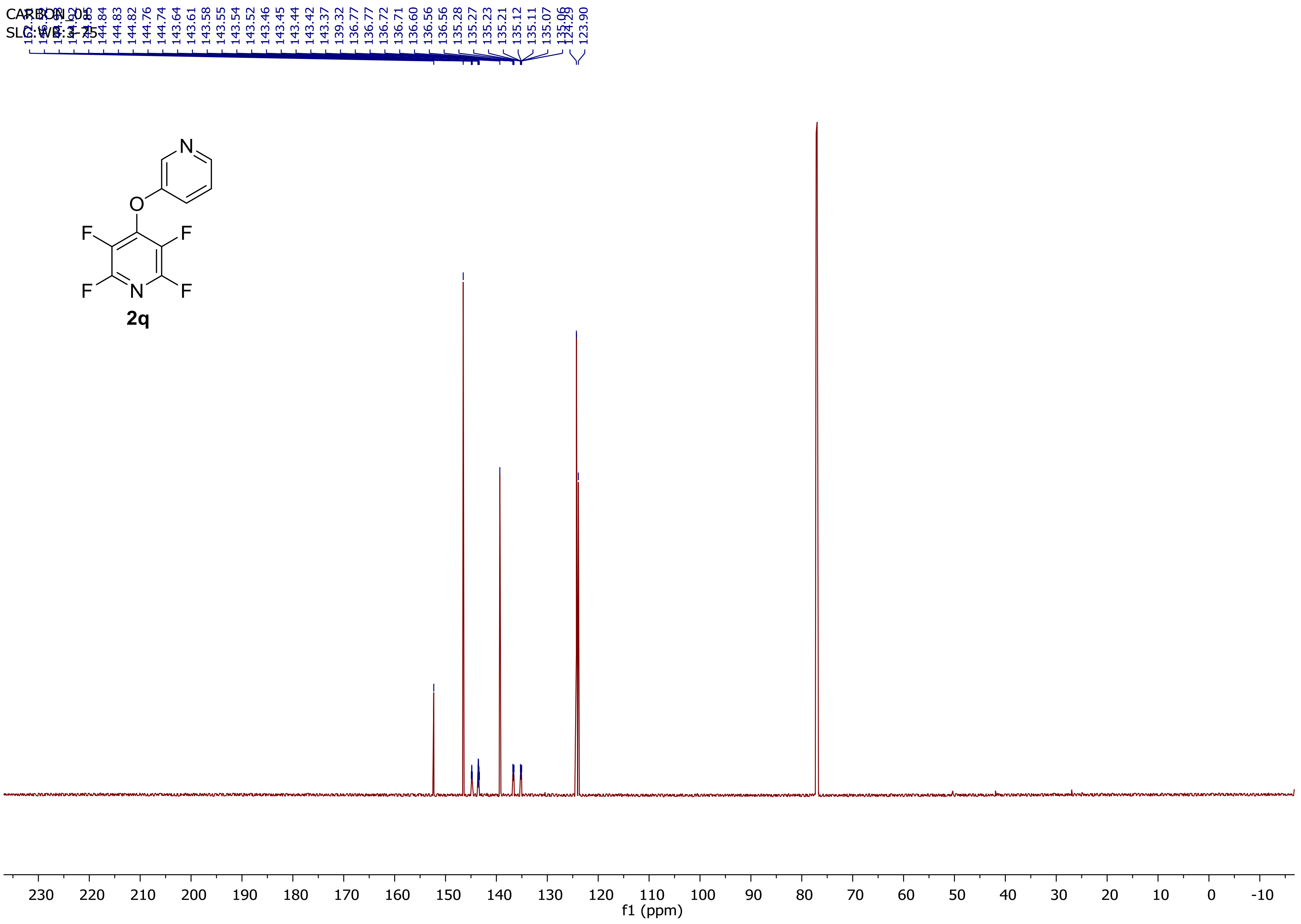

CARBON_01
SLC-WB-3-75
2q
f1 (ppm)

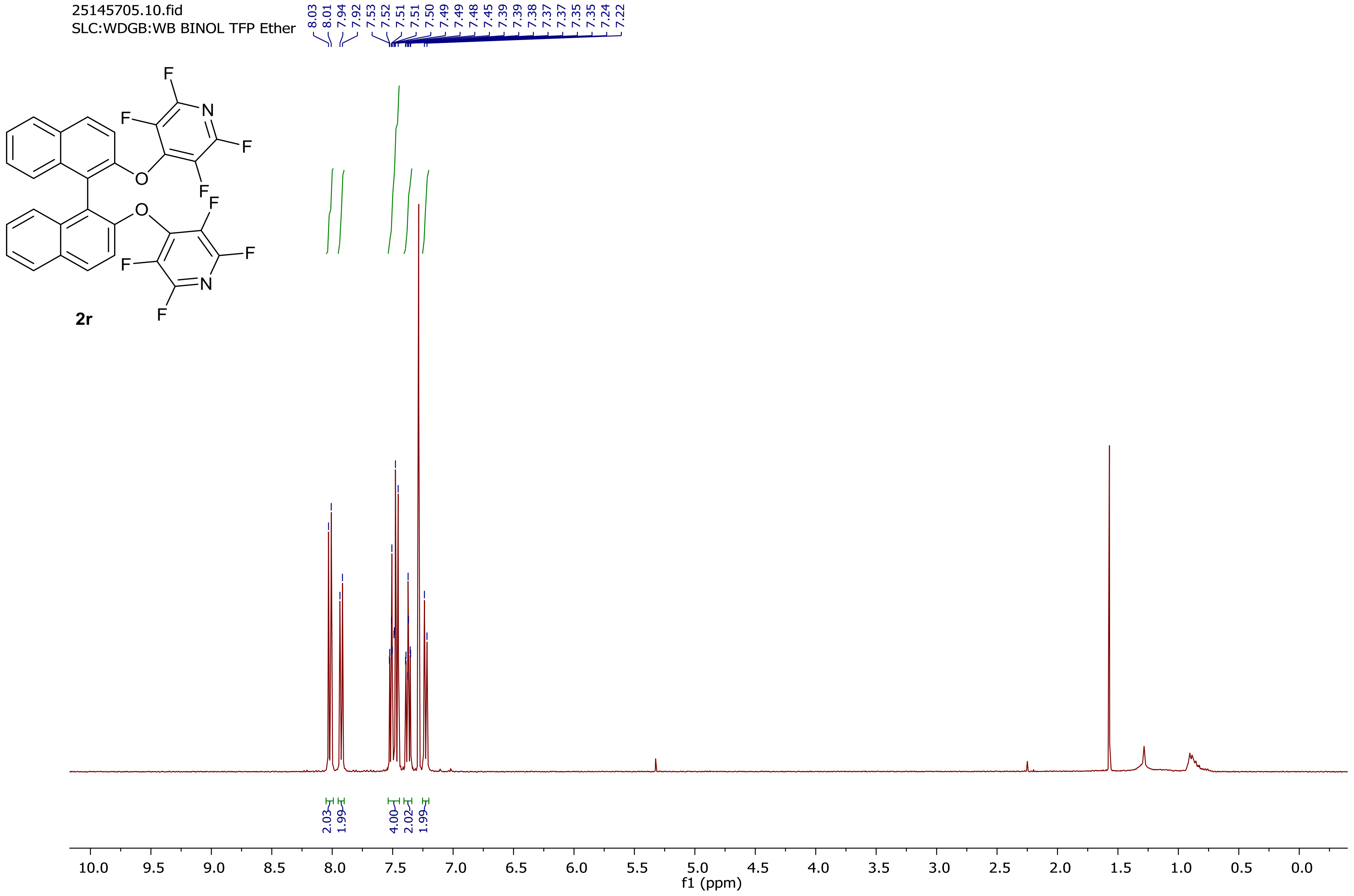
25145705.10.fid
SLC:WDGB:WB BINOL TFP Ether
8.03
8.01
7.94
7.92
7.53
7.52
7.51
7.51
7.50
7.49
7.49
7.48
7.45
7.39
7.39
7.38
7.37
7.37
7.35
7.35
7.24
7.22
F
N
O
2r
2.03
1.99
4.00
2.02
1.99
10.0
9.5
9.0
8.5
8.0
7.5
7.0
6.5
6.0
5.5
5.0
4.5
4.0
3.5
3.0
2.5
2.0
1.5
1.0
0.5
0.0
f1 (ppm)

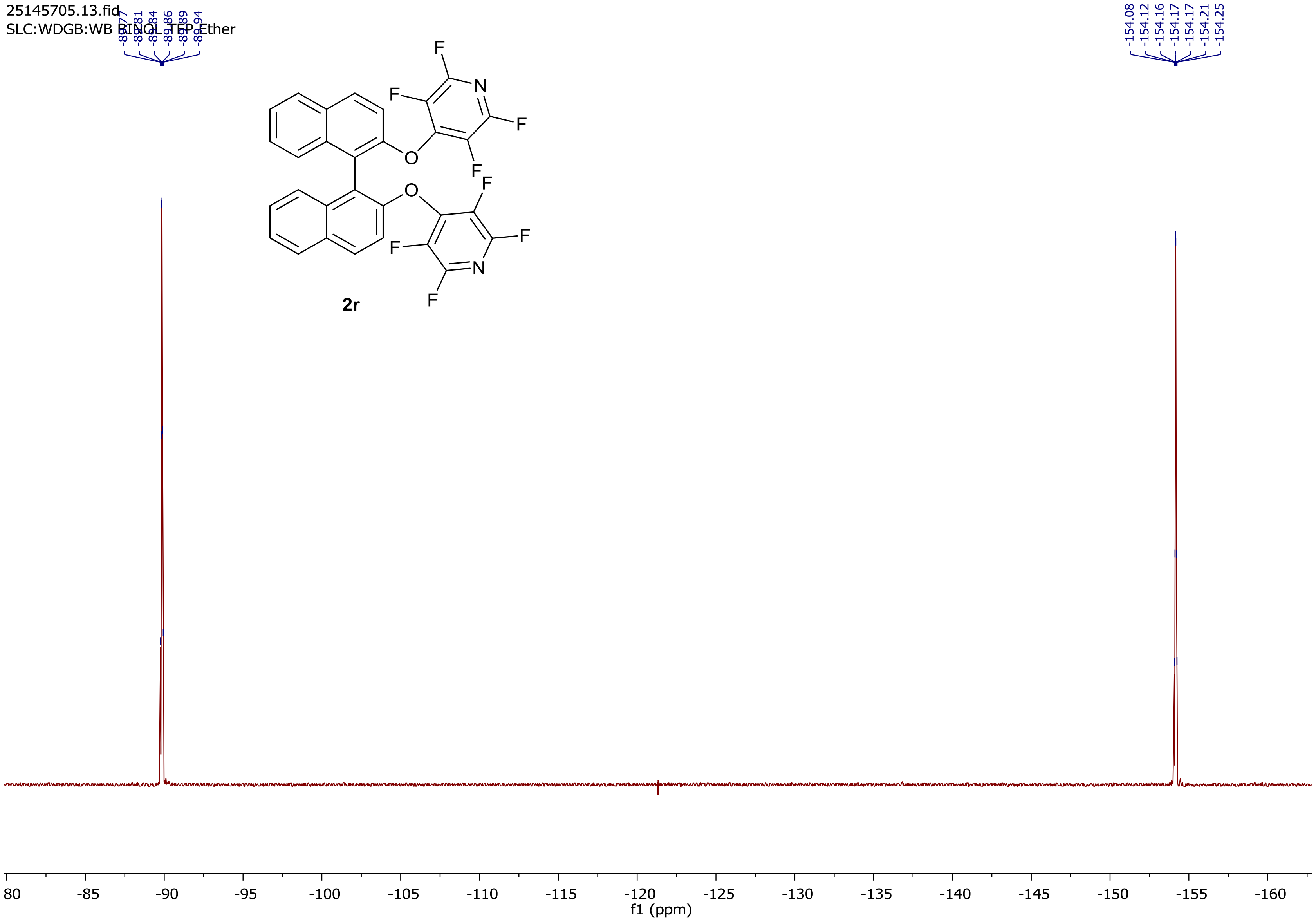
25145705.13.fid
SLC:WDGB:WB BINOL TFP Ether
-89.77
-89.81
-89.84
-89.86
-89.89
-89.94
-154.08
-154.12
-154.16
-154.17
-154.17
-154.21
-154.25
F
N
O
2r
80
-85
-90
-95
-100
-105
-110
-115
-120
-125
-130
-135
-140
-145
-150
-155
-160
f1 (ppm)

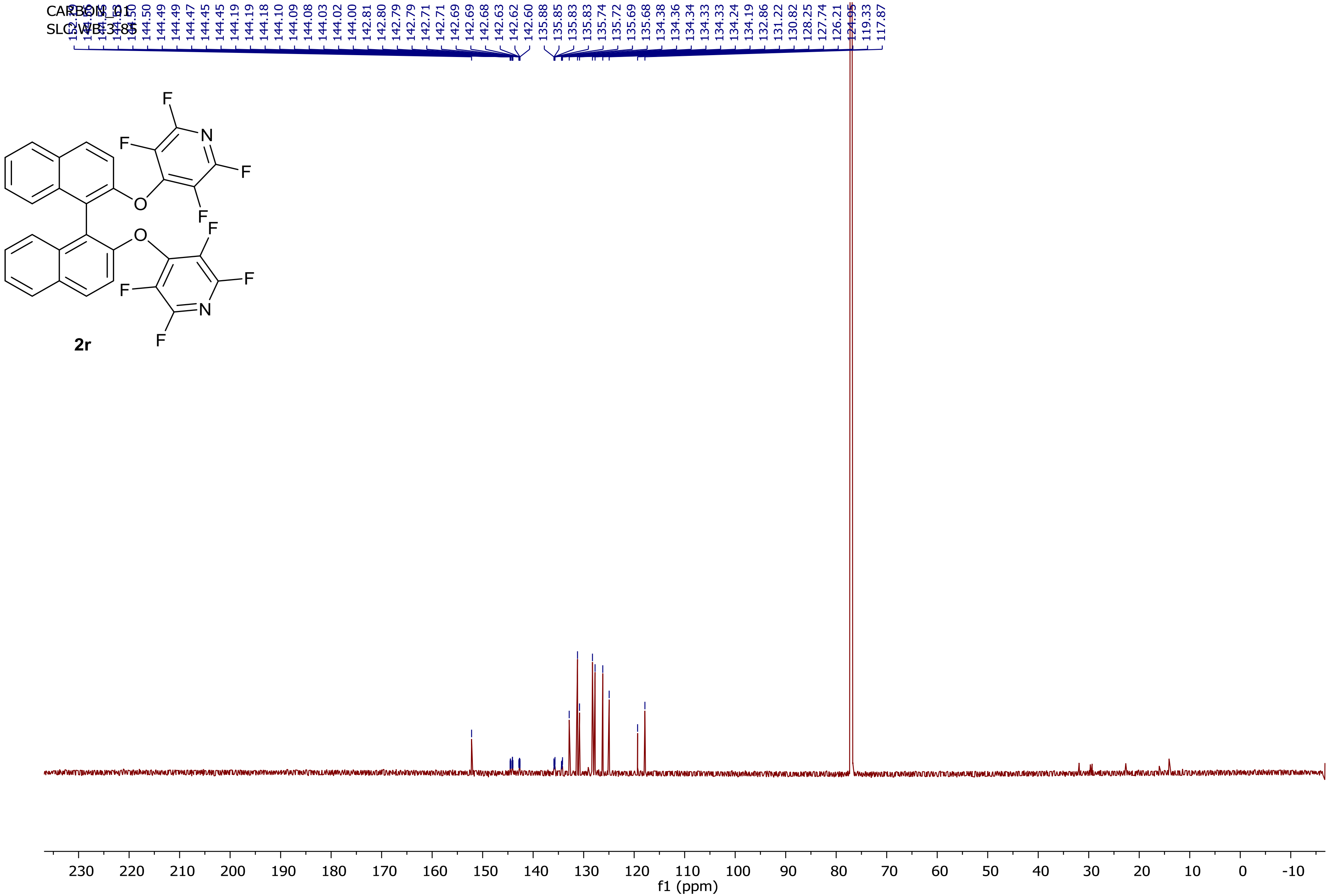
CARBON_01
SLC.WB.3.85
144.50
144.49
144.49
144.47
144.45
144.45
144.19
144.19
144.18
144.10
144.09
144.08
144.03
144.02
144.00
142.81
142.80
142.79
142.71
142.71
142.69
142.68
142.63
142.62
142.60
135.88
135.85
135.83
135.83
135.74
135.72
135.69
135.68
134.38
134.36
134.34
134.33
134.33
134.24
134.19
132.86
131.22
130.82
128.25
127.74
126.21
124.95
119.33
117.87
F
N
O
2r
230 220 210 200 190 180 170 160 150 140 130 120 110 100 90 80 70 60 50 40 30 20 10 0 -10
f1 (ppm)

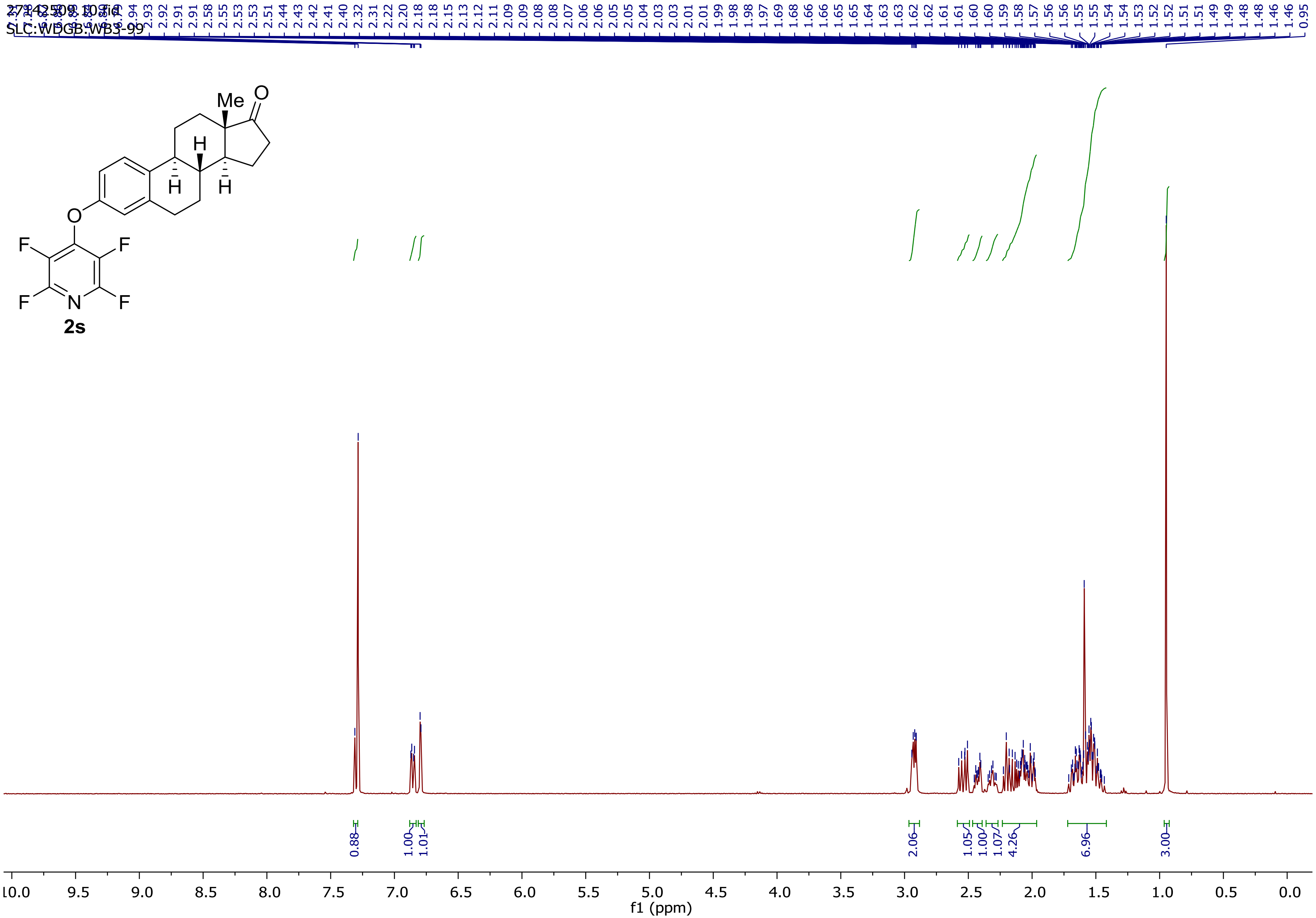

SLC:WDGB:WB3-99
2.93 2.92 2.91 2.91 2.58 2.55 2.53 2.53 2.51 2.44 2.43 2.42 2.41 2.40 2.32 2.31 2.22 2.20 2.18 2.18 2.15 2.13 2.12 2.11 2.09 2.08 2.08 2.07 2.06 2.06 2.05 2.05 2.04 2.03 2.03 2.01 2.01 1.99 1.98 1.98 1.97 1.69 1.68 1.66 1.66 1.65 1.65 1.64 1.63 1.63 1.62 1.62 1.61 1.61 1.60 1.60 1.59 1.58 1.57 1.56 1.56 1.55 1.55 1.54 1.54 1.53 1.52 1.52 1.51 1.51 1.49 1.48 1.48 1.46 1.46 0.95
Me
O
H
H
H
O
F
F
F
N
F
2s
0.88
1.00
1.01
2.06
1.05
1.00
1.07
4.26
6.96
3.00
10.0 9.5 9.0 8.5 8.0 7.5 7.0 6.5 6.0 5.5 5.0 4.5 4.0 3.5 3.0 2.5 2.0 1.5 1.0 0.5 0.0
f1 (ppm)

27142509.13.fid
SLC:WDGB:WB3-99

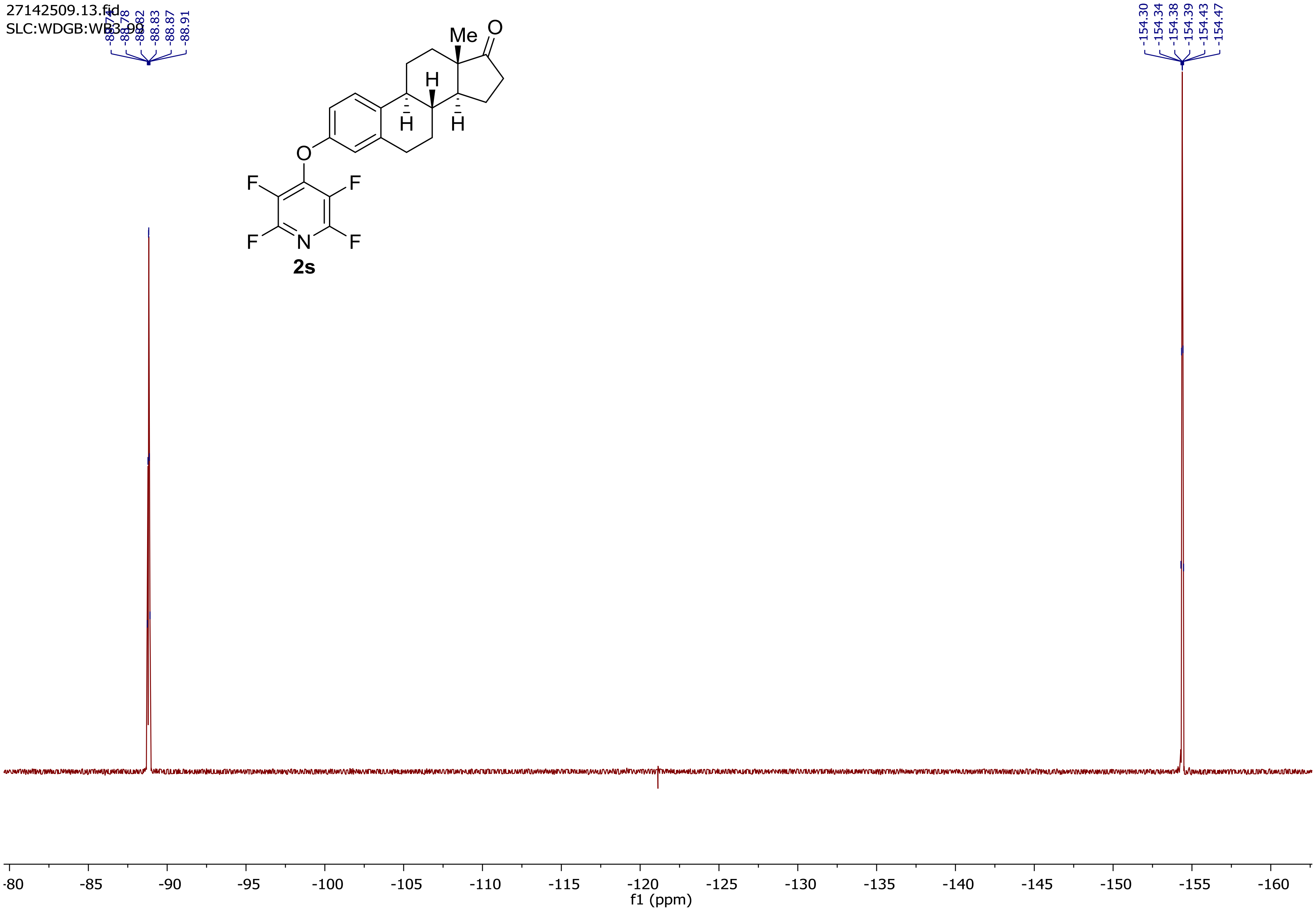

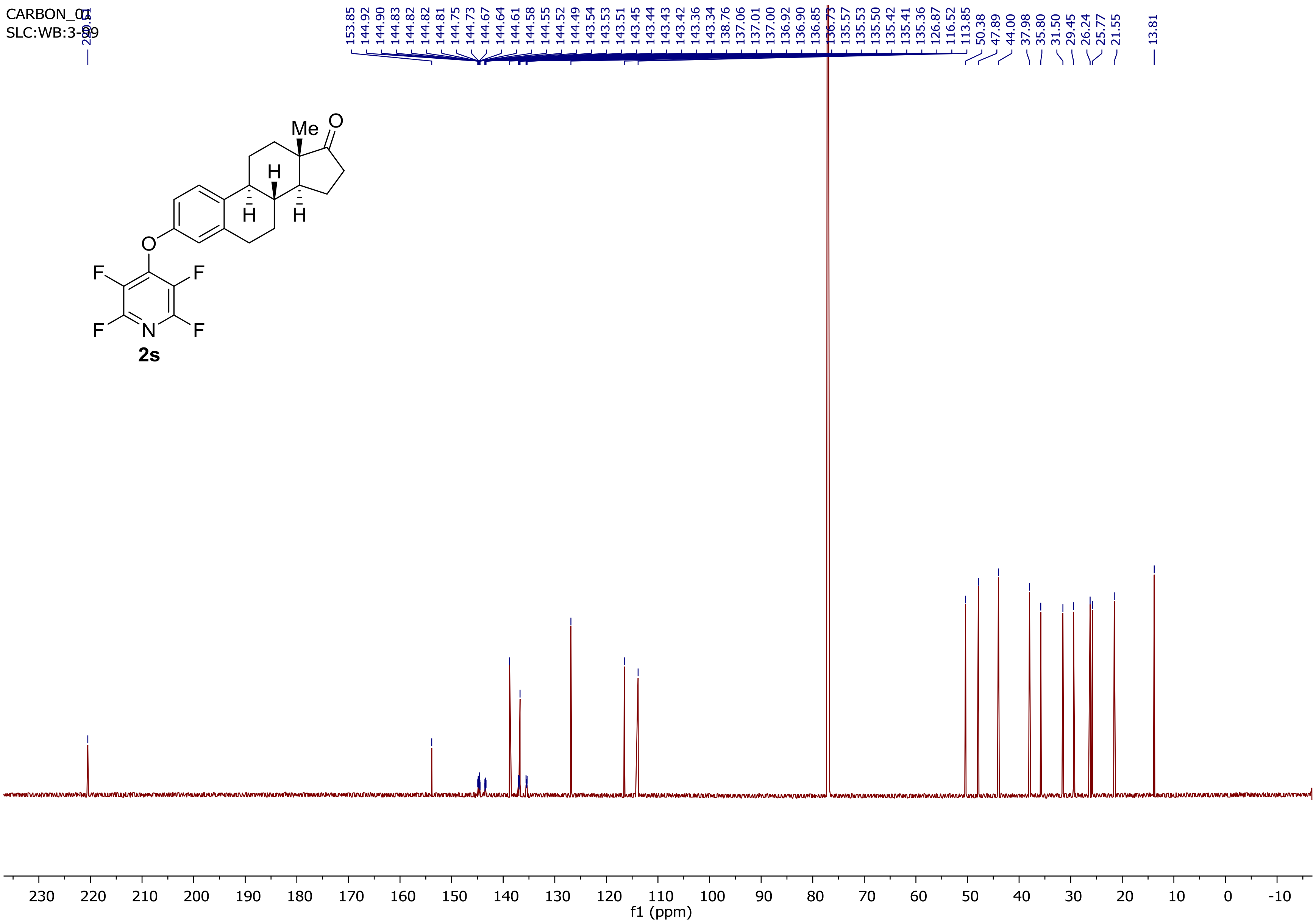
SLC:WB:3-89
153.85
144.92
144.90
144.83
144.82
144.82
144.81
144.75
144.73
144.67
144.64
144.61
144.58
144.55
144.52
144.49
143.54
143.53
143.51
143.45
143.44
143.43
143.42
143.36
143.34
138.76
137.06
137.01
137.00
136.92
136.90
136.85
135.57
135.53
135.50
135.42
135.41
135.36
126.87
116.52
113.85
50.38
47.89
44.00
37.98
35.80
31.50
29.45
26.24
25.77
21.55
13.81
Me
O
H
H
H
O
F
F
F
N
F
2s
f1 (ppm)

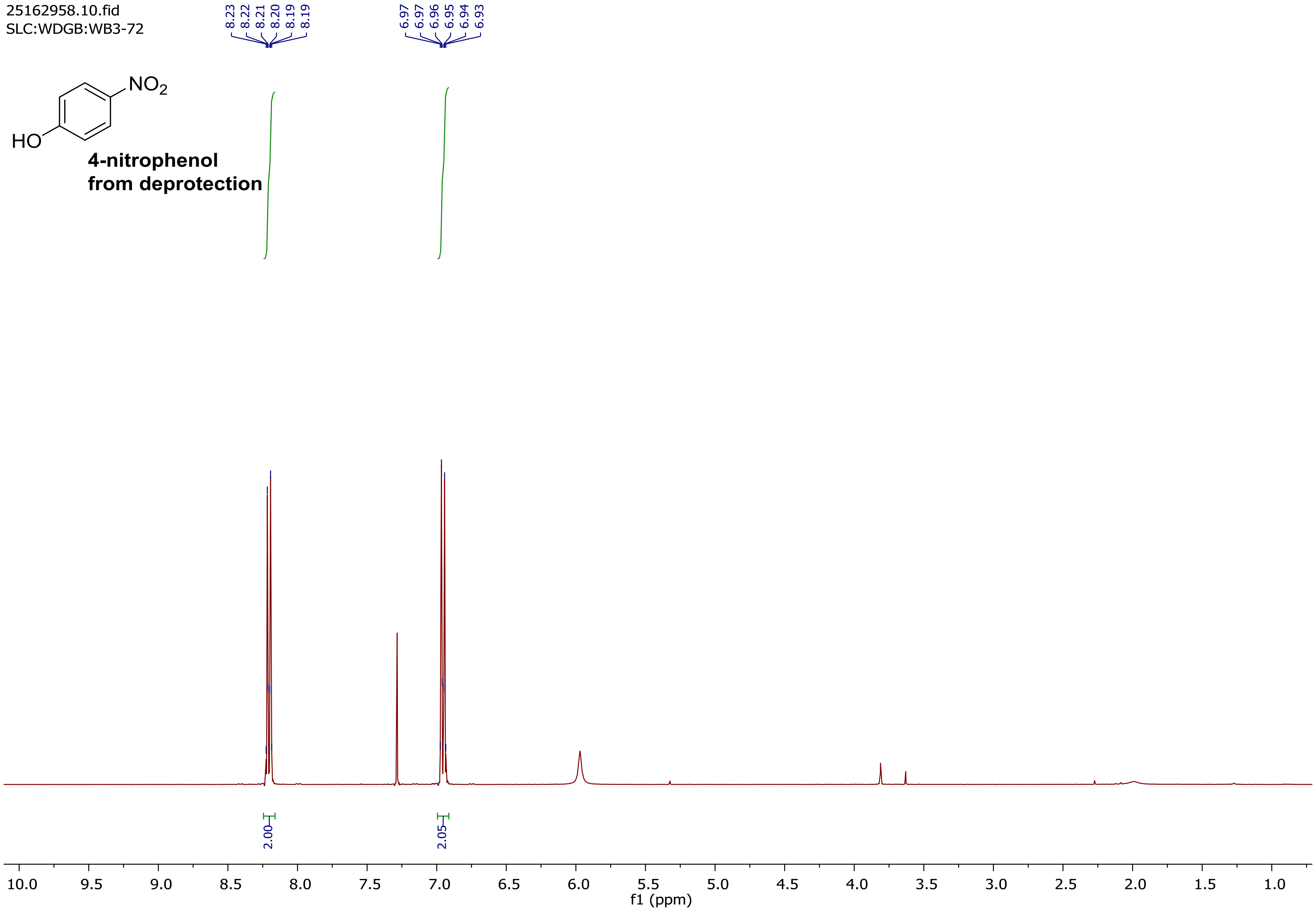
25162958.10.fid
SLC:WDGB:WB3-72
NO2
HO
4-nitrophenol
from deprotection
8.23
8.22
8.21
8.20
8.19
8.19
6.97
6.97
6.96
6.95
6.94
6.93
2.00
2.05
10.0
9.5
9.0
8.5
8.0
7.5
7.0
6.5
6.0
5.5
5.0
4.5
4.0
3.5
3.0
2.5
2.0
1.5
1.0
f1 (ppm)

25162958.11.fid
SLC:WDGB:WB3-72

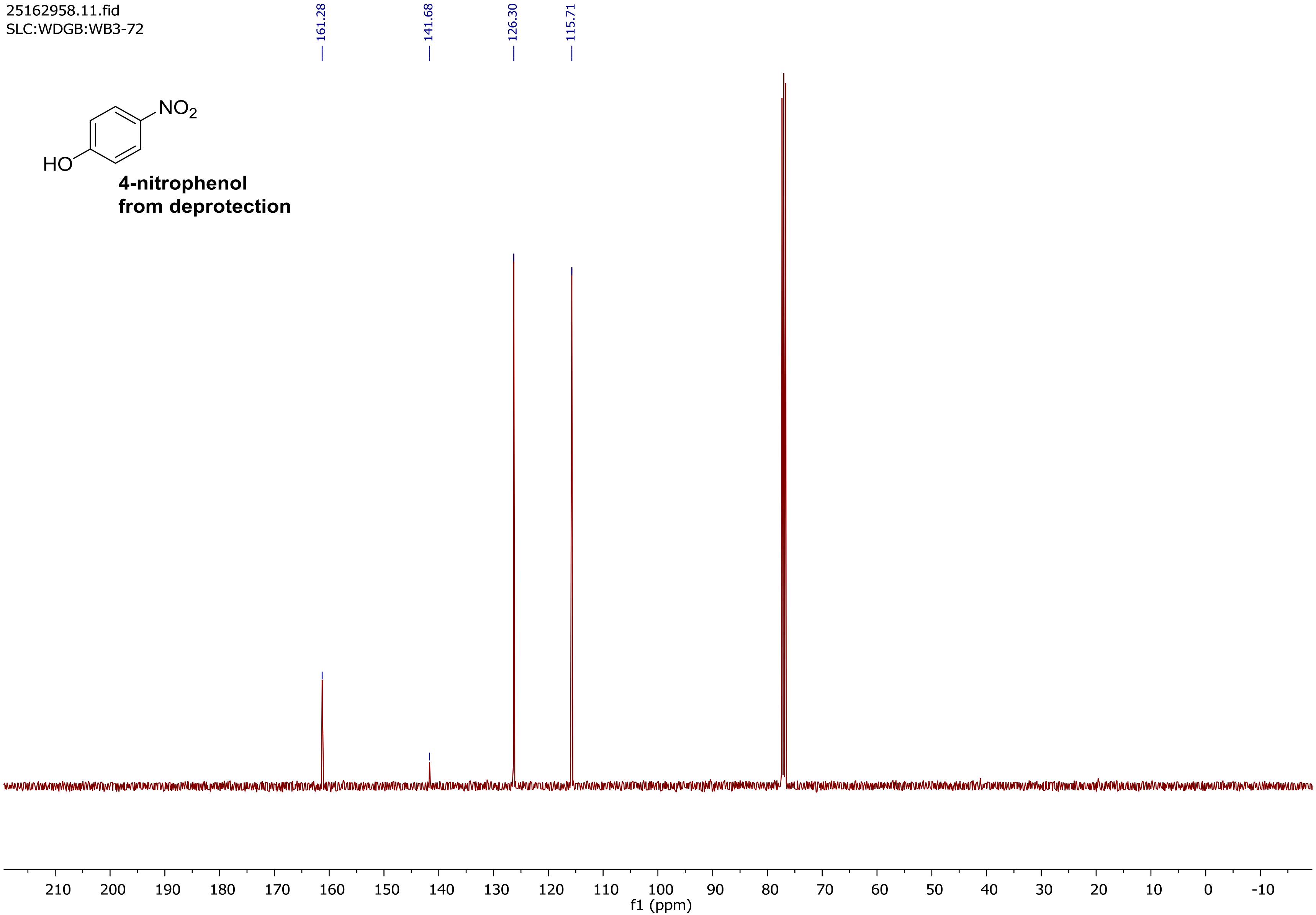

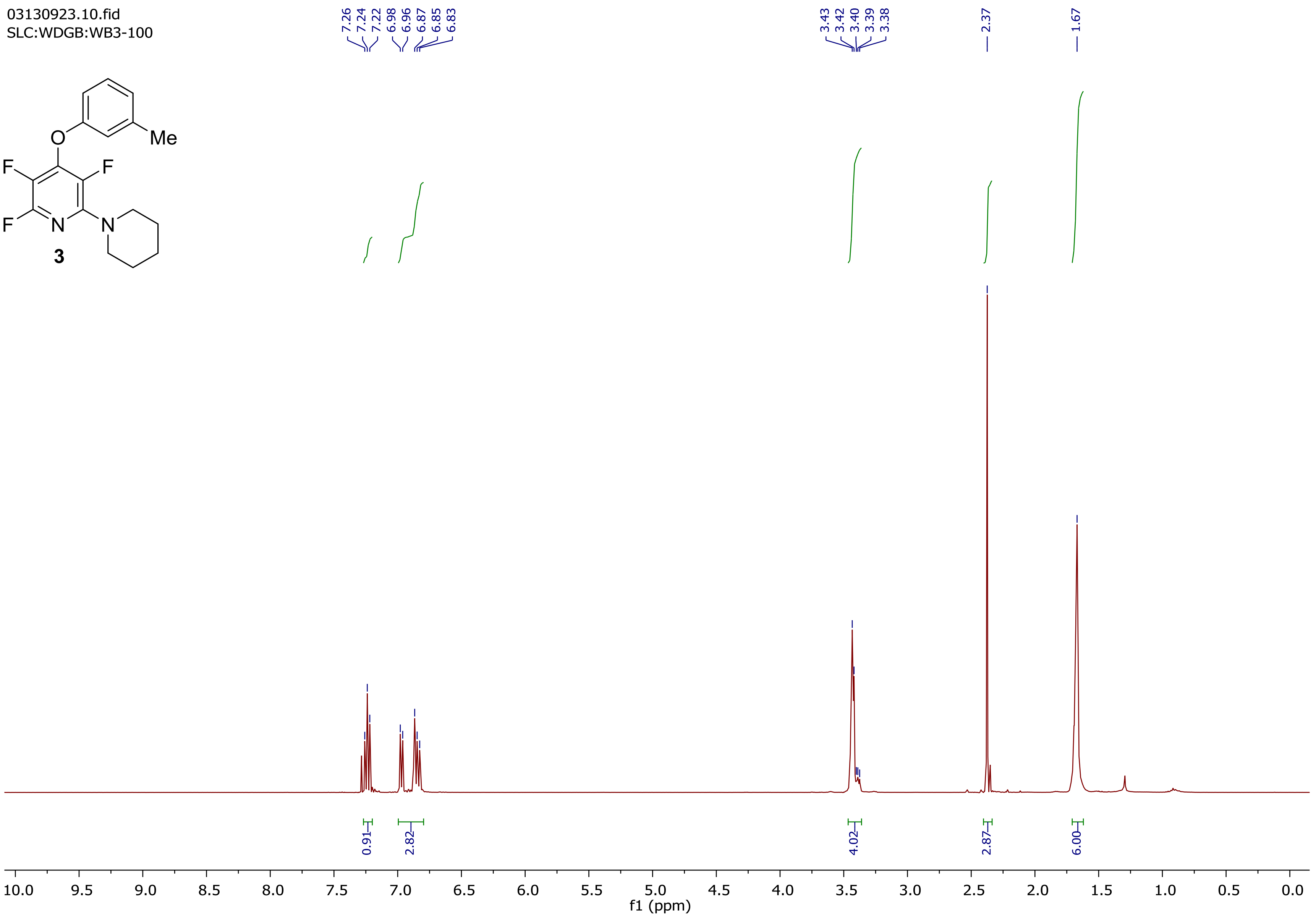
03130923.10.fid
SLC:WDGB:WB3-100
Me
O
F
F
F
N
N
3
7.26
7.24
7.22
6.98
6.96
6.87
6.85
6.83
3.43
3.42
3.40
3.39
3.38
2.37
1.67
0.91
2.82
4.02
2.87
6.00
10.0 9.5 9.0 8.5 8.0 7.5 7.0 6.5 6.0 5.5 5.0 4.5 4.0 3.5 3.0 2.5 2.0 1.5 1.0 0.5 0.0
f1 (ppm)

03130923.13.fid
SLC:WDGB:WB3-100

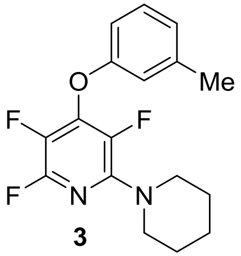

3

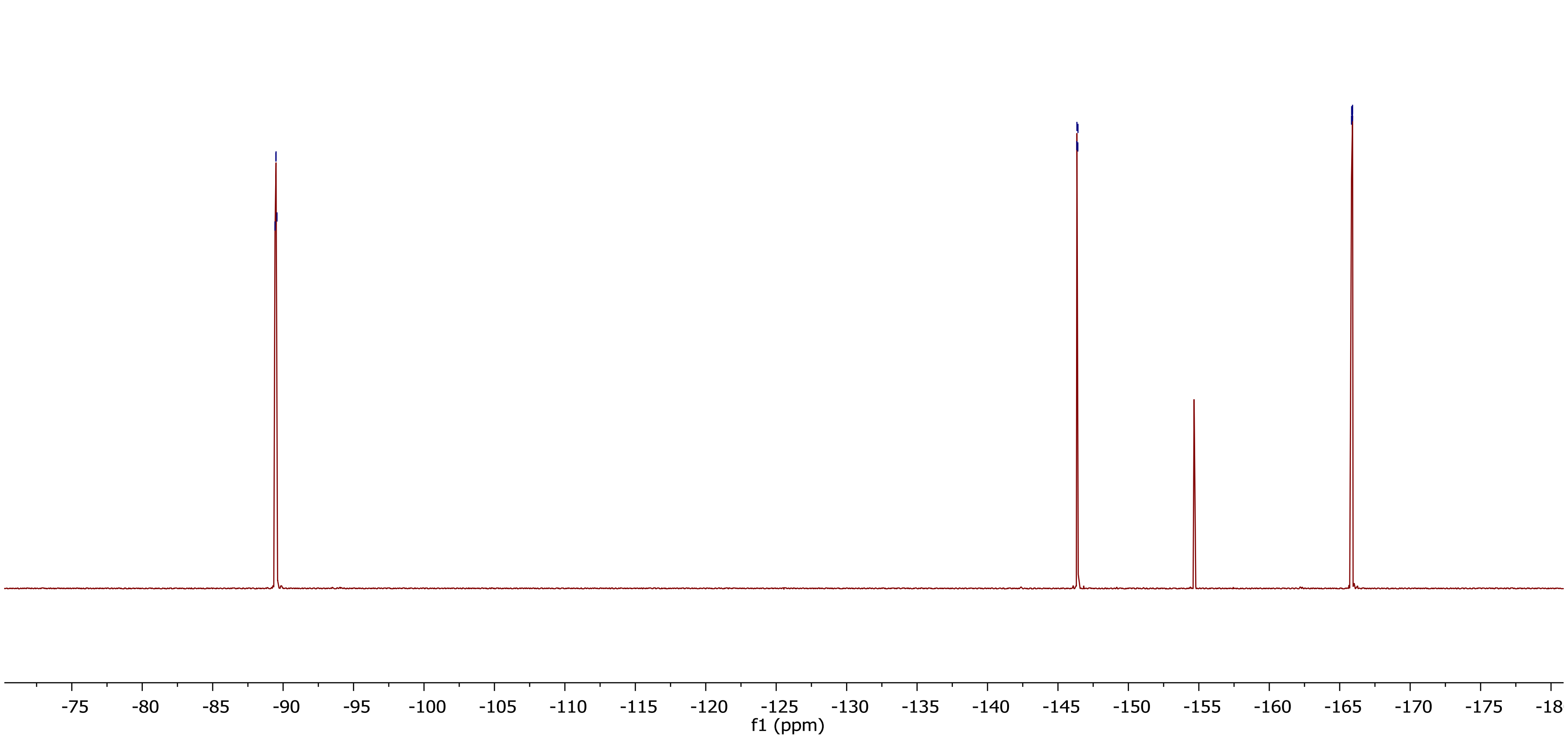

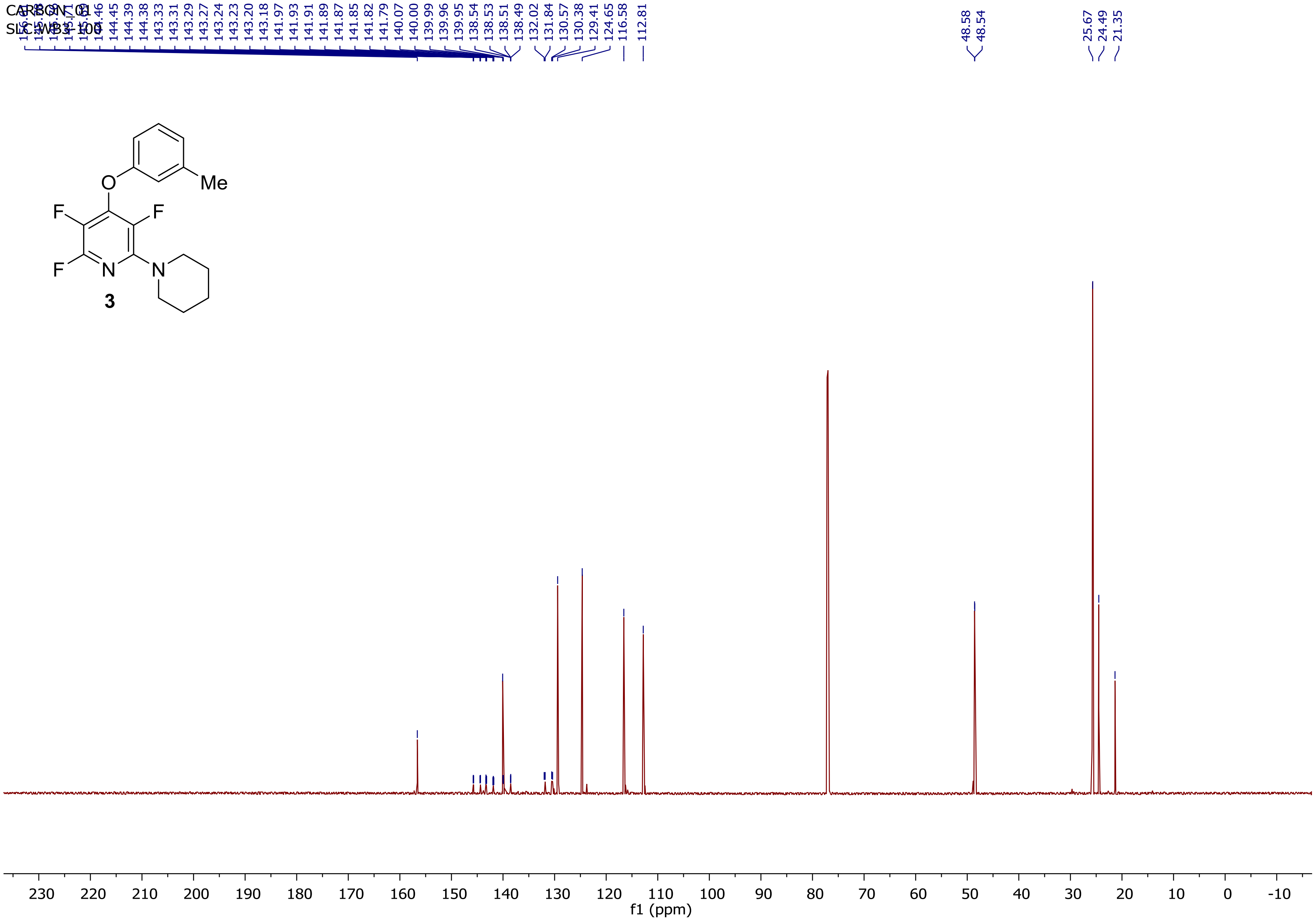
CARBON_01
SLC-WB3-100
156.61
145.78
145.76
145.71
145.69
144.46
144.45
144.39
144.38
143.33
143.31
143.29
143.27
143.24
143.23
143.20
143.18
141.97
141.93
141.91
141.89
141.87
141.85
141.82
141.79
140.07
140.00
139.99
139.96
139.95
138.54
138.53
138.51
138.49
132.02
131.84
130.57
130.38
129.41
124.65
116.58
112.81
48.58
48.54
25.67
24.49
21.35
O
Me
F
F
F
N
N
3
230
220
210
200
190
180
170
160
150
140
130
120
110
100
90
80
70
60
50
40
30
20
10
0
-10
f1 (ppm)

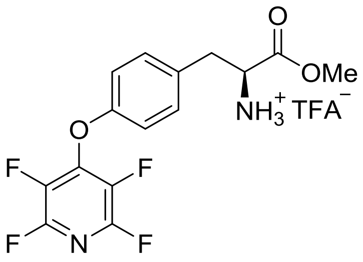
O
OMe
NH_3^+ TFA^-
O
F
F
F
N
F

4

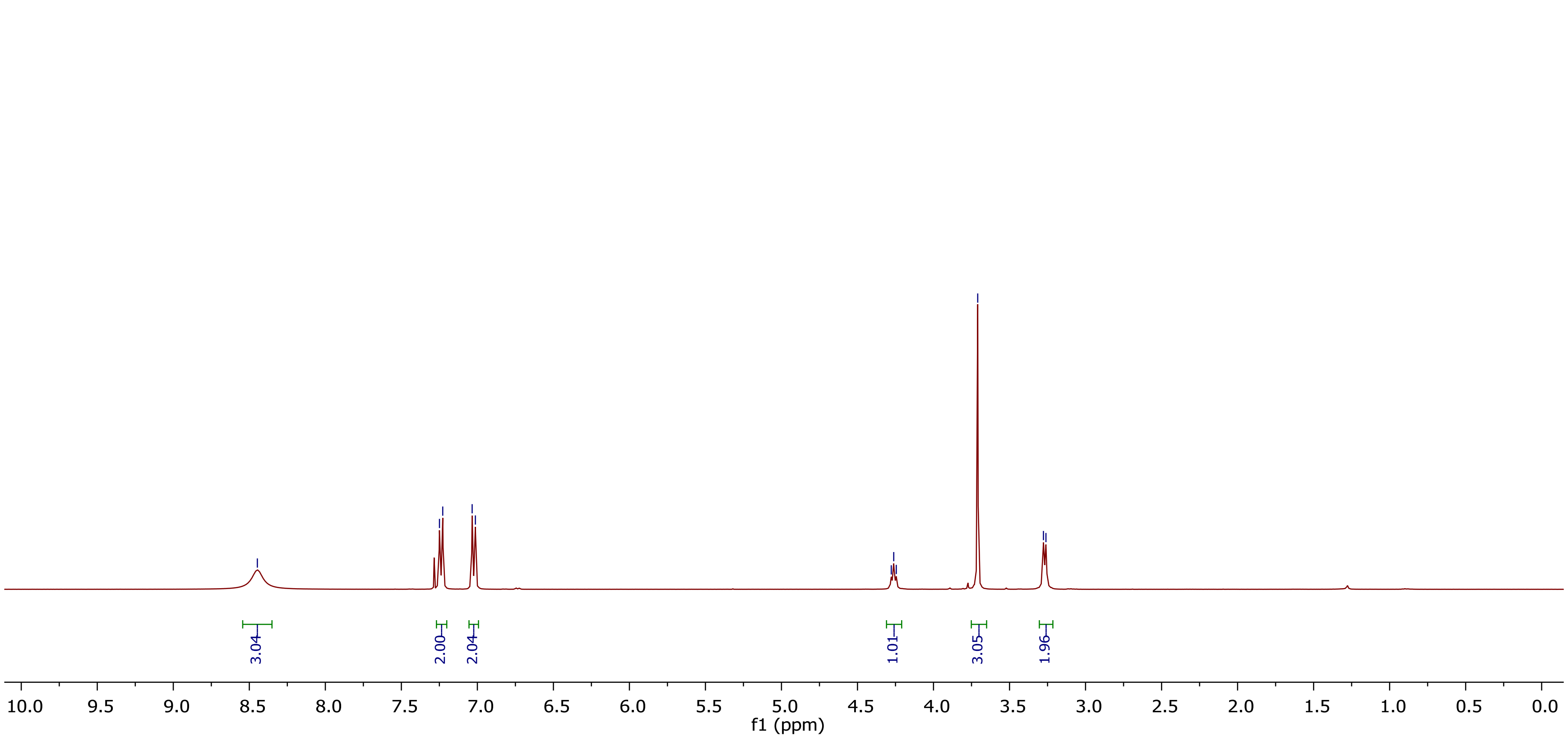
03142603.10.fid
SLC:WDGB:WB3-106
8.45
7.25
7.23
7.03
7.01
4.28
4.26
4.25
3.71
3.28
3.26
3.04
2.00
2.04
1.01
3.05
1.96
10.0 9.5 9.0 8.5 8.0 7.5 7.0 6.5 6.0 5.5 5.0 4.5 4.0 3.5 3.0 2.5 2.0 1.5 1.0 0.5 0.0
f1 (ppm)

03142603.13.fid
SLC:WDGB:WB3-106

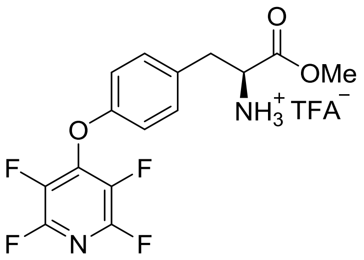

4

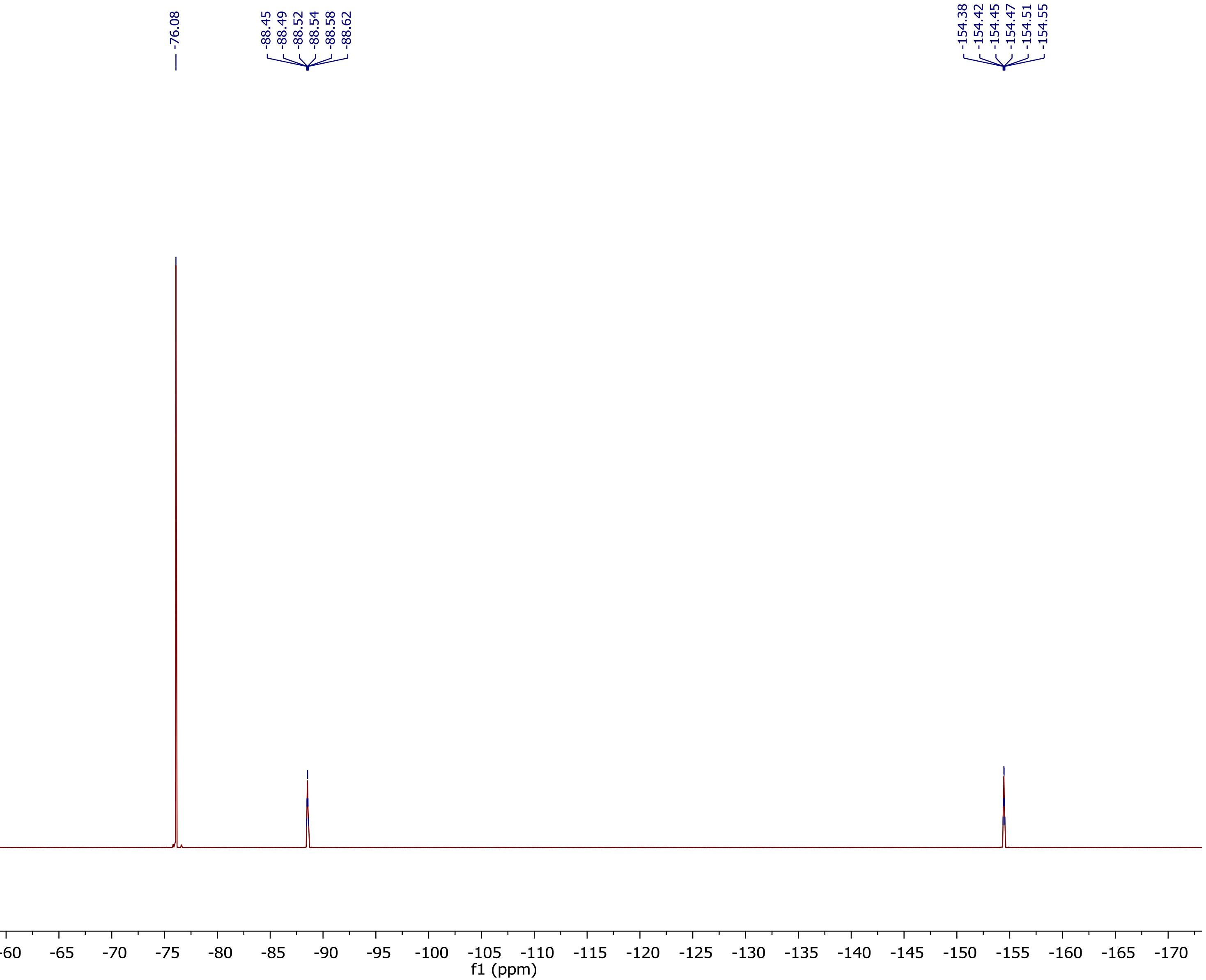

CARBON_01
SLC:WB3-106

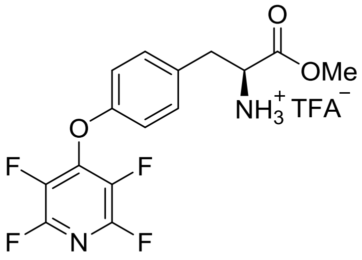

4

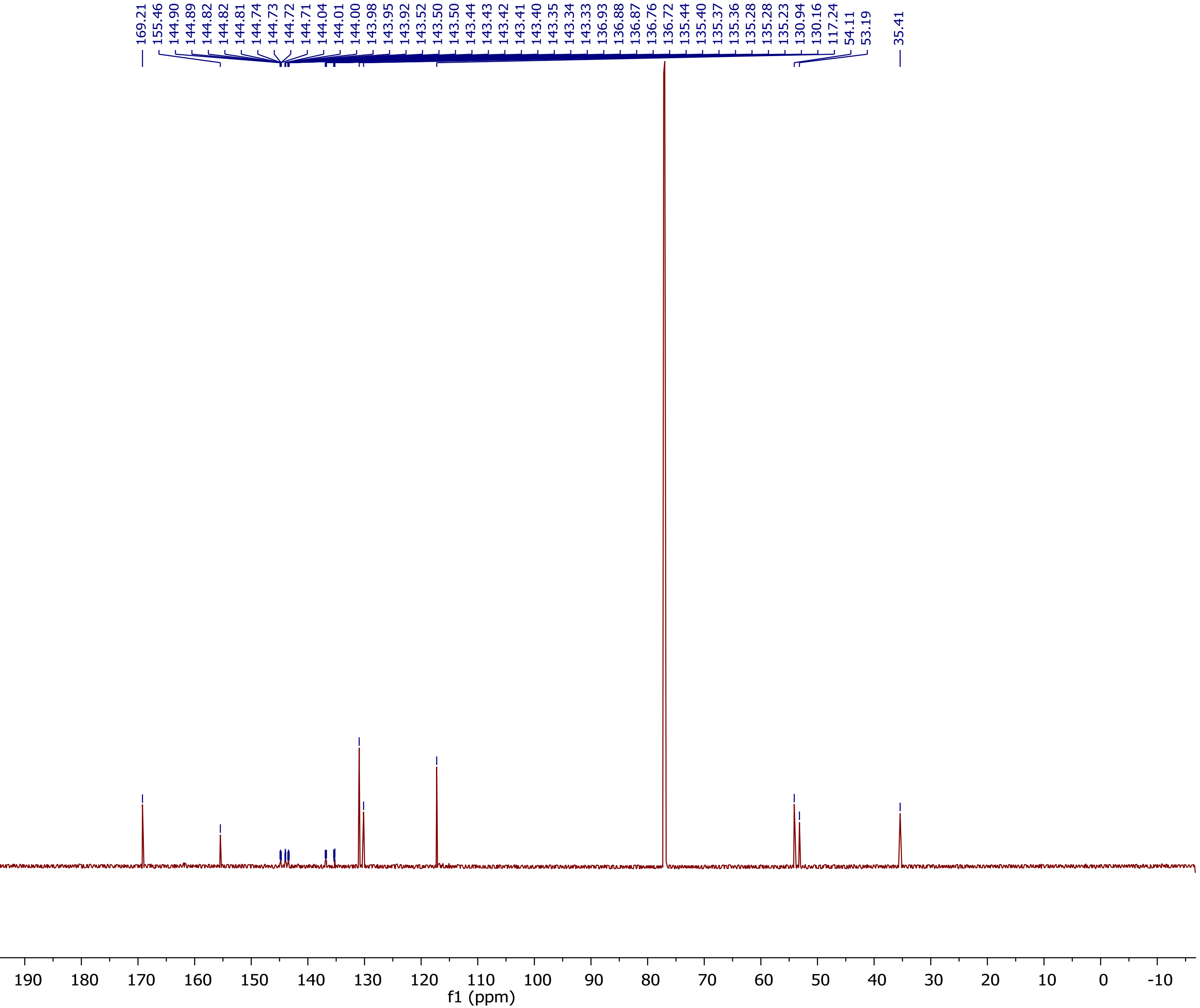

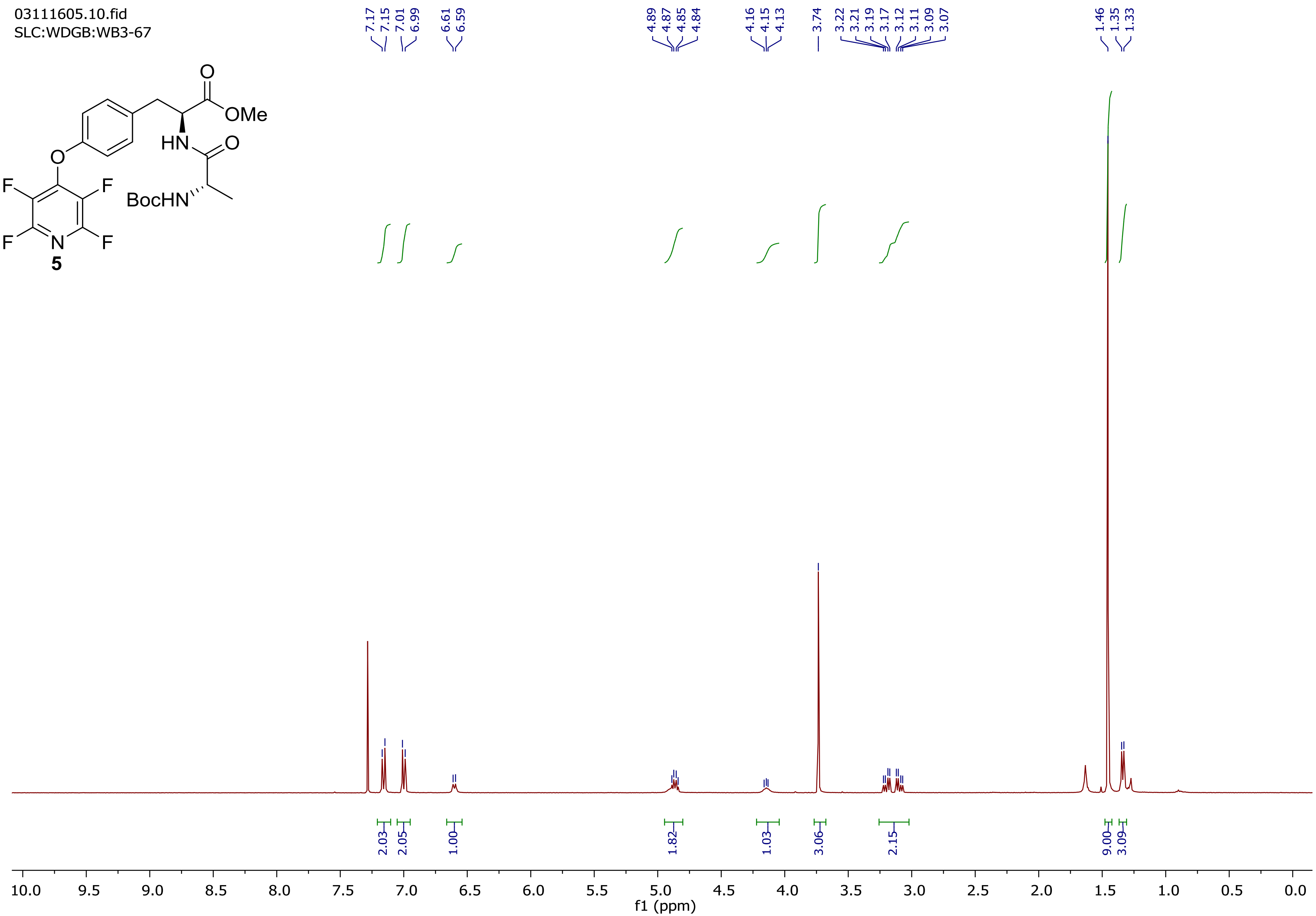
03111605.10.fid
SLC:WDGB:WB3-67
OMe
HN
BocHN
O
F
N
5
7.17 7.15 7.01 6.99 6.61 6.59
4.89 4.87 4.85 4.84
4.16 4.15 4.13
3.74
3.22 3.21 3.19 3.17 3.12 3.11 3.09 3.07
1.46 1.35 1.33
2.03 2.05 1.00 1.82 1.03 3.06 2.15 9.00 3.09
10.0 9.5 9.0 8.5 8.0 7.5 7.0 6.5 6.0 5.5 5.0 4.5 4.0 3.5 3.0 2.5 2.0 1.5 1.0 0.5 0.0
f1 (ppm)

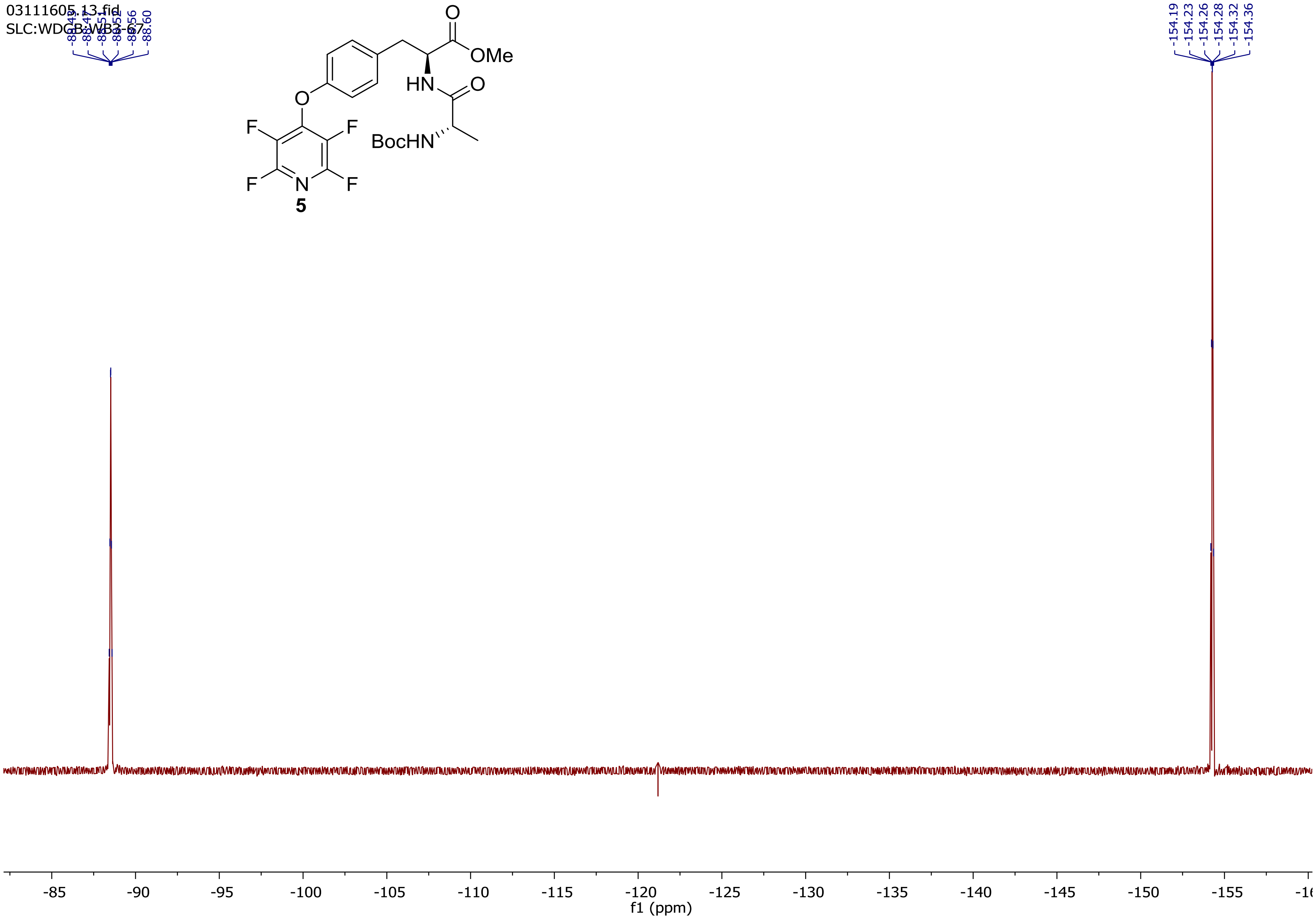
03111605.13.fid
SLC:WDCB:WB3-67
-88.43
-88.47
-88.51
-88.52
-88.56
-88.60
-154.19
-154.23
-154.26
-154.28
-154.32
-154.36
O
OMe
HN
O
O
F
F
BocHN
F
N
F
5
-85
-90
-95
-100
-105
-110
-115
-120
-125
-130
-135
-140
-145
-150
-155
f1 (ppm)

CARBON_01
SLC:WB:3-67

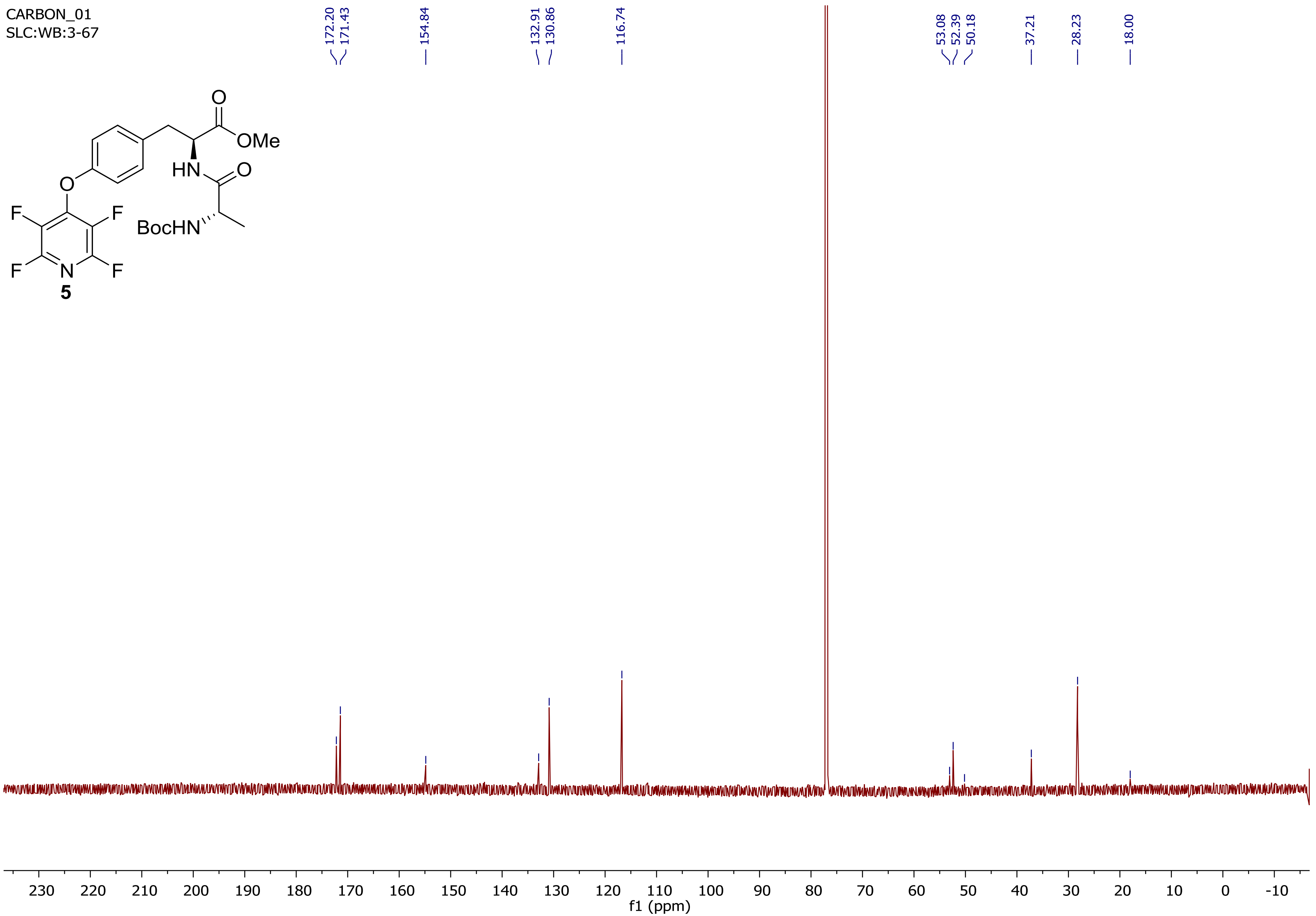

12110858.10.fid
SLC:WDGB:WB3-113

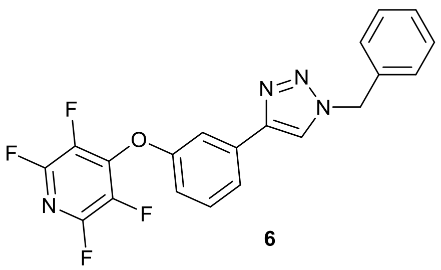

6

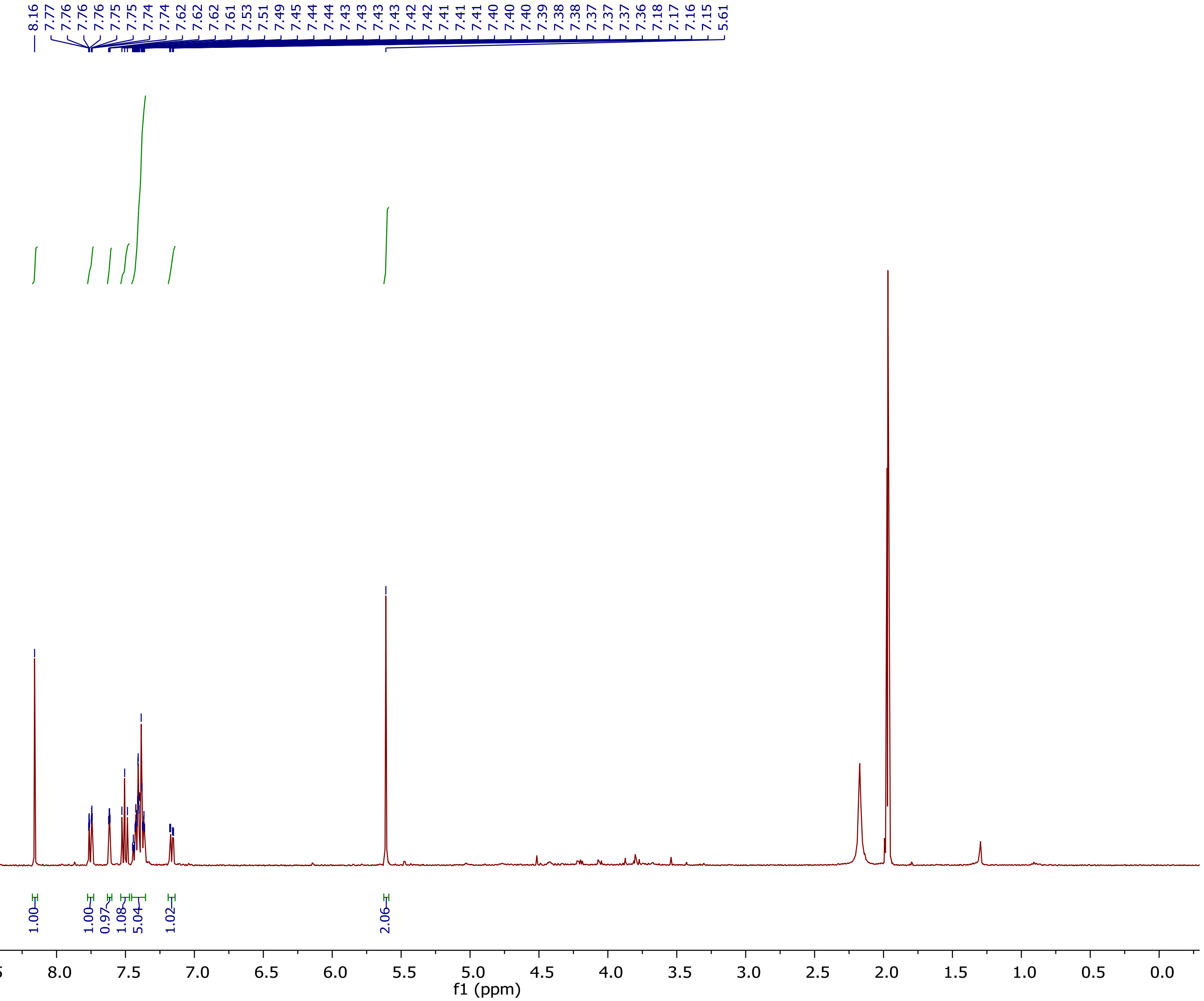

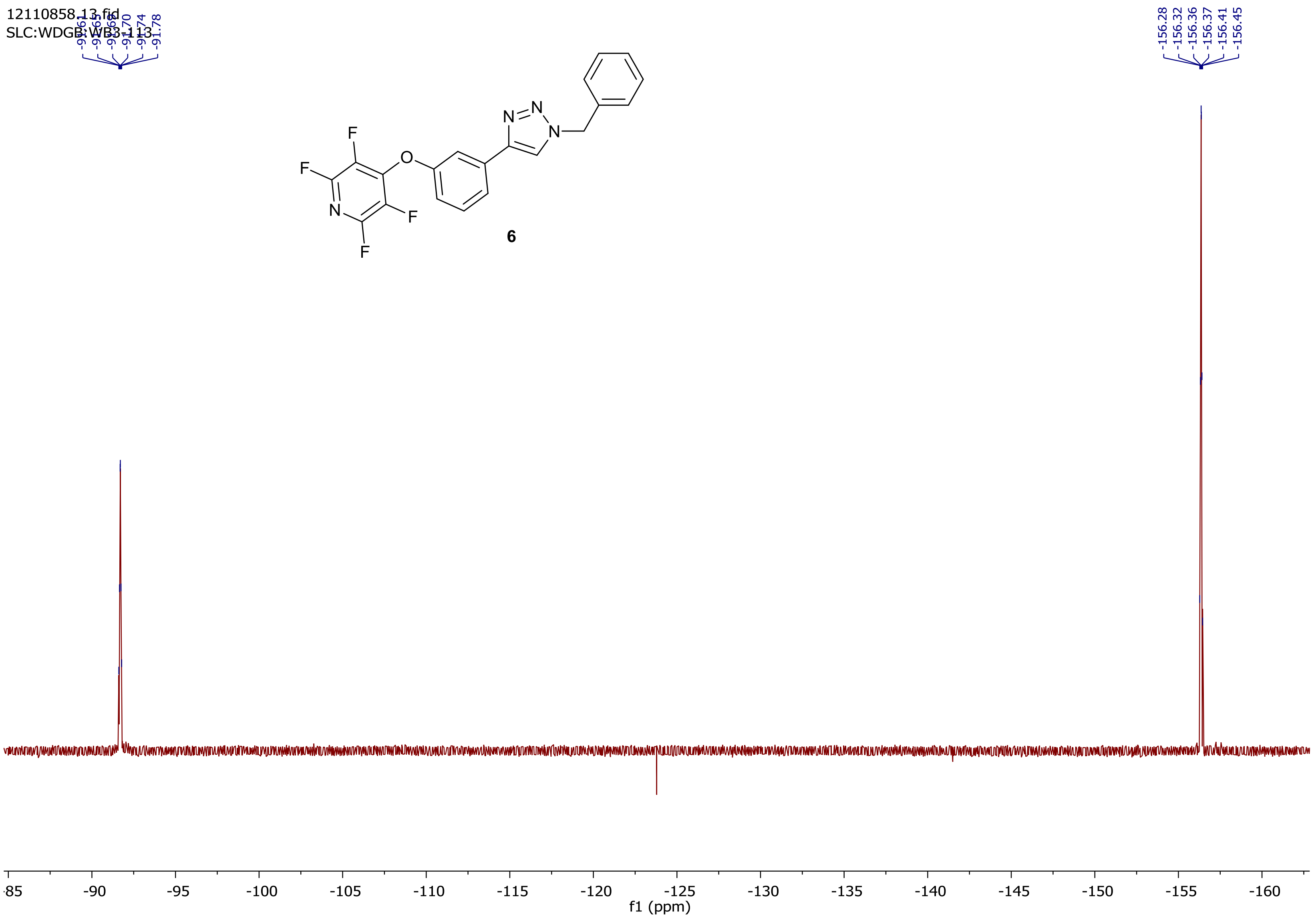

12110858.13.fid
SLC:WDGB:WB3-113
-91.61
-91.65
-91.69
-91.70
-91.74
-91.78
-156.28
-156.32
-156.36
-156.37
-156.41
-156.45
F
F
N
F
F
O
N
N
N
6
85
-90
-95
-100
-105
-110
-115
-120
-125
-130
-135
-140
-145
-150
-155
-160
f1 (ppm)

CARBON_01
SLC:WB3-113

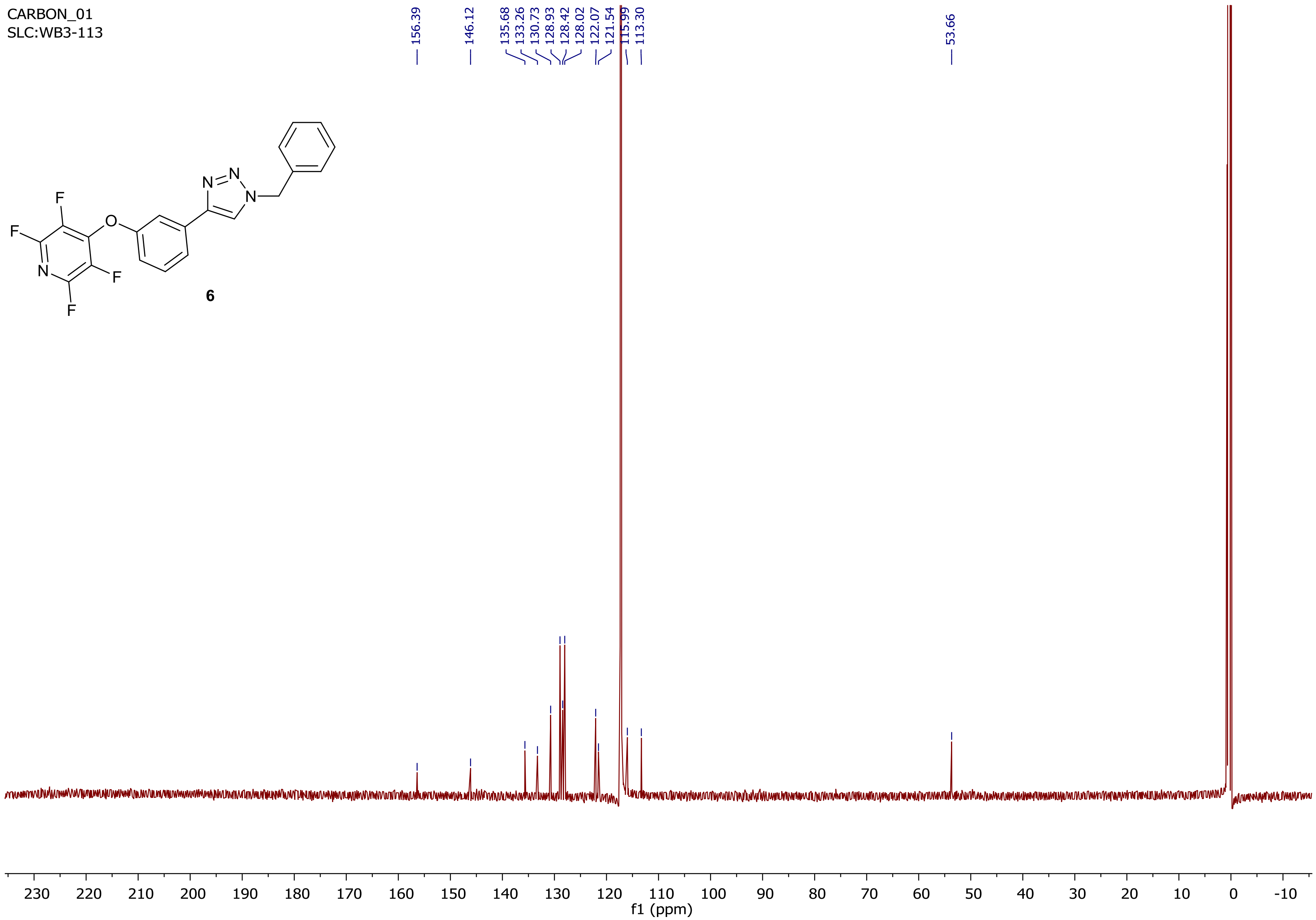

12110836.10.fid
SLC:WDGB:WB3-110

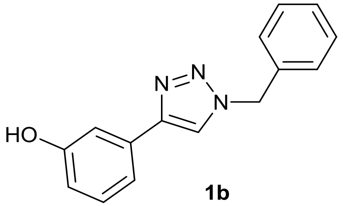

1b

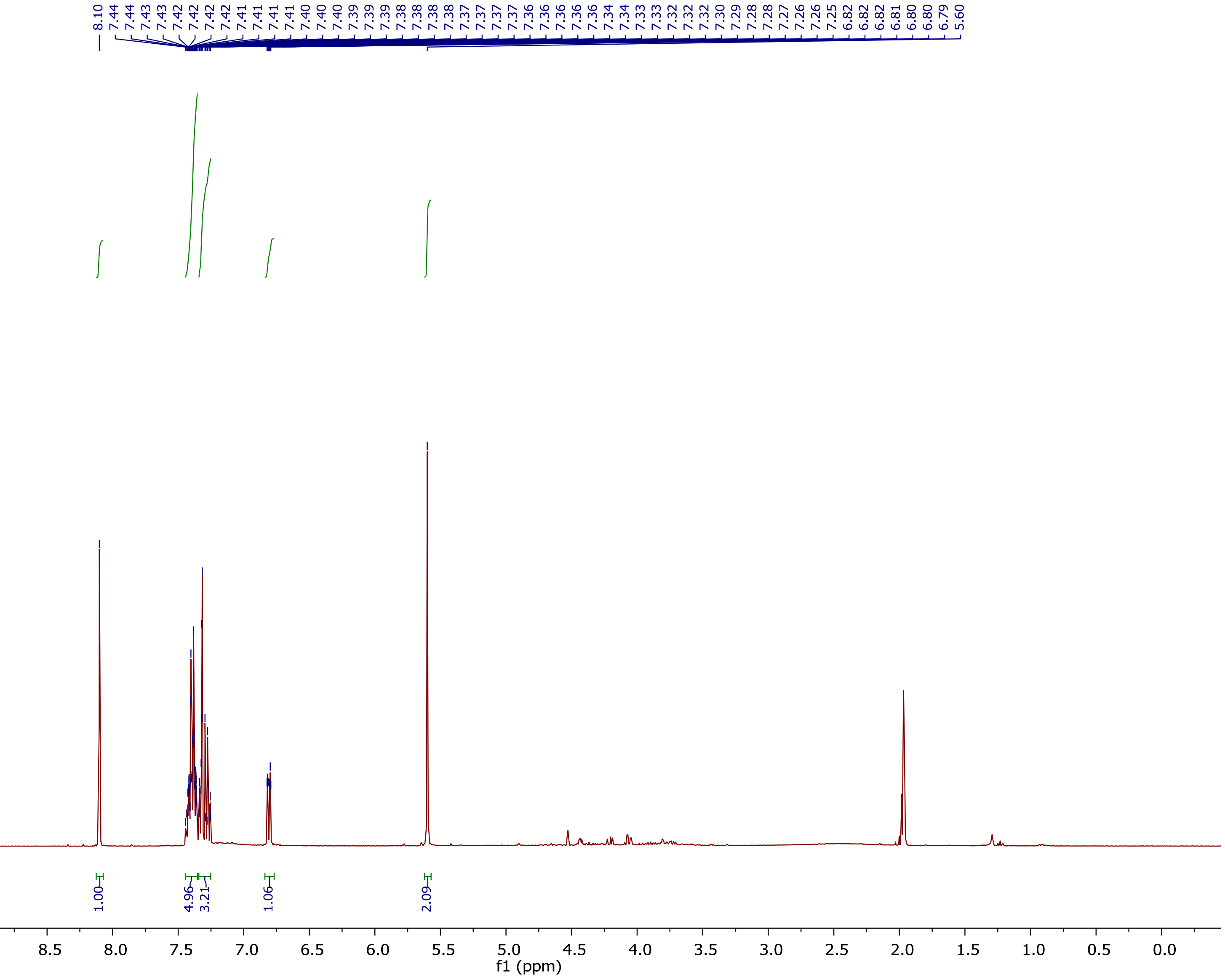

CARBON_01
SLC:WB3-110

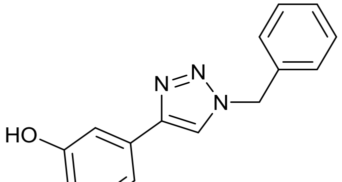

1b

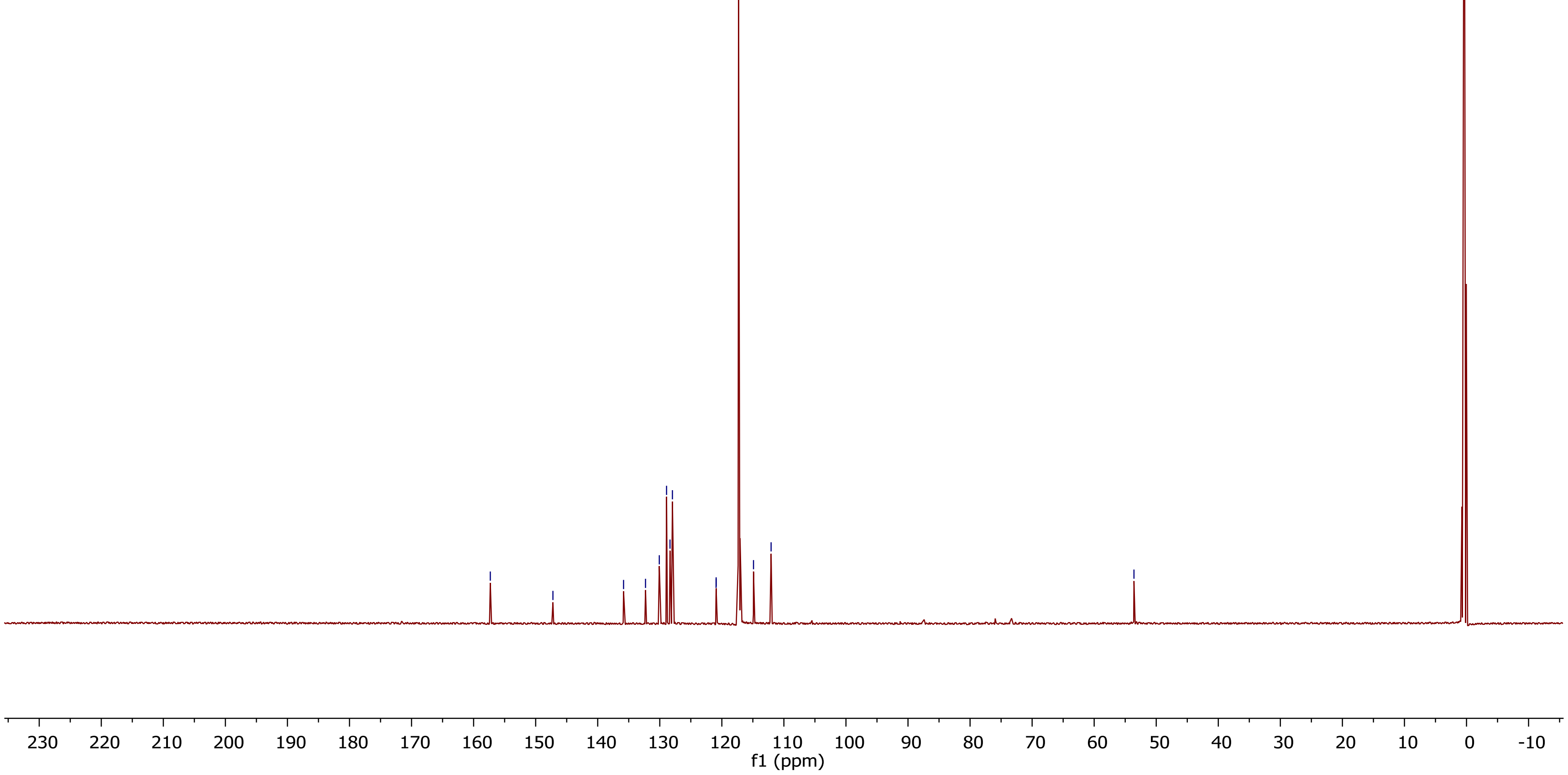

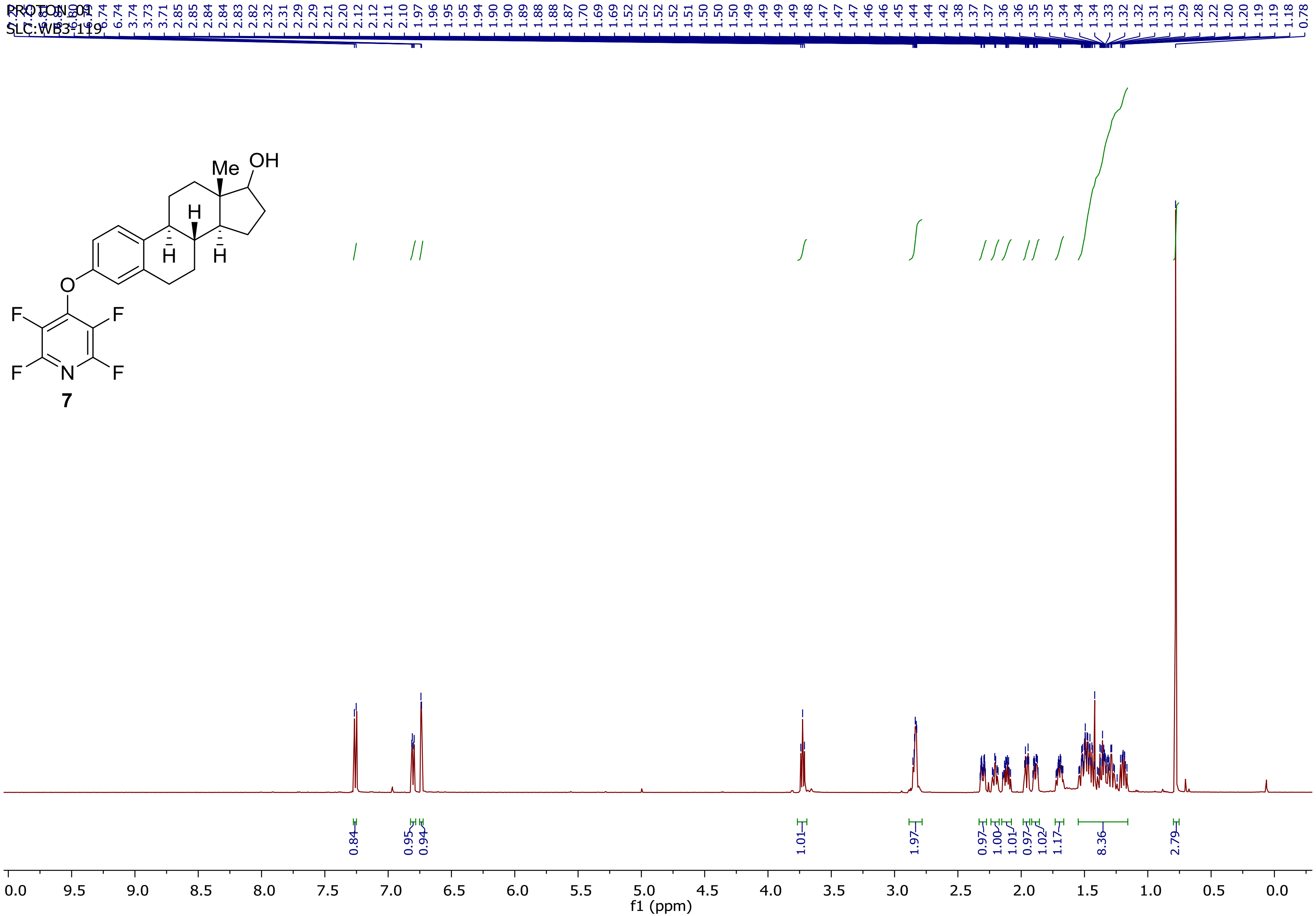
PROTON_01
SLC:WB3-119
6.74 6.74 3.74 3.73 3.71 2.85 2.85 2.84 2.84 2.83 2.82 2.32 2.31 2.29 2.29 2.21 2.20 2.12 2.12 2.11 2.10 1.97 1.96 1.95 1.95 1.94 1.90 1.90 1.89 1.88 1.88 1.87 1.70 1.69 1.69 1.52 1.52 1.52 1.52 1.51 1.50 1.50 1.50 1.49 1.49 1.49 1.49 1.48 1.47 1.47 1.47 1.46 1.46 1.45 1.44 1.44 1.42 1.38 1.37 1.37 1.36 1.36 1.35 1.35 1.34 1.34 1.34 1.33 1.33 1.32 1.32 1.31 1.31 1.29 1.28 1.22 1.20 1.20 1.19 1.19 1.18 0.78
Me
OH
H
H
H
O
F
F
F
N
F
7
0.84
0.95
0.94
1.01
1.97
0.97
1.00
1.01
0.97
1.02
1.17
8.36
2.79
0.0
9.5
9.0
8.5
8.0
7.5
7.0
6.5
6.0
5.5
5.0
4.5
4.0
3.5
3.0
2.5
2.0
1.5
1.0
0.5
0.0
f1 (ppm)

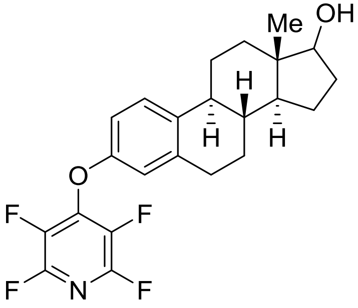

7

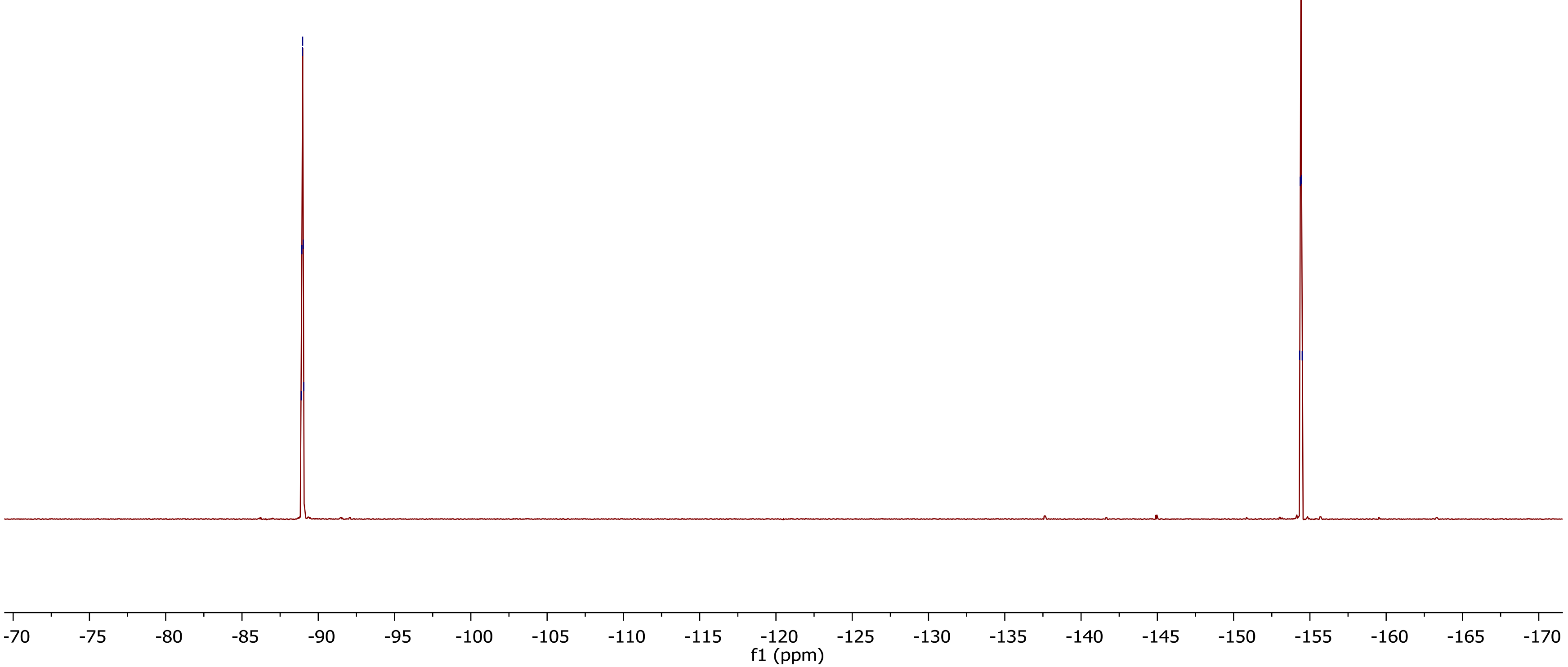
24142808.13.fid
SLC:WDGB:WB3-119
-88.88
-88.92
-88.96
-88.98
-89.01
-89.05
-154.33
-154.37
-154.40
-154.42
-154.46
-154.50
-70 -75 -80 -85 -90 -95 -100 -105 -110 -115 -120 -125 -130 -135 -140 -145 -150 -155 -160 -165 -170
f1 (ppm)

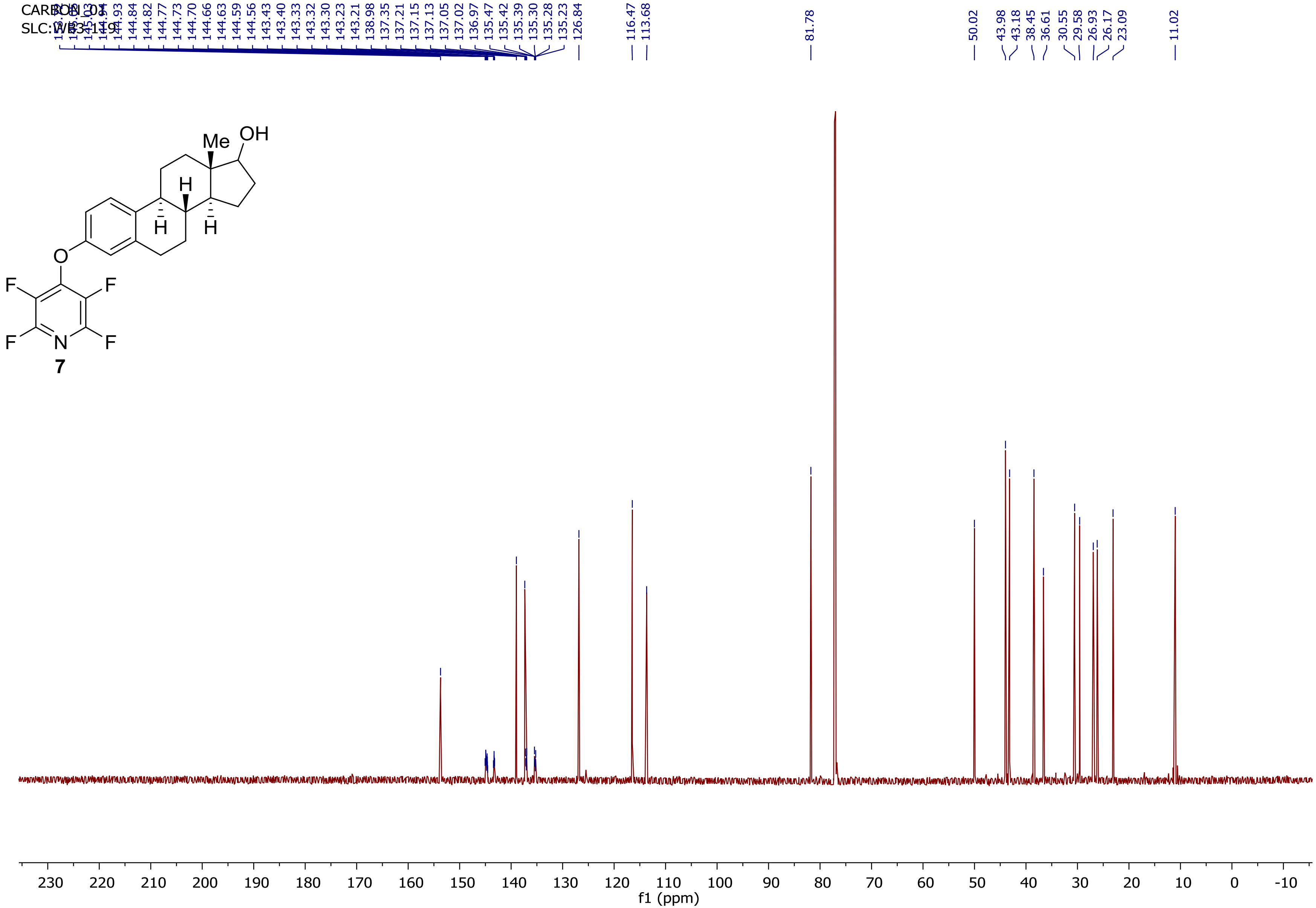

CARBON_01
SLC:WB3-119
145.03
144.94
144.93
144.84
144.82
144.77
144.73
144.70
144.66
144.63
144.59
144.56
143.43
143.40
143.33
143.32
143.30
143.23
143.21
138.98
137.35
137.21
137.15
137.13
137.05
137.02
136.97
135.47
135.42
135.39
135.30
135.28
135.23
126.84
116.47
113.68
81.78
50.02
43.98
43.18
38.45
36.61
30.55
29.58
26.93
26.17
23.09
11.02
230 220 210 200 190 180 170 160 150 140 130 120 110 100 90 80 70 60 50 40 30 20 10 0 -10
f1 (ppm)
Me
OH
H
H
H
O
F
F
F
N
F

7

Deprotection Reactions Monitored by NMR

General Procedure for TFP Ether Cleavage monitored by ^{1}H NMR

To a solution of TFP Ether (0.022 mmol, 1 equiv.) in CD_3CN (0.7 mL) was added KF (0.044 mmol, 2 equiv.), 18-C-6 (0.066 mmol, 3 equiv.) and methyl thioglycolate (0.22 mmol, 10 equiv.) and finally D_2O (0.1 mL). The reaction mixture was gently heated until all components were dissolved. The resulting mixture was transferred to a NMR tube and suspended in a water bath at 50 °C for 1 h. After this time the tube was removed from the water bath and a ^{1}H NMR spectrum of the reaction mixture recorded. In cases where full conversion was not reached after 1 h the NMR tube was returned to the water bath and heated until full conversion was reached.

Table *S1*

Table 1: Deprotection condition screening

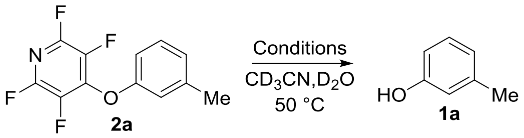

Entry	Conditions	Time/h	Conversion/%
1	KI (2 equiv.)	24	-
2	KBr (2 equiv.)	24	-
3	KCl (2 equiv.)	24	-
4	KF (2 equiv.)	24	<1
5	KI (2 equiv.), 18-C-6 (3 equiv.)	24	<1
6	KF (2 equiv.), 18-C-6 (3 equiv.)	24	1
7	NaI (2 equiv.), 18-C-6 (3 equiv.)	24	<1
8	KF (2 equiv.), 18-C-6 (3 equiv.), methyl thioglycolate (10 equiv.)	1	78
9	KF (2 equiv.), methyl thioglycolate (10 equiv.)	1	61
10	KF (1 equiv.), 18-C-6 (1 equiv.), methyl thioglycolate (2 equiv.)	1	57
11	Methyl thioglycolate (10 equiv.)	24	<1
12	methyl thioglycolate (10 equiv.). K_2CO_3 (2 equiv.)	24	90

Conversion was determined by ^{1}H NMR analysis of the reaction mixture (see SI). All reactions were carried out in a water bath set to 50 °C.

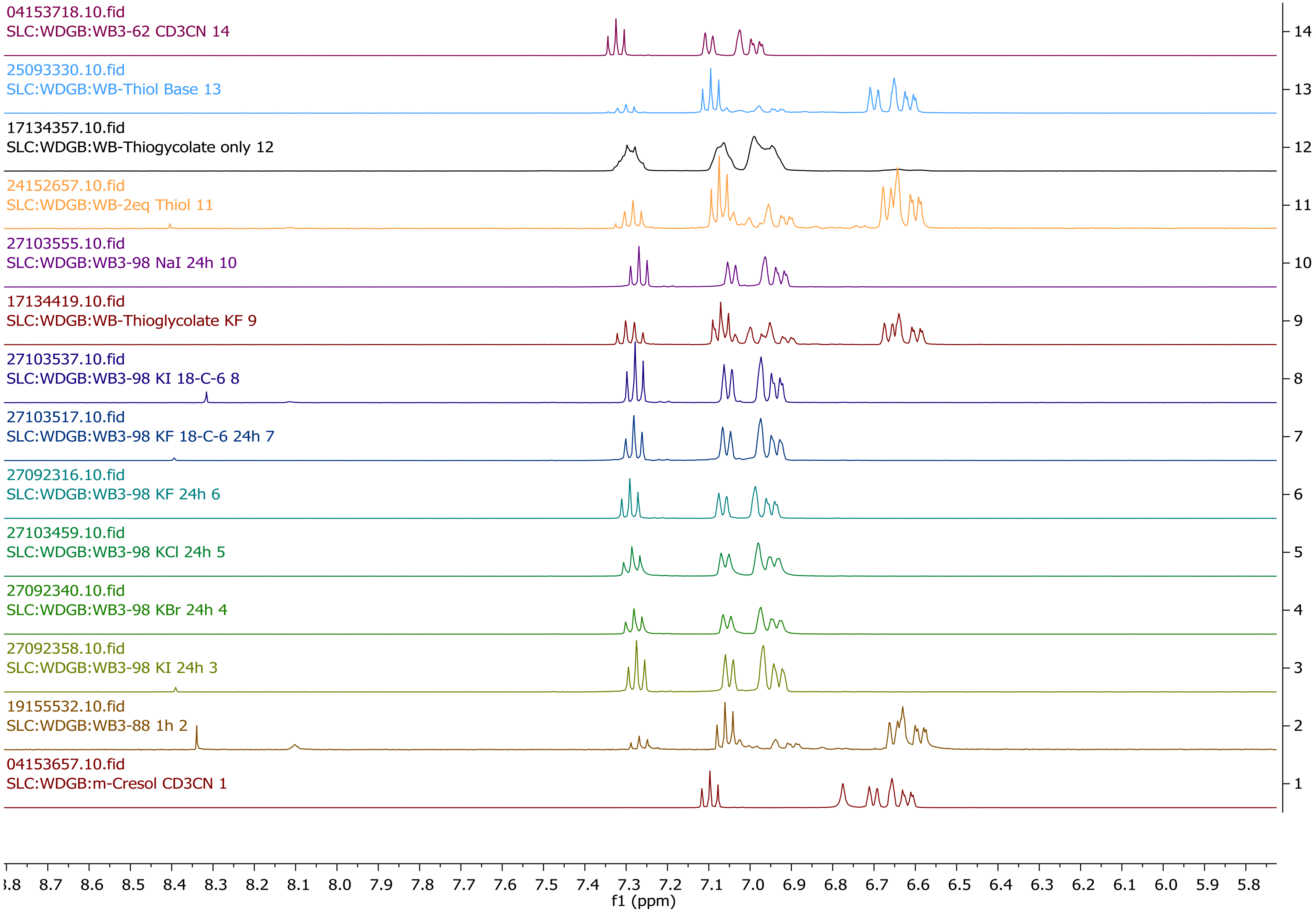

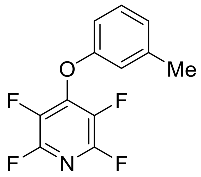

Reference spectrum in CD_3CN

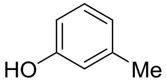

Reference spectrum in CD_3CN

General Procedure for stability testing using ^1H NMR

To a solution of TFP ether (10 mg) in deuterated solvent (0.7 mL) was added the desired reagent to be tested. The reaction mixture was transferred to an NMR tube and the reaction mixture left at rt for 24 h. After this time the reaction mixture was analysed by ^1H NMR spectroscopy.

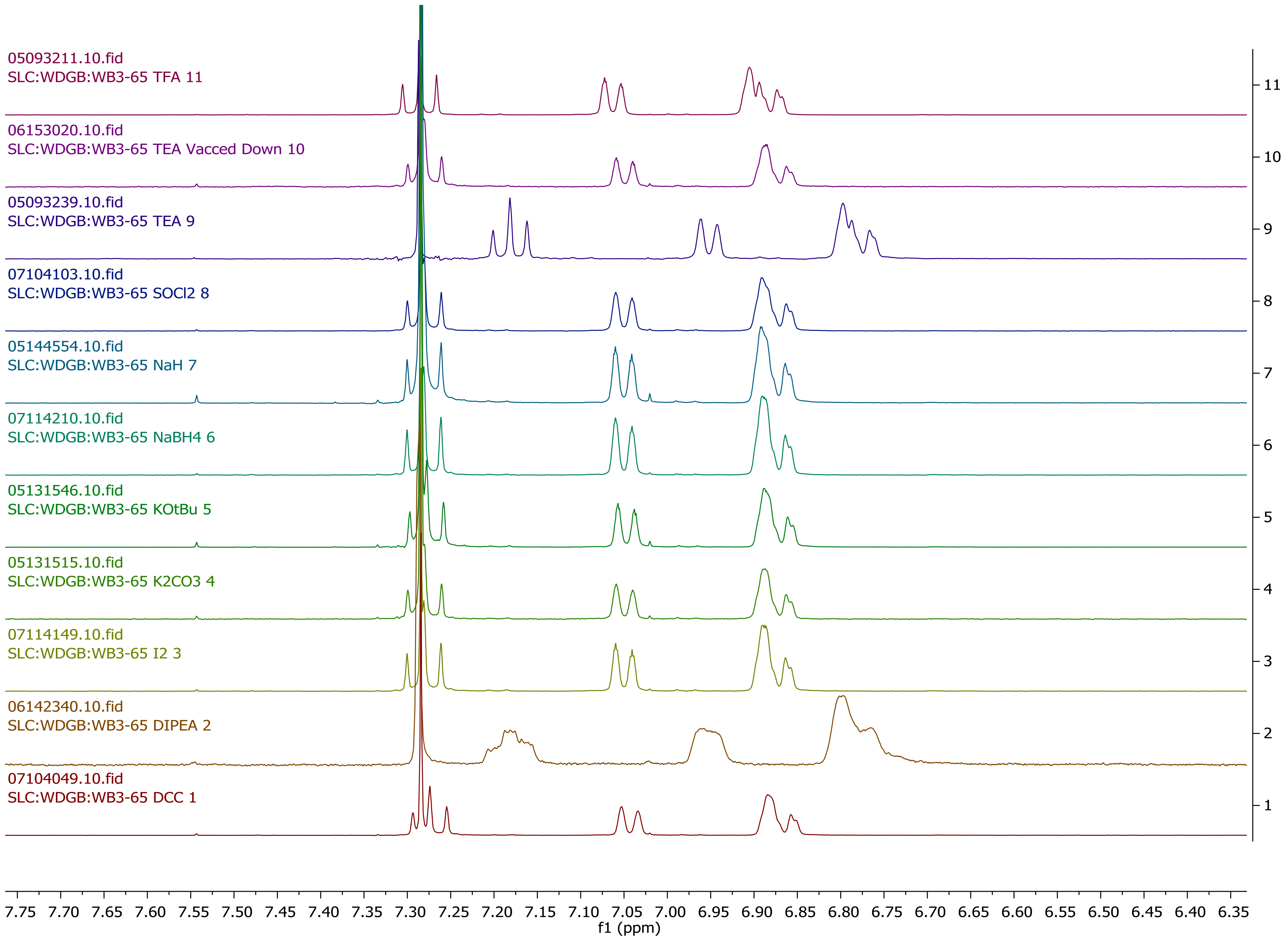

05093211.10.fid
SLC:WDGB:WB3-65 TFA 11
06153020.10.fid
SLC:WDGB:WB3-65 TEA Vacced Down 10
05093239.10.fid
SLC:WDGB:WB3-65 TEA 9
07104103.10.fid
SLC:WDGB:WB3-65 SOCl2 8
05144554.10.fid
SLC:WDGB:WB3-65 NaH 7
07114210.10.fid
SLC:WDGB:WB3-65 NaBH4 6
05131546.10.fid
SLC:WDGB:WB3-65 KOtBu 5
05131515.10.fid
SLC:WDGB:WB3-65 K2CO3 4
07114149.10.fid
SLC:WDGB:WB3-65 I2 3
06142340.10.fid
SLC:WDGB:WB3-65 DIPEA 2
07104049.10.fid
SLC:WDGB:WB3-65 DCC 1
7.75 7.70 7.65 7.60 7.55 7.50 7.45 7.40 7.35 7.30 7.25 7.20 7.15 7.10 7.05 7.00 6.95 6.90 6.85 6.80 6.75 6.70 6.65 6.60 6.55 6.50 6.45 6.40 6.35
f1 (ppm)
11 10 9 8 7 6 5 4 3 2 1

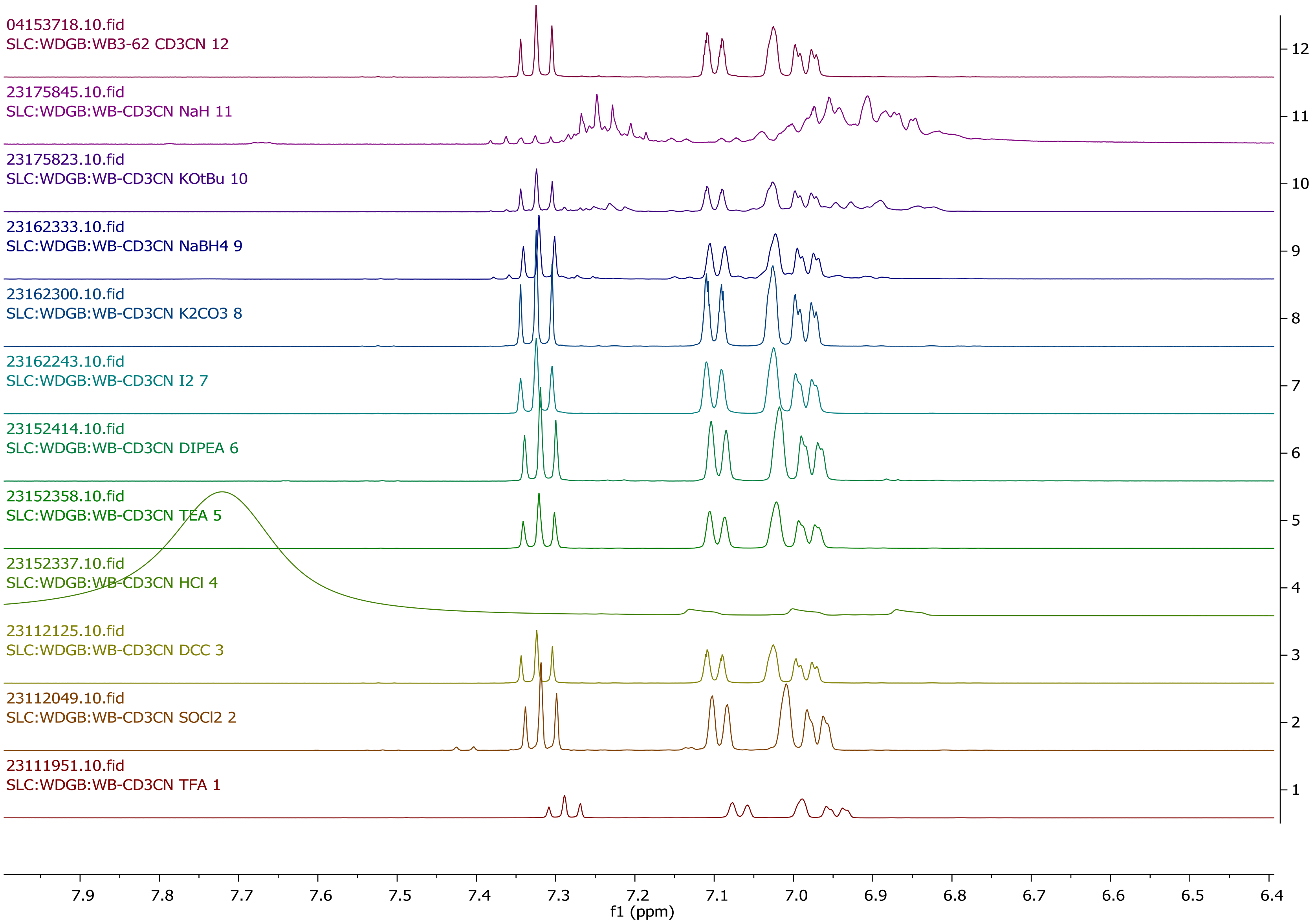
04153718.10.fid
SLC:WDGB:WB3-62 CD3CN 12
23175845.10.fid
SLC:WDGB:WB-CD3CN NaH 11
23175823.10.fid
SLC:WDGB:WB-CD3CN KOtBu 10
23162333.10.fid
SLC:WDGB:WB-CD3CN NaBH4 9
23162300.10.fid
SLC:WDGB:WB-CD3CN K2CO3 8
23162243.10.fid
SLC:WDGB:WB-CD3CN I2 7
23152414.10.fid
SLC:WDGB:WB-CD3CN DIPEA 6
23152358.10.fid
SLC:WDGB:WB-CD3CN TEA 5
23152337.10.fid
SLC:WDGB:WB-CD3CN HCl 4
23112125.10.fid
SLC:WDGB:WB-CD3CN DCC 3
23112049.10.fid
SLC:WDGB:WB-CD3CN SOCl2 2
23111951.10.fid
SLC:WDGB:WB-CD3CN TFA 1
7.9
7.8
7.7
7.6
7.5
7.4
7.3
7.2
7.1
7.0
6.9
6.8
6.7
6.6
6.5
6.4
f1 (ppm)

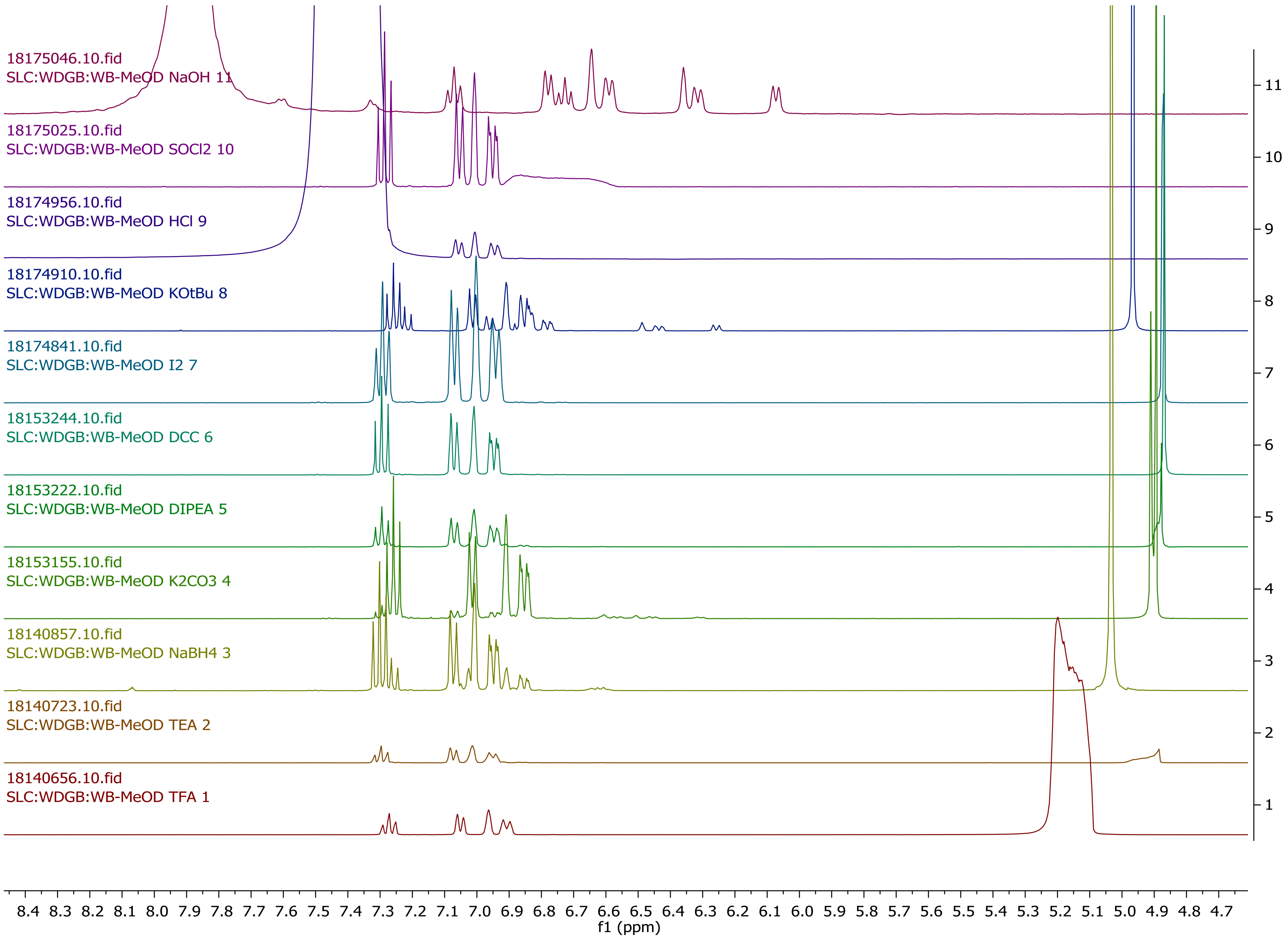
18175046.10.fid
SLC:WDGB:WB-MeOD NaOH 11
18175025.10.fid
SLC:WDGB:WB-MeOD SOCl2 10
18174956.10.fid
SLC:WDGB:WB-MeOD HCl 9
18174910.10.fid
SLC:WDGB:WB-MeOD KOtBu 8
18174841.10.fid
SLC:WDGB:WB-MeOD I2 7
18153244.10.fid
SLC:WDGB:WB-MeOD DCC 6
18153222.10.fid
SLC:WDGB:WB-MeOD DIPEA 5
18153155.10.fid
SLC:WDGB:WB-MeOD K2CO3 4
18140857.10.fid
SLC:WDGB:WB-MeOD NaBH4 3
18140723.10.fid
SLC:WDGB:WB-MeOD TEA 2
18140656.10.fid
SLC:WDGB:WB-MeOD TFA 1
8.4 8.3 8.2 8.1 8.0 7.9 7.8 7.7 7.6 7.5 7.4 7.3 7.2 7.1 7.0 6.9 6.8 6.7 6.6 6.5 6.4 6.3 6.2 6.1 6.0 5.9 5.8 5.7 5.6 5.5 5.4 5.3 5.2 5.1 5.0 4.9 4.8 4.7
f1 (ppm)
1 2 3 4 5 6 7 8 9 10 11

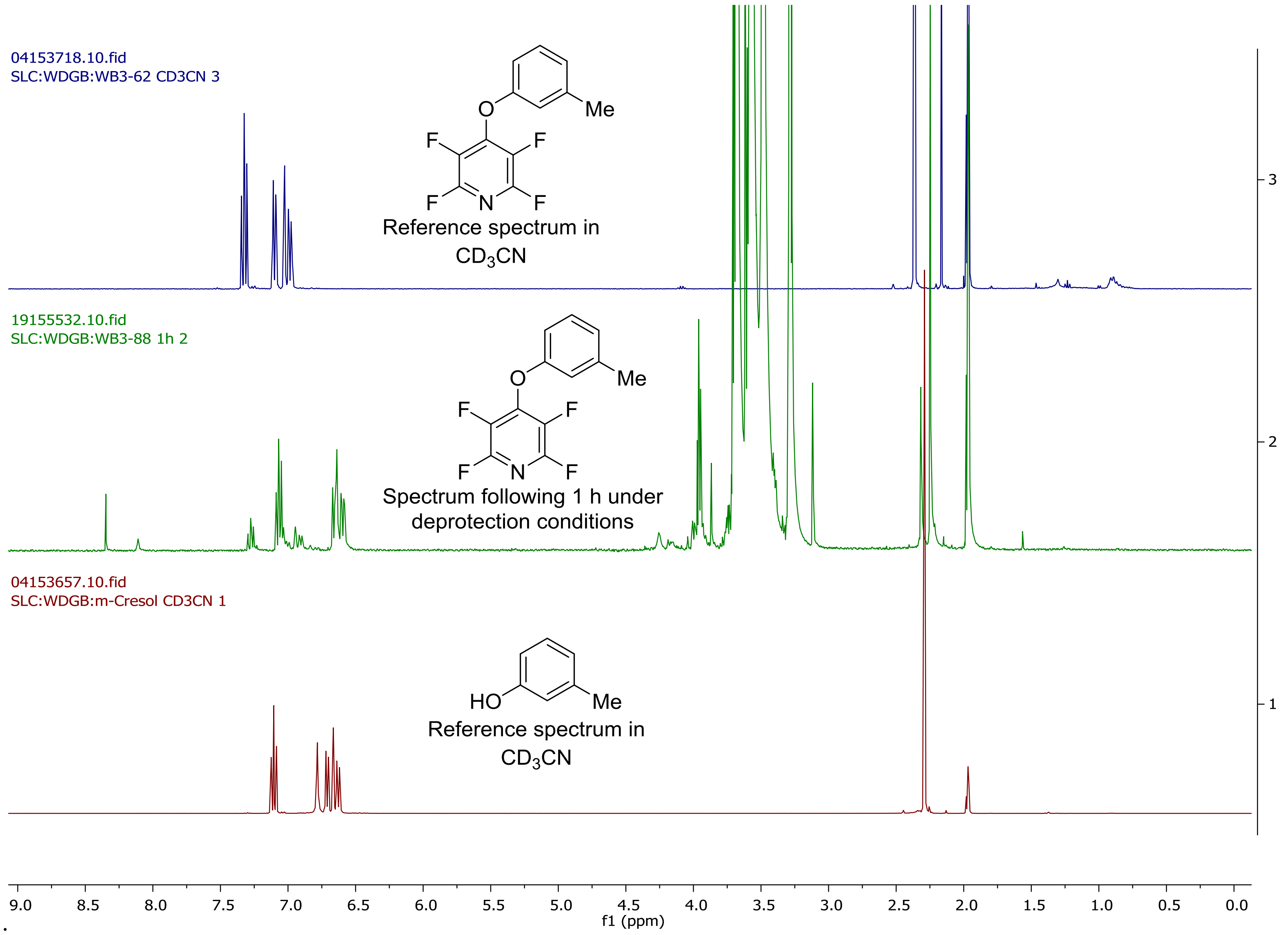
04153718.10.fid
SLC:WDGB:WB3-62 CD3CN 3
Me
O
F
F
F
N
F
Reference spectrum in
CD3CN
19155532.10.fid
SLC:WDGB:WB3-88 1h 2
Me
O
F
F
F
N
F
Spectrum following 1 h under
deprotection conditions
04153657.10.fid
SLC:WDGB:m-Cresol CD3CN 1
HO
Me
Reference spectrum in
CD3CN
3
2
1
9.0
8.5
8.0
7.5
7.0
6.5
6.0
5.5
5.0
4.5
4.0
3.5
3.0
2.5
2.0
1.5
1.0
0.5
0.0
f1 (ppm)

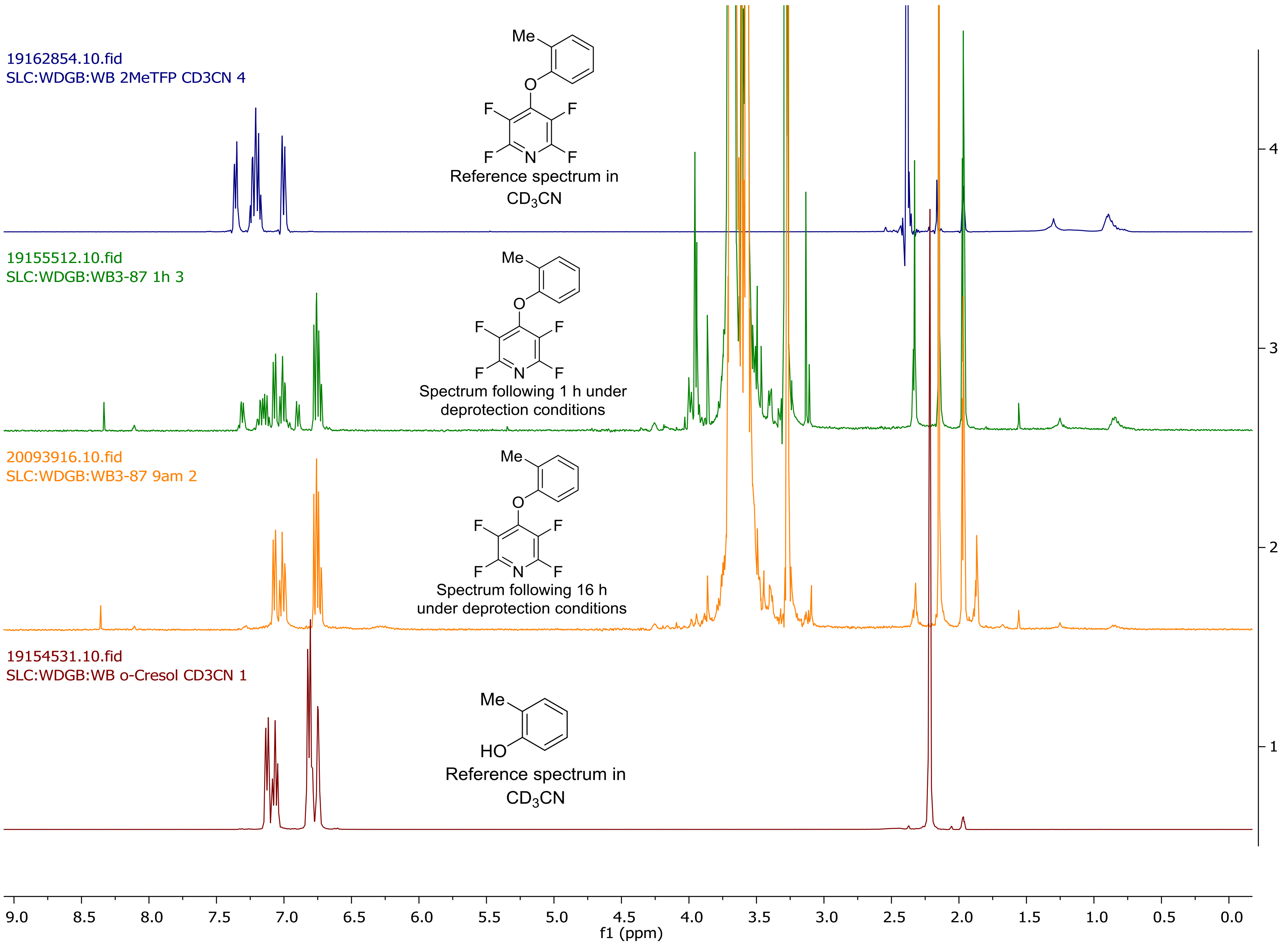
19162854.10.fid
SLC:WDGB:WB 2MeTFP CD3CN 4
Reference spectrum in CD3CN
19155512.10.fid
SLC:WDGB:WB3-87 1h 3
Spectrum following 1 h under deprotection conditions
20093916.10.fid
SLC:WDGB:WB3-87 9am 2
Spectrum following 16 h under deprotection conditions
19154531.10.fid
SLC:WDGB:WB o-Cresol CD3CN 1
Reference spectrum in CD3CN
f1 (ppm)

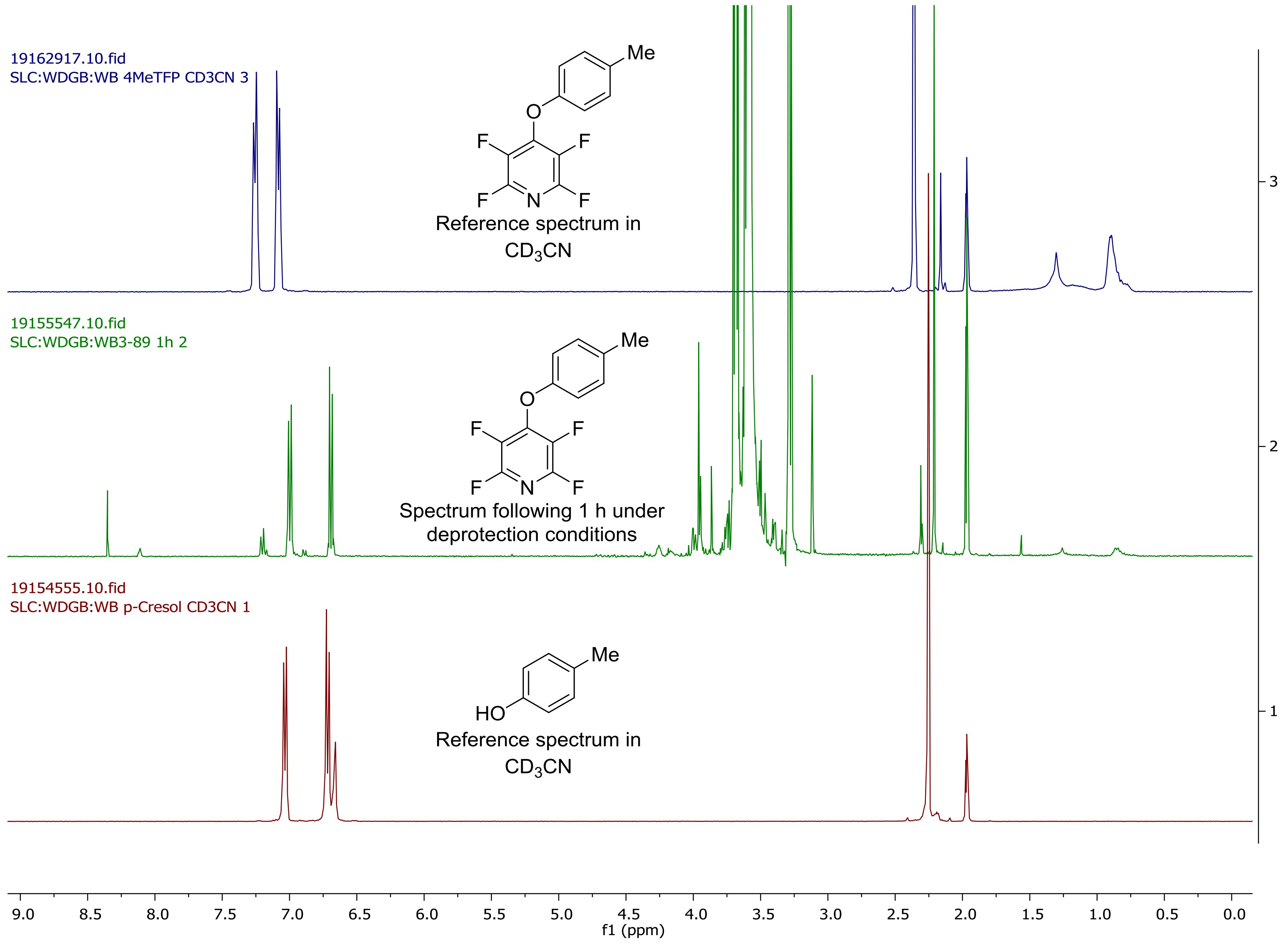
19162917.10.fid
SLC:WDGB:WB 4MeTFP CD3CN 3
Me
O
F
F
F
N
F
Reference spectrum in CD_3CN
19155547.10.fid
SLC:WDGB:WB3-89 1h 2
Spectrum following 1 h under deprotection conditions
19154555.10.fid
SLC:WDGB:WB p-Cresol CD3CN 1
HO
Reference spectrum in CD_3CN
9.0 8.5 8.0 7.5 7.0 6.5 6.0 5.5 5.0 4.5 4.0 3.5 3.0 2.5 2.0 1.5 1.0 0.5 0.0
f1 (ppm)
3
2
1

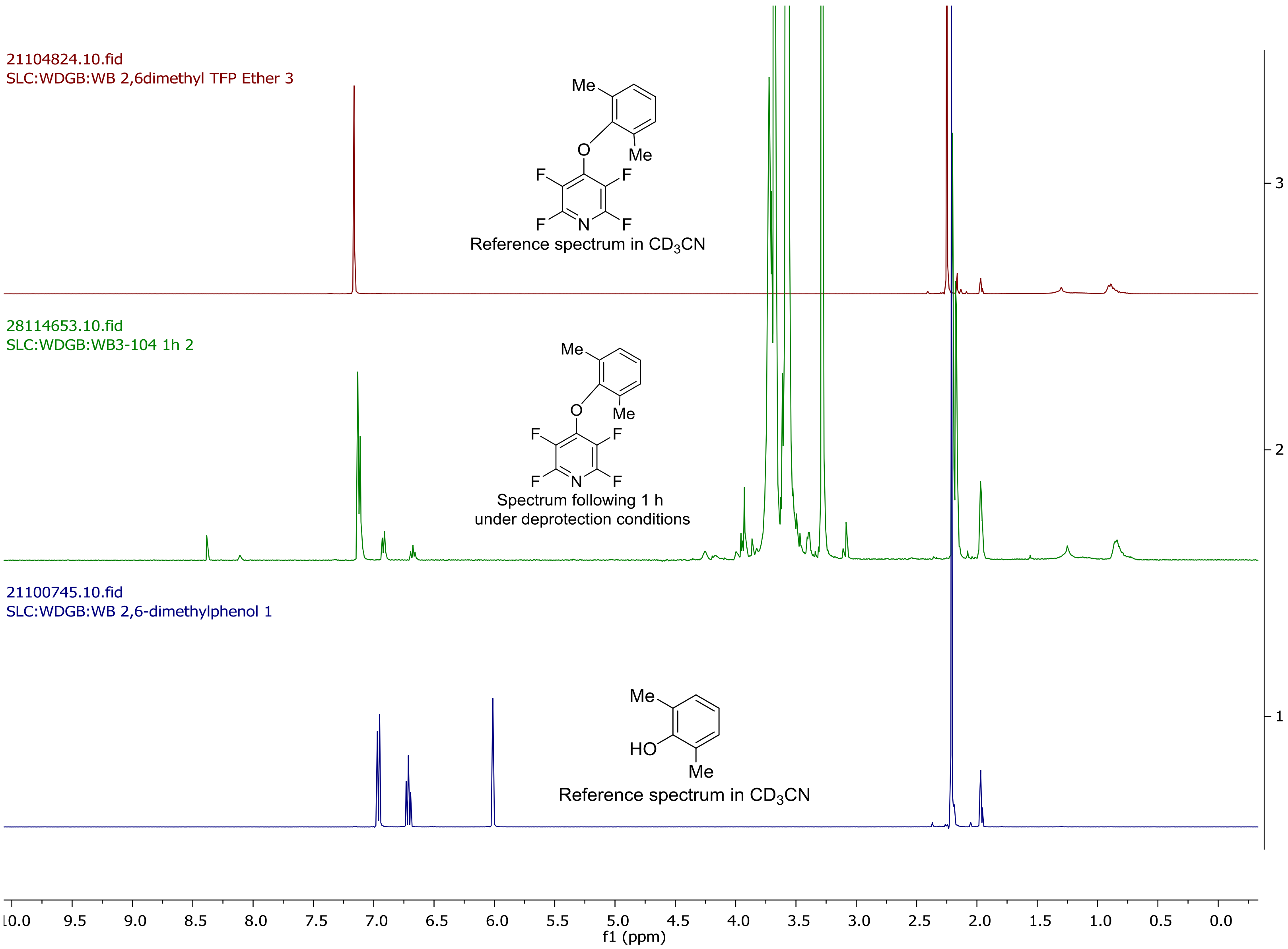
21104824.10.fid
SLC:WDGB:WB 2,6dimethyl TFP Ether 3
Me
O
Me
F
F
F
N
F
Reference spectrum in CD_3CN
28114653.10.fid
SLC:WDGB:WB3-104 1h 2
Spectrum following 1 h
under deprotection conditions
21100745.10.fid
SLC:WDGB:WB 2,6-dimethylphenol 1
HO
Reference spectrum in CD_3CN
3
2
1
10.0 9.5 9.0 8.5 8.0 7.5 7.0 6.5 6.0 5.5 5.0 4.5 4.0 3.5 3.0 2.5 2.0 1.5 1.0 0.5 0.0
f1 (ppm)

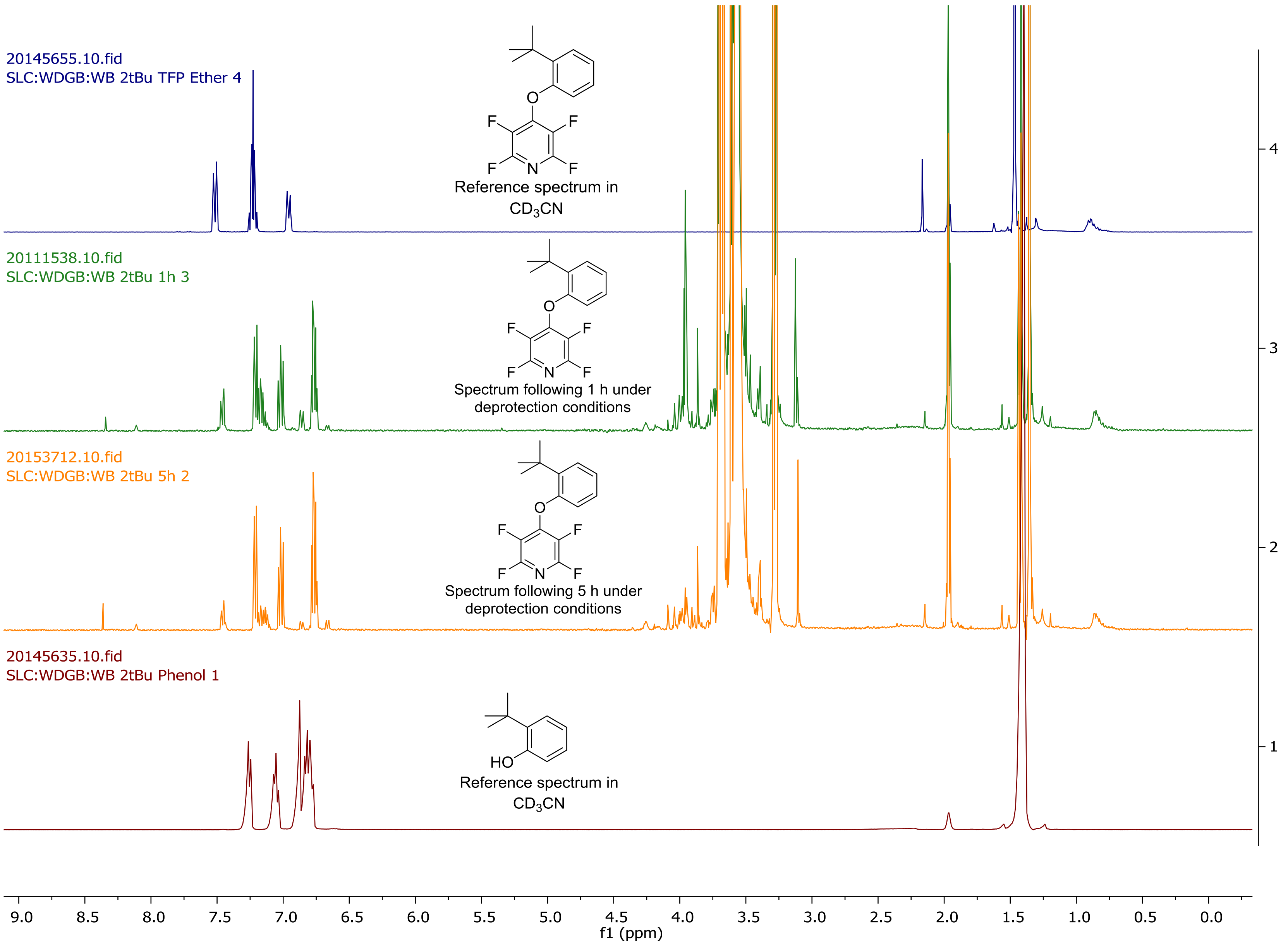
20145655.10.fid
SLC:WDGB:WB 2tBu TFP Ether 4
Reference spectrum in
CD_3CN
20111538.10.fid
SLC:WDGB:WB 2tBu 1h 3
Spectrum following 1 h under deprotection conditions
20153712.10.fid
SLC:WDGB:WB 2tBu 5h 2
Spectrum following 5 h under deprotection conditions
20145635.10.fid
SLC:WDGB:WB 2tBu Phenol 1
Reference spectrum in
CD_3CN
f1 (ppm)

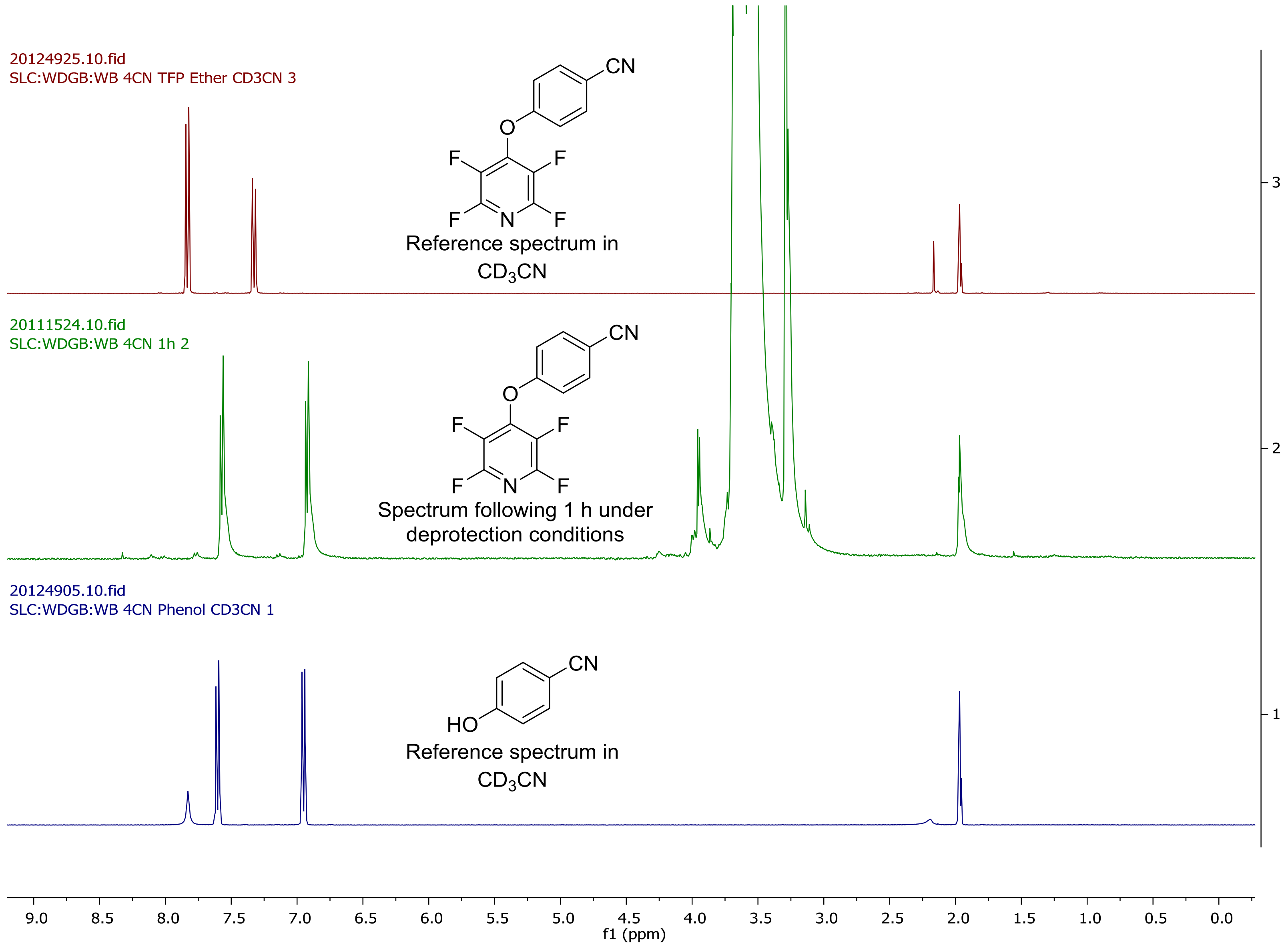
20124925.10.fid
SLC:WDGB:WB 4CN TFP Ether CD3CN 3
Reference spectrum in CD3CN
20111524.10.fid
SLC:WDGB:WB 4CN 1h 2
Spectrum following 1 h under deprotection conditions
20124905.10.fid
SLC:WDGB:WB 4CN Phenol CD3CN 1
Reference spectrum in CD3CN
f1 (ppm)

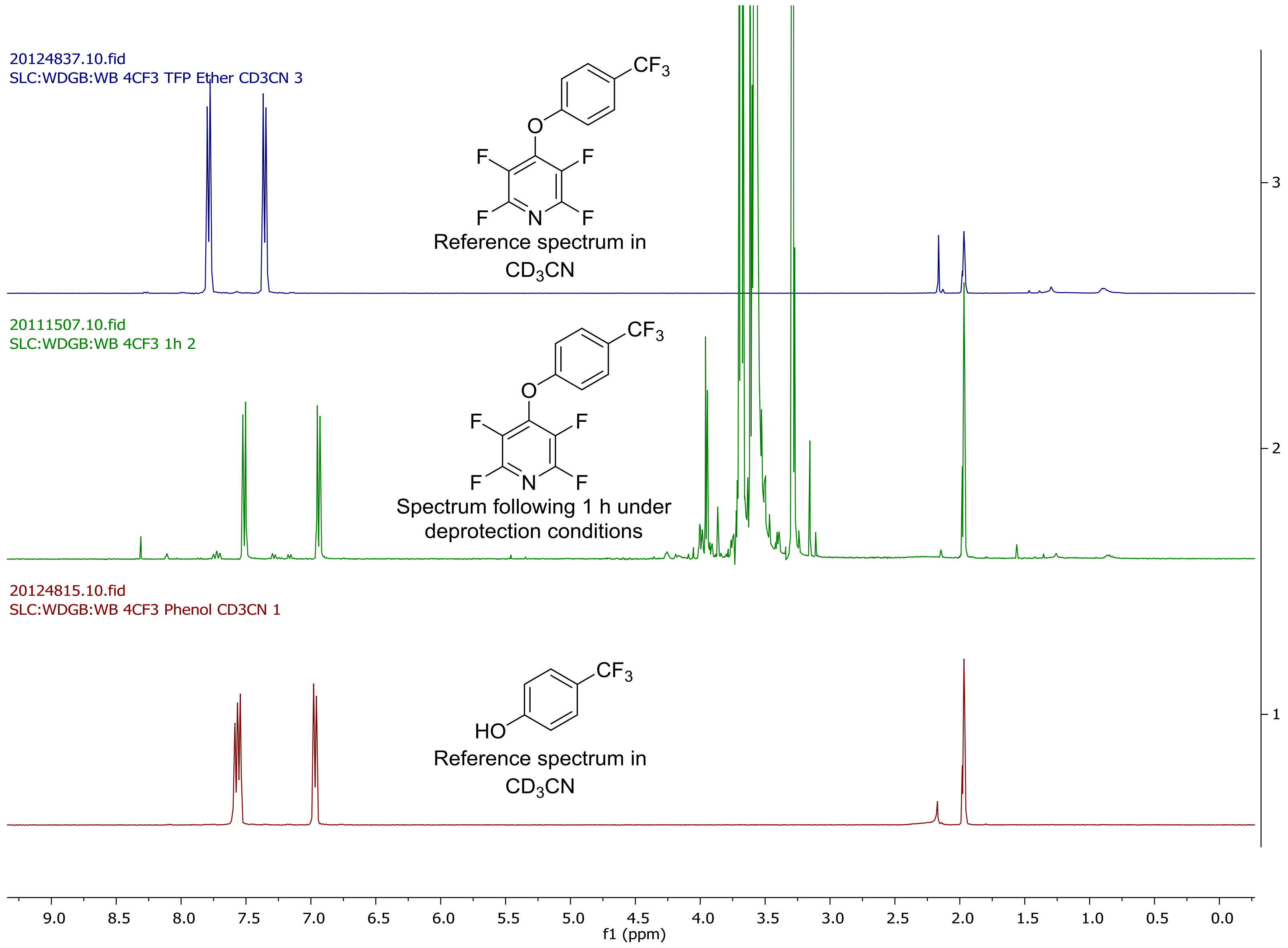
20124837.10.fid
SLC:WDGB:WB 4CF3 TFP Ether CD3CN 3
Reference spectrum in CD3CN
20111507.10.fid
SLC:WDGB:WB 4CF3 1h 2
Spectrum following 1 h under deprotection conditions
20124815.10.fid
SLC:WDGB:WB 4CF3 Phenol CD3CN 1
Reference spectrum in CD3CN
f1 (ppm)

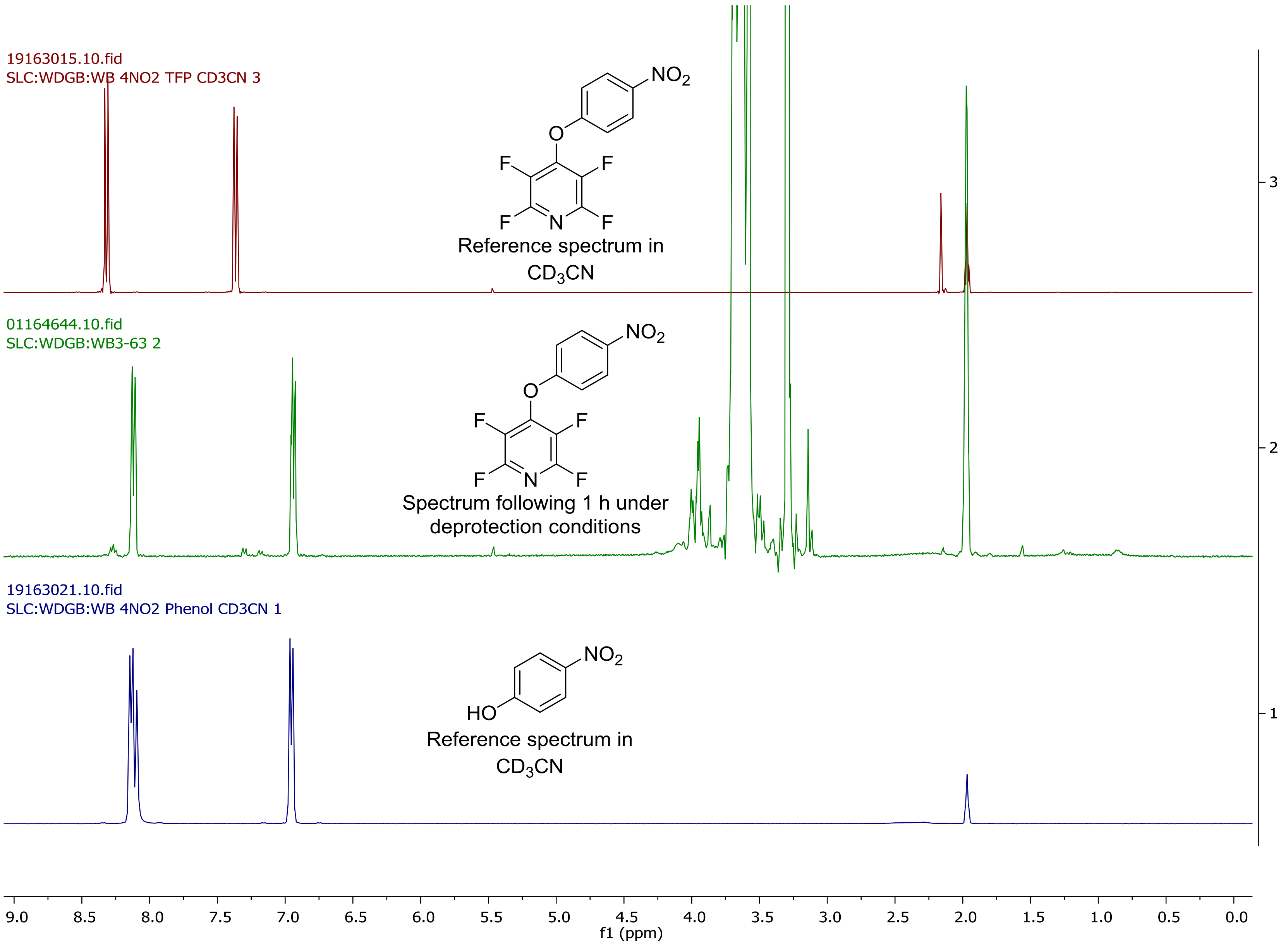
19163015.10.fid
SLC:WDGB:WB 4NO2 TFP CD3CN 3
Reference spectrum in
CD3CN
01164644.10.fid
SLC:WDGB:WB3-63 2
Spectrum following 1 h under
deprotection conditions
19163021.10.fid
SLC:WDGB:WB 4NO2 Phenol CD3CN 1
Reference spectrum in
CD3CN
f1 (ppm)

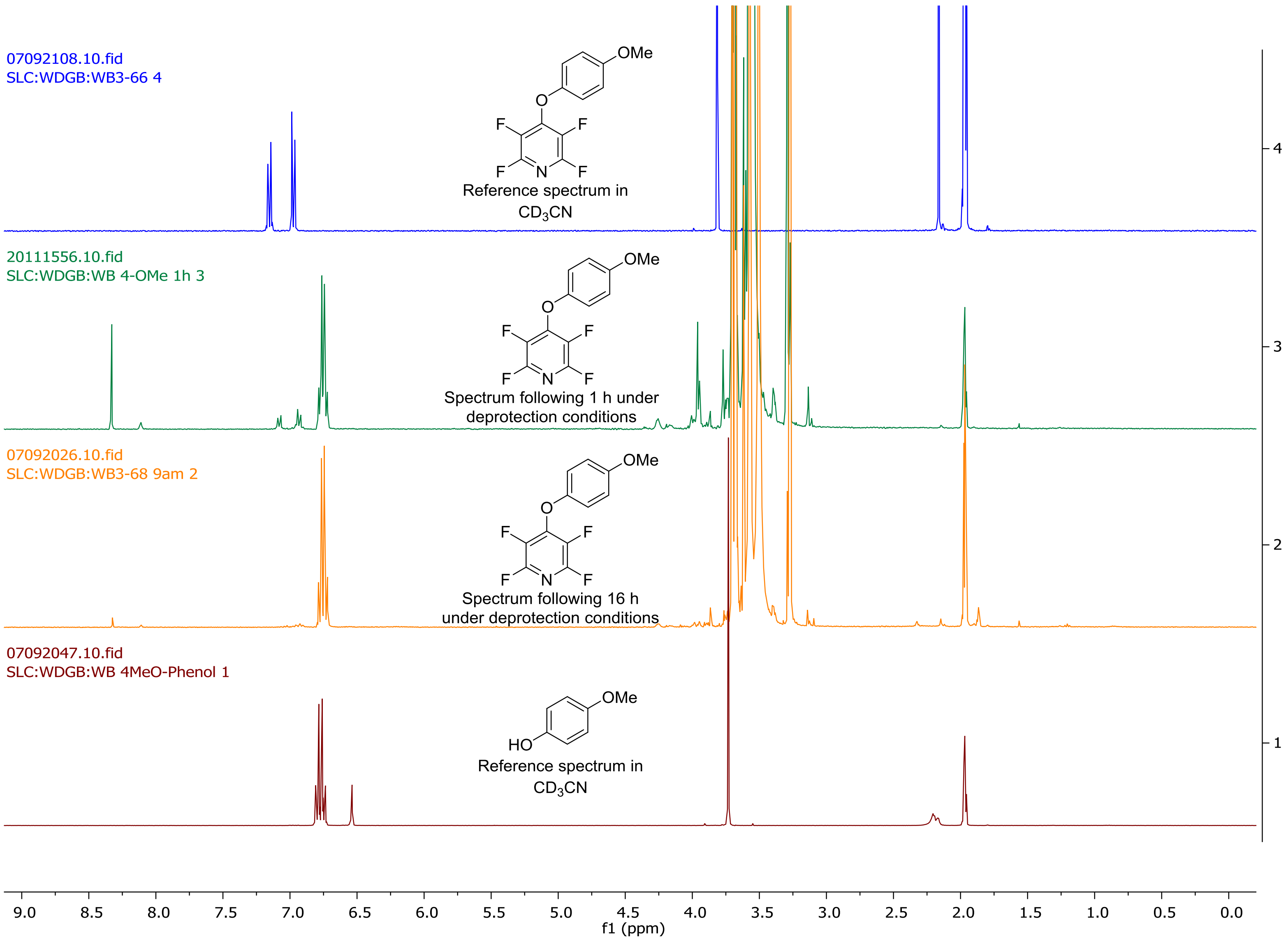
07092108.10.fid
SLC:WDGB:WB3-66 4
OMe
O
F
F
F
N
F
Reference spectrum in
CD3CN
20111556.10.fid
SLC:WDGB:WB 4-OMe 1h 3
Spectrum following 1 h under
deprotection conditions
07092026.10.fid
SLC:WDGB:WB3-68 9am 2
Spectrum following 16 h
under deprotection conditions
07092047.10.fid
SLC:WDGB:WB 4MeO-Phenol 1
HO
Reference spectrum in
CD3CN
f1 (ppm)

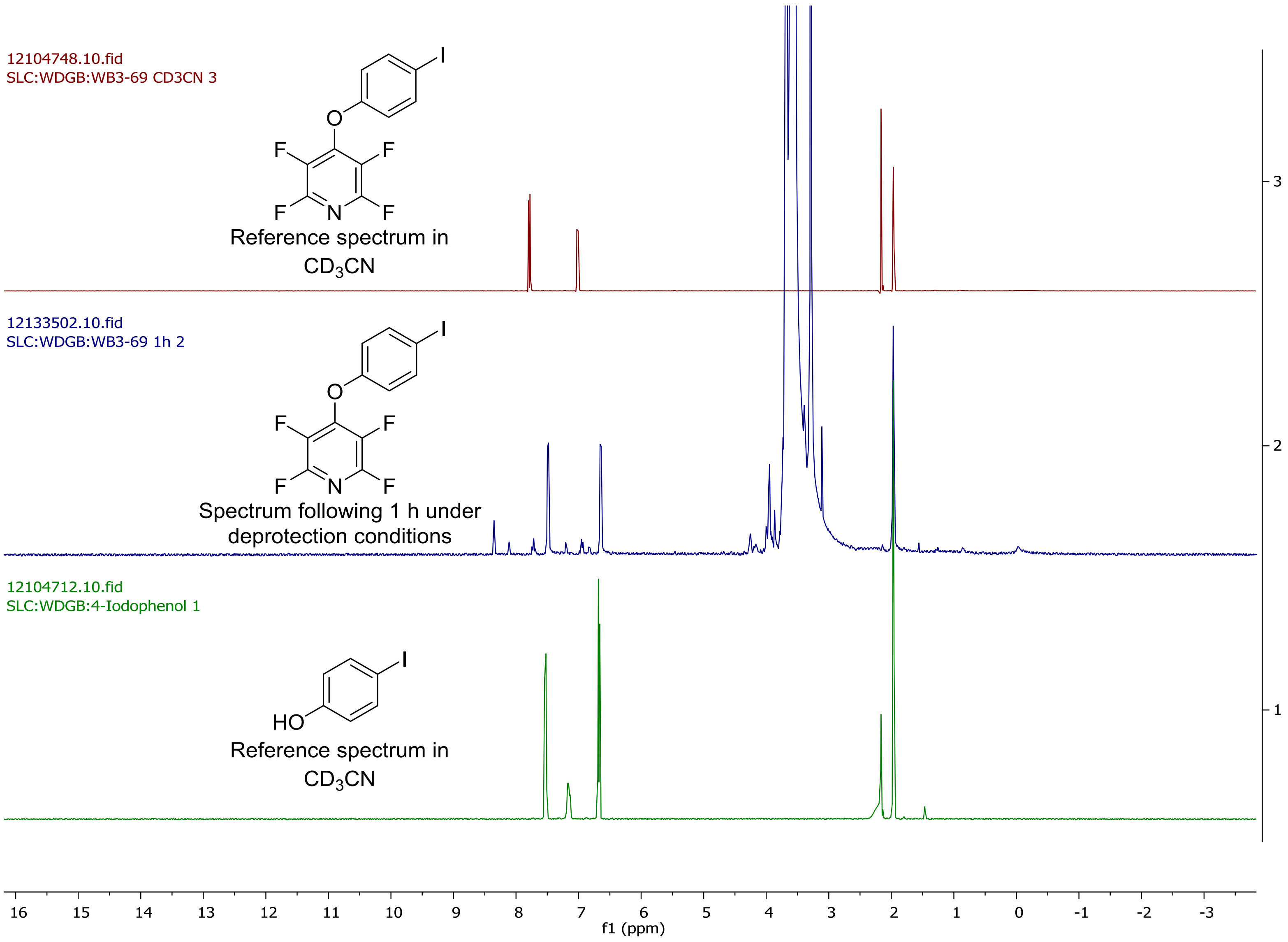
12104748.10.fid
SLC:WDGB:WB3-69 CD3CN 3
Reference spectrum in CD3CN
12133502.10.fid
SLC:WDGB:WB3-69 1h 2
Spectrum following 1 h under deprotection conditions
12104712.10.fid
SLC:WDGB:4-Iodophenol 1
Reference spectrum in CD3CN
f1 (ppm)

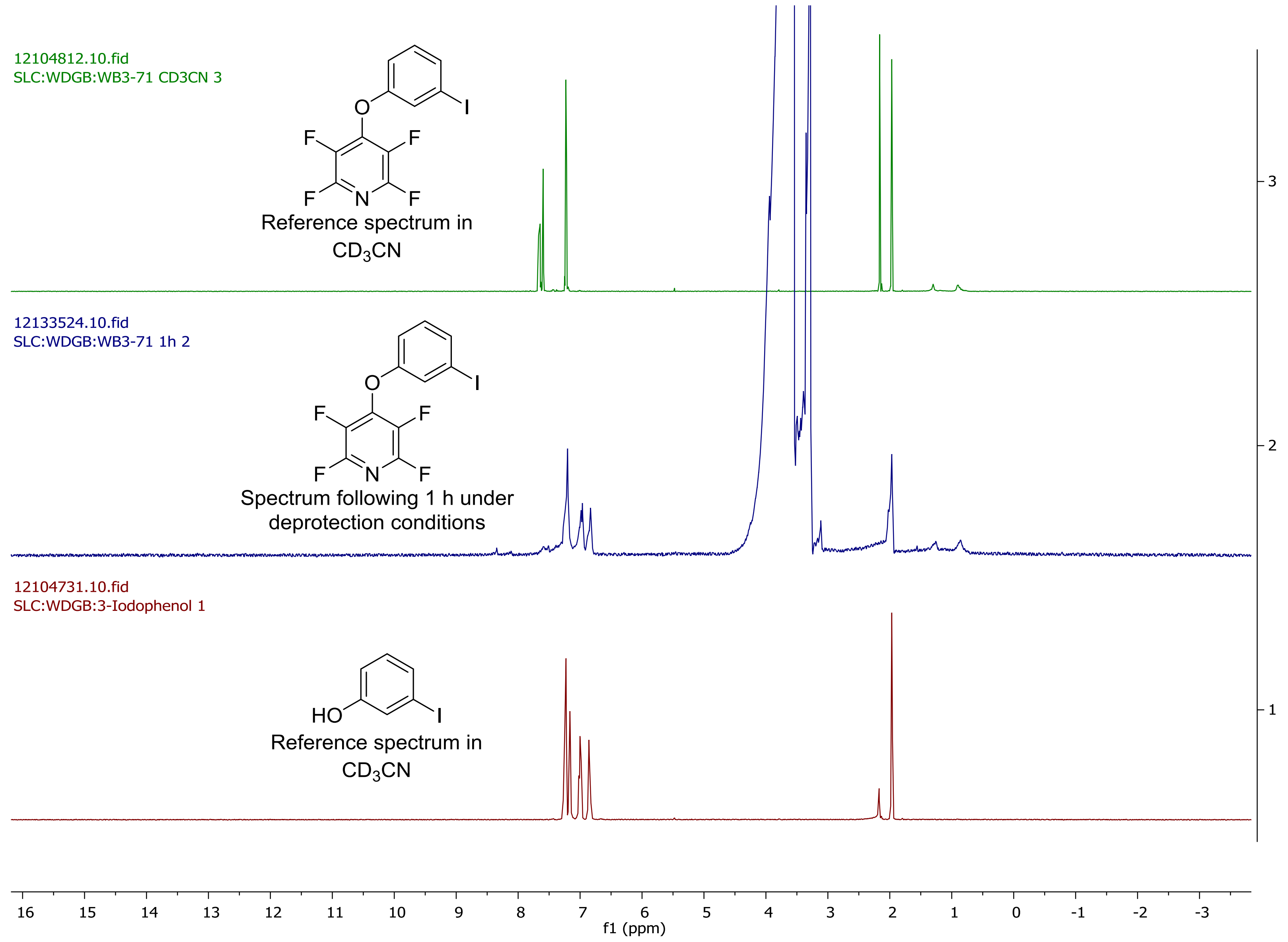
12104812.10.fid
SLC:WDGB:WB3-71 CD3CN 3
Reference spectrum in CD3CN
12133524.10.fid
SLC:WDGB:WB3-71 1h 2
Spectrum following 1 h under deprotection conditions
12104731.10.fid
SLC:WDGB:3-Iodophenol 1
HO
Reference spectrum in CD3CN
f1 (ppm)

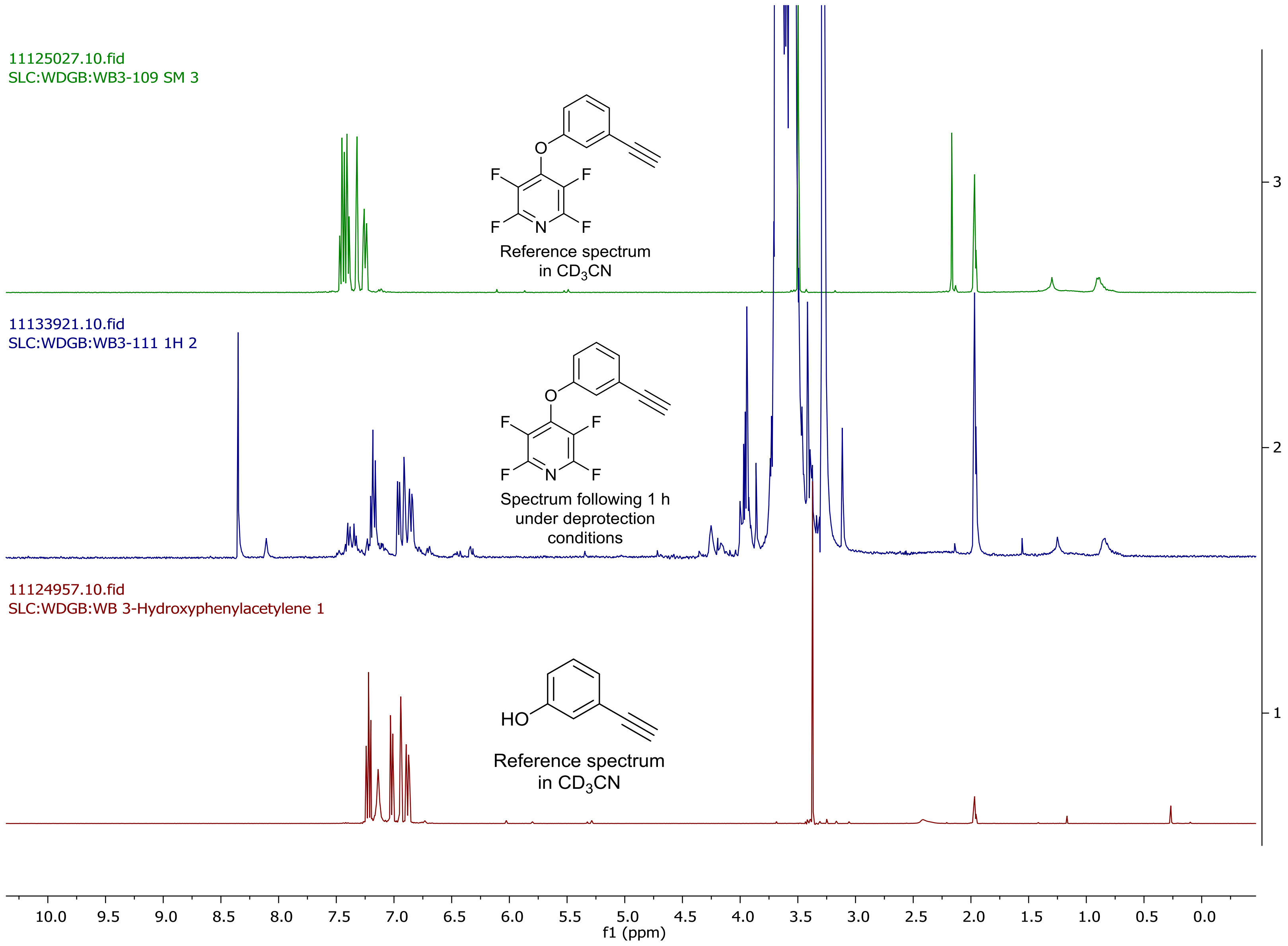

11125027.10.fid
SLC:WDGB:WB3-109 SM 3
Reference spectrum
in CD3CN
11133921.10.fid
SLC:WDGB:WB3-111 1H 2
Spectrum following 1 h
under deprotection
conditions
11124957.10.fid
SLC:WDGB:WB 3-Hydroxyphenylacetylene 1
HO
Reference spectrum
in CD3CN
f1 (ppm)

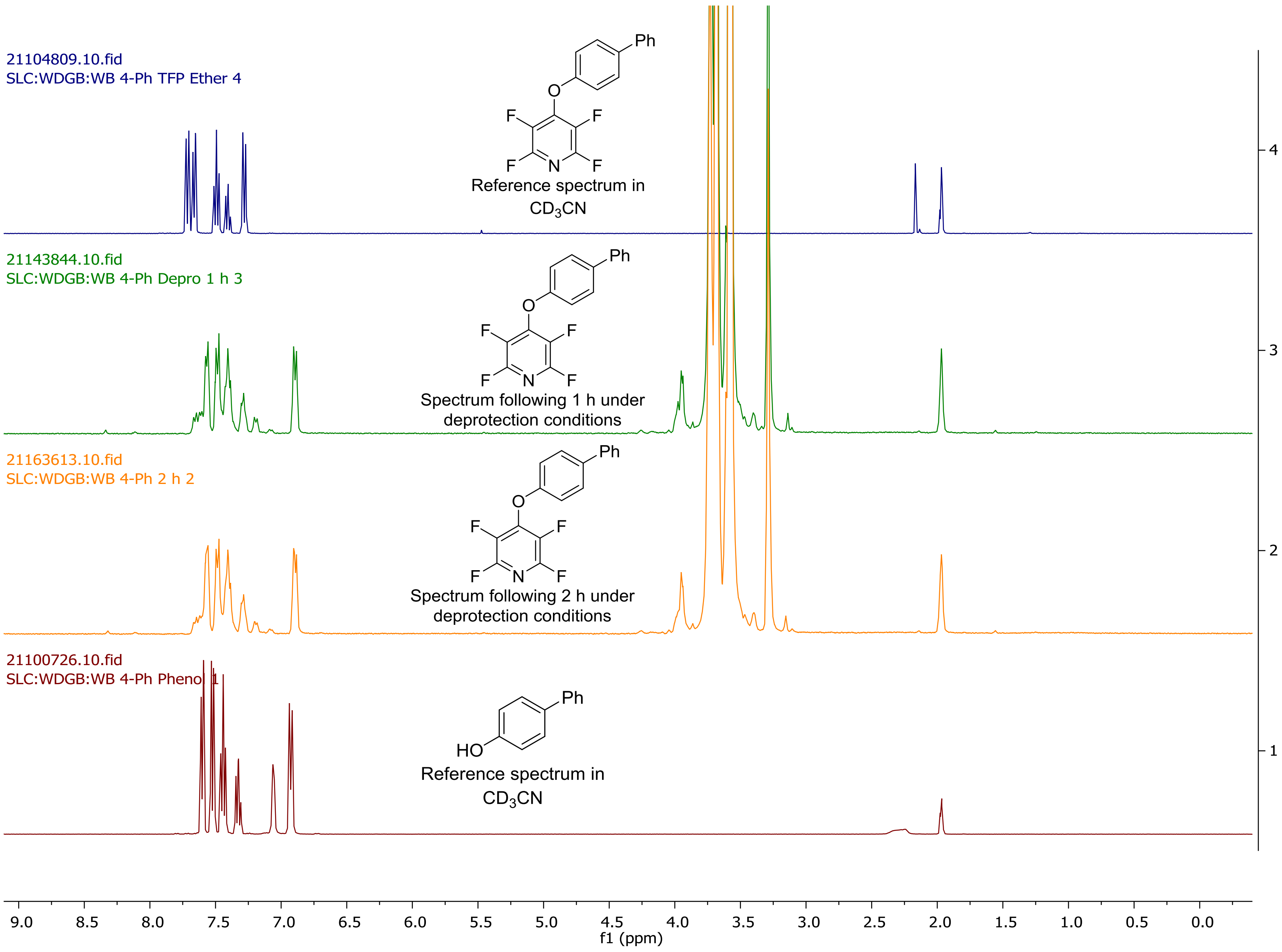

21104809.10.fid
SLC:WDGB:WB 4-Ph TFP Ether 4
Reference spectrum in CD3CN
21143844.10.fid
SLC:WDGB:WB 4-Ph Depro 1 h 3
Spectrum following 1 h under deprotection conditions
21163613.10.fid
SLC:WDGB:WB 4-Ph 2 h 2
Spectrum following 2 h under deprotection conditions
21100726.10.fid
SLC:WDGB:WB 4-Ph Phenol 1
Reference spectrum in CD3CN
f1 (ppm)

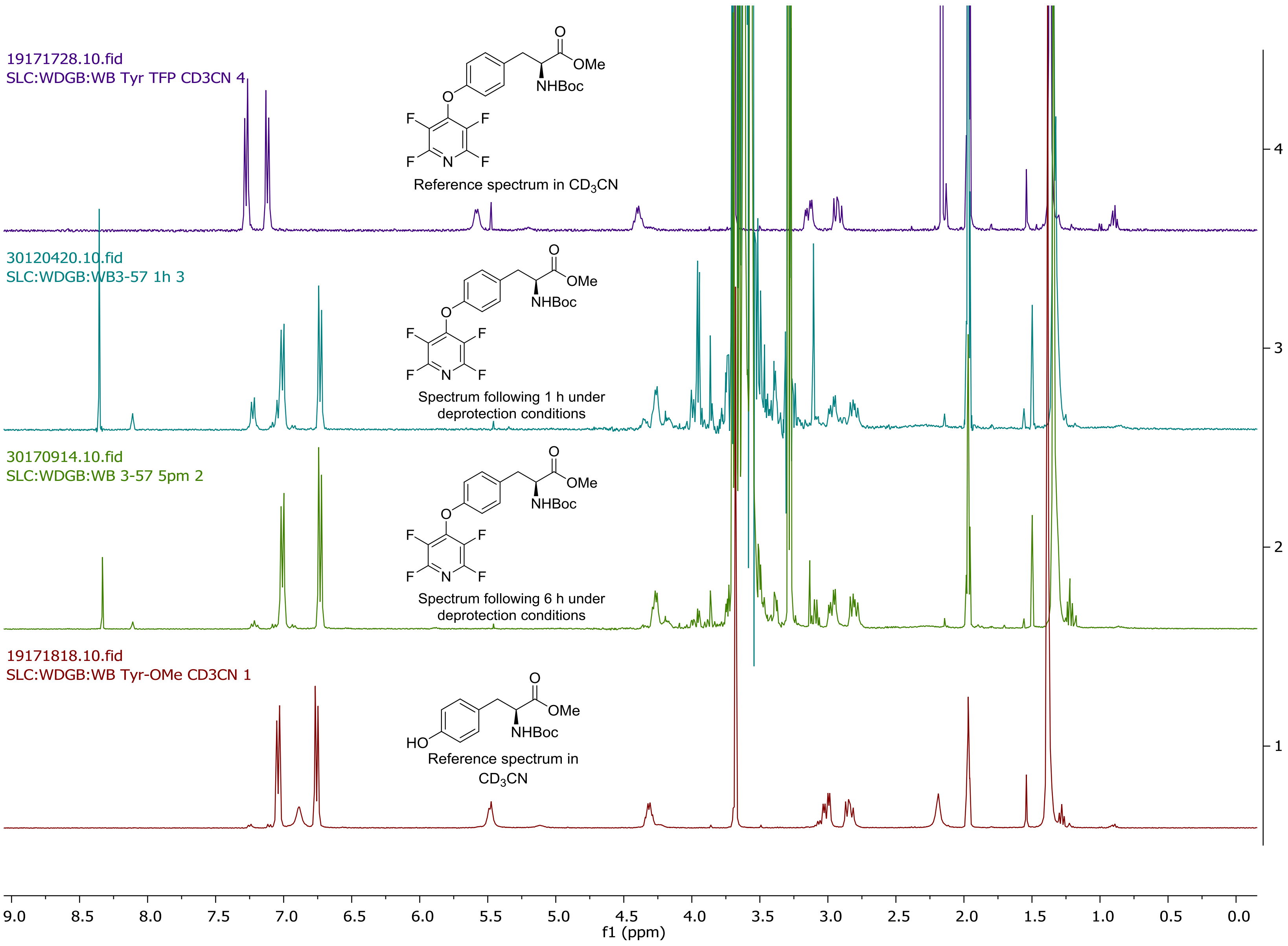

19171728.10.fid
SLC:WDGB:WB Tyr TFP CD3CN 4
Reference spectrum in CD_3CN
30120420.10.fid
SLC:WDGB:WB3-57 1h 3
Spectrum following 1 h under deprotection conditions
30170914.10.fid
SLC:WDGB:WB 3-57 5pm 2
Spectrum following 6 h under deprotection conditions
19171818.10.fid
SLC:WDGB:WB Tyr-OMe CD3CN 1
Reference spectrum in CD_3CN
f1 (ppm)

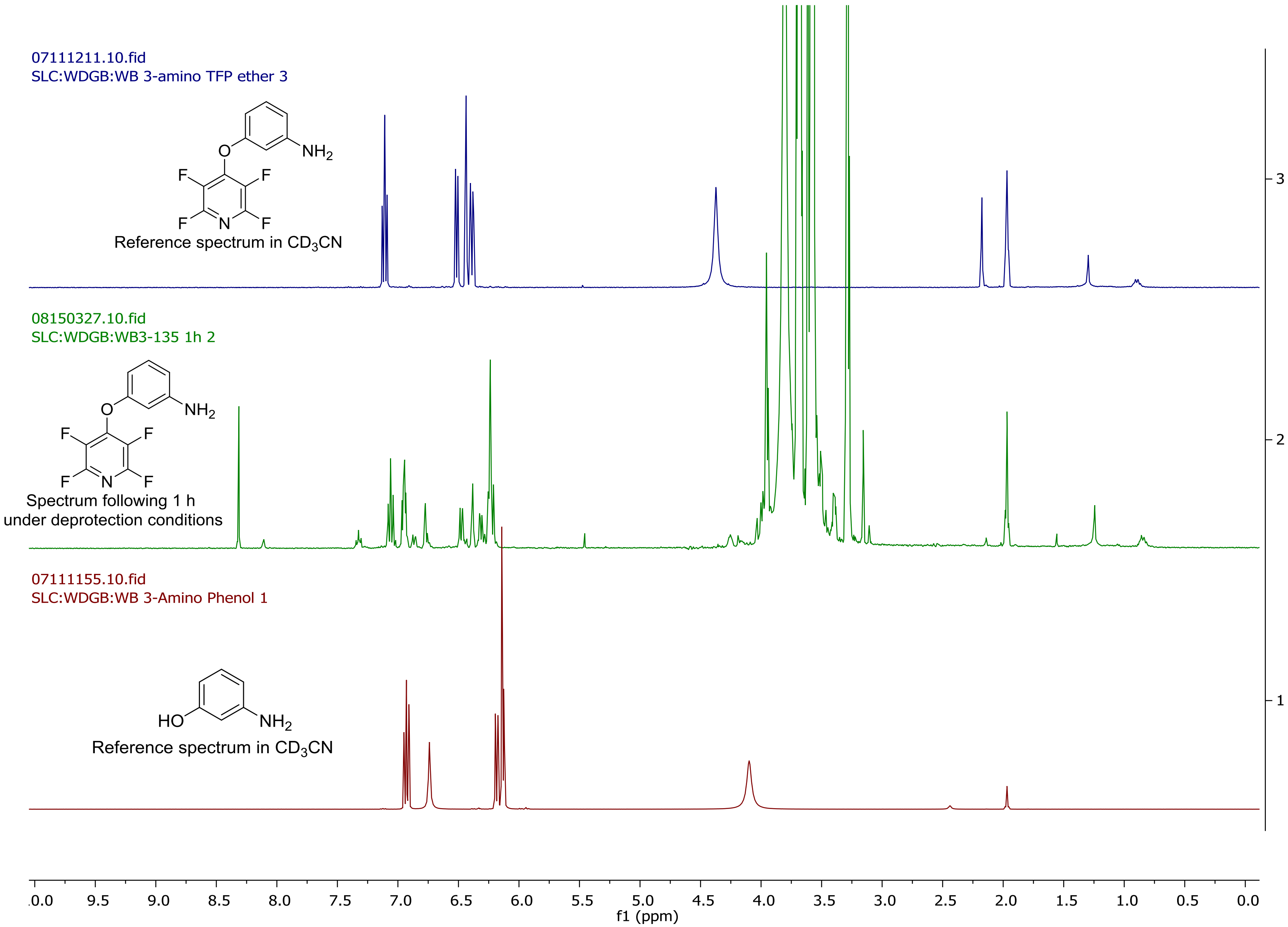
07111211.10.fid
SLC:WDGB:WB 3-amino TFP ether 3
Reference spectrum in CD_3CN
08150327.10.fid
SLC:WDGB:WB3-135 1h 2
Spectrum following 1 h
under deprotection conditions
07111155.10.fid
SLC:WDGB:WB 3-Amino Phenol 1
Reference spectrum in CD_3CN
f1 (ppm)

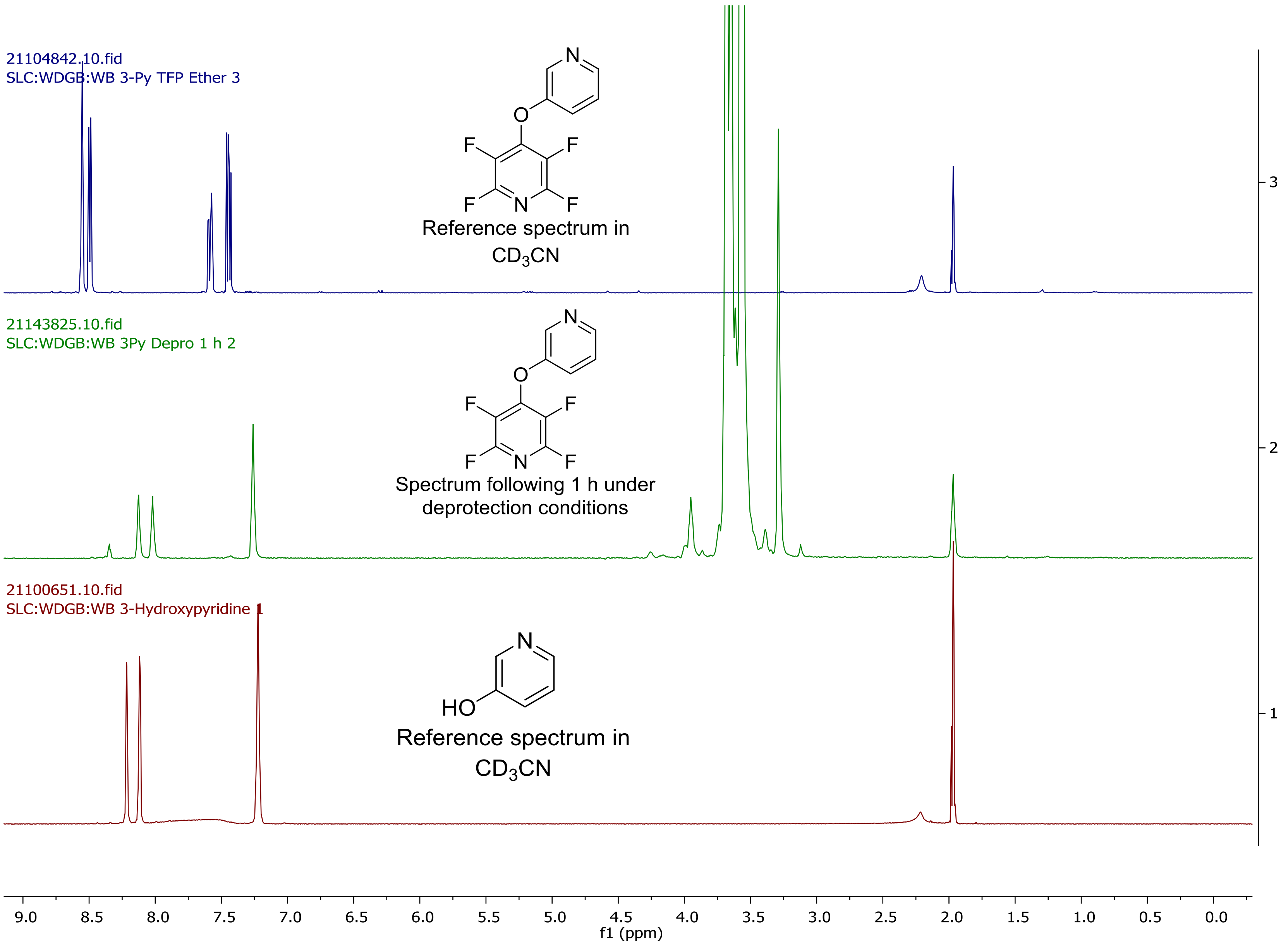
21104842.10.fid
SLC:WDGB:WB 3-Py TFP Ether 3
Reference spectrum in
CD3CN
21143825.10.fid
SLC:WDGB:WB 3Py Depro 1 h 2
Spectrum following 1 h under
deprotection conditions
21100651.10.fid
SLC:WDGB:WB 3-Hydroxypyridine 1
Reference spectrum in
CD3CN
f1 (ppm)

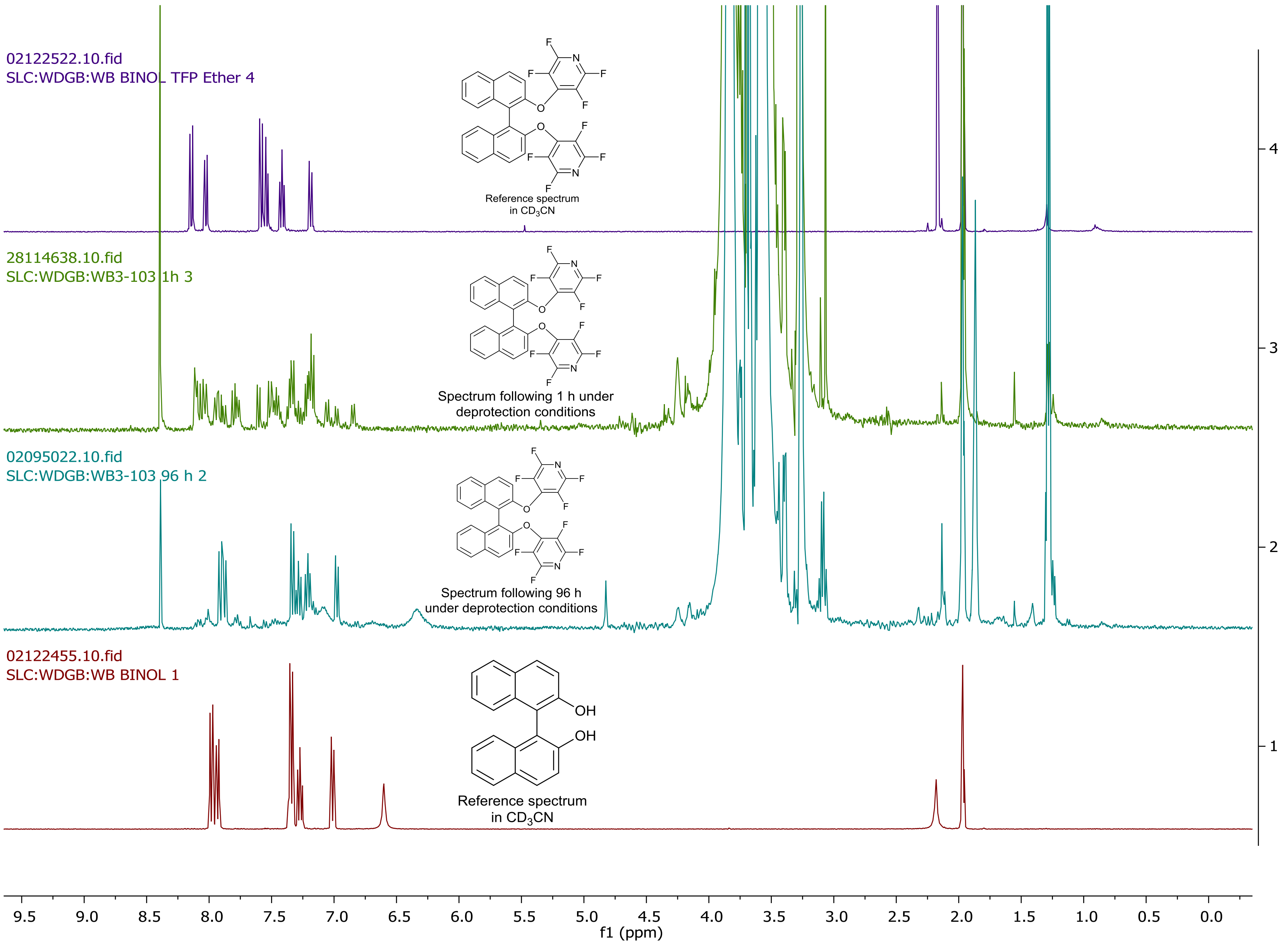

02122522.10.fid
SLC:WDGB:WB BINOL TFP Ether 4
Reference spectrum in CD_3CN
28114638.10.fid
SLC:WDGB:WB3-103 1h 3
Spectrum following 1 h under deprotection conditions
02095022.10.fid
SLC:WDGB:WB3-103 96 h 2
Spectrum following 96 h under deprotection conditions
02122455.10.fid
SLC:WDGB:WB BINOL 1
Reference spectrum in CD_3CN
f1 (ppm)

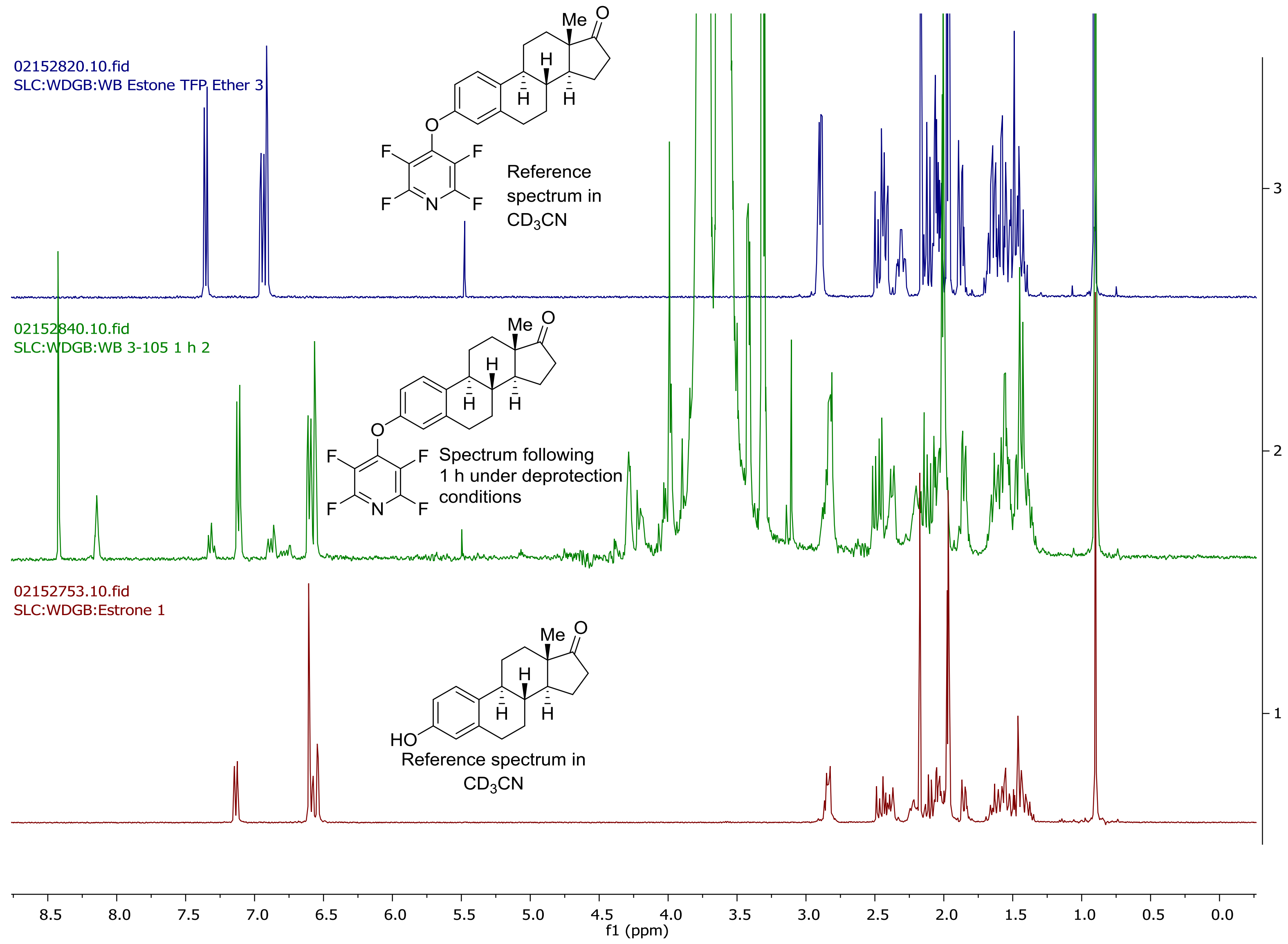

02152820.10.fid
SLC:WDGB:WB Estone TFP Ether 3
Reference spectrum in CD3CN
02152840.10.fid
SLC:WDGB:WB 3-105 1 h 2
Spectrum following 1 h under deprotection conditions
02152753.10.fid
SLC:WDGB:Estrone 1
Reference spectrum in CD3CN
f1 (ppm)

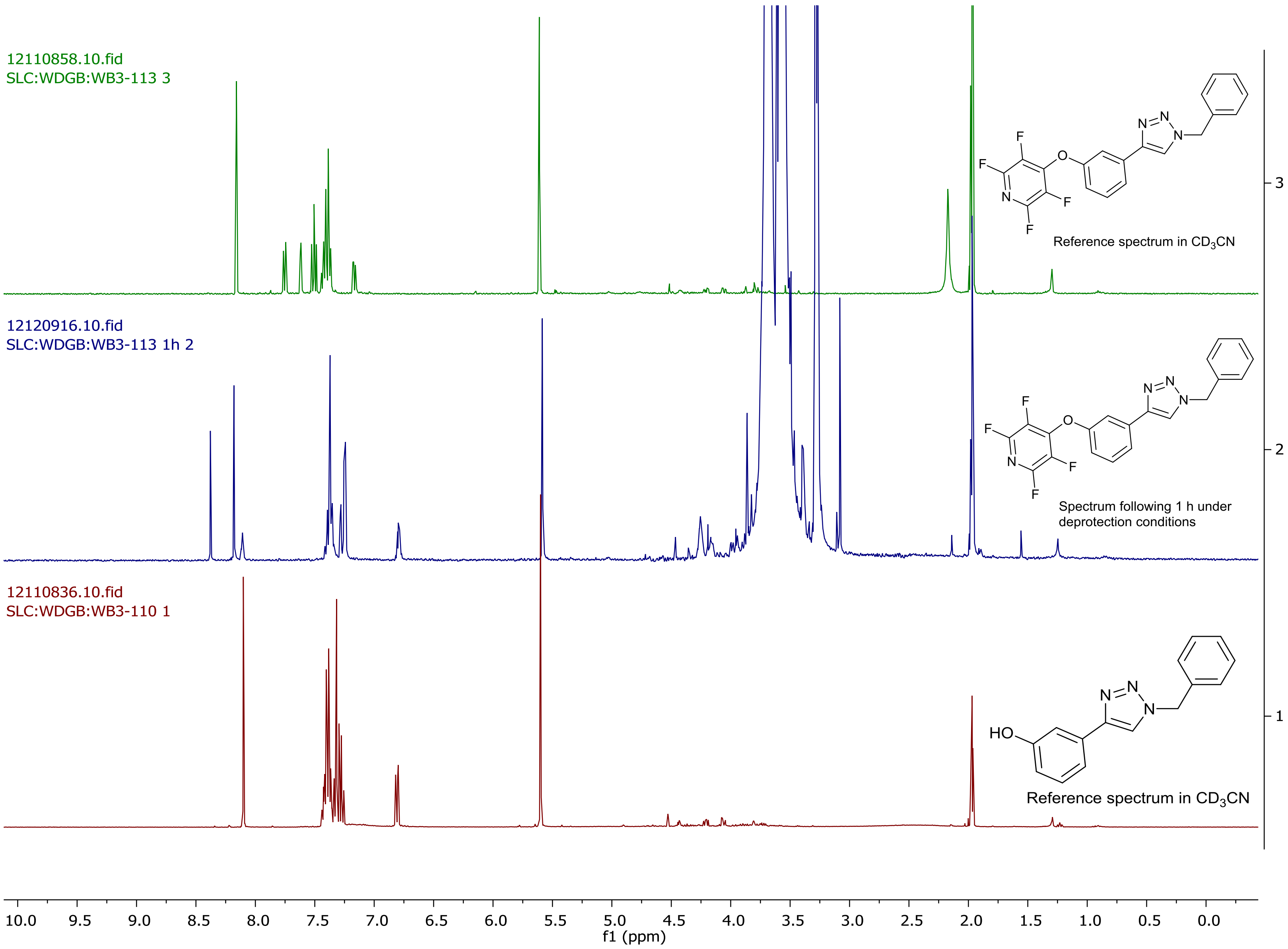

12110858.10.fid
SLC:WDGB:WB3-113 3
Reference spectrum in CD_3CN
12120916.10.fid
SLC:WDGB:WB3-113 1h 2
Spectrum following 1 h under deprotection conditions
12110836.10.fid
SLC:WDGB:WB3-110 1
HO
Reference spectrum in CD_3CN
f1 (ppm)

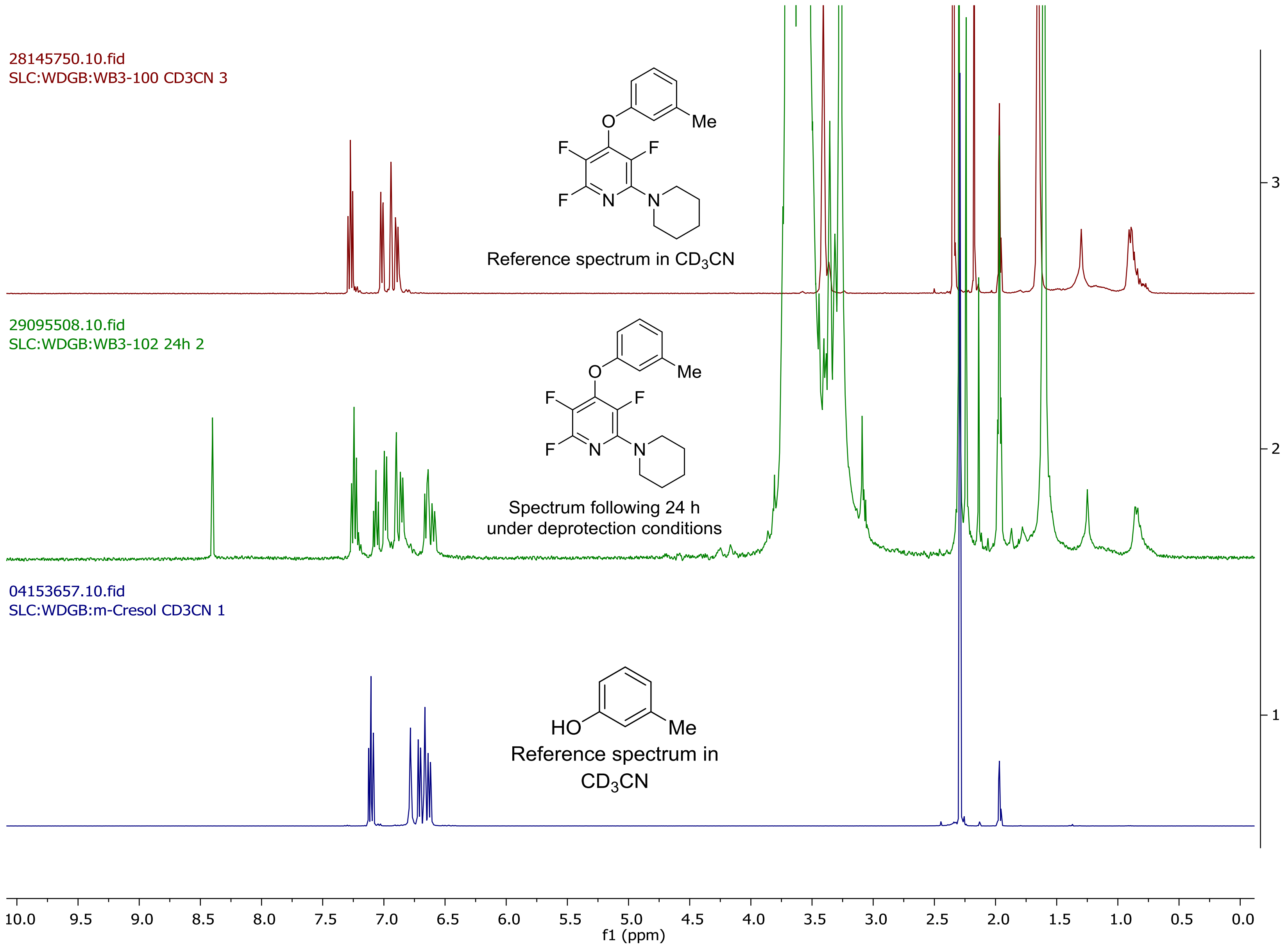

28145750.10.fid
SLC:WDGB:WB3-100 CD3CN 3
Me
O
F
F
F
N
N
Reference spectrum in CD_3CN
29095508.10.fid
SLC:WDGB:WB3-102 24h 2
Me
O
F
F
F
N
N
Spectrum following 24 h
under deprotection conditions
04153657.10.fid
SLC:WDGB:m-Cresol CD3CN 1
HO
Me
Reference spectrum in
CD_3CN
3
2
1
10.0 9.5 9.0 8.5 8.0 7.5 7.0 6.5 6.0 5.5 5.0 4.5 4.0 3.5 3.0 2.5 2.0 1.5 1.0 0.5 0.0
f1 (ppm)

NMR Spiking Experiments

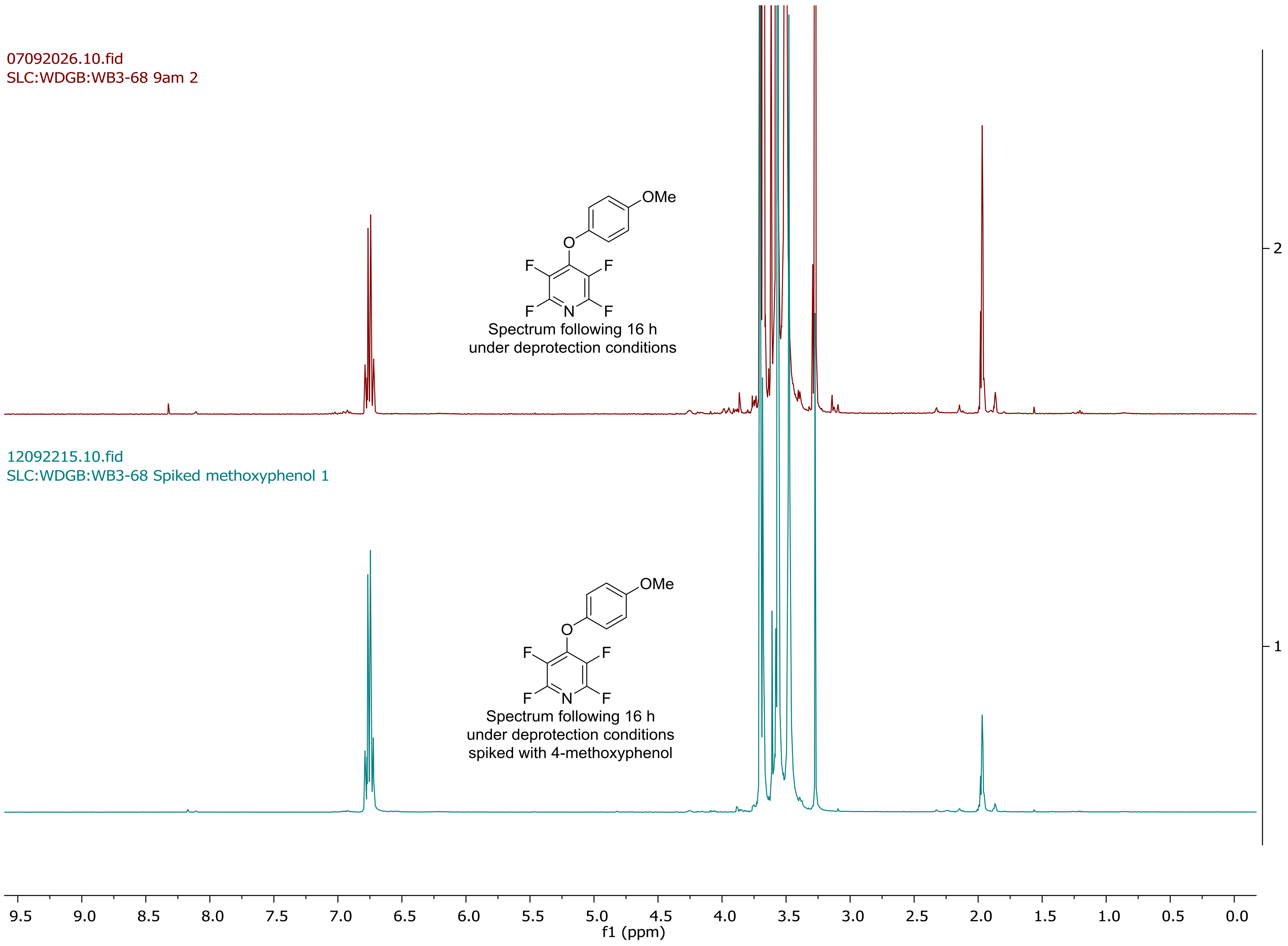
07092026.10.fid
SLC:WDGB:WB3-68 9am 2
OMe
O
F
F
F
N
F
Spectrum following 16 h
under deprotection conditions
12092215.10.fid
SLC:WDGB:WB3-68 Spiked methoxyphenol 1
OMe
O
F
F
F
N
F
Spectrum following 16 h
under deprotection conditions
spiked with 4-methoxyphenol
2
1
9.5 9.0 8.5 8.0 7.5 7.0 6.5 6.0 5.5 5.0 4.5 4.0 3.5 3.0 2.5 2.0 1.5 1.0 0.5 0.0
f1 (ppm)

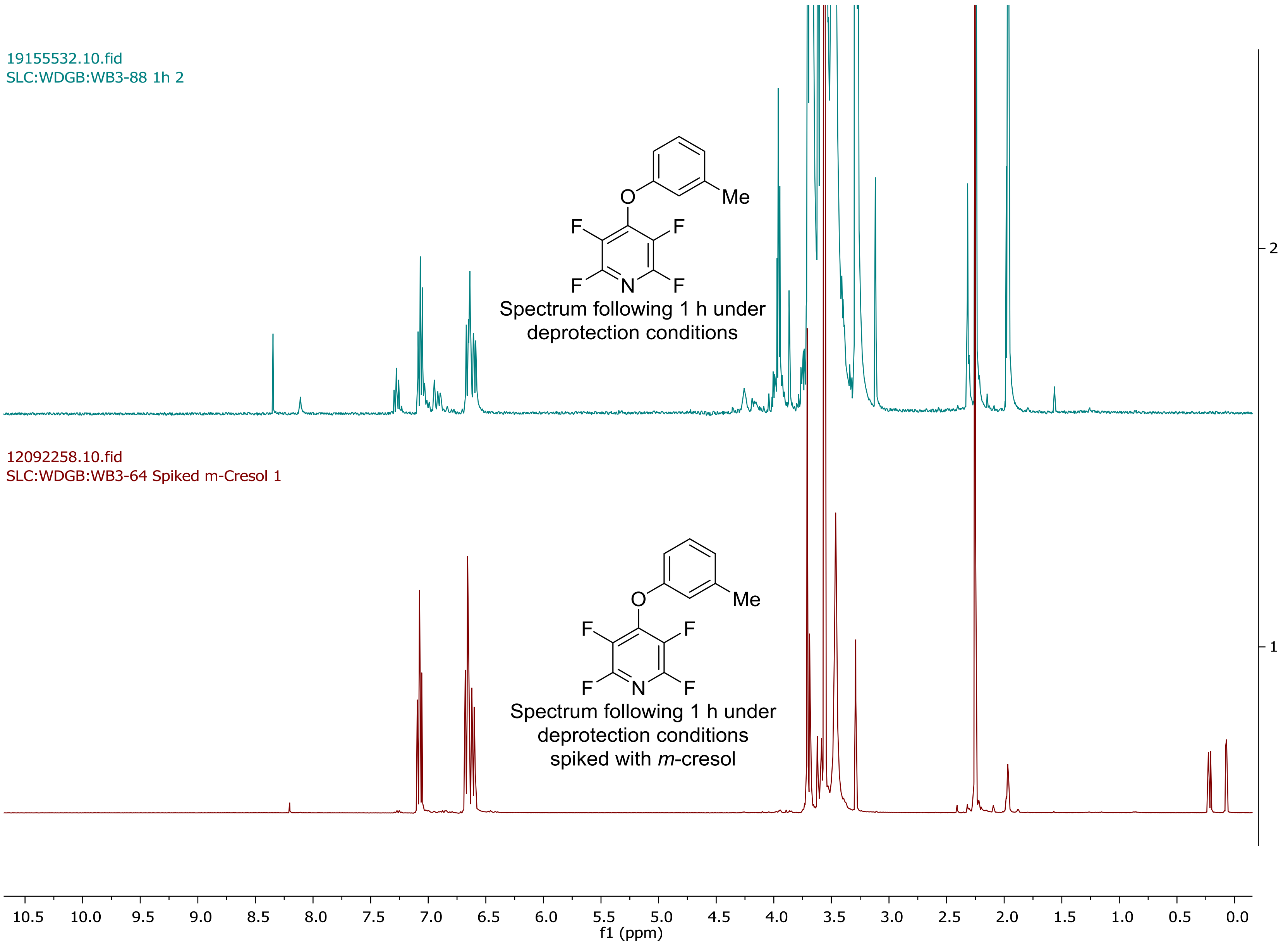
19155532.10.fid
SLC:WDGB:WB3-88 1h 2
Me
O
F
F
F
N
F
Spectrum following 1 h under deprotection conditions
12092258.10.fid
SLC:WDGB:WB3-64 Spiked m-Cresol 1
Me
O
F
F
F
N
F
Spectrum following 1 h under deprotection conditions spiked with m-cresol
2
1
10.5
10.0
9.5
9.0
8.5
8.0
7.5
7.0
6.5
6.0
5.5
5.0
4.5
4.0
3.5
3.0
2.5
2.0
1.5
1.0
0.5
0.0
f1 (ppm)

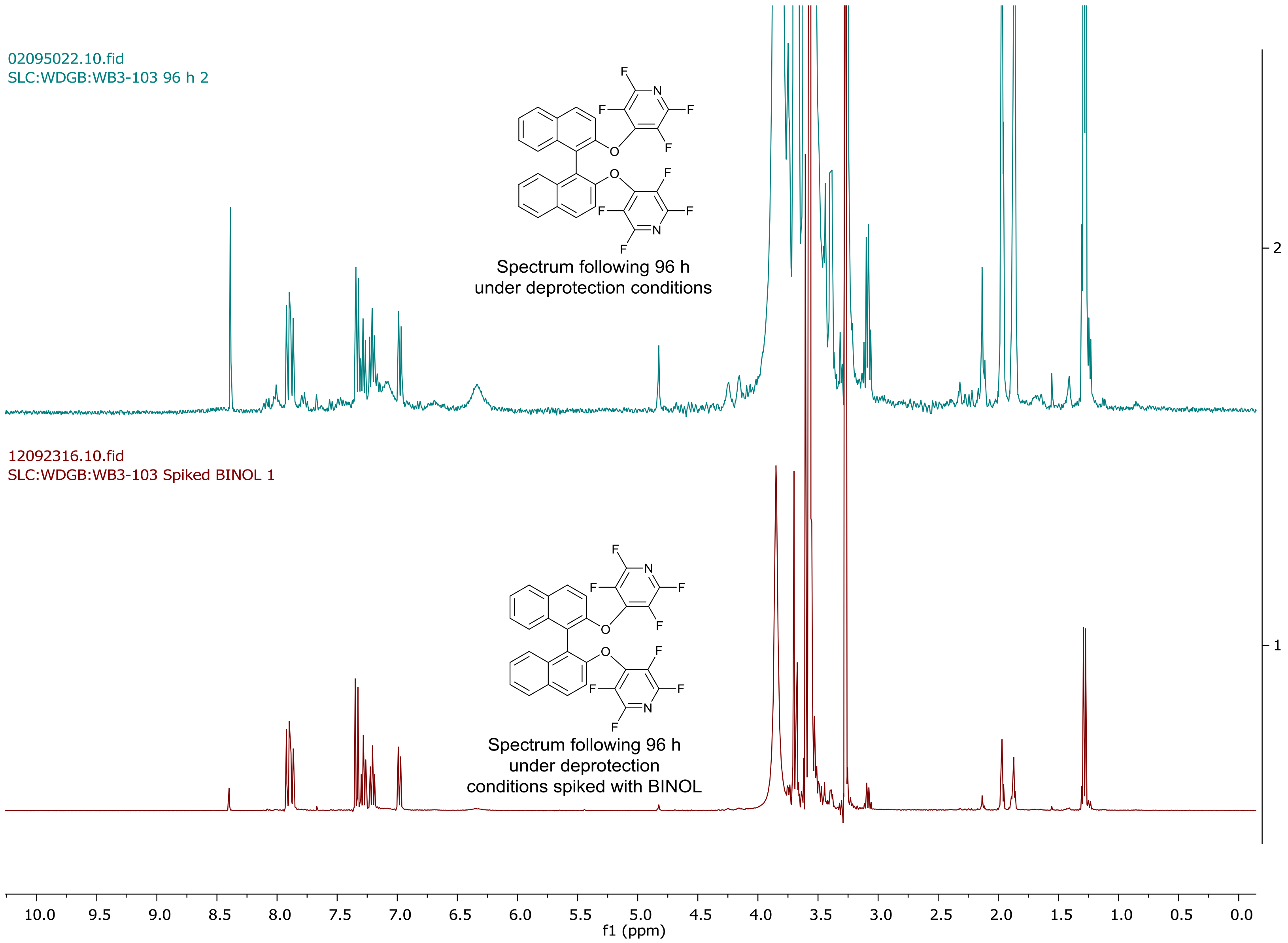
02095022.10.fid
SLC:WDGB:WB3-103 96 h 2
Spectrum following 96 h
under deprotection conditions
12092316.10.fid
SLC:WDGB:WB3-103 Spiked BINOL 1
Spectrum following 96 h
under deprotection
conditions spiked with BINOL
10.0 9.5 9.0 8.5 8.0 7.5 7.0 6.5 6.0 5.5 5.0 4.5 4.0 3.5 3.0 2.5 2.0 1.5 1.0 0.5 0.0
f1 (ppm)
2
1

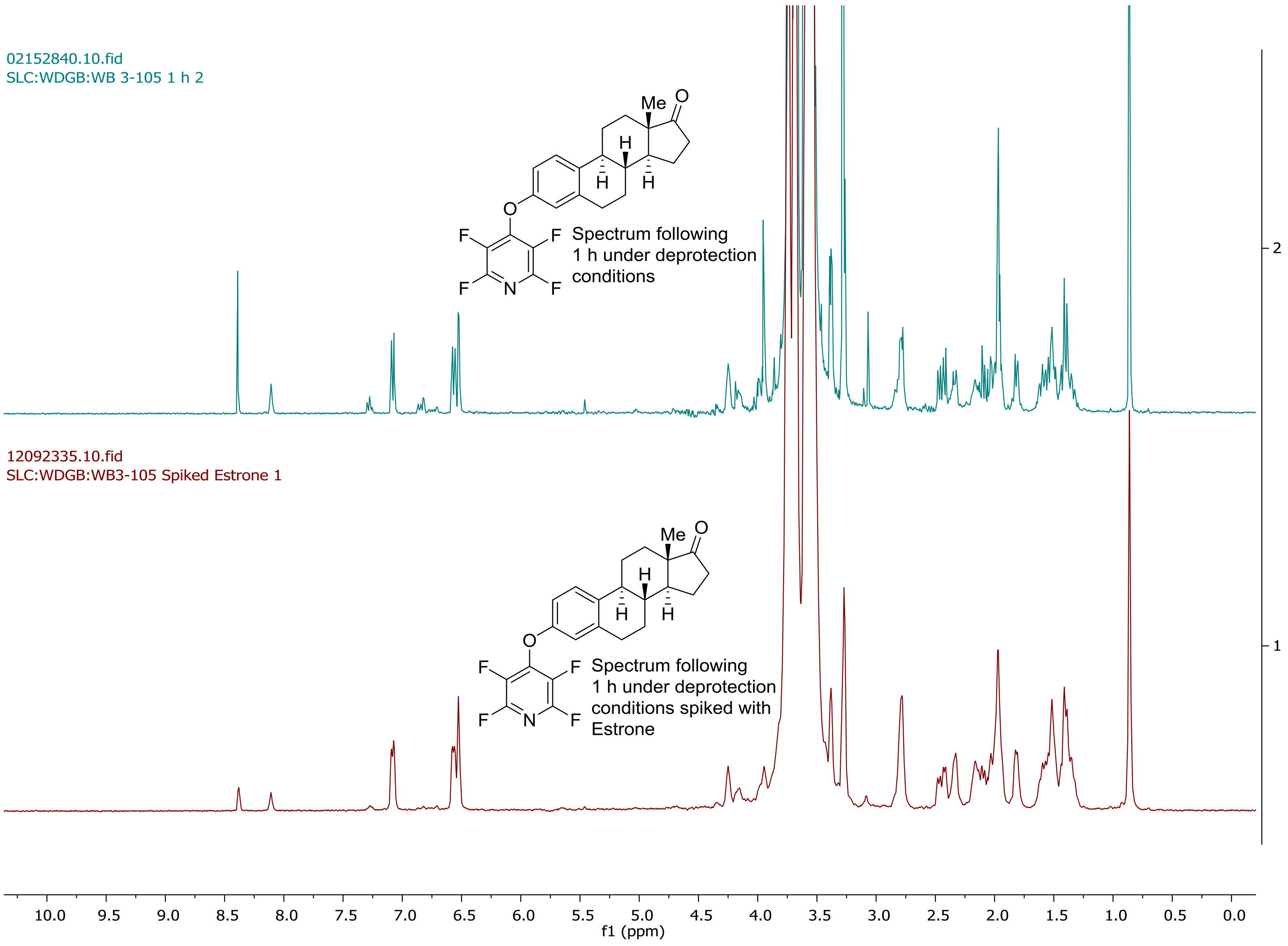

02152840.10.fid
SLC:WDGB:WB 3-105 1 h 2
Me
O
H
H
H
O
F
F
F
N
F
Spectrum following
1 h under deprotection
conditions
12092335.10.fid
SLC:WDGB:WB3-105 Spiked Estrone 1
Spectrum following
1 h under deprotection
conditions spiked with
Estrone
2
1
10.0 9.5 9.0 8.5 8.0 7.5 7.0 6.5 6.0 5.5 5.0 4.5 4.0 3.5 3.0 2.5 2.0 1.5 1.0 0.5 0.0
f1 (ppm)

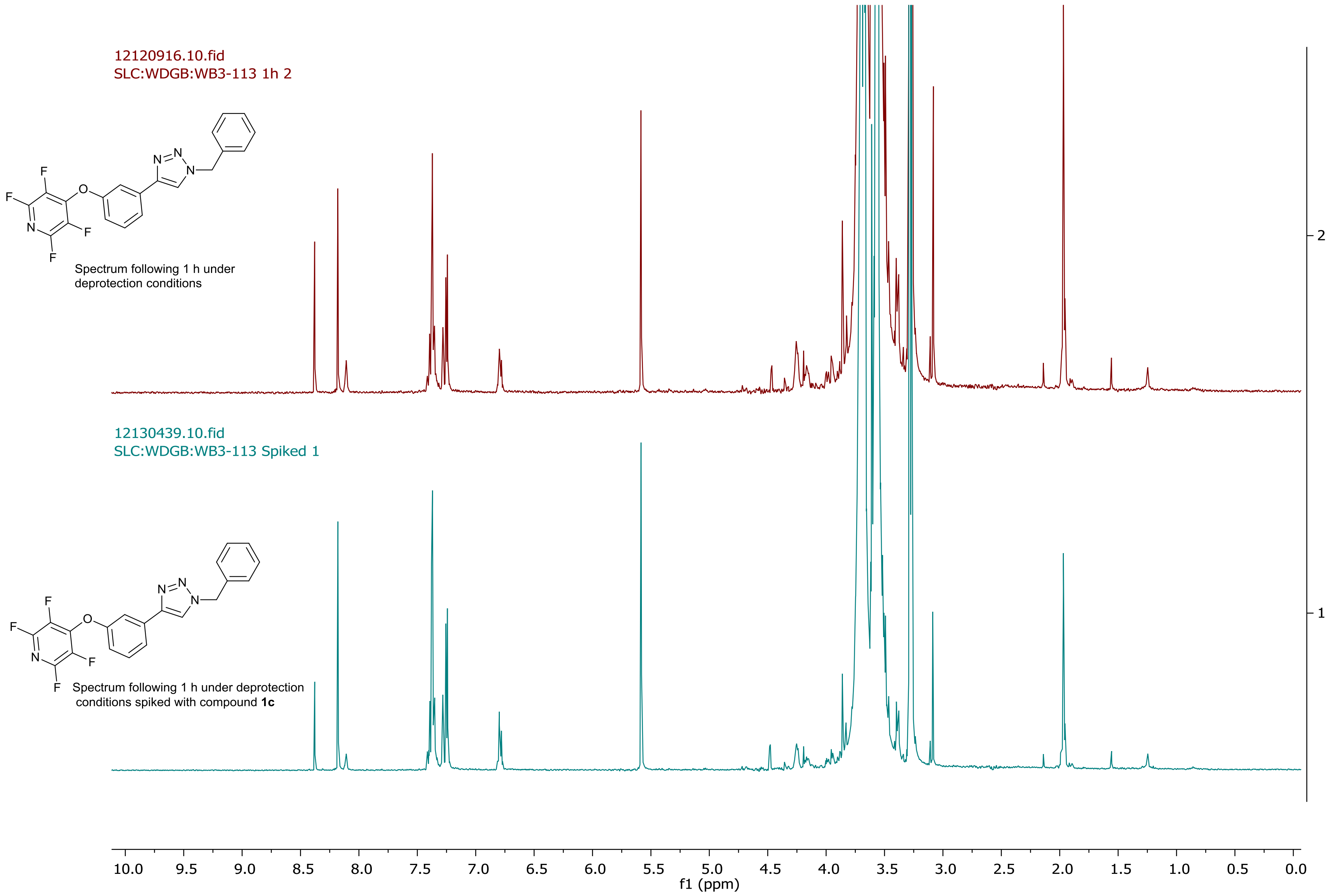
12120916.10.fid
SLC:WDGB:WB3-113 1h 2
Spectrum following 1 h under deprotection conditions
12130439.10.fid
SLC:WDGB:WB3-113 Spiked 1
Spectrum following 1 h under deprotection conditions spiked with compound 1c
10.0 9.5 9.0 8.5 8.0 7.5 7.0 6.5 6.0 5.5 5.0 4.5 4.0 3.5 3.0 2.5 2.0 1.5 1.0 0.5 0.0
f1 (ppm)
2
1

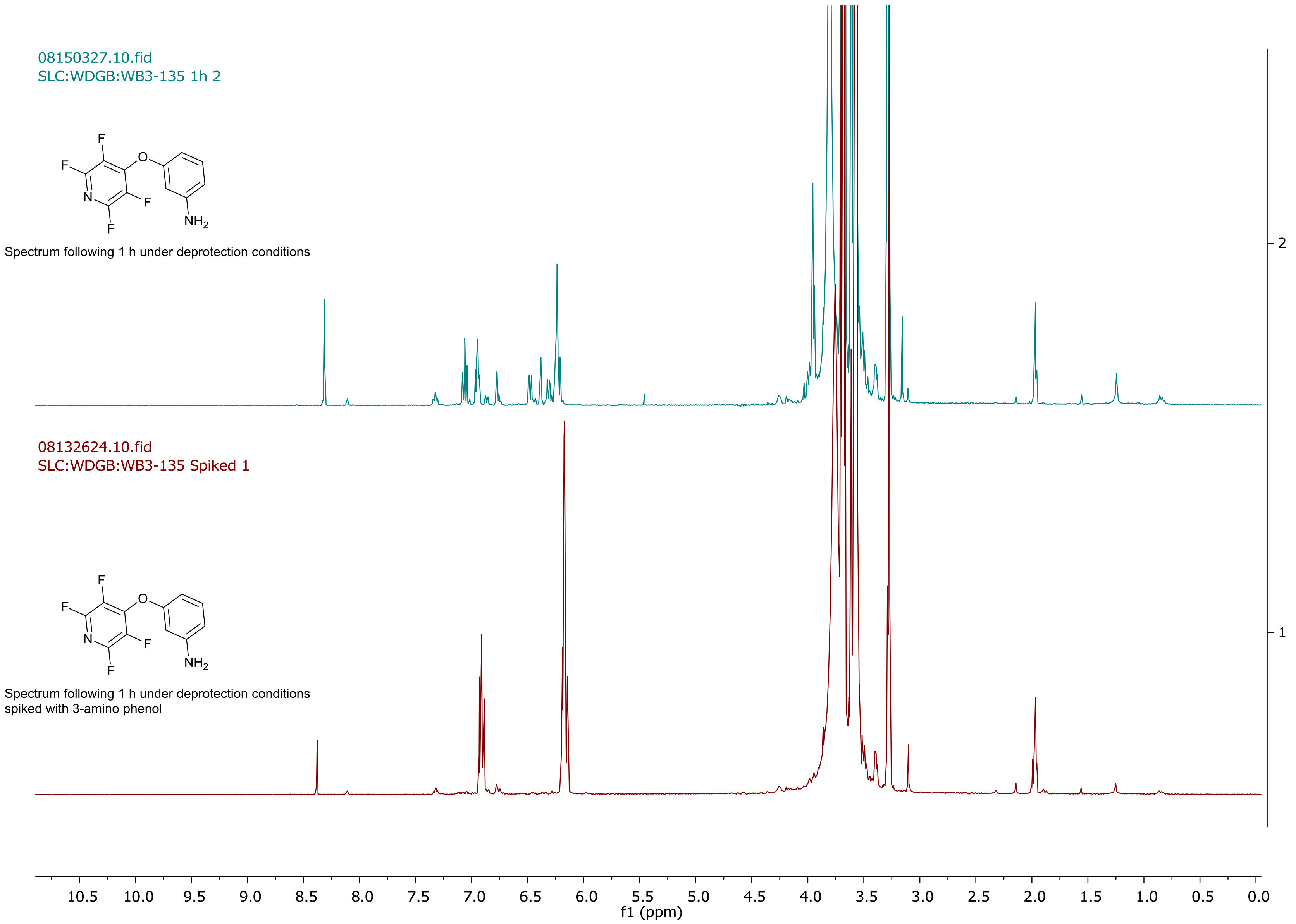
08150327.10.fid
SLC:WDGB:WB3-135 1h 2
F
F
O
N
F
F
NH2
Spectrum following 1 h under deprotection conditions
08132624.10.fid
SLC:WDGB:WB3-135 Spiked 1
Spectrum following 1 h under deprotection conditions
spiked with 3-amino phenol
2
1
10.5 10.0 9.5 9.0 8.5 8.0 7.5 7.0 6.5 6.0 5.5 5.0 4.5 4.0 3.5 3.0 2.5 2.0 1.5 1.0 0.5 0.0
f1 (ppm)

Mass Spectrometry of Reaction Mixture

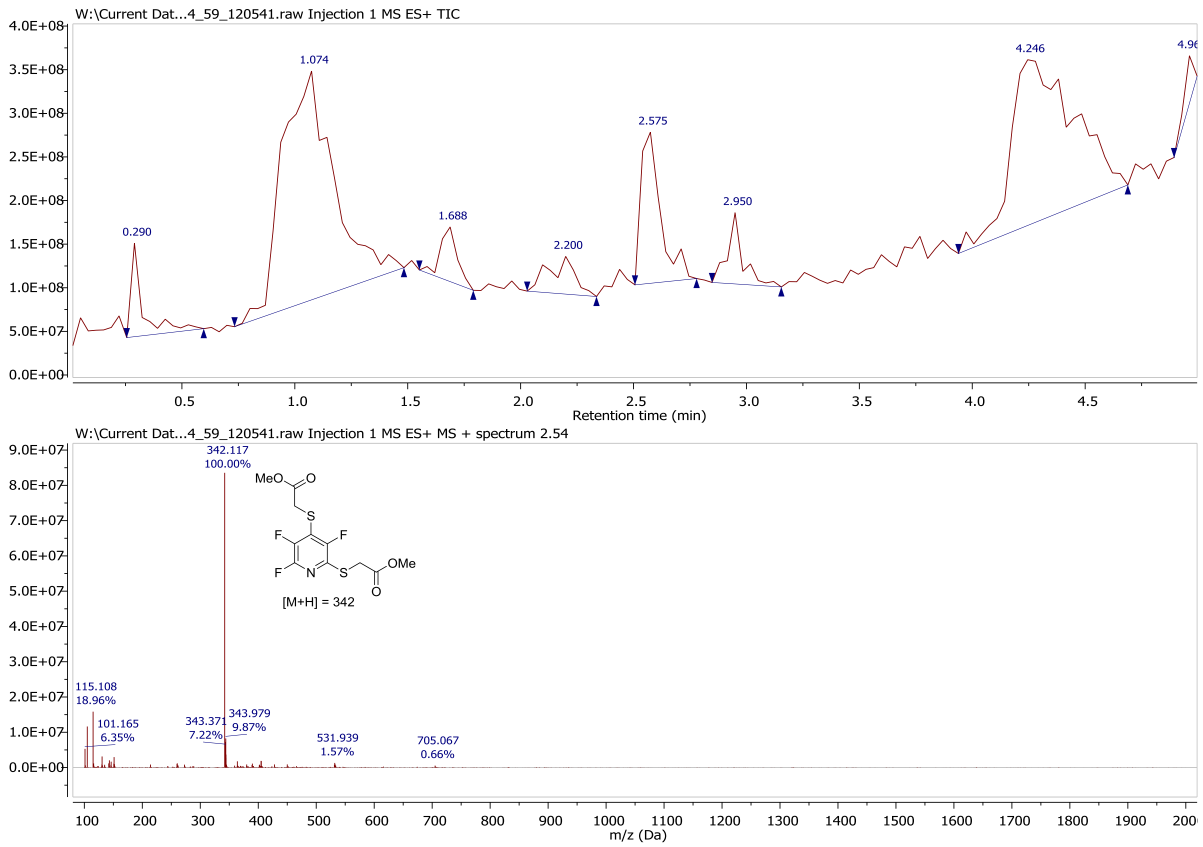

X-ray Crystallography

The X-ray single crystal data for **2r** and **2i** have been collected using λMoKα radiation (λ =0.71073Å) on a Bruker D8Venture diffractometer (Photon100 CMOS detector, IμS-microsource, focusing mirrors) equipped with a Cryostream (Oxford Cryosystems) open-flow nitrogen cryostats at the temperature 120.0(2) K. Both structures were solved by direct method and refined by full-matrix least squares on F^2 for all data using Olex2 [1] and SHELXTL [2] software. All non-hydrogen atoms were refined anisotropically, hydrogen atoms in structure **2r** were refined isotropically, while in the structure **2i** they were placed in the calculated positions and refined in riding mode. Crystal data and parameters of refinement are listed in Table SX. Crystallographic data for the structure have been deposited with the Cambridge Crystallographic Data Centre as supplementary publication CCDC-1856218-

1856219. Copies of the data can be obtained, free of charge, on application to CCDC, 12 Union Road, Cambridge CB2 1EZ, UK, (fax: +44 1223 336033 or e-mail:deposit@ccdc.cam.ac.uk).

1.O. V. Dolomanov, L. J. Bourhis, R. J. Gildea, J. A. K. Howard and H. Puschmann, J. *Appl. Cryst.* (2009), **42**, 339-341.
2. G.M. Sheldrick, *Acta Cryst.* (2008), **A64**, 112-122

*Table S3. Crystal data and structure refinement for **2q** and **2i**.*

Identification code	**2r**	**2i**
Empirical formula	$C_{30}H_{12}F_8N_2O_2$	$C_{11}H_4F_4N_2O_3$
Formula weight	584.42	288.16
Crystal system	monoclinic	monoclinic
Space group	$P2_1/c$	$P2_1/c$
a/Å	13.2804(5)	7.4591(17)
b/Å	8.7572(4)	10.786(3)
c/Å	21.1478(8)	26.797(6)
β/°	106.2235(15)	91.012(6)
Volume/Å^3	2361.53(17)	2155.6(9)
Z	4	8
ρ_{calc}g/cm^3	1.644	1.776
μ/mm^{-1}	0.147	0.175
F(000)	1176.0	1152.0
Reflections collected	49176	26864
Independent reflections, $R_{int,}$ Rσ	6565, 0.0333, 0.0211	5207, 0.1252, 0.1388
Data/restraints/parameters	6565/0/427	5207/0/361
Goodness-of-fit on F^2	1.047	1.010
Final R_1 indexes [I≥2σ (I)]	0.0384	0.0662
Final wR_2 indexes [all data]	0.1017	0.1393
Largest diff. peak/hole / e Å^{-3}	0.38/-0.25	0.28/-0.36

References

1. Y. Xiao, Y. Xu, H.-S. Cheon and J. Chae, *J. Org. Chem.*, 2013, **78**, 5804-5809.
2. L. Tianfei, S. Xinxin, W. Yaming and S. Qilong, *Angew. Chem. Int. Ed.*, 2012, **51**, 540-543.
